# Modulating stereoselectivity in allylic C(sp^3^)-H bond arylations via nickel and photoredox catalysis

**DOI:** 10.1038/s41467-023-36103-0

**Published:** 2023-02-01

**Authors:** Long Huang, Marcin Szewczyk, Rajesh Kancherla, Bholanath Maity, Chen Zhu, Luigi Cavallo, Magnus Rueping

**Affiliations:** 1 Institute of Organic Chemistry, RWTH Aachen University, Landoltweg 1, 52074 Aachen, Germany; 2 KAUST Catalysis Center (KCC), King Abdullah University of Science and Technology (KAUST), Thuwal, 23955-6900 Saudi Arabia; 3 Institute for Experimental Molecular Imaging, RWTH Aachen University, 52074 Aachen, Germany

**Keywords:** Stereochemistry, Photocatalysis, Catalytic mechanisms, Synthetic chemistry methodology

## Abstract

While significant progress has been made in developing selective C-H bond cross-couplings in the field of radical chemistry, the site and stereoselectivity remain a long-standing challenge. Here, we present the successful development of stereodivergent allylic C(sp^3^)-H bond arylations through a systematic investigation of the direction and degree of stereoselectivity in the cross-coupling process. In contrast to the signature photosensitized geometrical isomerization of alkenes, the catalytic reaction demonstrates the feasibility of switching the C-C double bond stereoselectivity by means of ligand control as well as steric and electronic effects. Computational studies explain the stereochemical outcome and indicate that excitation of a Ni-allyl complex from singlet to a triplet state results in a spontaneous change of the allyl group coordination and that the subsequent isomerization can be directed by the choice of the ligand to achieve *E*/*Z* selectivity.

## Abstract (web summary)

The selective construction of C-C double bonds in alkenes, which can be one of two isomers, is a fundamental focus of organic chemistry. Here, the authors systematically investigated the stereoselectivity in allylic C(sp3)-H bond arylations and demonstrated a ligand switch could allow a stereodivergent synthesis of silyl enol ethers.

## Introduction

Alkenes occur widely in nature and are feedstocks in various fields of the chemical industry, ranging from the vast scale manufacture of plastics and detergents to the synthesis of pharmaceutical intermediates and fragrances^1^. The selective construction of C–C double bonds in alkenes, either the *E* or the higher energetic *Z* isomer, has long been one of the fundamental yet elusive topics in the advancement of organic chemistry. In this regard, classical carbonyl olefination, elimination reaction and alkyne addition reaction are commonly used, albeit with the loss of valuable functionalities^2^. On the other hand, the ideal and most straightforward way is the direct functionalization of pre-existing alkenes. In the past decades, a variety of catalytic methodologies, including olefin metathesis, Heck-type cross-coupling and alkene isomerization have emerged as attractive approaches for the convergent synthesis of stereo-defined alkenes^3,4^. Of these approaches, methods based on transition-metal catalyzed alkene positional and geometrical isomerization were developed to selectively access isomers through an energetically net “downhill” process^5^, whereas photochemical geometrical isomerization has offered a route in the opposite direction but with the limitation only being suitable for chromophoric substrates^6–9^. Despite these advances, there are no corresponding transformations that are broadly applicable to the site-selective and stereodivergent preparation of multi-substituted alkenes under mild conditions. The quest for novel and selective methodologies remains a subject of intensive study.

In recent years, visible-light photocatalysis enabled C–H bond functionalization has received widespread attention^10–15^. One of the most recent prominent advances is photoredox nickel dual catalysis enabled C–H bond cross-coupling. Within this subdomain of metallaphotoredox catalysis^16,17^, two fundamentally distinct pathways have been utilized for the generation of reactive open-shell species^18^ from native C–H functionality: (1) direct oxidative single-electron transfer (SET) between substrates and excited state of photocatalyst^19,20^ and (2) hydrogen atom transfer (HAT) of native sp^3^ C–H bonds by appropriate photo-generated N, O, S, X (Cl, Br) radicals or photoexcited polyoxometalate species^21–31^. As a result, a very diverse class of substrates such as ethers, amides, alcohols, aldehydes, alkenes, and alkanes, have been successfully employed in the coupling with aryl or alkyl halides to create valuable C(sp^2^)-C(sp^3^) or C(sp^3^)-C(sp^3^) bonds^32,33^. Although significant effort has been dedicated to controlling site-selectivity among these developments, very few examples are associated with the controlling of stereochemistry^34–36^. Specifically, methods concerning the stereoselective preparation of multi-substituted alkenes in either *E* or *Z* isomeric forms are still not known. Our continuous interest in photoredox-enabled allylic C–H bond functionalization prompted us to develop a general method that can selectively give the alkenes in either *E* or *Z* configuration^37,38^.

**Fig. 1:**
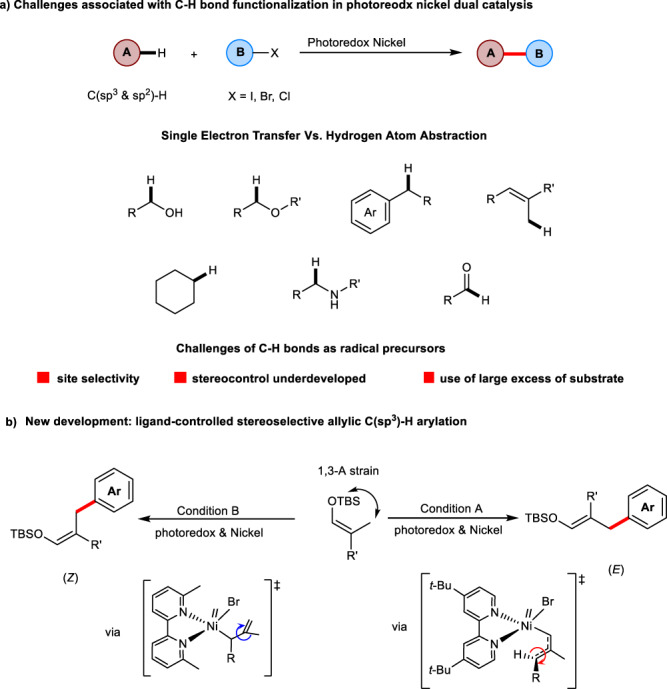
Reaction development. **a** Current developments in photoredox nickel synergistic catalysis enabled C–H bond functionalization. **b** our initial design and development of a stereodivergent arylation.

Herein, we demonstrate a highly stereoselective allylic C(sp^3^)-H bond cross-coupling of alkenes through the synergistic combination of photoredox and nickel catalysis (Fig. 1)^39^.

## Results

### Rational design and optimization

We initially tried to identify suitable reaction conditions that are effective for allylic C(sp^3^)-H cross-couplings of silyl enol ethers. We also like to highlight an important recent study by Studer and co-workers, who accomplished an efficient C(sp^3^)-H functionalization of enol ethers^40^. The investigation began with the coupling of silyl enol ether **1** and methyl 4-bromobenzoate (**2**). After considerable efforts, we were delighted to see that with readily available reagents, including photocatalyst Ir[dF(CF_3_)ppy]_2_(dtbbpy)PF_6_ (1 mol%), NiCl_2_•DME (10 mol%), 4,4’-di-*tert*-butylbipyridine (dtbbpy) (15 mol%) and 2,6-lutidine (1.5 equiv.) we were able to obtain the desired product in excellent yield (83%) with good *E* selectivity (88%). The reactions were carried out using blue light-emitting diodes (LEDs) at room temperature (Table 1). Control experiments showed that no detectable product was formed in the absence of light irradiation, ligand, base, nickel catalyst, or photocatalyst, showcasing the essential role of all components in this synergistic catalysis. Seeking to improve on the stereoselectivity, we next evaluated several classes of ligands and found that dtbbpy was the best choice of ligand for *E* selectivity. In the meantime, we were excited to find that a moderate *Z* selectivity (74%) was realized when 6,6’-dimethyl-2,2’-bipyridine (6,6’-dmbpy) was employed, albeit with low conversion. This observation of ligand effects prompted us to further examine the reaction conditions. After extensive evaluation of different reaction parameters (see Supporting Information), we successfully established two sets of reaction conditions allowing for a stereoselectivity switch: the use of dtbbpy (**L1**) led to *E* isomer **3** (93%) in 77% yield (condition **A**) and employing 6,6’-dmbpy (**L2**) gave *Z* isomer **4** in 66% yield (condition **B**) after isolation. To the best of our knowledge, the use of ligands to tune *E*/*Z* stereoselectivity in photoredox nickel dual catalysis has never been reported.

**Table 1:** Optimization of the reaction conditions^a^


Entry	Ni (mol%)	ligand	Yield (*E*/*Z*)^b^
1	NiCl_2_•DME (10)	**L1**	83% (88:12)
2	NiCl_2_•DME (10)	**L2**	27% (26:74)
Condition **A**^[c]^	NiBr_2_ (10)	**L1**	79% (93:7)
Condition **B**^[d]^	NiBr_2_ (20)	**L2**	69% (10:90)

^a^Unless otherwise specified, the reactions were performed on a 0.1 mmol scale under the following reaction conditions: **1** (3 equiv.), **2** (1 equiv.), 2,6-lutidine (1.5 equiv.), **PC-1** (1 mol%), Ni (10 mol%), ligand (15 mol%), dioxane (1 mL), rt, 48 h.

^b^Yield and *E*/*Z* ratio were determined by crude 1H NMR with reference to 1,3,5-trimethoxybenzene.

^c^THF/benzene 1:1 was used as solvent.

^d^20 mol% L2 and THF/dioxane 1:1 was used instead.

### Synthetic scope

**Fig. 2:**
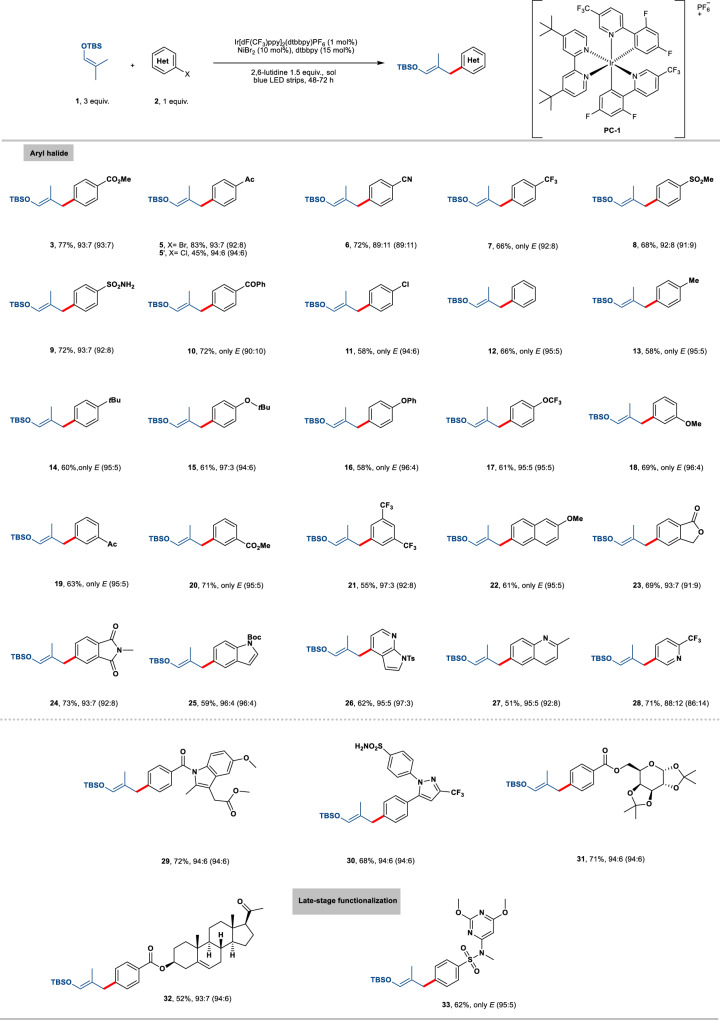
Scope for the formation of *E* isomer with aryl halides. Unless otherwise specified, the reactions were performed on a 0.2 mmol scale under the following reaction conditions: **1** (3 equiv.), aryl halide (1 equiv.), 2,6-lutidine (1.5 equiv.), PC-1 (1 mol%), NiBr_2_ (10 mol%), dtbbpy (15 mol%), benzene/THF 1:1 (2 mL), rt, 48-72 h, yields and *E*/*Z* after purification by chromatography (numbers in parentheses refer to *E*/*Z* before purification). Ac, acetyl group; TBS, *tert*-butyldimethylsilyl.

With these reaction conditions in hand, we first turned our attention to evaluating the electrophilic coupling partners for the scope of *E* isomer with condition **A**. As shown in Fig. 2, a wide range of electron-deficient functional groups (ketone, ester, trifluoromethyl, nitrile, sulfone, chloro, sulfonamide) at the *para* position on the aryl bromides were well-tolerated using this synergistic strategy and providing the corresponding products **3**–**11** in good yields (58-83%) and excellent *E* selectivities (89% to only *E* isomer). In addition, comparable good results could be obtained for both electron-neutral and electron-rich bromoarenes in contrast to their intrinsic difficulty in oxidative addition (**12**–**17**, 58-66% yield, 95% to only *E* isomer). Moreover, the installation of *meta*-monosubstituted or disubstituted functional groups on the aryl halide turned out to be successful (**18**–**21**, 55–71% yield), excellent levels of stereocontrol were archived regardless of their electronic nature (97% to only *E* isomer). However, it is noteworthy that the reaction failed with substrates bearing *ortho* substituents under current reaction conditions. Nearly quantitatively hydro-dehalogenated products were obtained instead. This can be attributed to steric bulkiness. Polycyclic bromoarenes derived from naphthalene, phthalide, and phthalimide performed very well in the reaction (**22**–**24**, 61–73% yield, 93% to only *E* isomer). Additionally, pharmaceutically important heteroaryl bromides bearing indole, azaindole, quinoline, and pyridine moieties proved to be competent coupling partners with this reaction manifold (**25**–**28**, 51–71% yield, 88–96% *E* isomer).

Having demonstrated the generality of this protocol for relatively simple substrates, we next set out to examine the synthetic potential for the late-stage cross-coupling of structurally diverse bioactive compounds. Aryl bromide electrophiles from indomethacin, sulfadimethoxine, celecoxib, diacetone-D-galactose, and pregnenolone were smoothly converted into their corresponding silyl enol ether (**29**–**33**, 52-72% yield, 93% to only *E* isomer). These results further highlight the robust functional group tolerance of the current development. Notably, only a trace amount of THF arylation adduct resulting from competitive solvent C(sp^3^)-H coupling was observed.

**Fig. 3:**
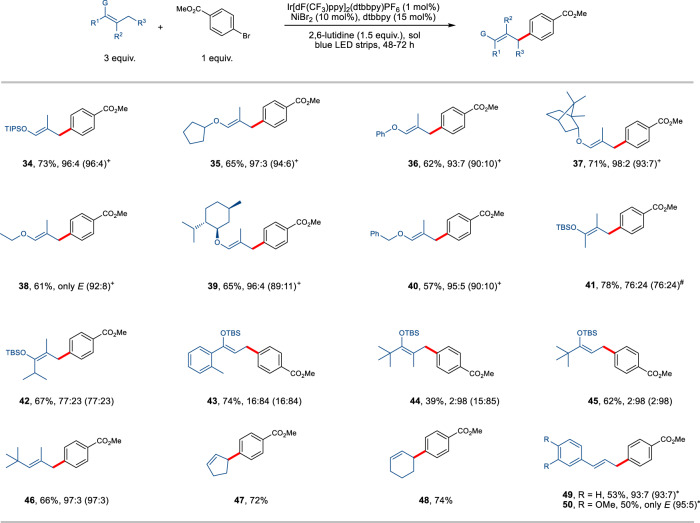
Scope of alkenes for the coupling with aryl bromide 2. Unless otherwise specified, the reactions were performed on a 0.2 mmol scale under the following reaction conditions: alkene (3 equiv.), aryl bromide (1 equiv.), 2,6-lutidine (1.5 equiv.), PC-1 (1 mol%), NiBr_2_ (10 mol%), dtbbpy (15 mol%), benzene/THF 1:1 (2 mL), rt, 48-72 h, yields and *E*/*Z* after purification by chromatography (numbers in parentheses refer to *E*/*Z* before purification). + *tert*-butyl acrylate (1 equiv.) was used as additive. TIPS, triisopropylsilyl. # Formal β-arylation of ketone was achieved after deprotection. *E/*Z* before isomerization, **49** (37:63), **50** (33:67), see Supporting Information for reaction condition details.

We next surveyed the scope of alkenes (Fig. 3) by increasing the steric bulkiness of the silyl group from TBS to TIPS, leading to comparable yield with higher level of *E* selectivity (**34**, 73% yield, 96% *E* isomer). High levels of stereoselectivity (93% to only *E* isomer) and good isolated yields (57–71%) were observed for a variety of enol ethers, as demonstrated by either cyclopentyl (**35**), phenyl (**36**), ethyl (**38**), benzyl (**40**) substituted or bulky ones from (-)-borneol (**37**) and (-)-menthol (**39**). If the size of the additional substituent in the α-position of silyl enol ether is increased, the stereoselectivity decreases, with lower *E* selectivity obtained for **41** (76%) and **42** (77%). The steric influence is high, and *Z* selectivity was achieved with substrates carrying *tert*-butyl and 2-methylphenyl substituents providing the corresponding products in good to excellent Z/E ratio (**43**–**45**, 84-98%). Similarly, excellent selectivity was observed in the case of steric-demanding alkene (**46**). Importantly, further investigation revealed that this method is not restricted to acyclic alkenes. Cyclic substrates have also been efficiently functionalized (**47** and **48**), showcasing the broad applicability of the current protocol. The cross-coupling can be further extended to styrenes, followed by a visible-light-induced *Z* to *E* isomerization. The corresponding styrene products were obtained in moderate yields and excellent *E* selectivity (**49** and **50**).

**Fig. 4:**
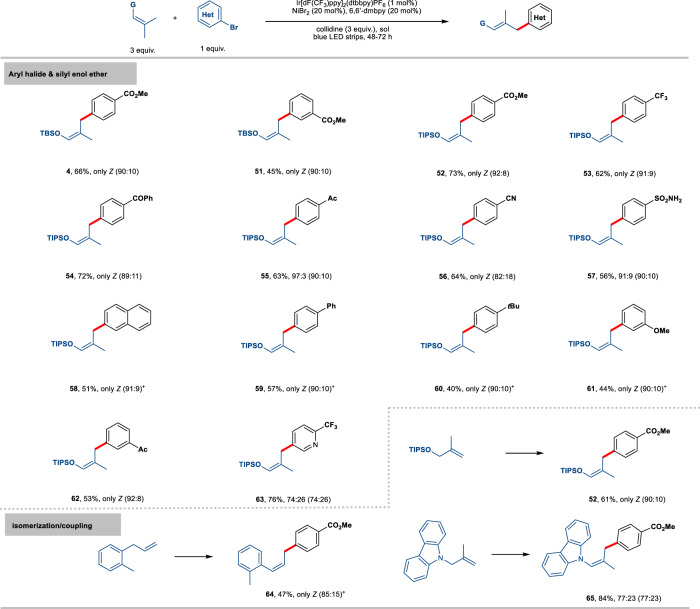
Scope for the formation of *Z* isomer with aryl halides and isomerization/cross-coupling. Unless otherwise specified, the reactions were performed on a 0.2 mmol scale under the following reaction conditions: alkene (3 equiv.), aryl halide (1 equiv.), 2,4,6-collidine (3 or 1.5 equiv.), **PC-1** (1 mol%), NiBr_2_ (20 mol%), 6,6’-dmbpy (20 mol%), dioxane/THF 1:1 (2 mL), rt, 48-72 h, yields and Z/E after purification by chromatography (numbers in parentheses refer to Z/E before purification). + two parallel runs in 0.1 mmol were carried out with LiBr (1 equiv.) as additive.

We subsequently pursued to examine the generality of condition **B** that could promote *Z* selective arylation of the allylic C(sp^3^)-H bonds. As outlined in Fig. 4, a broad range of electron-rich and electron-poor aryl bromides served as effective coupling partners. In all cases, the arylation reactions proceeded with excellent stereoselectivity under the standard conditions (**4**, **51**–**57**, 91% to only *Z* isomer). To achieve good efficiency for electron-rich substrates, the use of LiBr as an external bromine additive is crucial (**58** to **61**, 40–57% yield, only *Z* isomer). In contrast, only moderate *Z* selectivity can be obtained for heterocycle derived from pyridine (**63**, 74% *Z*). The superior reactivity of C2-substituted bidentate ligand in alkene isomerization^41–44^ prompted us to question whether the current protocol could be used to initiate the double bond positional isomerization, allowing for the subsequent regio- and stereoselective arylation reaction at the terminal position of the allylic system. With our optimized condition **B**, we were pleased to find that the synergistic catalytic mode allows the allylic silyl ether to smoothly participate in the desired isomerization/cross-coupling sequence while achieving high levels of stereocontrol (**52**, 61% yield, only *Z*). Furthermore, a commercially available allyl benzene derivative could also be employed, generating the corresponding arylation adduct **64** with a high degree of *Z* selectivity (only *Z*). The scope was further extended to an *N*-allyl-carbazole derivative, affording the desired product **65**. Lastly, the synthetic potential of this method was showcased in the stereospecific Simmon-Smith cyclopropanation of silyl enol ether (see Supporting Information).

### Mechanistic investigations

**Fig. 5:**
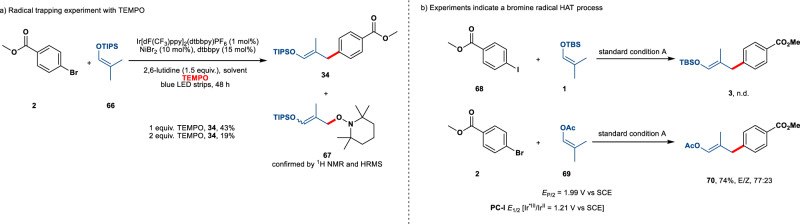
Preliminary mechanistic studies. **a** Radical trap experiment. **b** Mechanistic hints from using either vinyl acetate or aryl iodide as coupling components.

To investigate a plausible mechanism for the stereodivergent cross-coupling, we initially looked for direct evidence of allylic radical formation by introducing a well-known carbon radical scavenger TEMPO (2,2,6,6-tetramethyl-1-piperidinyloxy). The reaction efficiency diminished dramatically in the presence of the radical inhibitor; the yield dropped to only 19% when 2 equiv. TEMPO was used. The TEMPO adduct **67** was observed by ^1^H NMR of the crude reaction mixture and further confirmed by HRMS analysis. We next sought to understand the elementary steps that are closely associated with the allylic radical generation. Although (silyl) enol ethers are known to be electron-rich alkenes, cyclic voltammetry studies showed that the direct SET oxidation by the photoexcited **PC**–**1** (*E_1/2_ = 1.21 V vs. SCE in MeCN) should be difficult. The steady-state Stern-Volmer quenching study also revealed that the silyl enol ether does not quench the excited state of Ir photocatalyst (see Supporting Information). These findings preclude the generation of allylic radicals by the SET event. Moreover, aryl iodide **68** failed to be a competent coupling partner because of the weak hydrogen abstraction ability (Fig. 5b, H-I BDE = 71 kcal/mol)^45^. We therefore hypothesized a HAT process by bromine radical might be operative. Consistent with this hypothesis, we found that several representative olefins with very high oxidation potentials, cyclopentene (**47**), cyclohexene (**48**) and enol acetate (**70**) were suitable candidates for the cross-coupling.

**Fig. 6:**
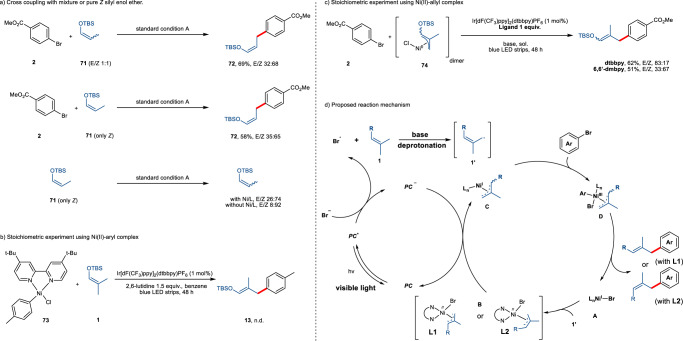
A proposed mechanism based on experimental investigation. **a** Experiments supporting the intermediacy of a nickel-allyl species. **b** Stoichiometric study with Ni(II)-Aryl complex. **c** Stoichiometric study with Ni(II)-Allyl complex. **d** Proposed reaction mechanism.

Employing an *E*/*Z* mixture of silyl enol ether **71** gave similar results as in the case of the *Z* isomer only, indicating the geometry loss of the double bond during the course of the reaction. Interestingly, we found that the *Z*-configured starting material in the latter case was converted into an *E/Z* mixture following a careful inspection of the reaction crude mixture. In a control experiment without nickel catalyst under the standard condition, the *Z* isomer remained to a high degree (92%) intact (Fig. 6a). These experiments suggest the possibility that the nickel-allyl species was involved in the reaction. We then carried out the stoichiometric experiment of aryl-Ni(II) complex **73** (derived from 4-methylchlorobenzene and dtbbpy) with **1**, no product of **13** was found (Fig. 6b). In comparison, the stoichiometric reaction of in-situ generated allylnickel(II) complex **74** produced the corresponding silyl enol ether product in moderate yields. More importantly, the stereoselectivity for product formation by choice of the ligand is consistent with the catalytic reaction, albeit in lower ratio (Fig. 6c). We also observed that the ratio of ligand to nickel could affect the stereoselectivity with dtbbpy ligand, and the nickel-allyl isomerization mechanism can be excluded given that the same *E* selectivity was obtained when the stoichiometric experiment was performed in the presence of Zn as reductant without light irradiation (see Supporting Information). Based on our mechanistic studies, a plausible mechanism is proposed (Fig. 6d). Upon irradiation with visible light, the Ir photocatalyst generates a long-lived triplet excited state, which should be able to oxidize the bromide anion to the active bromine radical. Subsequent hydrogen atom abstraction from the allylic position of alkene **1** yields the carbon-centered radical **1′**. In line with previous reports, this allyl radical is readily trapped by the doublet Ni(I)Br (**A**) to produce the Ni(II)(allyl)Br intermediate **B**. Single-electron reduction of **B** by the reduced state of the photocatalyst leads to the Ni(I)(allyl) complex **C**, which undergoes oxidative addition of the aryl bromide to produce the corresponding Ni(III)(allyl)(aryl)Br complex **D**. The catalytic cycle ends with the facile reductive elimination of the desired cross-coupling product and concurrent regeneration of the active catalyst **A**.

In support of the above control experiments and our proposed reaction mechanism, we performed DFT calculations (see Computational Details in Supporting Information) to define the catalytic mechanism. Possible pathways of photocatalytic cycles are discussed in Supplementary Figs. 15 and 16. Our results show that the oxidative photoredox cycle is favored during the formation of active Ni-complex from the pre-catalyst NiBr_2_. On the other hand, the reductive cycle is favored in the case of the Ni-catalytic pathway.

**Fig. 7:**
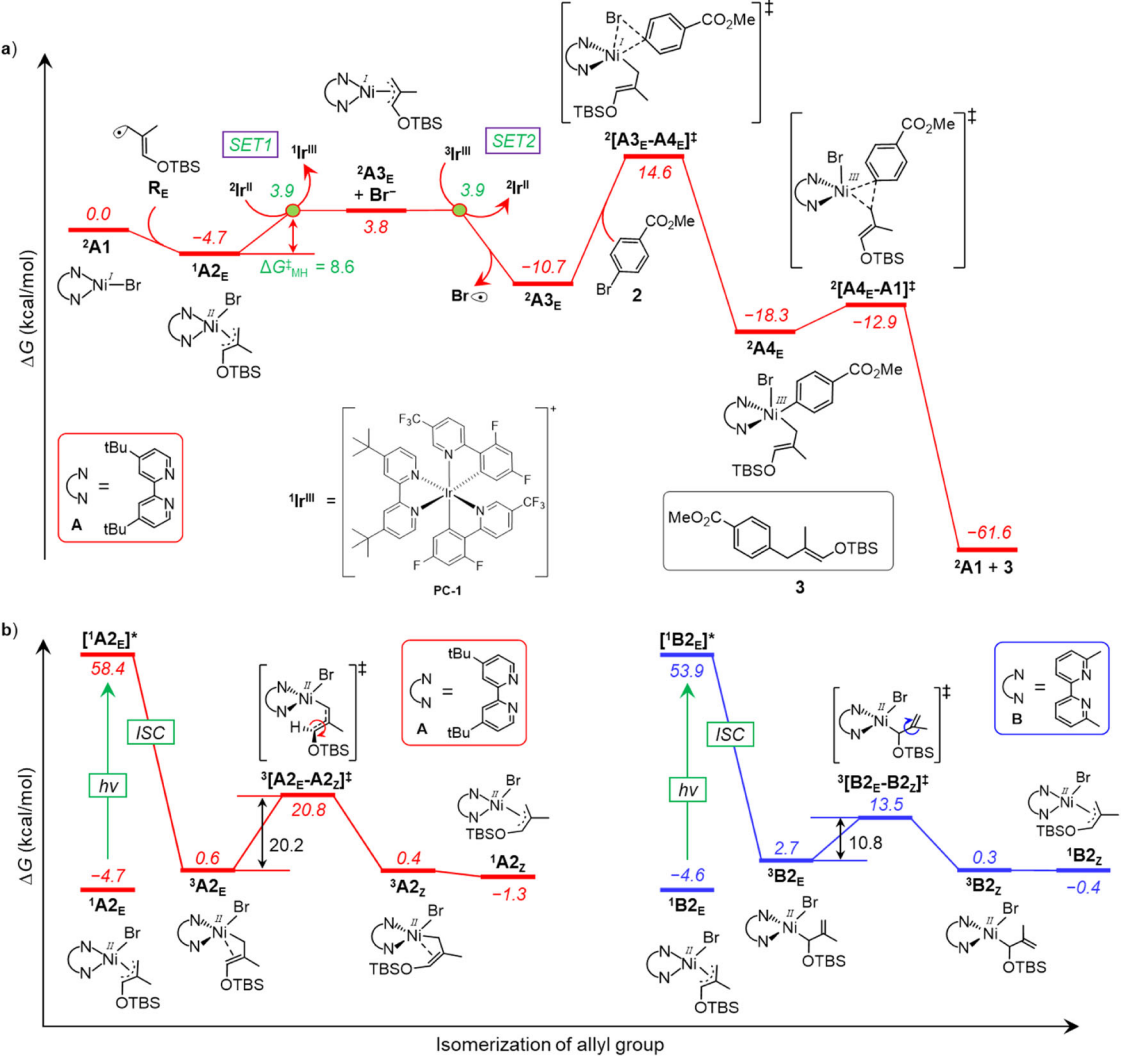
DFT calculated reaction energy profile. **a** Free-energy profile of Ni-catalytic cycle in the presence of dtbbpy ligand for *E*-product (**3**) formation. The M06(SMD-THF)/def2-TZVPP//PBE/def2-SVP/def2-TZVP level of theory was used to calculate the reported values. **b** Free-energy profiles for isomerization of allyl group at LNi(II)(allyl)Br complex for dtbbpy ligand (left) and 6,6’-dmbpy ligand (right). For energy conventions, refer to Fig. 7a.

We have investigated the Ni-catalytic cycle starting from LNi^I^-Br as the active catalyst^46,47^. The free-energy profile of the complete nickel catalytic pathway for the *E*-product (**3**) formation in the presence of dtbbpy ligand is shown in Fig. 7a, while the *Z*-product (**4**) pathway is shown in the Supplementary Fig. 17. In addition, both *E*- and *Z*-product formation pathways with 6,6’-dmbpy ligand are depicted in the Supplementary Figs. 18 and 19. The Ni-catalytic cycle starts with the radical addition **R**_**E**_ to the nickel atom of ^**2**^**A1** (Fig. 7a). The resulting ^**1**^**A2**_**E**_ undergoes endergonic 1e^−^ reduction by ^**2**^**Ir**^**II**^ to generate ^**2**^**A3**_**E**_ and Br^−^ (SET1, Fig. 7a). Further 1e^−^ oxidation of Br^−^ coupled with ^**3**^**Ir**^**III**^/^**2**^**Ir**^**II**^ reduction (SET2) leads to Br•, which is involved in the HAT step to generate allyl radical from **1**. The reaction continues with the oxidative addition of **2** to the ^**2**^**A3**_**E**_ via transition state ^**2**^**[A3**_**E**_**-A4**_**E**_**]**^**‡**^ and the energy barrier of 25.3 kcal/mol, leading to ^**2**^**A4**_**E**_. The Ni-cycle is completed by facile reductive elimination of the product from ^**2**^**A4**_**E**_ via transition state ^**2**^**[A4**_**E**_**-A1]**^**‡**^ and an activation barrier of 5.4 kcal/mol, regenerating active catalyst ^**2**^**A1** with the liberation of the *E*-product **3**. Consistently with the experiments (Fig. 6c), calculations suggest that *E*–*Z* isomerization of the allyl moiety is possible at the LNi(II)(allyl)Br complex, ^**1**^**A2**_**E**_ in Fig. 7a. Therefore, the *E*-/*Z*- product selectivity is occurring at this intermediate, depending on the relative feasibility between isomerization step and the corresponding SET.

The allyl isomerization is expected to occur via a classic η^3^ → η^1^ → η^3^ mechanism involving C−C bond rotation after the formation of the η^1^ Ni–C–C=C species and dissociation of the localized C=C bond from Ni. However, in the singlet state this isomerization is highly disfavored for ^**1**^**A2**_**E**_ and ^**1**^**B2**_**E**_, with free-energy barriers of 48.4 and 50.8 kcal/mol, respectively (Supplementary Figs. 21, 22). Importantly, under irradiation of light, promoting excitation of the complexes from singlet to a triplet state (^**1**^**A2**_**E**_/^**1**^**B2**_**E**_ → ^**3**^**A2**_**E**_/^**3**^**B2**_**E**_), the coordination mode of the allyl group spontaneously changes from ɳ^3^ to promote one electron from a filled d-orbital of Ni to the empty Ni d-orbital involved in accepting the 4e of the ɳ^3^ coordinated allyl. The ɳ^1^-isomers of ^**3**^**A2**_**E**_ and ^**3**^**B2**_**E**_ undergo C−C bond rotation to generate the respective *Z*-isomer ^**3**^**A2**_**Z**_ and ^**3**^**B2**_**Z**_ at the triplet state (Fig. 7b). In the case of the dtbbpy ligand, the most stable ɳ^1^ isomer has the unsubstituted carbon of the allyl group σ-bonded to the Ni atom, which requires *Z*-isomerization occurring through rotation around the partial C−C(OTBS) double bond, with an energy barrier (Δ*G*^‡^ = 20.2 kcal/mol) higher than that of the 1e^−^ reduction step, (Δ*G*_MH_^‡^ = 8.6 kcal/mol, SET1 in Fig. 7a). This indicates that formation of the *Z*-product is unfeasible. Differently, in the case of the 6,6′-dmbpy ligand, the most stable ɳ^1^ isomer has a well-defined Ni–C(OTBS) σ-bond. Consequently, *E*/*Z* isomerization occurs via rotation around a C–C single bond with a low free-energy barrier (Δ*G*^‡^ = 10.8 kcal/mol), and the corresponding *Z*-isomer (^**3**^**B2**_**Z**_) is favored energetically. This suggests a higher population of the *Z*-isomer over the *E* one prior to the 1e^−^ reduction step (Δ*G*_MH_^‡^ = 20.5 kcal/mol, SET4 in Supplementary Fig. 18). Alternative mechanisms were explored for the *E*/*Z* isomerization with dtbbpy ligand. However, those are unfavored due to very high energy pathways (see Supplementary Fig. 19).

**Fig. 8:**
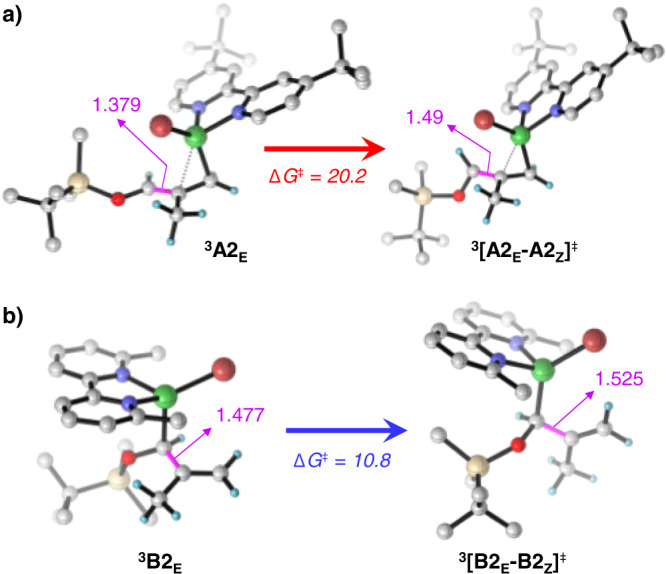
Transition states of isomerization step. For **a**) ligand A (dtbbpy) and **b**) ligand B (6,6'-dmpy), Rotation occurs around the C−C bond highlighted in pink.

Comparison of the ^**3**^**A2**_**E**_ and ^**3**^**B2**_**E**_ geometries explains the different impact of the two ligands on the isomerization step (Fig. 7b). The reduced steric hindrance in ^**3**^**A2**_**E**_ allows partial π coordination to nickel of the formally ɳ^1^ allyl group, with a relatively short C−C(OTBS) bond (1.379 Å), allowing rotation with the high energy barrier of Fig. 7b. Differently, due to steric substitutes at 6,6’ position in ^**3**^**B2**_**E**_, only one C atom of the ɳ^1^ allyl is interacting with nickel, allowing for low energy rotation (see Fig. 7b) around a C−C bond having a strong single bond character (1.477 Å, Fig. 8).

**Fig. 9:**
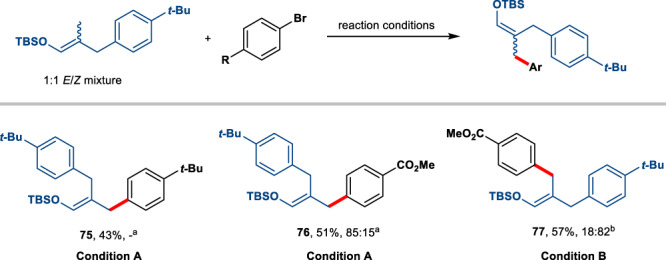
Electronic effect in the sequential diarylation coupling. **a** Condition **A**: the reactions were performed on a 0.2 mmol scale under the following reaction conditions: alkene (3 equiv.), aryl bromide (1 equiv.), 2,6-lutidine (1.5 equiv.), **PC-1** (1 mol%), NiBr_2_ (10 mol%), dtbbpy (15 mol%), benzene (2 mL), rt, 60 h. **b** Condition **B**: the reaction was performed on a 0.2 mmol scale under the following reaction conditions: alkene (3 equiv.), aryl bromide (1 equiv.), 2,4,6-collidine (3 equiv.), **PC-1** (1 mol%), NiBr_2_ (20 mol%), 6,6’-dmbpy (20 mol%), dioxane (2 mL), rt, 60 h. Yields and *E*/*Z* after purification by chromatography.

Although the cross-coupling of substrates bearing geminal dimethyl moiety has occurred with high levels of regio- and stereocontrol, we observed a small amount of product (2%) from diarylation. With this in mind, we next examined the feasibility of the direct use of a monoarylated product as the coupling partner. To our delight, the sequential diarylation is successfully applied with both electron-rich and electron-deficient aryl bromides to prepare diarylated silyl enol ethers (Fig. 9). Interestingly, our standard reaction conditions can achieve stereocontrol by discriminating substituent electronics. For instance, if the aryl ring of the substrate has an electron-donating substituent, introducing another one bearing an electron-withdrawing para substituent could give **76** in 51% yield and 85% *E* selectivity using **L1**. By switching to conditions with **L2**, the inverse configuration of the double bond can be obtained (**77**, 57% yield and 82% *Z* selectivity). This shows the potential for stereoselective alkene synthesis by exploiting the electronic property of the substrates.

In conclusion, we have developed a general method that permits the regio- and stereoselective synthesis of alkenes. This protocol enables catalytic allylic radical generation directly from simple and readily available alkenes and accommodates a wide variety of aryl halide coupling partners. Different from previous mechanistic proposals on C(sp^3^)-H functionalization with photoredox nickel dual catalysis, our experimental evidence, and detailed computational mechanistic studies support a Ni(I)-allylNi(II)-allylNi(I)-Ni(III) catalytic cycle. Computational studies further explain the stereochemical outcome and indicate that excitation of a Ni(II)-allyl complex from singlet to a triplet state results in a spontaneous change of the allyl group coordination and that the subsequent ligand-dependent isomerization can be directed by choice of the ligand to achieve *E*/*Z* selectivity. These unexpected findings led us systematically investigate the stereoselectivity and to develop stereodivergent allylic C(sp^3^)-H bond arylations in which a ligand switch allows the target synthesis of *E* and *Z* isomers of silyl enol ethers.

## Methods

### General procedure E for the cross-coupling reaction towards *E* selectivity (condition A)

To a 15 mL vial equipped with a stir bar was added NiBr_2_ (4.4 mg, 20 µmol, 0.1 equiv.) and 4,4’-di-*tert*-butyl-2,2’-bipyridine (8.1 mg, 30 µmol, 0.15 equiv), Ir[dF(CF_3_)ppy]_2_(dtbbpy)PF_6_ (2.3 mg, 0.002 mmol, 1 mol%), aryl halide (0.2 mmol, 1 equiv.), 2,6-lutidine (32.1 mg (∼35 µL), 0.3 mmol, 1.5 equiv.) and alkene (0.6 mmol, 3 equiv.). A mixture of benzene (1 mL) and THF (1 mL) was added, then the vial was degassed and under stirring irradiated with the corresponding blue LEDs photoreactor.

### General procedure F for the cross-coupling reaction towards *Z* selectivity (condition B)

To a 15 mL vial equipped with a stir bar was added NiBr_2_ (8.8 mg, 40 µmol, 0.2 equiv.) and 6,6′-dimethyl-2,2′-bipyridine (7.4 mg, 0.04 mmol, 20 mol%), Ir[dF(CF_3_)ppy]_2_(dtbbpy)PF_6_ (2.3 mg, 0.002 mmol, 1 mol%), aryl halide (0.2 mmol, 1 equiv.), 2,4,6-collidine (72.7 mg (∼80 µL), 0.6 mmol, 3.0 equiv.) and alkene (0.6 mmol, 3 equiv.). A mixture of dioxane (1 mL) and THF (1 mL) was added, then the vial was degassed and under stirring irradiated with the corresponding blue LEDs photoreactor.

## Supplementary information

Supplementary Information

Description of Additional Supplementary Files

Supplementary Data 1

## Data Availability

The authors declare that all data generated in this study are available within the article and the Supplementary Information.
