# Supplementary material for: Modulating stereoselectivity in allylic C(sp3)-H bond arylations via nickel and photoredox catalysis
Source: Nat Commun. 2023 Feb 1;14:548. doi: 10.1038/s41467-023-36103-0 (PMC9892578; doi:10.1038/s41467-023-36103-0)
Supplement: Supplementary file 1 — Supplementary Information [file 41467_2023_36103_MOESM1_ESM.docx]

***Supplementary Information for***

**Modulating Stereoselectivity in Allylic C(sp^3^)-H Bond Arylations via Nickel and Photoredox Catalysis**

Long Huang^1^*, Marcin Szewczyk^1^, Rajesh Kancherla^2^, Bholanath Maity^2^, Chen Zhu^2^, Luigi Cavallo^2^* and Magnus Rueping^1,3^*

^1^Institute of Organic Chemistry, RWTH Aachen University, 52074 Aachen, (Germany)

^2^KAUST Catalysis Center (KCC), King Abdullah University of Science and Technology (KAUST), Thuwal, 23955-6900 (Saudi Arabia)

^3^Institute for Experimental Molecular Imaging, RWTH Aachen University, 52074 Aachen, Germany

**Table of Contents**

1. **Supplementary Methods**

General Methods

1. **Supplementary Discussion**

Preparation and Characterization of alkenes

Optimization of the Reaction Conditions

Cyclic Voltammetry Data

Mechanistic Studies and Simmon-Smith Cyclopropanation

General Procedure for the Cross Coupling Reaction

Confirmation of *E*/*Z* isomer for the cross coupling with silyl enol ether **1**

Computational Details

1. **Supplementary Data**

Characterization of the Products

NMR Spectra

1. **Supplementary Reference**
2. **Supplementary Methods**

**General Methods.** Unless otherwise noted, all commercially available compounds were used as provided without further purification. Solvents for chromatography were technical grade and distilled prior to use. Dry toluene, 1, 2-dichloroethane (DCE), chloroform, chlorobenzene used in reactions were obtained by distilling over calcium hydride and were stored over activated molecular sieves (4 Å). Dry isopropyl ether (*^i^*Pr_2_O), diethyl ether (Et_2_O), tetrahydrofuran (THF) and toluene used in reactions were obtained by distilling over sodium-benzophenone ketyl. Analytical thin-layer chromatography (TLC) was performed on Macherey-Nagel silica gel 60 aluminium plates with F-254 indicator, visualised by UV irradiation. Column chromatography was performed using MN silica gel (particle size 0.040-0.063 mm). ^1^H-NMR and ^13^C-NMR spectra were recorded on a vnmrs-400 or vnmrs-600 spectrometer in CDCl_3_ with residual proton signal of the deuterated solvents as the internal reference (δH = 7.26 ppm and δC = 77.16 ppm for CDCl_3_). Data are reported in the following order: chemical shift (δ) in ppm; multiplicities of ^1^H NMR are indicated s (singlet), d (doublet), t (triplet), q (quartet), m (multiplet), dd (doublet of doublet), tt (triplet of triplet), dt (doublet of triplet), td (triplet of doublet); coupling constants (*J*) are in Hertz (Hz). All ^13^C NMR spectra were measured with ^1^H decoupling. Carbon assignment was determined by APT spectra: s stands for quarternary carbon, d stands for CH-group, t stands for CH_2_-group, q stands for CH_3_-group. IR spectra were recorded on a Jasco FT/IR-420 spectrometer and are reported in terms of frequency of absorption (cm^-1^). Mass spectra were acquired on a Finnigan SSQ7000 (EI/CI) spectrometer and high resolution mass spectra on a Finnigan MAT 95 (EI/CI) or on a ThermoFisher Scientific LTQOrbitrap XL (ESI). The Blue LED strips 24 V (19.2 W/m, with emission maximum at λ_max_ = 467 nm) were purchased from Ledxon GmbH (Germany).

1. **Supplementary Discussion**

## General Procedure for the Synthesis of Alkenes

Supplementary Figure 1. Alkenes studied in the current report. Compound 1^1^, 2^2^, 4^3^, 5^3^, 6,^4^ 8^5^, 9^6^, 10^6^, 18^1^, 20^7^, 21^8^, 22^1^ are known.

**General procedure A with *tert*-Butyldimethylchlorosilane and Aldehyde:**

The *tert*-butyldimethylchlorosilane (1 equiv.) at 0 °C was added to a solution of the corresponding aldehyde (1.5 equiv.) and DBU (1.1 equiv.) in dichloromethane. The mixture was heated in reflux for 2 h. The solution was then cooled and most of solvent was removed under reduced pressure. The residue was poured into ice water and extracted with pentane. After drying over anhydrous Na_2_SO_4_, it was filtered and concentrated in vacuo. The crude product was purified by either neutral alumina flash column chromatography or vacuum distillation to obtain the pure silyl enol ether. The ^1^H NMR spectrum were consistent with literature data.

**General procedure B with TIPS or TBS triflate and carbonyl compounds:**

A flame-dried round-bottom flask equipped with a stir bar under argon was charged with carbonyl compound (1.0 equiv.) and Et_3_N (3.0 equiv.) in DCM. Then the reaction mixture was cooled at 0 °C with an ice bath and the TIPSOTf or TBSOTf (1.0 equiv.) was added dropwise. The reaction mixture was stirred overnight at room temperature, followed by quenching with sat. aqueous NaHCO_3_ and extracted with ether three times. After drying over anhydrous Na_2_SO_4_ it was filtered and concentrated in vacuo. The crude product was purified by neutral alumina flash column chromatography to afford the corresponding product.

**General procedure C with TBSCl and carbonyl compounds:**

The ketone (1.0 equiv.) was added dropwise at -78 ^o^C under argon to a solution of LDA (1.1 equiv.) in dry THF. The resulting mixture was stirred at the same temperature for 30 mins. After this time, a solution of TBSCl (1.1 equiv.) in THF was added dropwise. The reaction mixture was stirred for an addition 30 mins then allowed to warm to room temperature and stirred overnight. The reaction was stopped by quenching with saturated ammonium chloride solution and extracted with ether three times. After drying over anhydrous Na_2_SO_4_ it was filtered and concentrated in vacuo. The crude product was purified by neutral alumina flash column chromatography to afford the corresponding product.

**General procedure D with allylation of O/N nucleophile and isomerization reaction:**

Step 1: To a solution of the nucleophile (1 equiv) in THF was added sodium hydride (1.2 equiv.) under an atmosphere of nitrogen at 0 ^o^C. The mixture was allowed to stir for 30 minutes at the same temprature. After adding allyl halide (1.3 equiv), the mixture was then allowed to stir at room temperature for 30 minutes and reflux overnight. The reaction was quenched by the addition of saturated ammonium chloride solution and extracted with ether three times. After drying over Na_2_SO_4_ and concentrated in vacuo. The crude residue was purified by column chromatography.

Step 2: *n*-Butyllithium (1.6 M solution in hexane, 2 equiv.) was added slowly to a stirred solution of diisopropylamine (2.0 equiv.) in THF at ‒78 °C. The mixture was stirred for 10 min and allowed to warm to 0 ^o^C for 10 min. After cooling to ‒78 °C again, the corresponding allyl substrate from Step 1 (1 equiv.) was added dropwise *via* syringe. The mixture was stirred overnight till full conversion, followed by quenching with saturated ammonium chloride solution and extracting with ether. After drying over anhydrous Na_2_SO_4_ it was filtered and concentrated in vacuo. The crude product was purified by neutral alumina flash column chromatography to obtain the pure alkene.

**Compound 3:** Prepared following the general procedure D.

**^1^H NMR** (400 MHz, CDCl_3_) δ 5.81 – 5.73 (m, 1H), 4.24 – 4.10 (m, 1H), 1.77 – 1.65 (m, 6H), 1.58 (s, 3H), 1.57 – 1.53 (m, 5H).

**^13^C NMR** (101 MHz, CDCl_3_) δ 138.9, 111.2, 82.7, 32.8, 23.7, 19.8, 15.2.

**IR (ATR)** ν 2958, 2920, 1688, 1444, 1378, 1338, 1157, 984, 825 cm^-1^;

**HRMS (EI)** *m/z* Calcd for C_9_H_16_O^+^ [M]^+^: 140.11957, found: 140.11928.

**Compound 5:** Prepared following the general procedure D.

**^1^H NMR** (600 MHz, CDCl_3_) 5.80 (s, 1H), 3.90 – 3.79 (m, 1H), 2.15 (ddd, *J* = 14.0, 9.5, 4.4 Hz, 1H), 2.05 (ddd, *J* = 13.3, 9.8, 4.5 Hz, 1H), 1.77 – 1.67 (m, 1H), 1.66 – 1.59 (m, 4H), 1.53 (s, 3H), 1.30 – 1.19 (m, 2H), 1.07 (dd, *J* = 13.4, 3.3 Hz, 1H), 0.88 (s, 3H), 0.85 (s, 6H).

**^13^C NMR** (151 MHz, CDCl_3_) δ 140.4, 109.9, 86.3, 49.5, 48.1, 45.1, 36.5, 28.3, 26.9, 20.0, 19.7, 19.0, 15.4, 14.0.

**IR (ATR)** ν 2950, 2879, 1689, 1453, 1383, 1162, 1053, 1021, 989, 826 cm^-1^;

**HRMS (ESI)** *m/z* Calcd for C_14_H_24_ONa^+^ [M+Na]^+^: 231.17194, found: 231.17179.

**Compound 7:** Prepared following the general procedure D.

**^1^H NMR** (600 MHz, CDCl_3_) δ 5.77 (p, *J* = 1.5 Hz, 1H), 3.20 (td, *J* = 10.7, 4.3 Hz, 1H), 2.10 (pd, *J* = 7.0, 2.8 Hz, 1H), 1.96 – 1.88 (m, 1H), 1.62 – 1.49 (m, 5H), 1.47 (d, *J* = 1.3 Hz, 3H), 1.33 – 1.23 (m, 2H), 0.97 – 0.85 (m, 2H), 0.83 (t, *J* = 6.8 Hz, 6H), 0.80 – 0.76 (m, 1H), 0.71 (d, *J* = 6.9 Hz, 3H).

**^13^C NMR** (151 MHz, CDCl_3_) δ 139.3, 109.9, 80.8, 48.0, 41.7, 34.7, 31.8, 26.0, 23.7, 22.4, 21.0, 19.8, 16.5, 15.3.

**IR (ATR)** ν 2869, 1689, 1375, 1331, 1170, 1044, 995, 830 cm^-1^;

**HRMS (ESI)** *m/z* Calcd for C_14_H_26_ONa^+^ [M+Na]^+^: 233.18759, found: 233.18763.

**Compound 11:** Prepared following the general procedure C.

**^1^H NMR** (**400 MHz, CDCl_3_,** **mixture of isomers *E*/*Z*, 69:31**)**:** ***E* isomer** (major)δ 7.24 – 7.07 (m, 4H), 5.08 (q, *J* = 7.0 Hz, 1H), 2.32 (s, 3H), 1.43 (d, *J* = 7.0 Hz, 3H), 0.87 (s, 9H), -0.03 (s, 6H); ***Z* isomer** (minor) 4.79 (q, *J* = 6.7 Hz, 1H), 2.35 (s, 3H), 1.72 (d, *J* = 6.9 Hz, 3H), 0.92 (s, 9H), -0.17 (s, 6H), the remaining resonances are insufficiently resolved from those of the ***E* isomer** to be reported.

**^13^C NMR (101 MHz, CDCl_3_, mixture of isomers *E*/*Z*, 69:31):** ***E* isomer** (major) δ 150.5, 137.3, 136.9, 130.2, 129.8, 127.8, 125.2, 104.9, 25.8, 19.7, 18.2, 12.7, -4.5; ***Z* isomer** (minor) 150.7, 140.2, 136.4, 129.2, 127.7, 125.4, 107.3, 25.9, 20.3, 18.4, 11.3, -4.5. the remaining resonances are insufficiently resolved from those of the ***E* isomer** to be reported.

**IR (ATR)** ν 2930, 2325, 2112, 1804, 1660, 1463, 1341, 1312, 1252, 1219, 1112, 1058, 950, 838, 778 cm^-1^;

**HRMS (ESI)** *m/z* Calcd for C_16_H_27_OSi^+^ [M+H]^+^: 263.18257, found: 263.18261.

**Compound 11:** Prepared following the general procedure C.

**^1^H NMR** (400 MHz, CDCl_3_) δ 1.71 (s, 3H), 1.60 (s, 3H), 1.19 (s, 9H), 0.98 (s, 9H), 0.14 (s, 6H).

**^13^C NMR** (101 MHz, CDCl_3_) δ 151.1, 109.3, 36.6, 30.3, 26.6, 22.0, 20.4, 19.1, -2.3.

**IR (ATR)** ν 2931, 1647, 1467, 1391, 1362, 1255, 1144, 1076, 931, 837, 774 cm^-1^;

**HRMS (ESI)** *m/z* Calcd for C_14_H_31_OSi^+^ [M+H]^+^: 243.21387, found: 243.21383.

**Compound 11:** Prepared following the general procedure C.

**^1^H NMR** (400 MHz, CDCl_3_) 4.58 (q, *J* = 6.8 Hz, 1H), 1.53 (d, *J* = 6.9 Hz, 3H), 1.06 (s, 9H), 1.00 (s, 9H), 0.19 (s, 6H).

**^13^C NMR** (101 MHz, CDCl_3_) δ 159.1, 97.0, 36.7, 29.0, 26.7, 19.4, 12.0, -2.7.

**IR (ATR)** ν 2957, 2862, 1663, 1469, 1389, 1319, 1255, 1147, 1075, 1003, 898, 828, 775 cm^-1^;

**HRMS (ESI)** *m/z* Calcd for C_13_H_29_OSi^+^ [M+H]^+^: 229.19822, found: 229.19827.

**Compound 11:** Prepared using 3-(4-*tert*-Butylphenyl)isobutyraldehyde (25 mmol), DBU (3 equiv.) and TBSCl (1.1 equiv.) in THF 30 mL. The mixture was stirred for 2 h. After that time, the reaction was quenched by addition of 50 mL of water and the product was extracted with ether three times. The combined organic phases were washed with brine, dired and concentrated. The crude product was purified by neutral alumina flash column chromatography to afford the titled compound as a colorless oil (4.65 g, 58% yield).

**^1^H NMR** (400 MHz, CDCl_3_, **mixture of isomers *E*/*Z*, 1.1:1**) δ 7.33 – 7.25 (m, 2H*_E_*_+_*_Z_*), 7.17 – 7.07 (m, 2H*_E_*_+_*_Z_*), 6.22 (d, *J* = 1.5 Hz, 0.52H*_E_*), 6.15 (d, *J* = 1.5 Hz, 0.48H*_Z_*), 3.38 (s, 0.96H*_Z_*), 3.15 (s, 1.04H*_E_*), 1.52 (d, *J* = 1.5 Hz, 1.56H*_E_*), 1.46 (d, *J* = 1.5 Hz, 1.44H*_E_*), 1.31 (s, 9H*_E_*_+_*_Z_*), 0.95 (s, 9H*_E_*_+_*_Z_*), 0.16 (s, 2.88H*_Z_*), 0.15 (s, 3.12 H*_E_*).

**^13^C NMR** (101 MHz, CDCl_3_, **mixture of isomers *E*/*Z*, 1.1:1**) δ 148.8, 148.4, 138.0, 137.7, 135.4, 134.2, 128.5, 128.4, 125.2, 116.9, 116.2, 39.8, 34.6, 34.5, 34.5, 31.6, 25.9, 18.4, 18.3, 17.1, 12.8, -5.0, -5.1.

IR (ATR) ν 2956, 2859, 2324, 2106, 1898, 1672, 1466, 1362, 1255, 1192, 1152, 914, 838, 779 cm^-1^;

HRMS (EI) for C_20_H_34_OSi^+^ (M)^+^ : 318.23735; Found : 318.23735.

**Optimization of the Reaction Conditions**

**Supplementary Table 1.** Screening of photocatalyst

| Entry | Photocatalyst | *E*/*Z* ratio | Yield [%] |
| --- | --- | --- | --- |
| 1 | **PC-I** | 88:12 | 75% |
| 2 | **PC-II** | 91:9 | 15% |
| 3 | **PC-III** | 94:6 | 18% |
| 4 | **PC-IV** | 86:14 | 65% |
| 5 | **PC-V** | 84:16 | 45% |
| 6 | **PC-VI** | - | 0 |

^a^Reactions were carried out at 0.1 mmol scale. ^b^Yield and *E*/*Z* ratio were determined by crude ^1^H NMR with reference to 1,3,5-trimethoxybenzene.

**Supplementary Table 2.** Screening of Nickel catalyst

**Supplementary Table 3.** Ligand effect

^a^Reactions were carried out at 0.1 mmol scale. ^b^Yield and *E*/*Z* ratio were determined by crude ^1^H NMR with reference to 1,3,5-trimethoxybenzene.

**Supplementary Table 4.** Screening of light source

**Supplementary Table 5.** Solvent effect

**Supplementary Table 6.** Control experiments

| Entry | Change from standard conditions | Yield (E/Z) |
| --- | --- | --- |
| 1 | No change | 79 (93:7) |
| 2 | Without PC | 0 (-) |
| 3 | Without NiBr_2_ | 0 (-) |
| 4 | Without dtbpy | trace (-) |
| 5 | Without base | 0 (-) |
| 6 | Without light | 0 (-) |

**Supplementary Table 7.** Selective optimizations for Z slectivity

| Entry | PC ( mol%) | Ni | 6,6-dmebpy | base | Solvent (x mL) | Yield (Z/E) |
| --- | --- | --- | --- | --- | --- | --- |
| 1 | 1 | NiCl_2_ DME  10 mol% | 15 mol% | Lutidine 1.5 equiv. | dioxane  1 mL | 27 (74:26) |
| 2 | 1 | NiBr_2_  20 mol% | 25 mol% | Lutidine 1.5 equiv. | dioxane  1 mL | 60 (83:17) |
| 3 | 1 | NiBr_2_ diglyme  20 mol% | 25 mol% | Lutidine 1.5 equiv. | dioxane  1 mL | 58 (83:17) |
| 4 | 1 | NiBr_2_  20 mol% | 25 mol% | Lutidine 1.5 equiv. | dioxane  2 mL | 54 (82:18) |
| 5 | 1 | NiBr_2_  20 mol% | 25 mol% | Lutidine 3 equiv. | dioxane  1 mL | 71 (84:16) |
| 6 | 1 | NiBr_2_  20 mol% | 20 mol% | collidine 3 equiv. | dioxane  1 mL | 76 (90:10) |
| 7 | 1 | NiBr_2_  20 mol% | 20 mol% | collidine 5 equiv. | dioxane  1 mL | 75 (88:12) |
| 8 | 1 | NiBr_2_  20 mol% | 20 mol% | collidine 3 equiv. | dioxane/THF  1:1 | 69 (90:10) |

^a^Reactions were carried out at 0.1 mmol scale. ^b^Yield and *E*/*Z* ratio were determined by crude ^1^H NMR with reference to 1,3,5-trimethoxybenzene.

**Formal beta-arylation of carbonyl compounds**

**Cyclic Voltammetry Data**

Cyclic voltammetry was performed using a PGSTAT101 from Metrohm Autolab with a platinum working electrode, a Ag^+^ (0.01 M AgNO_3_, 0.1 M NBu_4_PF_6_, MeCN)/Ag as reference electrode, a platinum wire counter electrode. All measurements were taken in N_2_-sparged MeCN with 0.1 M NBu_4_PF_6_ as supporting electrolyte where the tertiary alcohol concentration was 1 mM. The sweep rate was 10 mV/s and no reversible electrochemical event was observed in all cases. The values for *E*_p/2_ are referenced to SCE (Saturated Calomel Electrode) by adding 0.262 V to the measured potential.

Supplementary Figure ****2**.** Cyclic voltammogram of *tert*-butyldimethyl((2-methylprop-1-en-1-yl)oxy)silane in MeCN shows an irreversible oxidation event at 1.64 V vs. SCE.

Supplementary Figure ****3**.** Cyclic voltammogram of triisopropyl((2-methylprop-1-en-1-yl)oxy)silane in MeCN shows an irreversible oxidation event at 1.64 V vs. SCE

Supplementary Figure ****4**.** Cyclic voltammogram of 2-methylprop-1-en-1-yl acetate in MeCN shows an irreversible oxidation event at 2.18 V vs. SCE

Supplementary Figure ****5**.** Cyclic voltammogram of (1S,2R,4S)-1,7,7-trimethyl-2-((2-methylprop-1-en-1-yl)oxy)bicyclo[2.2.1]heptane in MeCN shows an irreversible oxidation event at 1.3 V vs. SCE

Supplementary Figure ****6**.** Cyclic voltammogram of 1-ethoxy-2-methylprop-1-ene in MeCN shows an irreversible oxidation event at 1.34 V vs. SCE

**Radical trapping experiment with TEMPO**

To a 15 mL vial equipped with a stir bar was added NiBr_2_ (4.4 mg, 20 µmol, 0.1 equiv.) and 4,4’-di-*tert*-butyl-2,2’-bipyridine (8.1 mg, 30 µmol, 0.15 equiv), Ir[dF(CF_3_)ppy]_2_(dtbpy)PF_6_ (2.3 mg, 0.002 mmol, 1 mol%), arylhalide (43 mg, 0.2 mmol, 1 equiv.), 2,6-lutidine (32.1 mg (⁓35 µL), 0.3 mmol, 1.5 equiv.), triisopropyl((2-methylprop-1-en-1-yl)oxy)silane (137 mg, 0.6 mmol, 3 equiv.) and (2,2,6,6-tetramethylpiperidin-1-yl)oxyl (TEMPO, 1 or 2 equiv.). Dioxane (2 mL) was added, then the vial was degassed with Freeze-Pump-Thaw methods for three cycles, it was next stirred and irradiated with the corresponding blue LEDs photoreactor for 48 h. Yields were determined by ^1^H NMR analysis of the crude mixture using an internal standard.


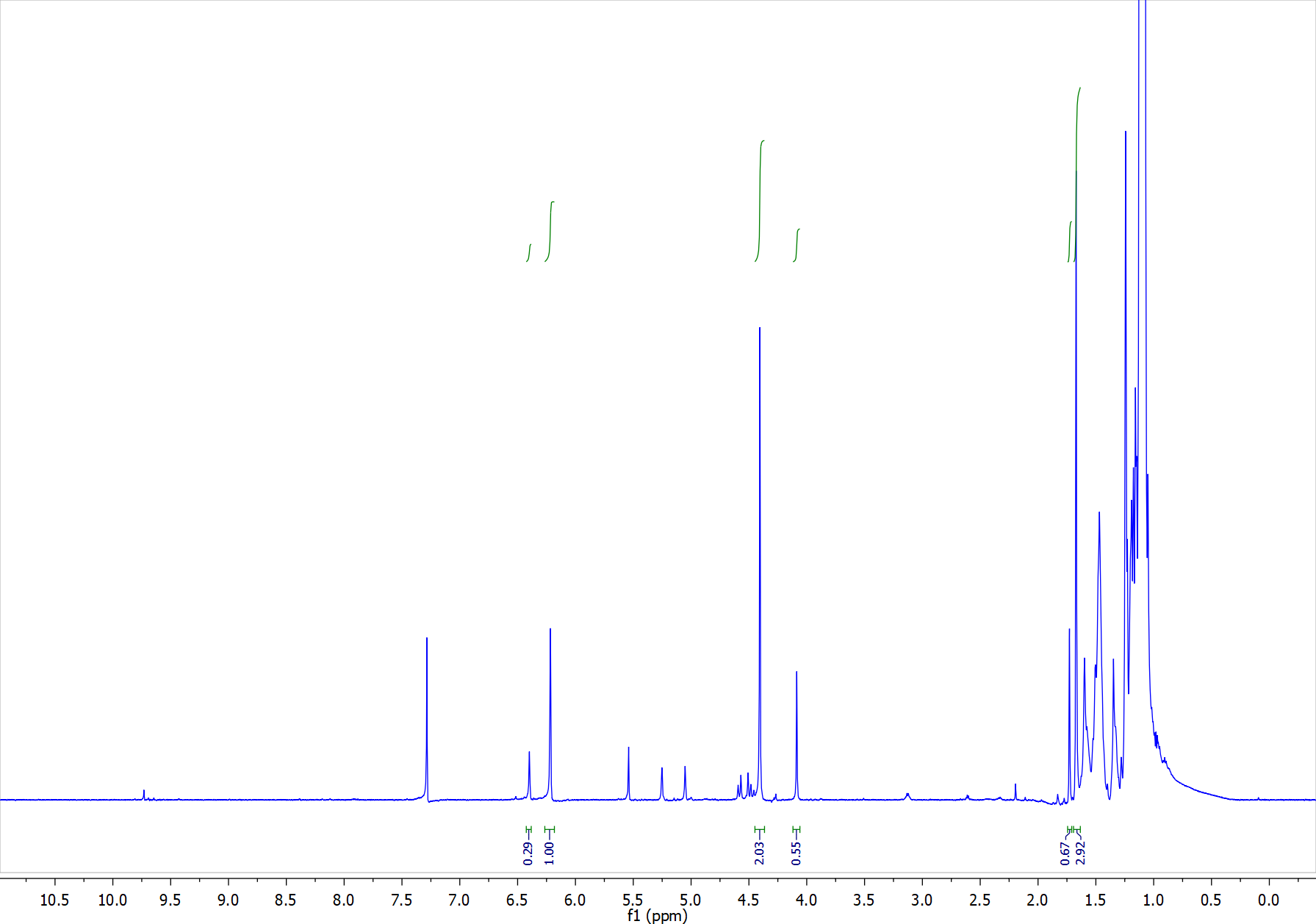


Supplementary Figure ****7**.** ^1^H NMR of the crude reaction mixture.


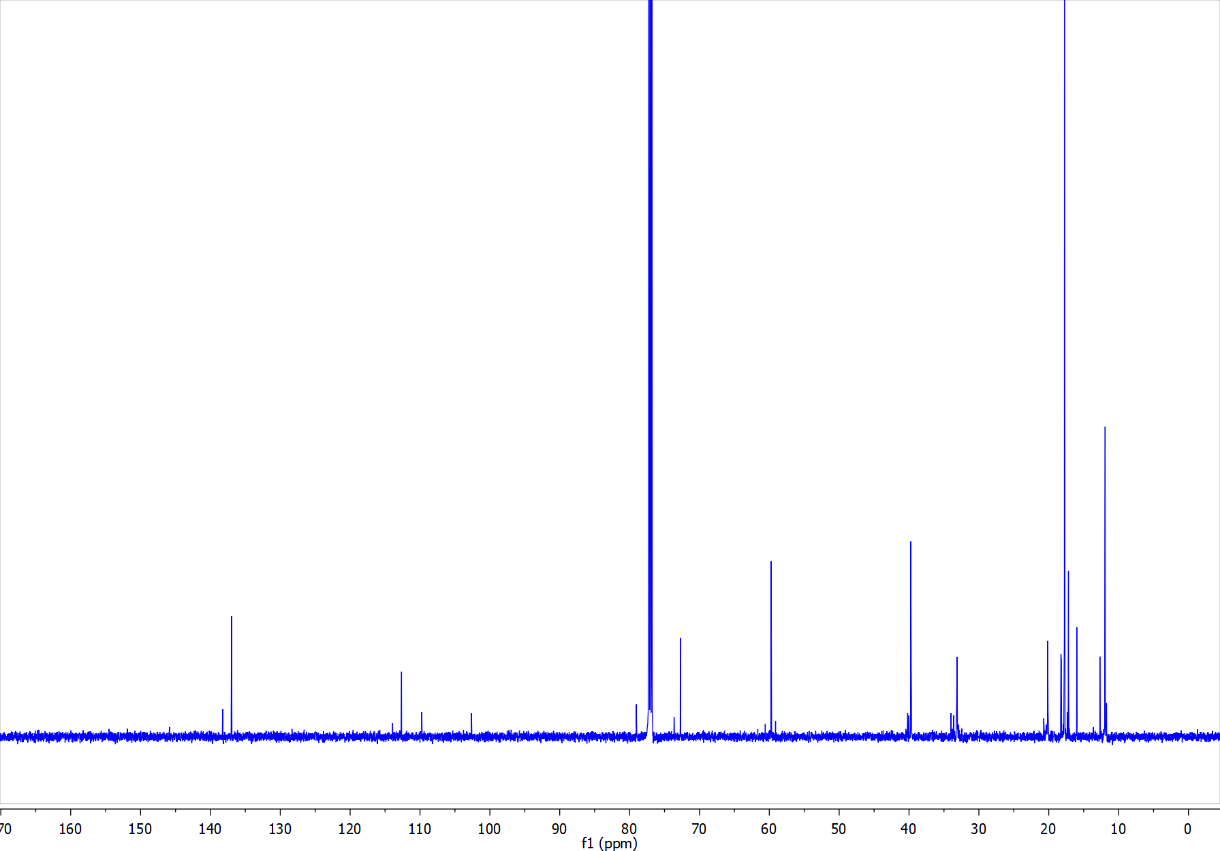
 Supplementary Figure ****8**.** ^13^C NMR of the crude reaction mixture.


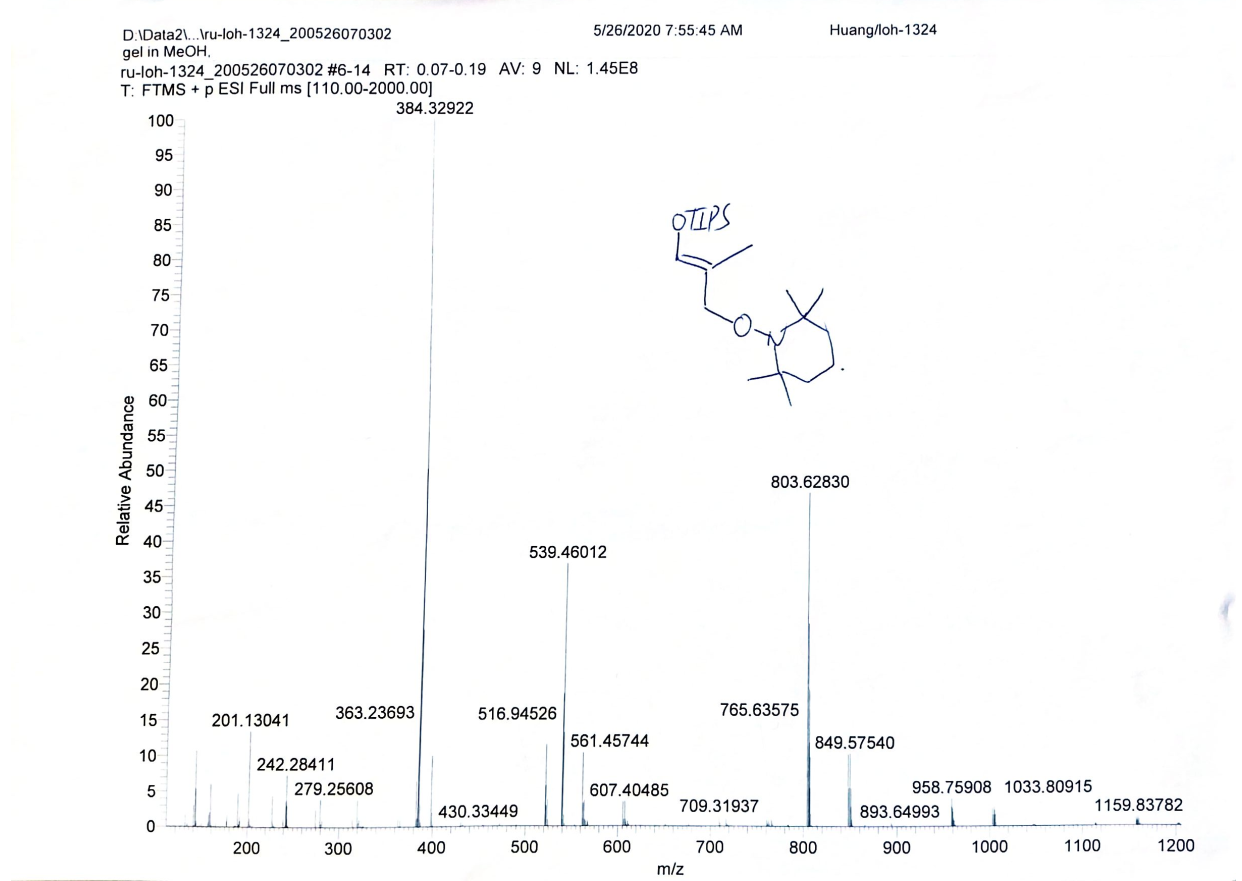


Supplementary Figure ****9**.** HRMS (ESI) analysis of the crude reaction mixture; HRMS (ESI) for C_22_H_45_O_2_NSi^+^ (M+H)^+^ : 384.32923; Found : 384.32922.

**Steady-state Stern-Volmer quenching experiments:**

Emission spectra were collected on fluoromax-4 spectrophotometer with excitation and emission slit widths of 3.5 nm. Quenching experiments were carried out using a 1x10^-5^ M solution of Ir[dF(CF_3_)(ppy)]_2_(dtbbpy)PF_6_ (**PC**) in Benzene:THF (1:1) and variable concentrations of quencher **1** (0.1, 0.3, 0.5, 0.7, 0.9 mM) in Benzene:THF (1:1). The samples were prepared in 2 mL quartz cuvettes, equipped with PTFE stoppers, and sealed with parafilm inside argon filled glove-box, removed from the glovebox and an emission spectrum was collected. Samples were excited at 390 nm and the intensity of emission was monitored at 474 nm expressed as the ratio I_0_/I, where I_0_ is the emission intensity of **PC** at 474 nm in the absence of a quencher and I is the observed intensity, as a function of the quencher concentration was measured (Figure S6 and S7).

Supplementary Figure ****10**.** Emission spectra of **PC-1** (1x10^-5^ M) at different concentrations **1**.

Supplementary Figure ****11**.** Steady-state Stern-Volmer quenching of **PC-1** using **1**.

**General procedure for the stoichiometric experiment using in situ formed nickel allyl complex.**

1. Following a precedent procedure by Mackenzie^9^: in a nitrogen filled glove box, a Schlenk tube with a stir bar was charged with TBSCl (118 mg, 0.78 mmol, 1.3 equiv.), methacrolein (100 µL, 1.2 mmol, 2 equiv.), 1-methoxy-2-methyl-1-(trimethylsilyloxy)propene (proton scavenger, 120 µL ,0.6 mmol , 1 equiv.) and 1 mL MeCN. The mixture was stirred for 5 min, after which it was transferred into another Schlenk tube containing Ni(COD)_2_ (165mg, 0.6 mmol, 1 equiv.). The mixture was stirred for 1 h, and next concentrated under high vacuum overnight to afford the nickel allyl dimer as a red solid. The complex was used for the next step without further purification.
2. To an oven-dried 15 mL tube equipped with a stir bar was added 4,4’-di-*tert*-butyl-2,2’-bipyridine (26.9 mg, 0.1 mmol, 1 equiv), Ir[dF(CF_3_)ppy]_2_(dtbpy)PF_6_ (1.1 mg, 0.001 mmol, 1 mol%), methyl 4-bromobenzoate (21.5 mg, 0.1 mmol, 1 equiv.), 2,6-lutidine (16.1 mg (⁓18 µL), 0.15 mmol, 1.5 equiv.). The nickel allyl complex from step 1 was dissolved in benzene (2 mL) and added carefully one half of the amount to the above tube. In a seperate tube equipped with a stir bar was added 4,4’-di-*tert*-butyl-2,2’-bipyridine (80.5 mg, 0.3 mmol, 3 equiv), Ir[dF(CF_3_)ppy]_2_(dtbpy)PF_6_ (1.1 mg, 0.001 mmol, 1 mol%), methyl 4-bromobenzoate (21.5 mg, 0.1 mmol, 1 equiv.), 2,6-lutidine (16.1 mg (⁓18 µL), 0.15 mmol, 1.5 equiv.). The other half of the nickel compelx solution was added to this tube. Then both vials were sealed, stirred, and irradiated with the corresponding blue LEDs photoreactor for 48 h. Yields and *E*/*Z* ratio were determined by crude ^1^H NMR with reference to 1,3,5-trimethoxybenzene.

Following a similar procedure as above, but collidine (3 equiv.), 6,6’-dimethyl-2,2’-bipyridine (6,6’-dmbpy, 1 or 3 equiv.) and dioxane were used instead. Unlike the dtbpy ligand, it is noteworthy that higher yield was obtained with increasing amount of 6,6’-dmbpy.

Following a similar procedure as above, but 2 equiv. of TEMPO was used.

Following a similar procedure as above, but no PC and light irradiation were employed, 3 equiv. of Zn was used instead. These reuslts suggest the allyl-Ni isomerization without light irradiation can be excluded.

**Simmon-Smith cyclopropanation of silyl enol ether**

To a dried and Ar-filled 10 mL round bottom flask was added silyl enol ether (0.2 mmol, 1equiv.) in 0.2 mL DCM at 0 ^o^C, the diiodomethane (0.5 mmol, 2.5 equiv) was added slowly. Next the Et_2_Zn (0.6 mmol, 3 equiv., 1 M hexane solution) was added dropwise. The reaction mixture was allowed to warm up to room temperature slowly and reacted overnight. The reaction was then quenched with NH_4_Cl solution and purified by column chromatography.

**General procedure E for the cross coupling reaction towards *E* selectivity (condition A)**

To a 15 mL vial equipped with a stir bar was added NiBr_2_ (4.4 mg, 20 µmol, 0.1 equiv.) and 4,4’-di-*tert*-butyl-2,2’-bipyridine (8.1 mg, 30 µmol, 0.15 equiv), Ir[dF(CF_3_)ppy]_2_(dtbpy)PF_6_ (2.3 mg, 0.002 mmol, 1 mol%), arylhalide (0.2 mmol, 1 equiv.), 2,6-lutidine (32.1 mg (⁓35 µL), 0.3 mmol, 1.5 equiv.) and alkene (0.6 mmol, 3 equiv.). A mixture of benzene (1 mL) and THF (1 mL) was added, then the vial was degassed with Freeze-Pump-Thaw methods for three cycles, it was next stirred and irradiated with the corresponding blue LEDs photoreactor.

**General procedure F for the cross coupling reaction towards *Z* selectivity (condition B)**

To a 15 mL vial equipped with a stir bar was added NiBr_2_ (8.8 mg, 40 µmol, 0.2 equiv.) and 6,6′-dimethyl-2,2′-bipyridine (7.4 mg, 0.04 mmol, 20 mol%), Ir[dF(CF_3_)ppy]_2_(dtbpy)PF_6_ (2.3 mg, 0.002 mmol, 1 mol%), arylhalide (0.2 mmol, 1 equiv.), 2,4,6-collidine (72.7 mg (⁓80 µL), 0.6 mmol, 3.0 equiv.) and alkene (0.6 mmol, 3 equiv.). A mixture of dioxane (1 mL) and THF (1 mL) was added, then the vial was degassed with Freeze-Pump-Thaw methods for three cycles, it was next stirred and irradiated with the corresponding blue LEDs photoreactor.

**Confirmation of *E*/*Z* isomer for the cross coupling with silyl enol ether 1.**

In the early phase of the project, we confirmed the *E*/*Z* isomer through 2D NMR analysis (NOESY and HSQC) of product **5** (E/Z 82:18), obtained under unoptimized conditions (see below).

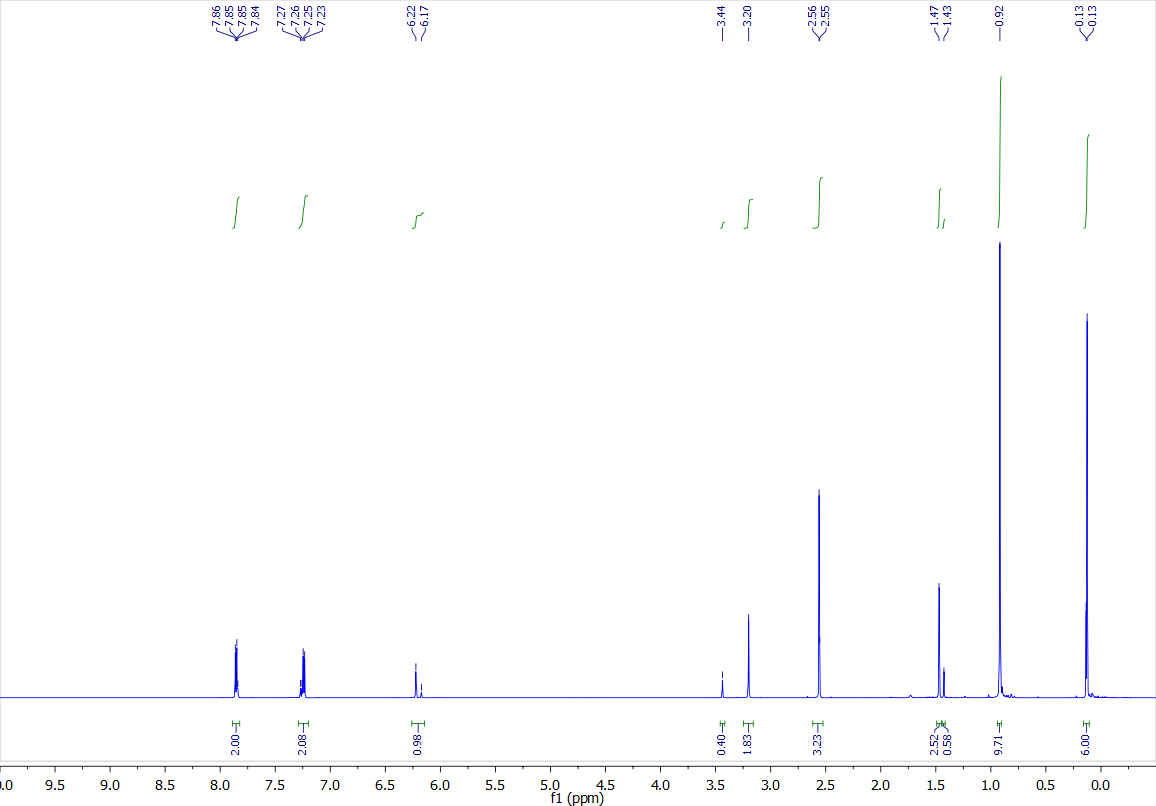


Supplementary Figure ****12**.** ^1^H NMR spectra of **5**.


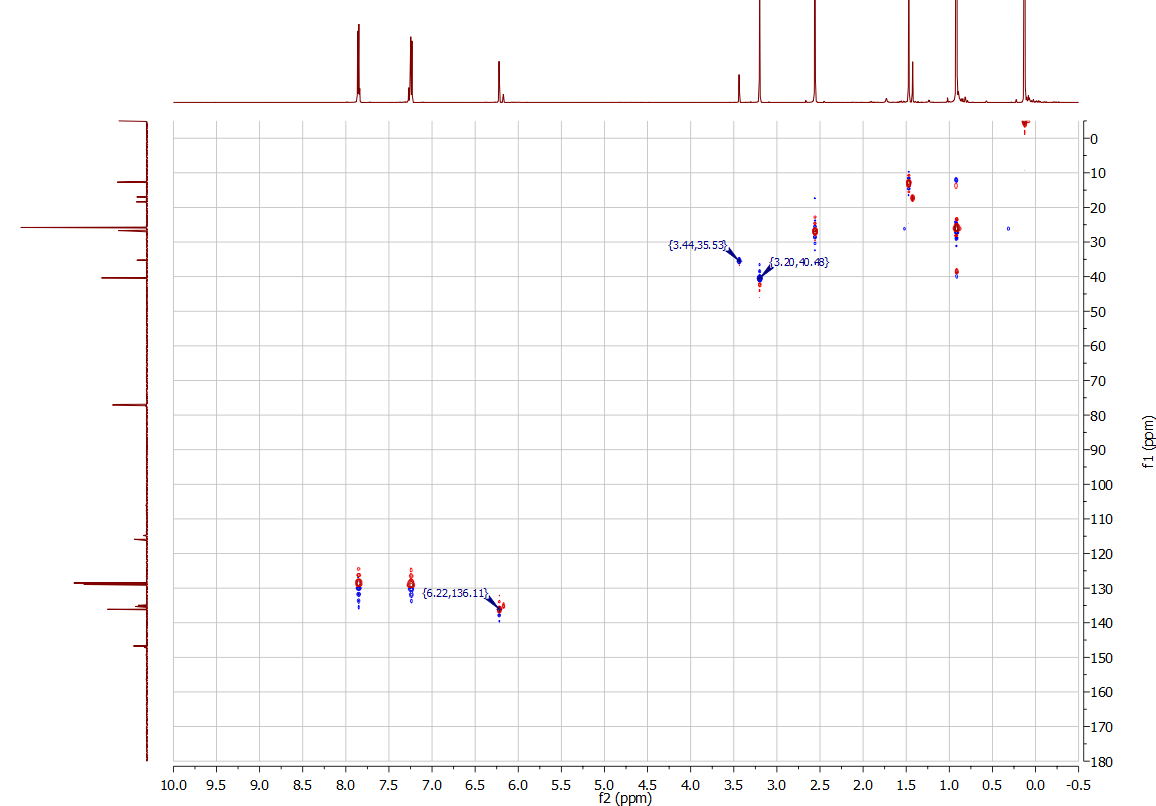


Supplementary Figure ****13**.** ^1^H-^13^C HSQC spectra of **5**.


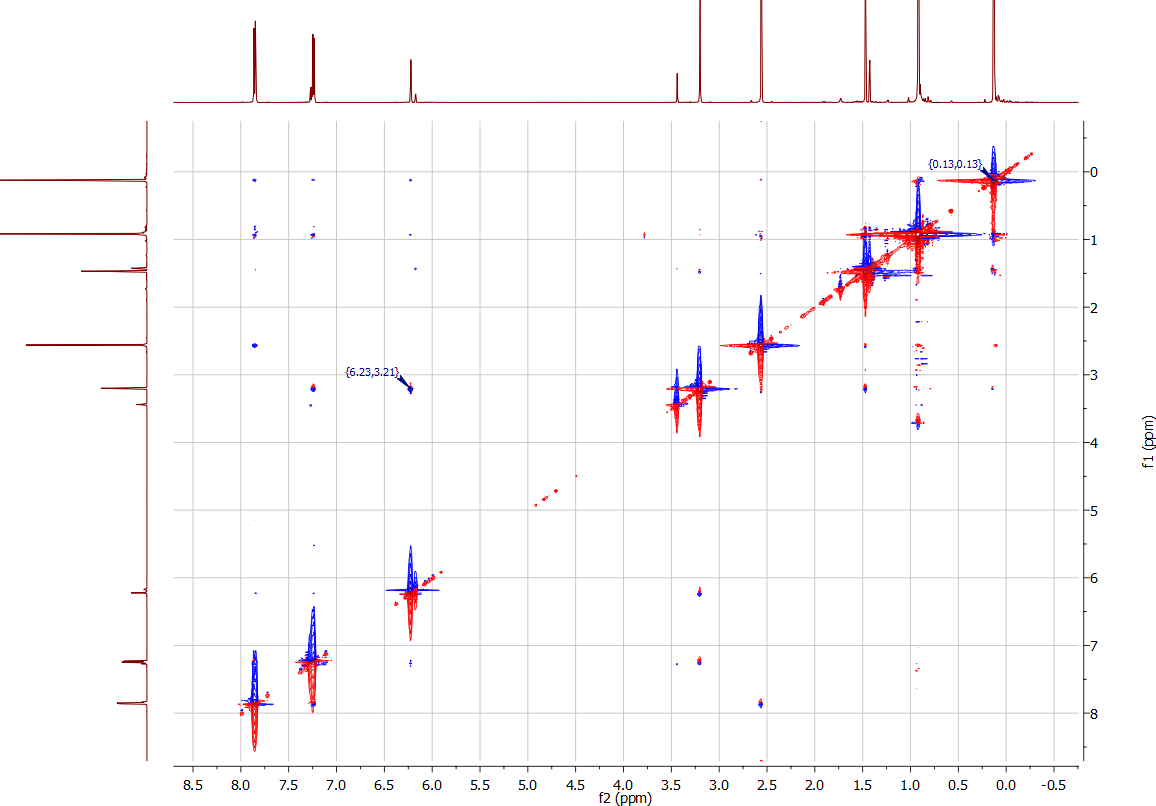


Supplementary Figure ****14**.** ^1^H-^1^H NOESY spectra of **5**.

**Computational Details**

All of the calculations were performed with the Gaussian 16 program package.^1^ Full geometry optimizations were performed at the generalized-gradient approximation (GGA) PBE functional.^2^ Numerical integrations were performed with an ultrafine grid. For geometry optimizations, the def2-TZVP^3^ pseudopotential and its associated double-ζ basis set was employed for Ni and Br and the def2-SVP^3^ basis set was used for rest of the atoms. The natures of all stationary points were determined by calculation of the analytical vibrational frequencies. These were also used to compute the molecular partition functions (298 K, 1 atm) with the conventional particle-in-a-box, rigid-rotator, quantum-mechanical harmonic oscillator approximation. Single point energy calculations were carried out at M06^4^ level of theory using def2-TZVPP^3^ basis sets for all atoms. The SMD (density based solvation model)^5^ was used to simulate the implicit solvent effect (solvent = THF; ε = 7.6). Unless specified otherwise, the Δ*G* was used throughout the text. The Δ*G* value was obtained by augmenting the Δ*E*_e_^S^ energy terms at M06(SMD-THF)/def2-TZVPP with the respective free energy corrections at the PBE/def2-SVP/def2-TZVP level. TD-DFT calculations were performed at the M06(SMD-THF)/def2-TZVPP level of theory. As it is known that translational and rotational entropies in solution for association and dissociation processes are overestimated and underestimated respectively, and that the deviation in the free energies is approximately 1.89 kcal/mol from the standard state (1 atm) to 1 M in solution.^6^ Therefore, we have reduced by 1.89 kcal/mol the free energy for addition steps and we have added 1.89 kcal/mol to the free energy of the dissociation steps.

**Concentration corrections**

The Gibbs free energy corrections of each component is modified by incorporating its concentration in solution in terms of partial pressure.^7^ In the Gaussian program, the concentration can be specified by adjusting the pressure value based on the ideal gas law p_i_ = (n_i_/V)RT, where p_i_ is the partial pressure, R the gas constant (0.082 L·atm·K^-1^·mol^-1^), T the absolute temperature, n_i_ the molar quantity, and V the reaction volume. The experimental concentrations of catalyst and reactants of the arylation and alkylation reaction are approximated by setting the partial pressures as follows:

| component | n_i_ (mol) | V (L) | R (L·atm·K^-1^·mol^-1^) | T (K) | p_i_ (atm) |
| --- | --- | --- | --- | --- | --- |
| **PC-1** | 0.2x10^-5^ | 2.0x10^-3^ | 0.082 | 298.15 | 0.024 |
| **LNiBr_2_** | 0.2x10^-4^ |  |  |  | 0.244 |
| **1** | 0.6x10^-3^ |  |  |  | 7.335 |
| **2** | 0.2x10^-3^ |  |  |  | 2.445 |
| **lutidine** | 0.3x10^-3^ |  |  |  | 3.667 |

**Energy barriers of single electron transfer steps, involved in this whole study, by using Marcus-Hush theory^8^**

Applying the Marcus-Hush theory of electron transfer, the free energy barrier of a singlet electron transfer process can be estimated according to the following equation:

$$\Delta G_{\mathrm{MH}}^{\ddagger}=\frac{{({\Delta G}_{r}+\lambda)}^{2}}{4\lambda}$$

∆*G*_r_ is the free energy change of the step, λ is the reorganization energy, which has two components, inner sphere and outer sphere. However the first one is considered to be neglected, and hence, the total λ will the outer sphere reorganization energy, which can be calculated by the equation:

$$\lambda=\lambda_{\mathrm{outer}}=332(\frac{1}{2a_{1}}+\frac{1}{2a_{2}}-\frac{1}{R})(\frac{1}{\varepsilon_{\mathrm{opt}}}-\frac{1}{\varepsilon})$$

a_1_ and a_2_ are the radii of donor and acceptor, R is the sum of a_1_ and a_2_, ε_opt_ and ε is the optical dielectric constant and static dielectric constant of solvent respectively (for THF ε_opt_ = 1.98 ε = 7.43) [ε_opt_ = (refractive index)^2^].

**Table S1.** Calculated free energy barriers (∆*G*^‡^_MH_) of single electron transfer steps and their relevant parameters.

|  | a_1_ (Å) | a_2_ (Å) | R (Å) | λ | ∆*G*_r_ | ∆*G*^‡^_MH_ |
| --- | --- | --- | --- | --- | --- | --- |
| ***SET1*** | 6.65 | 6.6 | 13.25 | 9.28 | 8.6 | 8.6 |
| ***SET2*** | 7.09 | 3.47 | 10.56 | 14.75 | -14.6 | 0.1 |
| ***SET3*** | 6.65 | 6.6 | 13.25 | 9.28 | 7.9 | 8.0 |
| ***SET4*** | 6.65 | 6.33 | 12.98 | 9.49 | 18.4 | 20.5 |
| ***SET5*** | 6.65 | 6.15 | 12.8 | 9.64 | 14.1 | 14.6 |
| ***SET6*** | 7.09 | 4.26 | 11.35 | 12.27 | 22.4 | 24.4 |
| ***SET7*** | 6.65 | 6.0 | 12.65 | 9.77 | 6.7 | 6.9 |
| ***SET8*** | 7.09 | 6.0 | 13.09 | 9.53 | 13.7 | 14.1 |
| ***SET9*** | 6.92 | 4.26 | 11.18 | 12.32 | 10.4 | 10.5 |
| ***SET10*** | 6.92 | 3.47 | 10.39 | 14.77 | −26.6 | 2.4 |
| ***SET11*** | 7.09 | 6.6 | 13.69 | 9.01 | 18.7 | 21.3 |

**Photoredox Catalytic Cycle**

Two different phtotoredox catalytic cycles have beene considered starting from TIrIII for both the active catalyst formation step and Ni-catalytic pathway.

**SETs involve in active Ni-catalyst formation (**Supplementary Figure ****15**):** Along the reductive cycle **^3^Ir^III^** is reduced to **^2^Ir^II^** by oxidation of lutidine (SET6), and so-formed **^2^Ir^II^** is then oxidized to **^1^Ir^III^** by the reduction of **^3^A-NiBr_2_** (SET7). Along the oxidative cycle **^3^Ir^III^** is oxidized to **^2^Ir^IV^** by the reduction of **^3^A-NiBr_2_** to **^2^A1** (SET8), and the so-formed **^2^Ir^IV^** is then reduced to **^1^Ir^III^** by the oxidation of either lutidine (SET9) or oxidation of Br^−^ (SET10). Calculated free energy change and extimated barrier of SETs indicate that the oxidative cycle is favored than the reductive cycle in the case of initial catalyst activation to form the active Ni-complex **^2^A1**.


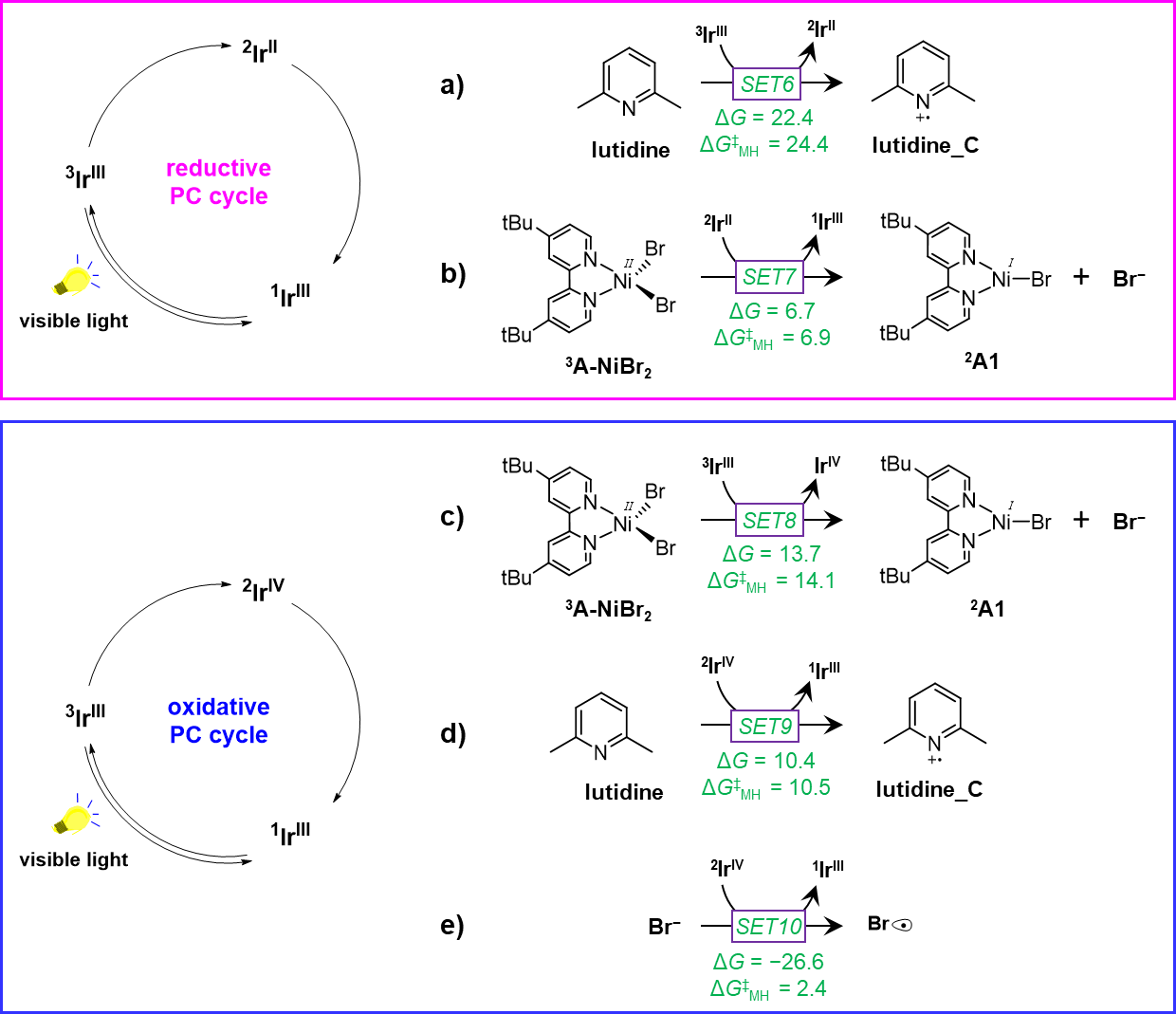


Supplementary Figure ****15**.** Energetics of SET steps involve in the activation of nickel precatalyst. The energy values are at M06(SMD-THF)/def2-TZVPP//PBE/def2-SVP/def2-TZVP.

**SETs involve in Ni-catalyst pathways (**Supplementary Figure ****16**):** *E*-product formation pathway with dtbbpy ligand has been considered as reference to check the feasibility of photocatalytic cycles. Along the reductive cycle **^3^Ir^III^** is reduced to **Ir^II^** by oxidation of Br^−^ to Br• (SET2), and so-formed **^2^Ir^II^** is then oxidized to **^1^Ir^III^** by the reduction of LNi(allyl)Br complex **^1^A2_E_** (SET1). Along the oxidative cycle **^3^Ir^III^** is oxidized to **^2^Ir^IV^** by the reduction of **^1^A2_E_** to **^1^A3_E_** (SET11), and the so-formed **^2^Ir^IV^** is then reduced to **^1^Ir^III^** by the oxidation of Br^−^ (SET10). Calculated free energy change and extimated barrier of SETs indicate that the reductive cycle is favored than the oxidative cycle Ni-catalytic pathway.


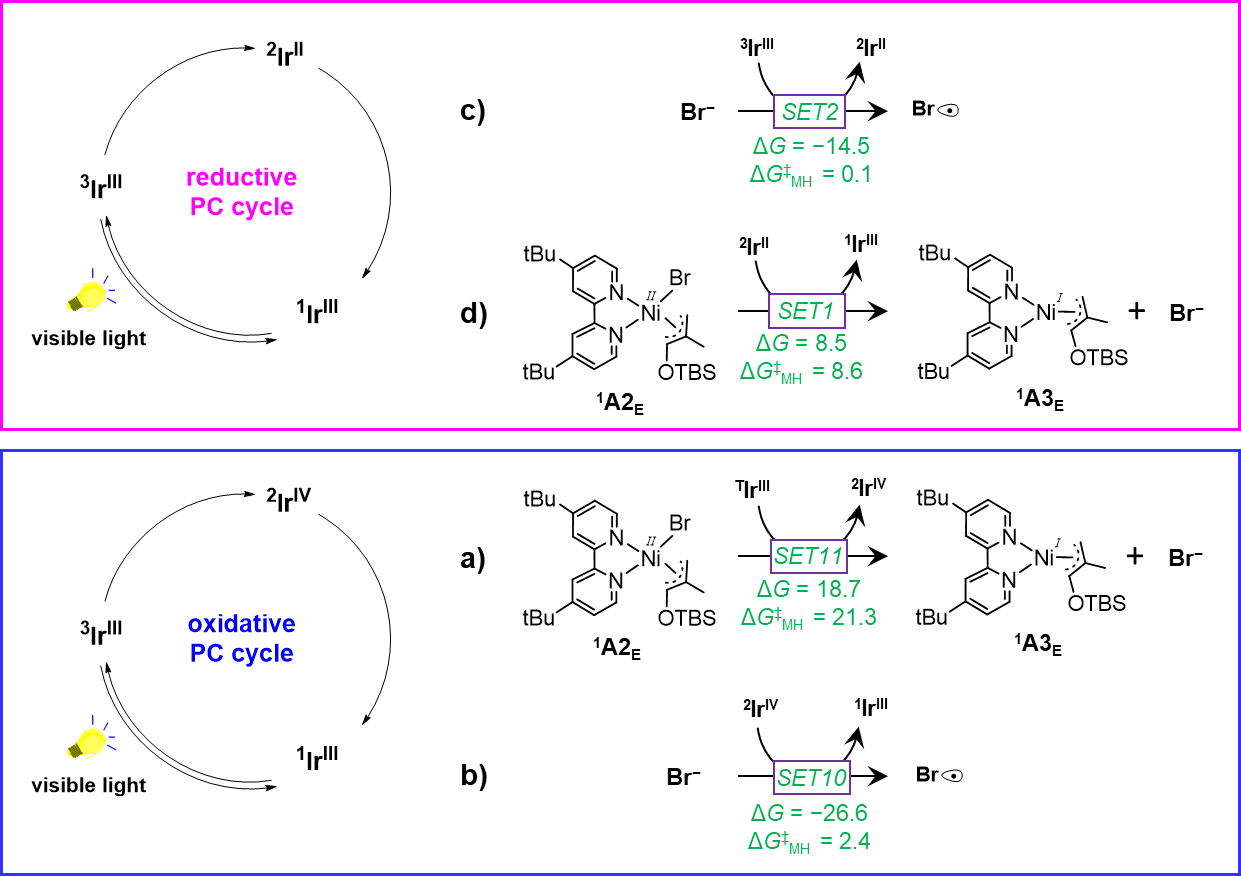


Supplementary Figure ****16**.** Energetics of SET steps involve in the nickel catalytic cycle. For energy conventions refer Supplementary Figure 15.

Supplementary Figure ****17**.** Free energy profile of Ni-catalytic cycle in presence of dtbbpy ligand for *Z*-product (**4**) formation. For energy conventions refer Supplementary Figure 15.

Supplementary Figure 18**.** Free energy profile of Ni-catalytic cycle in presence of 6,6′-dmbpy ligand for *E*-product (**3**) formation. For energy conventions refer Supplementary Figure 15.

Supplementary Figure 19**.** Free energy profile of Ni-catalytic cycle in presence of 6,6′-dmbpy ligand for *Z*-product (**4**) formation. For energy conventions refer Supplementary Figure 15.

Supplementary Figure 20**.** Calculated absorption spectra of **^1^A2_E_** and **^1^B2_E_** at M06(SMD-THF)/def2-TZVPP level of theory.

Supplementary Figure 21**.** a) Free-energy profiles for isomerization of allyl group and b) optimized geometry of **^1^A2_E_**. For energy conventions refer Supplementary Figure 15.

Supplementary Figure 22**.** a) Free-energy profiles for isomerization of allyl group and b) optimized geometry of **^1^B2_E_**. For energy conventions refer Supplementary Figure 15.

Supplementary Figure 23**.** Free-energy profiles for alternative isomerization of allyl group at LNi(II)(allyl)Br complex for dtbbpy ligand. For energy conventions refer Supplementary Figure 15.

We have considered to DFT study of E→Z isomerization of allyl radical via the transition state of **[R_E_-R_Z_]^‡^** in an outer sphere mechanism (Supplementary Figure 24). The overall free energy barrier requires for this isomerization is 22.9 kcal/mol, which is quite higher than that of the inner sphere mechanism (Δ*G*^‡^=10.8 kcal/mol) via transition state of **^3^[B2_E_-B2_Z_]^‡^** (Figure 7a).

Supplementary Figure 24**.** Free energy profile of *E*→*Z* isomerization of allyl radical in outer sphere mechanism.

**References for density functional theory (DFT) calculations**

[1] Gaussian 16, Revision **B.01**, Frisch, M. J., Trucks, G. W., Schlegel, H. B., Scuseria, G. E., Robb, M. A., Cheeseman, J. R., Scalmani, G., Barone, V., Petersson, G. A., Nakatsuji, H., Li, X., Caricato, M., Marenich, A. V., Bloino, J., Janesko, B. G., Gomperts, R., Mennucci, B., Hratchian, H. P., Ortiz, J. V., Izmaylov, A. F., Sonnenberg, J. L., Williams-Young, D., Ding, F., Lipparini, F., Egidi, F., Goings, J., Peng, B., Petrone, A., Henderson, T., Ranasinghe, D., Zakrzewski, V. G., Gao, J., Rega, N., Zheng, G., Liang, W., Hada, M., Ehara, M., Toyota, K., Fukuda, R., Hasegawa, J., Ishida, M., Nakajima, T., Honda, Y., Kitao, O., Nakai, H., Vreven, T., Throssell, K., Montgomery, Jr., J. A., Peralta, J. E., Ogliaro, F., Bearpark, M. J., Heyd, J. J., Brothers, E. N., Kudin, K. N., Staroverov, V. N., Keith, T. A., Kobayashi, R., Normand, J., Raghavachari, K., Rendell, A. P., Burant, J. C., Iyengar, S. S., Tomasi, J., Cossi, M., Millam, J. M., Klene, M., Adamo, C., Cammi, R., Ochterski, J. W., Martin, R. L., Morokuma, K., Farkas, O., Foresman, J. B., Fox, D. J. Gaussian, Inc., Wallingford CT, **2016**.

[2] Perdew, J. P.; Burke, K.; Ernzerhof, M., Generalized gradient approximation made simple. *Phys. Rev. Lett*. **1996**, *77*, 3865-3868.

[3] Weigend, F.; Ahlrichs, R., Balanced basis sets of split valence, triple zeta valence and quadruple zeta valence quality for H to Rn: Design and assessment of accuracy. *Phys. Chem. Chem. Phys*. **2005**, *7*, 3297-3305.

[4] Zhao, Y.; Truhlar, D. G., The M06 suite of density functionals for main group thermochemistry, thermochemical kinetics, noncovalent interactions, excited states, and transition elements: two new functionals and systematic testing of four M06-class functionals and 12 other functionals. *Theor. Chem. Acc*. **2008**, *120*, 215-241.

[5] Marenich, A. V.; Cramer, C. J.; Truhlar, D. G., Universal Solvation Model Based on Solute Electron Density and on a Continuum Model of the Solvent Defined by the Bulk Dielectric Constant and Atomic Surface Tensions. *J. Phys. Chem. B* **2009**, *113*, 6378-6396.

[6] a) Kelly, C. P.; Cramer, C. J.; Truhlar, D. G., SM6:  A Density Functional Theory Continuum Solvation Model for Calculating Aqueous Solvation Free Energies of Neutrals, Ions, and Solute−Water Clusters. *J. Chem. Theory Comput*. **2005**, *1*, 1133–1152; b) Kelly, C. P.; Cramer, C. J.; Truhlar, D. G., Aqueous Solvation Free Energies of Ions and Ion−Water Clusters Based on an Accurate Value for the Absolute Aqueous Solvation Free Energy of the Proton. *J. Phys. Chem. B* **2006**, *110*, 16066–16081

[7] Maity, B.; Koley, D.; Gooßen, L. J., Computational study of the mechanism and selectivity of ruthenium-catalyzed hydroamidations of terminal alkynes. *Chem. Sci*. **2015**, *6*, 2532–2552

[8] a) Hush, N. S., Adiabatic Rate Processes at Electrodes .1. Energy-Charge Relationships. *J. Chem. Phys*. **1958**, *28*, 962-972; b) Hush, N. S., Adiabatic Theory of Outer Sphere Electron-Transfer Reactions in Solution. *T. Faraday Soc*. **1961**, *57*, 557; c) Marcus, R. A., On the Theory of Oxidation-Reduction Reactions Involving Electron Transfer. *J. Chem. Phys*. **1956**, *24*, 966-978; d) Marcus, R. A., Electrostatic Free Energy and Other Properties of States Having Nonequilibrium Polarization. *J. Chem. Phys*. **1956**, *24*, 979-989; e) Marcus, R. A., Theory of Oxidation-Reduction Reactions Involving Electron Transfer .3. Applications to Data on the Rates of Organic Redox Reactions. *J. Chem. Phys*. **1957**, *26*, 872-877; f) Marcus, R. A., On the Theory of Electrochemical and Chemical Electron Transfer Processes. *Can. J. Chem*. **1959**, *37*, 155-163; g) Marcus, R. A., The 2nd Robinson, R. A. Memorial Lecture - Electron, Proton and Related Transfers. *Faraday Discuss*. **1982**, *74*, 7-15; h) Marcus, R. A.; Sutin, N., Electron Transfers in Chemistry and Biology. *Biochim Biophys Acta* **1985**, *811*, 265-322.

**Reaction setup**

**
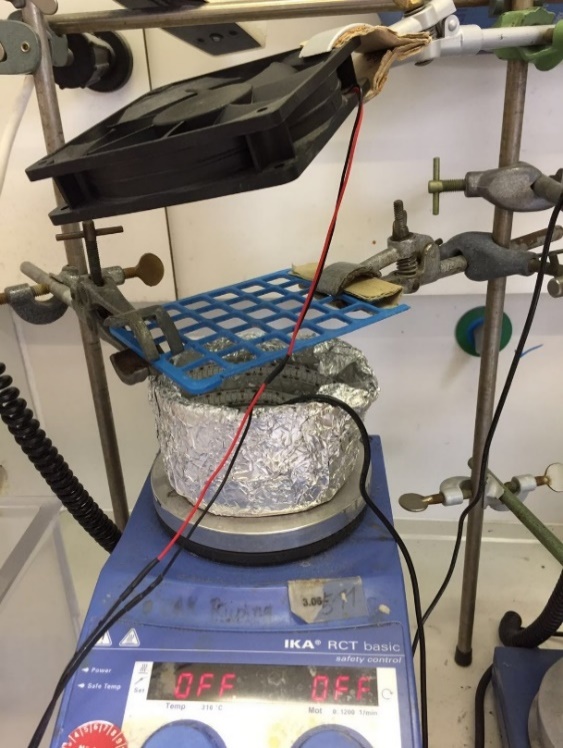
**

Reaction setup: Photoreactor was assembled by wrapping the 2 m Blue LED strips (24 V) inside of a Pyrex crystallizing dish (~12 cm diameter). The sample was positioned 2-3 cm from strips. A fan was installed above to ensure reactions remained near rt (typically 5-10 °C above) when using LEDs.

1. **Supplementary Data**

**Characterization of the Products**

**Product 3**: the title compound was prepared following the general procedure **E** using NiBr_2_ (4.4 mg, 0.02 mmol, 10 mol%), 4,4'-di-*tert*-butyl-2,2'-bipyridine (8.1 mg, 0.03 mmol, 15 mol%), methyl 4-bromobenzoate (43 mg, 0.2 mmol, 1 equiv.), Ir[dF(CF_3_)ppy]_2_(dtbpy)PF_6_ (2.3 mg, 0.002 mmol, 1 mol%), 2,6-lutidine (32.1 mg (⁓35 µL), 0.3 mmol, 1.5 equiv.) and *tert*-butyldimethyl((2-methylprop-1-en-1-yl)oxy)silane (112 mg, 0.6 mmol, 3 equiv.). Dissolved in the mixture solvent of benzene (1 mL) and THF (1 mL). After light irradiation with a 2 m blue LED strip at room temperature for 48 h, the crude product (93:7 *E*/*Z*) was purified by flash column chromatography to afford **3** as a colorless oil in 77% yield (93:7 *E*/*Z*, 49.4 mg).

**^1^H NMR (400 MHz, CDCl_3_, mixture of isomers *E*/*Z*, 93:7):** ***E* isomer** (major) δ 7.88 (d, *J* = 7.8 Hz, 2H), 7.17 (d, *J* = 8.0 Hz, 2H), 6.17 (s, 1H), 3.83 (s, 3H), 3.15 (s, 2H), 1.43 (s, 3H), 0.87 (s, 9H), 0.08 (s, 6H); ***Z* isomer** (minor) 6.13 (s, 1H), 3.39 (s, 2H) the remaining resonances are insufficiently resolved from those of the ***E* isomer** to be reported.

**^13^C NMR (101 MHz, CDCl_3_, mixture of isomers *E*/*Z*, 93:7):** ***E* isomer** (major) δ 167.3, 146.4, 136.1, 129.7, 128.8, 128.1, 116.1, 52.1, 40.4, 25.8, 18.4, 12.7, -5.1; ***Z* isomer** (minor) 135.0, 52.0, 35.3, 17.0, -5.1 the remaining resonances are insufficiently resolved from those of the ***E* isomer** to be reported.

IR (ATR) ν 2953, 2932, 2858, 1723, 1673, 1610, 1465, 1435, 1276, 1153, 1107, 1015, 836, 780, 754 cm^-1^.

HRMS (ESI) for C_18_H_28_O_3_NaSi^+^ (M+Na)^+^ : 343.16999; Found : 343.16965.

**Product 4**: the title compound was prepared following the general procedure **E** using NiBr_2_ (8.8 mg, 0.04 mmol, 20 mol%), 6,6′-dimethyl-2,2′-bipyridine (7.4 mg, 0.04 mmol, 20 mol%), methyl 4-bromobenzoate (43 mg, 0.2 mmol, 1 equiv.), Ir[dF(CF_3_)ppy]_2_(dtbpy)PF_6_ (2.3 mg, 0.002 mmol, 1 mol%), 2,4,6-collidine (72.7 mg (⁓80 µL), 0.6 mmol, 3.0 equiv.) and *tert*-butyldimethyl((2-methylprop-1-en-1-yl)oxy)silane (112 mg, 0.6 mmol, 3 equiv.). Dissolved in the mixture solvent of dioxane (1 mL) and THF (1 mL). After light irradiation with a 2 m blue LED strip at room temperature for 72 h, the crude product (10:90 *E*/*Z*) was purified by flash column chromatography to afford **4** as a colorless oil in 66% yield (only Z, 42.2 mg).

**^1^H NMR (400 MHz, CDCl_3_, only *Z*):** δ 7.92 (d, *J* = 8.2 Hz, 2H), 7.24 (d, *J* = 8.1 Hz, 2H), 6.17 (s, 1H), 3.87 (s, 3H), 3.44 (s, 2H), 1.42 (d, *J* = 1.5 Hz, 3H), 0.92 (s, 9H), 0.13 (s, 6H).

**^13^C NMR (101 MHz, CDCl_3_, only *Z*):** δ 167.4, 146.8, 134.9, 129.7, 128.8, 127.7, 114.9, 52.0, 35.3, 25.8, 18.3, 17.0, -5.1.

IR (ATR) ν 2953, 2932, 2858, 1721, 1675, 1610, 1436, 1276, 1182, 1139, 1108, 1015, 838, 780 cm^-1^;

HRMS (ESI) for C_18_H_28_O_3_NaSi^+^ (M+Na)^+^ : 343.16999; Found : 343.16950.

**Product 5**: the title compound was prepared following the general procedure **E** using NiBr_2_ (4.4 mg, 0.02 mmol, 10 mol%), 4,4'-di-*tert*-butyl-2,2'-bipyridine (8.1 mg, 0.15 mmol, 15 mol%), 1-(4-bromophenyl)ethan-1-one (39.9 mg, 0.2 mmol, 1 equiv.), Ir[dF(CF_3_)ppy]_2_(dtbpy)PF_6_ (2.3 mg, 0.002 mmol, 1 mol%), 2,6-lutidine (32.1 mg (⁓35 µL), 0.3 mmol, 1.5 equiv.) and *tert*-butyldimethyl((2-methylprop-1-en-1-yl)oxy)silane (112 mg, 0.6 mmol, 3 equiv.). Dissolved in the mixture solvent of benzene (1 mL) and THF (1 mL). After light irradiation with a 2 m blue LED strip at room temperature for 48 h, the crude product (92:8 *E*/*Z*) was purified by flash column chromatography to afford **5** as a colorless oil in 83% yield (93:7 *E*/*Z*, 50.6 mg).

**^1^H NMR (400 MHz, CDCl_3_, mixture of isomers *E*/*Z*, 93:7):** ***E* isomer** (major) δ 7.86 (d, *J* = 8.0 Hz, 2H), 7.24 (d, *J* = 7.9 Hz, 2H), 6.22 (s, 1H), 3.20 (s, 2H), 2.56 (s, 3H), 1.52 – 1.40 (m, 3H), 0.92 (s, 9H), 0.12 (s, 6H); ***Z* isomer** (minor) 6.17 (s, 1H), 3.44 (s, 2H) the remaining resonances are insufficiently resolved from those of the ***E* isomer** to be reported.

**^13^C NMR (101 MHz, CDCl_3_, mixture of isomers *E*/*Z*, 93:7):** ***E* isomer** (major) δ 198.0, 146.7, 136.1, 135.3, 129.0, 128.5, 115.9, 40.4, 35.2, 26.7, 25.8, 18.4, 17.0, 12.7, -5.1, -5.1; ***Z* isomer** (minor) 35.2, 17.0, -5.1 the remaining resonances are insufficiently resolved from those of the ***E* isomer** to be reported.

IR (ATR) ν 2931, 2858, 1680, 1606, 1466, 1412, 1358, 1260, 1190, 1153, 837, 779 cm^-1^;

HRMS (APCI) for C_18_H_29_O_2_Si^+^ (M+H)^+^ : 305.19314; Found : 305.19358.

**Product 6**: the title compound was prepared following the general procedure **E** using NiBr_2_ (4.4 mg, 0.02 mmol, 10 mol%), 4,4'-di-*tert*-butyl-2,2'-bipyridine (8.1 mg, 0.15 mmol, 15 mol%), 4-bromobenzonitrile (36.4 mg, 0.2 mmol, 1 equiv.), Ir[dF(CF_3_)ppy]_2_(dtbpy)PF_6_ (2.3 mg, 0.002 mmol, 1 mol%), 2,6-lutidine (32.1 mg (⁓35 µL), 0.3 mmol, 1.5 equiv.) and *tert*-butyldimethyl((2-methylprop-1-en-1-yl)oxy)silane (112 mg, 0.6 mmol, 3 equiv.). Dissolved in the mixture solvent of benzene (1 mL) and THF (1 mL). After light irradiation with a 2 m blue LED strip at room temperature for 48 h, the crude product (89:11 *E*/*Z*) was purified by flash column chromatography to afford **6** as a colorless oil in 69% yield (89:11 *E*/*Z*, 39.8 mg).

**^1^H NMR (600 MHz, CDCl_3_, mixture of isomers *E*/*Z*, 89:11):** ***E* isomer** (major) δ 7.57 (d, *J* = 8.0 Hz, 2H), 7.28 (d, *J* = 8.1 Hz, 2H), 6.25 (s, 1H), 3.23 (s, 2H), 1.49 (s, 3H), 0.95 (s, 9H), 0.16 (s, 6H); ***Z* isomer** (minor) 6.21 (s, 1H), 3.46 (s, 2H), 1.46 (s, 3H) the remaining resonances are insufficiently resolved from those of the ***E* isomer** to be reported.

**^13^C NMR (151 MHz, CDCl_3_, mixture of isomers *E*/*Z*, 89:11):** ***E* isomer** (major) δ 146.7, 136.5, 132.2, 129.5, 119.3, 115.3, 110.0, 40.5, 25.8, 18.4, 12.7, -5.1; ***Z* isomer** (minor) 147.0, 135.4, 132.1, 129.5, 119.4, 114.2, 109.6, 35.4, 18.3, 17.0, -5.1.

IR (ATR) ν 2931, 2858, 2228, 1672, 1607, 1466, 1254, 1193, 1154, 1008, 838, 780 cm^-1^;

HRMS (ESI) for C_17_H_25_ONNaSi^+^ (M+Na)^+^ : 310.15976; Found : 310.15891.

**Product 7**: the title compound was prepared following the general procedure **E** using NiBr_2_ (4.4 mg, 0.02 mmol, 10 mol%), 4,4'-di-*tert*-butyl-2,2'-bipyridine (8.1 mg, 0.15 mmol, 15 mol%), 1-bromo-4-(trifluoromethyl)benzene (45 mg, 0.2 mmol, 1 equiv.), Ir[dF(CF_3_)ppy]_2_(dtbpy)PF_6_ (2.3 mg, 0.002 mmol, 1 mol%), 2,6-lutidine (32.1 mg (⁓35 µL), 0.3 mmol, 1.5 equiv.) and *tert*-butyldimethyl((2-methylprop-1-en-1-yl)oxy)silane (112 mg, 0.6 mmol, 3 equiv.). Dissolved in the mixture solvent of benzene (1 mL) and THF (1 mL). After light irradiation with a 2 m blue LED strip at room temperature for 48 h, the crude product (92:8 *E*/*Z*) was purified by flash column chromatography to afford **7** as a colorless oil in 66% yield (pure *E* isomer, 43.8 mg).

**^1^H NMR (600 MHz, CDCl_3_, pure *E* isomer):** δ 7.53 (d, *J* = 7.9 Hz, 2H), 7.29 (d, *J* = 7.9 Hz, 2H), 6.26 (s, 1H), 3.23 (s, 2H), 1.50 (s, 3H), 0.95 (s, 9H), 0.16 (s, 6H).

**^13^C NMR (151 MHz, CDCl_3_, pure *E* isomer):** δ 145.0, 136.2, 129.1, 128.5 (q, *J*_C-F_ = 32.0 Hz), 125.3 (q, *J* _C-F_ = 3.8 Hz), 124.5 (q, *J*_C-F_ = 272.0 Hz), 115.9, 40.2, 25.8, 18.4, 12.7, -5.1.

**^19^F NMR (565 MHz, CDCl_3_, pure *E* isomer):** δ -62.28.

IR (ATR) ν 2932, 2859, 2325, 2112, 1673, 1619, 1467, 1324, 1255, 1194, 1159, 1124, 1066, 1016, 837, 780 cm^-1^;

HRMS (EI) for C_17_H_25_OF_3_Si^+^ (M)^+^ : 330.16213; Found : 330.16243.

**Product 8**: the title compound was prepared following the general procedure **E** using NiBr_2_ (4.4 mg, 0.02 mmol, 10 mol%), 4,4'-di-*tert*-butyl-2,2'-bipyridine (8.1 mg, 0.15 mmol, 15 mol%), 1-bromo-4-(methylsulfonyl)benzene (47 mg, 0.2 mmol, 1 equiv.), Ir[dF(CF_3_)ppy]_2_(dtbpy)PF_6_ (2.3 mg, 0.002 mmol, 1 mol%), 2,6-lutidine (32.1 mg (⁓35 µL), 0.3 mmol, 1.5 equiv.) and *tert*-butyldimethyl((2-methylprop-1-en-1-yl)oxy)silane (112 mg, 0.6 mmol, 3 equiv.). Dissolved in the mixture solvent of benzene (1 mL) and THF (1 mL). After light irradiation with a 2 m blue LED strip at room temperature for 48 h, the crude product (91:9 *E*/*Z*) was purified by flash column chromatography to afford **8** as a white solid in 68% yield (92:8 *E*/*Z*, 46.0 mg).

**^1^H NMR (600 MHz, CDCl_3_, mixture of isomers *E*/*Z*, 92:8):** ***E* isomer** (major) δ 7.84 (d, *J* = 8.2 Hz, 2H), 7.36 (d, *J* = 8.0 Hz, 2H), 6.25 (s, 1H), 3.25 (s, 2H), 3.03 (s, 3H), 1.48 (s, 3H), 0.93 (s, 9H), 0.14 (s, 6H); ***Z* isomer** (minor) 6.20 (s, 1H), 3.47 (s, 2H), 1.45 (s, 3H) the remaining resonances are insufficiently resolved from those of the ***E* isomer** to be reported.

**^13^C NMR (151 MHz, CDCl_3_, mixture of isomers *E*/*Z*, 92:8):** ***E* isomer** (major) δ 148.0, 138.3, 136.5, 129.6, 127.5, 115.3, 44.7, 40.3, 25.8, 18.4, 12.7, -5.1; ***Z* isomer** (minor) 147.6, 138.0, 135.4, 127.4, 114.3, 35.2, 17.0, -5.1, the remaining resonances are insufficiently resolved from those of the ***E* isomer** to be reported.

IR (ATR) ν 2931, 2857, 1719, 1672, 1597, 1467, 1408, 1305, 1255, 1192, 1146, 1090, 956, 838, 770 cm^-1^;

HRMS (EI) for C_17_H_28_O_3_SSi^+^ (M)^+^ : 340.15230; Found : 340.15277.

**Product 9**: the title compound was prepared following the general procedure **E** using NiBr_2_ (4.4 mg, 0.02 mmol, 10 mol%), 4,4'-di-*tert*-butyl-2,2'-bipyridine (8.1 mg, 0.03 mmol, 15 mol%), 4-bromobenzenesulfonamide (47.2 mg, 0.2 mmol, 1 equiv.), Ir[dF(CF_3_)ppy]_2_(dtbpy)PF_6_ (2.3 mg, 0.002 mmol, 1 mol%), 2,6-lutidine (32.1 mg (⁓35 µL), 0.3 mmol, 1.5 equiv.) and *tert*-butyldimethyl((2-methylprop-1-en-1-yl)oxy)silane (112 mg, 0.6 mmol, 3 equiv.). Dissolved in the mixture solvent of benzene (1 mL) and THF (1 mL). After light irradiation with a 2 m blue LED strip at room temperature for 48 h, the crude product (92:8 *E*/*Z*) was purified by flash column chromatography to afford **9** as a white solid in 72% yield (93:7 *E*/*Z*, 48.9 mg).

**^1^H NMR (600 MHz, CDCl_3_, mixture of isomers *E*/*Z*, 93:7):** ***E* isomer** (major) δ 7.83 (d, *J* = 8.0 Hz, 2H), 7.30 (d, *J* = 7.9 Hz, 2H), 6.25 (s, 1H), 5.10 (s, 2H), 3.23 (s, 2H), 1.48 (s, 3H), 0.94 (s, 9H), 0.15 (s, 6H); ***Z* isomer** (minor) 6.20 (s, 1H), 3.46 (s, 2H), 1.44 (s, 3H), the remaining resonances are insufficiently resolved from those of the ***E* isomer** to be reported.

**^13^C NMR (151 MHz, CDCl_3_, mixture of isomers *E*/*Z*, 93:7):** ***E* isomer** (major) δ 146.5, 139.7, 136.4, 129.4, 128.1, 126.5, 115.5, 40.2, 25.8, 18.4, 12.7, -5.1; ***Z* isomer** (minor) 146.9, 139.3, 135.3, 129.5, 126.5, 114.4, 35.1, 18.3, 16.9, -5.1, the remaining resonances are insufficiently resolved from those of the ***E* isomer** to be reported.

IR (ATR) ν 3401, 3268, 2931, 2895, 2857, 1673, 1597, 1556, 1467, 1407, 1329, 1255, 1146, 1097, 1009, 911, 840, 776, 733 cm^-1^;

HRMS (ESI) for C_16_H_27_O_3_NNaSSi^+^ (M+Na)^+^ : 364.13731; Found : 364.13708.

**Prodcut 10**: the title compound was prepared following the general procedure **E** using NiBr_2_ (4.4 mg, 0.02 mmol, 10 mol%), 4,4'-di-*tert*-butyl-2,2'-bipyridine (8.1 mg, 0.15 mmol, 15 mol%), (4-bromophenyl)(phenyl)methanone (52.2 mg, 0.2 mmol, 1 equiv.), Ir[dF(CF_3_)ppy]_2_(dtbpy)PF_6_ (2.3 mg, 0.002 mmol, 1 mol%), 2,6-lutidine (32.1 mg (⁓35 µL), 0.3 mmol, 1.5 equiv.) and *tert*-butyldimethyl((2-methylprop-1-en-1-yl)oxy)silane (112 mg, 0.6 mmol, 3 equiv.). Dissolved in the mixture solvent of benzene (1 mL) and THF (1 mL). After light irradiation with a 2 m blue LED strip at room temperature for 48 h, the crude product (90:10 *E*/*Z*) was purified by flash column chromatography to afford **10** as a colorless oil in 72% yield (pure *E* isomer, 52.9 mg).

**^1^H NMR (400 MHz, CDCl_3_, pure *E* isomer):** δ 7.79 (d, *J* = 7.4 Hz, 2H), 7.74 (d, *J* = 7.8 Hz, 2H), 7.58 (t, *J* = 7.4 Hz, 1H), 7.48 (t, *J* = 7.5 Hz, 2H), 7.29 (d, *J* = 7.8 Hz, 2H), 6.27 (s, 1H), 3.25 (s, 2H), 1.53 (s, 3H), 0.95 (s, 9H), 0.15 (s, 6H).

**^13^C NMR (101 MHz, CDCl_3_, pure *E* isomer):** δ 196.7, 146.1, 138.0, 136.1, 135.5, 132.3, 130.4, 130.1, 128.7, 128.3, 116.0, 40.4, 25.8, 18.4, 12.8, -5.1.

IR (ATR) ν 2930, 2857, 1652, 1603, 1463, 1313, 1273, 1187, 1152, 922, 834, 777 cm^-1^;

HRMS (APCI) for C_23_H_31_O_2_Si^+^ (M+H)^+^ : 367.20879; Found : 367.20966.

**Product 11**: the title compound was prepared following the general procedure **E** using NiBr_2_ (4.4 mg, 0.02 mmol, 10 mol%), 4,4'-di-*tert*-butyl-2,2'-bipyridine (8.1 mg, 0.03 mmol, 15 mol%), 1-bromo-4-chlorobenzene (38.3 mg, 0.2 mmol, 1 equiv.), Ir[dF(CF_3_)ppy]_2_(dtbpy)PF_6_ (2.3 mg, 0.002 mmol, 1 mol%), 2,6-lutidine (32.1 mg (⁓35 µL), 0.3 mmol, 1.5 equiv.) and *tert*-butyldimethyl((2-methylprop-1-en-1-yl)oxy)silane (112 mg, 0.6 mmol, 3 equiv.). Dissolved in the mixture solvent of benzene (1 mL) and THF (1 mL). After light irradiation with a 2 m blue LED strip at room temperature for 48 h, the crude product (94:6 *E*/*Z*) was purified by flash column chromatography to afford **11** as a colorless oil in 60% yield (only *E* isomer, 35.5 mg).

**^1^H NMR (400 MHz, CDCl_3_, only *E* isomer):** δ 7.23 (d, *J* = 8.3 Hz, 2H), 7.09 (d, *J* = 8.0 Hz, 2H), 6.21 (s, 1H), 3.13 (s, 2H), 1.48 (s, 3H), 0.94 (s, 9H), 0.14 (s, 6H).

**^13^C NMR (101 MHz, CDCl_3_, only *E* isomer):** δ 139.2, 135.8, 131.7, 130.1, 128.4, 116.4, 39.7, 25.8, 18.4, 12.7, -5.1.

IR (ATR) ν 2931, 2858, 1672, 1488, 1255, 1193, 1153, 1091, 1012, 838, 779 cm^-1^;

HRMS (APCI) for C_16_H_26_OClSi^+^ (M+H)^+^ : 297.14360; Found : 297.14427.

**Product 12**: the title compound was prepared following the general procedure **E** using NiBr_2_ (4.4 mg, 0.02 mmol, 10 mol%), 4,4'-di-*tert*-butyl-2,2'-bipyridine (8.1 mg, 0.15 mmol, 15 mol%), bromobenzene (31.4 mg, 0.2 mmol, 1 equiv.), Ir[dF(CF_3_)ppy]_2_(dtbpy)PF_6_ (2.3 mg, 0.002 mmol, 1 mol%), 2,6-lutidine (32.1 mg (⁓35 µL), 0.3 mmol, 1.5 equiv.) and *tert*-butyldimethyl((2-methylprop-1-en-1-yl)oxy)silane (112 mg, 0.6 mmol, 3 equiv.). Dissolved in the mixture solvent of benzene (1 mL) and THF (1 mL). After light irradiation with a 2 m blue LED strip at room temperature for 42 h, the crude product (95:5 *E*/*Z*) was purified by flash column chromatography to afford **12** as a colorless oil in 66% yield (pure *E* isomer, 34.4 mg).

**^1^H NMR (400 MHz, CDCl_3_, *E* isomer only):** δ 7.47 – 7.05 (m, 5H), 6.22 (s, 1H), 3.17 (s, 2H), 1.51 (s, 3H), 0.94 (s, 9H), 0.14 (s, 6H).

**^13^C NMR (101 MHz, CDCl_3_, *E* isomer only):** δ 140.7, 135.5, 128.8, 128.3, 126.0, 116.9, 40.4, 25.9, 18.4, 12.7, -5.1.

IR (ATR) ν 2930, 2857, 1672, 1462, 1389, 1253, 1193, 1153, 1005, 838, 779 cm^-1^;

HRMS (EI) for C_16_H_26_OSi^+^ (M)^+^ : 262.17475; Found : 262.17487.

**Product 13**: the title compound was prepared following the general procedure **E** using NiBr_2_ (4.4 mg, 0.02 mmol, 10 mol%), 4,4'-di-*tert*-butyl-2,2'-bipyridine (8.1 mg, 0.15 mmol, 15 mol%), 1-bromo-4-methylbenzene (34.2 mg, 0.2 mmol, 1 equiv.), Ir[dF(CF_3_)ppy]_2_(dtbpy)PF_6_ (2.3 mg, 0.002 mmol, 1 mol%), 2,6-lutidine (32.1 mg (⁓35 µL), 0.3 mmol, 1.5 equiv.) and *tert*-butyldimethyl((2-methylprop-1-en-1-yl)oxy)silane (112 mg, 0.6 mmol, 3 equiv.). Dissolved in the mixture solvent of benzene (1 mL) and THF (1 mL). After light irradiation with a 2 m blue LED strip at room temperature for 48 h, the crude product (95:5 *E*/*Z*) was purified by flash column chromatography to afford **13** as a colorless oil in 58% yield (pure *E* isomer, 31.9 mg).

**^1^H NMR (600 MHz, CDCl_3_, *E* isomer only):** δ 7.10 (d, *J* = 7.7 Hz, 2H), 7.08 (d, *J* = 7.9 Hz, 2H), 6.24 (s, 1H), 3.15 (s, 2H), 2.34 (s, 3H), 1.52 (s, 3H), 0.96 (s, 9H), 0.16 (s, 6H).

**^13^C NMR (151 MHz, CDCl_3_, *E* isomer only):** δ 137.6, 135.4, 129.0, 128.7, 117.1, 39.9, 25.9, 21.2, 18.4, 12.7, -5.1.

IR (ATR) ν 2930, 2858, 1672, 1512, 1465, 1388, 1254, 1192, 1151, 1006, 839, 779 cm^-1^;

HRMS (EI) for C_17_H_28_OSi^+^ (M)^+^ : 276.19040; Found : 276.19107.

**Product 14**: the title compound was prepared following the general procedure **E** using NiBr_2_ (4.4 mg, 0.02 mmol, 10 mol%), 4,4'-di-*tert*-butyl-2,2'-bipyridine (8.1 mg, 0.15 mmol, 15 mol%), 1-bromo-4-(*tert*-butyl)benzene (42.6 mg, 0.2 mmol, 1 equiv.), Ir[dF(CF_3_)ppy]_2_(dtbpy)PF_6_ (2.3 mg, 0.002 mmol, 1 mol%), 2,6-lutidine (32.1 mg (⁓35 µL), 0.3 mmol, 1.5 equiv.) and *tert*-butyldimethyl((2-methylprop-1-en-1-yl)oxy)silane (112 mg, 0.6 mmol, 3 equiv.). Dissolved in the mixture solvent of benzene (1 mL) and THF (1 mL). After light irradiation with a 2 m blue LED strip at room temperature for 48 h, the crude product (95:5 *E*/*Z*) was purified by flash column chromatography to afford **14** as a colorless oil in 60% yield (pure *E* isomer, 38.4 mg).

**^1^H NMR (600 MHz, CDCl_3_, *E* isomer only):** δ 7.31 (d, *J* = 8.3 Hz, 2H), 7.12 (d, *J* = 8.2 Hz, 2H), 6.24 (s, 1H), 3.17 (s, 2H), 1.54 (s, 3H), 1.33 (s, 9H), 0.97 (s, 9H), 0.17 (s, 6H).

**^13^C NMR (151 MHz, CDCl_3_, *E* isomer only):** δ 148.8, 137.7, 135.5, 128.4, 125.2, 116.9, 39.8, 34.5, 31.6, 25.9, 18.4, 12.8, -5.1.

IR (ATR) ν 2956, 2859, 2324, 2106, 1898, 1672, 1466, 1362, 1255, 1192, 1152, 914, 838, 779 cm^-1^;

HRMS (EI) for C_20_H_34_OSi^+^ (M)^+^ : 318.23735; Found : 318.23735.

**Product 15**: the title compound was prepared following the general procedure **E** using NiBr_2_ (4.4 mg, 0.02 mmol, 10 mol%), 4,4'-di-*tert*-butyl-2,2'-bipyridine (8.1 mg, 0.15 mmol, 15 mol%), 1-bromo-4-(*tert*-butoxy)benzene (45.8 mg, 0.2 mmol, 1 equiv.), Ir[dF(CF_3_)ppy]_2_(dtbpy)PF_6_ (2.3 mg, 0.002 mmol, 1 mol%), 2,6-lutidine (32.1 mg (⁓35 µL), 0.3 mmol, 1.5 equiv.) and *tert*-butyldimethyl((2-methylprop-1-en-1-yl)oxy)silane (112 mg, 0.6 mmol, 3 equiv.). Dissolved in the mixture solvent of benzene (1 mL) and THF (1 mL). After light irradiation with a 2 m blue LED strip at room temperature for 43 h, the crude product (94:6 *E*/*Z*) was purified by flash column chromatography to afford **15** as a colorless oil in 61% yield (97:3 *E*/*Z*, 40.8 mg).

**^1^H NMR (400 MHz, CDCl_3_, *E* isomer only):** δ 7.05 (d, *J* = 8.3 Hz, 2H), 6.89 (d, *J* = 8.3 Hz, 2H), 6.17 (d, *J* = 1.5 Hz, 1H), 3.13 (s, 2H), 1.51 (d, *J* = 1.4 Hz, 3H), 1.33 (s, 9H), 0.94 (s, 9H), 0.14 (s, 6H).

**^13^C NMR (101 MHz, CDCl_3_, *E* isomer only):** δ 153.5, 135.6, 135.4, 129.1, 124.1, 117.2, 78.2, 39.7, 29.0, 25.9, 18.4, 12.8, -5.1.

IR (ATR) ν 2957, 2858, 1673, 1504, 1467, 1364, 1251, 1153, 1008, 897, 837, 779 cm^-1^;

HRMS (EI) for C_20_H_34_O_2_Si^+^ (M)^+^ : 334.23226; Found : 334.23282.

**Product 16**: the title compound was prepared following the general procedure **E** using NiBr_2_ (4.4 mg, 0.02 mmol, 10 mol%), 4,4'-di-*tert*-butyl-2,2'-bipyridine (8.1 mg, 0.15 mmol, 15 mol%), 1-bromo-4-phenoxybenzene (49.8 mg, 0.2 mmol, 1 equiv.), Ir[dF(CF_3_)ppy]_2_(dtbpy)PF_6_ (2.3 mg, 0.002 mmol, 1 mol%), 2,6-lutidine (32.1 mg (⁓35 µL), 0.3 mmol, 1.5 equiv.) and *tert*-butyldimethyl((2-methylprop-1-en-1-yl)oxy)silane (112 mg, 0.6 mmol, 3 equiv.). Dissolved in the mixture solvent of benzene (1 mL) and THF (1 mL). After light irradiation with a 2 m blue LED strip at room temperature for 43 h, the crude product (96:4 *E*/*Z*) was purified by flash column chromatography to afford **16** as a colorless oil in 59% yield (pure *E* isomer, 41.5 mg).

**^1^H NMR (600 MHz, CDCl_3_, *E* isomer only):** δ 7.33 (t, *J* = 7.8 Hz, 2H), 7.14 (d, *J* = 8.0 Hz, 2H), 7.08 (t, *J* = 7.4 Hz, 1H), 7.00 (d, *J* = 8.0 Hz, 2H), 6.94 (d, *J* = 8.0 Hz, 2H), 6.23 (s, 1H), 3.17 (s, 2H), 1.54 (s, 3H), 0.96 (s, 9H), 0.16 (s, 6H).

**^13^C NMR (151 MHz, CDCl_3_, *E* isomer only):** δ 157.8, 155.3, 135.8, 135.6, 130.0, 129.8, 123.0, 119.1, 118.6, 116.9, 39.7, 25.9, 18.4, 12.8, -5.1.

IR (ATR) ν 2930, 2857, 2326, 2091, 1921, 1672, 1590, 1488, 1237, 1194, 1152, 1010, 837, 779 cm^-1^;

HRMS (EI) for C_22_H_30_O_2_Si^+^ (M)^+^ : 354.20096; Found : 354.20060.

**Product 17**: the title compound was prepared following the general procedure **E** using NiBr_2_ (4.4 mg, 0.02 mmol, 10 mol%), 4,4'-di-*tert*-butyl-2,2'-bipyridine (8.1 mg, 0.15 mmol, 15 mol%), 1-bromo-4-(trifluoromethoxy)benzene (48.2 mg, 0.2 mmol, 1 equiv.), Ir[dF(CF_3_)ppy]_2_(dtbpy)PF_6_ (2.3 mg, 0.002 mmol, 1 mol%), 2,6-lutidine (32.1 mg (⁓35 µL), 0.3 mmol, 1.5 equiv.) and *tert*-butyldimethyl((2-methylprop-1-en-1-yl)oxy)silane (112 mg, 0.6 mmol, 3 equiv.). Dissolved in the mixture solvent of benzene (1 mL) and THF (1 mL). After light irradiation with a 2 m blue LED strip at room temperature for 48 h, the crude product (95:5 *E*/*Z*) was purified by flash column chromatography to afford **17** as a colorless oil in 61% yield (*E*/Z 95:5, 42.2 mg).

**^1^H NMR (600 MHz, CDCl_3_, mixture of isomers *E*/*Z*, 95:5):** ***E* isomer** (major) δ 7.18 (d, *J* = 8.1 Hz, 2H), 7.12 (d, *J* = 8.3 Hz, 2H), 6.22 (s, 1H), 3.17 (s, 2H), 1.50 (s, 3H), 0.95 (s, 9H), 0.15 (s, 6H).

**^13^C NMR (151 MHz, CDCl_3_, mixture of isomers *E*/*Z*, 95:5):** ***E* isomer** (major) δ 147.5, 139.4, 135.8, 129.8, 120.5 (q, *J*_C-F_ = 257.5 Hz), 118.0, 116.1, 39.5, 25.7, 18.3, 12.5, -5.3

**^19^F NMR (565 MHz, CDCl_3_):** ***E* isomer** (major) δ -58.0.

IR (ATR) ν 2932, 2859, 1673, 1507, 1467, 1256, 1158, 837, 780 cm^-1^;

HRMS (EI) for C_17_H_25_O_2_F_2_Si^+^ (M)^+^ : 346.15704; Found : 346.15726.

**Product 18**: the title compound was prepared following the general procedure **E** using NiBr_2_ (4.4 mg, 0.02 mmol, 10 mol%), 4,4'-di-*tert*-butyl-2,2'-bipyridine (8.1 mg, 0.15 mmol, 15 mol%), 1-bromo-3-methoxybenzene (37.4 mg, 0.2 mmol, 1 equiv.), Ir[dF(CF_3_)ppy]_2_(dtbpy)PF_6_ (2.3 mg, 0.002 mmol, 1 mol%), 2,6-lutidine (32.1 mg (⁓35 µL), 0.3 mmol, 1.5 equiv.) and *tert*-butyldimethyl((2-methylprop-1-en-1-yl)oxy)silane (112 mg, 0.6 mmol, 3 equiv.). Dissolved in the mixture solvent of benzene (1 mL) and THF (1 mL). After light irradiation with a 2 m blue LED strip at room temperature for 48 h, the crude product (96:4 *E*/*Z*) was purified by flash column chromatography to afford **18** as a colorless oil in 69% yield (pure *E* isomer (NMR), 40.5 mg).

**^1^H NMR (600 MHz, CDCl_3_, *E* isomer only):** δ 7.20 (t, *J* = 8.0 Hz, 1H), 6.78 (d, *J* = 7.5 Hz, 1H), 6.77 – 6.71 (m, 2H), 6.24 (d, *J* = 1.5 Hz, 1H), 3.80 (s, 3H), 3.16 (s, 2H), 1.52 (d, *J* = 1.5 Hz, 3H), 0.96 (s, 9H), 0.16 (s, 6H).

**^13^C NMR (151 MHz, CDCl_3_, *E* isomer only):** δ 159.7, 142.5, 135.7, 129.2, 121.3, 116.7, 114.3, 111.5, 55.2, 40.4, 25.9, 18.4, 12.7, -5.1.

IR (ATR) ν 2931, 2857, 2324, 2089, 1919, 1673, 1597, 1463, 1255, 1192, 1151, 1048, 836, 778 cm^-1^;

HRMS (EI) for C_17_H_28_O_2_Si^+^ (M)^+^ : 292.18531; Found : 292.18561.

**Product 19**: the title compound was prepared following the general procedure **E** using NiBr_2_ (4.4 mg, 0.02 mmol, 10 mol%), 4,4'-di-*tert*-butyl-2,2'-bipyridine (8.1 mg, 0.15 mmol, 15 mol%), 1-(3-bromophenyl)ethan-1-one (39.8 mg, 0.2 mmol, 1 equiv.), Ir[dF(CF_3_)ppy]_2_(dtbpy)PF_6_ (2.3 mg, 0.002 mmol, 1 mol%), 2,6-lutidine (32.1 mg (⁓35 µL), 0.3 mmol, 1.5 equiv.) and *tert*-butyldimethyl((2-methylprop-1-en-1-yl)oxy)silane (112 mg, 0.6 mmol, 3 equiv.). Dissolved in the mixture solvent of benzene (1 mL) and THF (1 mL). After light irradiation with a 2 m blue LED strip at room temperature for 72 h, the crude product (95:5 *E*/*Z*) was purified by flash column chromatography to afford **19** as a colorless oil in 63% yield (pure *E* isomer, 38.4 mg).

**^1^H NMR (600 MHz, CDCl_3_, pure *E* isomer):** δ 7.83 – 7.72 (m, 2H), 7.43 – 7.32 (m, 2H), 6.25 (s, 1H), 3.22 (s, 2H), 2.59 (s, 3H), 1.49 (s, 3H), 0.94 (s, 9H), 0.15 (s, 6H).

**^13^C NMR (151 MHz, CDCl_3_, pure *E* isomer):** δ 198.5, 141.4, 137.3, 136.0, 133.6, 128.6, 128.5, 126.3, 116.3, 40.2, 26.8, 25.8, 18.4, 12.7, -5.1.

IR (ATR) ν 3468, 2930, 2857, 2091, 1725, 1682, 1598, 1466, 1434, 1358, 1260, 1192, 1153, 838, 781 cm^-1^;

HRMS (APCI) for C_18_H_29_O_2_Si^+^ (M+H)^+^ : 305.19314; Found : 305.19384.

**Product 20**: the title compound was prepared following the general procedure **E** using NiBr_2_ (4.4 mg, 0.02 mmol, 10 mol%), 4,4'-di-*tert*-butyl-2,2'-bipyridine (8.1 mg, 0.15 mmol, 15 mol%), methyl 3-bromobenzoate (43 mg, 0.2 mmol, 1 equiv.), Ir[dF(CF_3_)ppy]_2_(dtbpy)PF_6_ (2.3 mg, 0.002 mmol, 1 mol%), 2,6-lutidine (32.1 mg (⁓35 µL), 0.3 mmol, 1.5 equiv.) and *tert*-butyldimethyl((2-methylprop-1-en-1-yl)oxy)silane (112 mg, 0.6 mmol, 3 equiv.). Dissolved in the mixture solvent of benzene (1 mL) and THF (1 mL). After light irradiation with a 2 m blue LED strip at room temperature for 48 h, the crude product (95:5 *E*/*Z*) was purified by flash column chromatography to afford **20** as a colorless oil in 71% yield (pure *E* isomer, 45.6 mg).

**^1^H NMR (400 MHz, CDCl_3_, pure *E* isomer):** δ 8.01 – 7.80 (m, 2H), 7.49 – 7.31 (m, 2H), 6.25 (s, 1H), 3.90 (s, 3H), 3.21 (s, 2H), 1.49 (s, 3H), 0.94 (s, 9H), 0.15 (s, 6H).

**^13^C NMR (101 MHz, CDCl_3_, pure *E* isomer):** δ 167.2, 141.0, 135.8, 133.2, 130.0, 129.7, 128.2, 127.3, 116.2, 52.0, 40.0, 25.7, 18.3, 12.5, -5.3.

IR (ATR) ν 2932, 2857, 1723, 1674, 1438, 1280, 1194, 1153, 1105, 998, 838, 780 cm^-1^;

HRMS (APCI) for C_18_H_29_O_3_Si^+^ (M+H)^+^ : 321.18805; Found : 321.18888.

**Product 21**: the title compound was prepared following the general procedure **E** using NiBr_2_ (4.4 mg, 0.02 mmol, 10 mol%), 4,4'-di-*tert*-butyl-2,2'-bipyridine (8.1 mg, 0.15 mmol, 15 mol%), 1-bromo-3,5-bis(trifluoromethyl)benzene (58.6 mg, 0.2 mmol, 1 equiv.), Ir[dF(CF_3_)ppy]_2_(dtbpy)PF_6_ (2.3 mg, 0.002 mmol, 1 mol%), 2,6-lutidine (32.1 mg (⁓35 µL), 0.3 mmol, 1.5 equiv.) and *tert*-butyldimethyl((2-methylprop-1-en-1-yl)oxy)silane (112 mg, 0.6 mmol, 3 equiv.). Dissolved in the mixture solvent of benzene (1 mL) and THF (1 mL). After light irradiation with a 2 m blue LED strip at room temperature for 48 h, the crude product (92:8 *E*/*Z*) was purified by flash column chromatography to afford **21** as a colorless oil in 55% yield (97:3 *E*/*Z*, 44.1 mg).

**^1^H NMR (600 MHz, CDCl_3_, mixture of isomers *E*/*Z*, 97:3):** δ 7.71 (s, 1H), 7.62 (s, 2H), 6.28 (s, 1H), 3.29 (s, 2H), 1.51 (s, 3H), 0.95 (s, 9H), 0.17 (s, 6H).

**^13^C NMR (151 MHz,** **CDCl_3_, mixture of isomers *E*/*Z*, 97:3)****:** δ 143.5, 137.1, 131.6 (q, *J*_C-F_ = 32.8 Hz), 128.9 (d, *J*_C-F_ = 3.8 Hz), 123.6 (q, *J*_C-F_ = 272.5 Hz), 120.3 (hept, *J*_C-F_ = 3.7 Hz), 115.0, 40.1, 25.8, 18.5, 12.7, -5.2.

**^19^F NMR (565 MHz, CDCl_3_, mixture of isomers *E*/*Z*, 97:3):** δ -62.88.

IR (ATR) ν 2934, 2860, 1674, 1467, 1373, 1276, 1165, 1132, 896, 837, 781 cm^-1^;

HRMS (EI) for C_18_H_24_OF_6_Si^+^ (M)^+^ : 398.14952; Found : 398.14924.

**Product 22**: the title compound was prepared following the general procedure **E** using NiBr_2_ (4.4 mg, 0.02 mmol, 10 mol%), 4,4'-di-*tert*-butyl-2,2'-bipyridine (8.1 mg, 0.15 mmol, 15 mol%), 2-bromo-6-methoxynaphthalene (47.4 mg, 0.2 mmol, 1 equiv.), Ir[dF(CF_3_)ppy]_2_(dtbpy)PF_6_ (2.3 mg, 0.002 mmol, 1 mol%), 2,6-lutidine (32.1 mg (⁓35 µL), 0.3 mmol, 1.5 equiv.) and *tert*-butyldimethyl((2-methylprop-1-en-1-yl)oxy)silane (112 mg, 0.6 mmol, 3 equiv.). Dissolved in the mixture solvent of benzene (1 mL) and THF (1 mL). After light irradiation with a 2 m blue LED strip at room temperature for 72 h, the crude product (95:5 *E*/*Z*) was purified by flash column chromatography to afford **22** as a colorless oil in 61% yield (pure *E* isomer, 41.6 mg).

**^1^H NMR (600 MHz, CDCl_3_, pure *E* isomer):** δ 7.68 (t, *J* = 9.1 Hz, 2H), 7.55 (s, 1H), 7.30 (d, *J* = 8.3 Hz, 1H), 7.18 – 7.09 (m, 2H), 6.30 (s, 1H), 3.92 (s, 3H), 3.31 (s, 2H), 1.54 (s, 3H), 0.97 (s, 9H), 0.18 (s, 6H).

**^13^C NMR (151 MHz, CDCl_3_, pure *E* isomer):** δ 157.3, 135.9, 135.6, 133.3, 129.2, 129.1, 128.0, 126.8, 126.7, 118.7, 117.0, 105.8, 55.4, 40.3, 25.9, 18.5, 12.8, -5.1.

IR (ATR) ν 2934, 2858, 1674, 1604, 1472, 1384, 1231, 1191, 1150, 1026, 841, 778 cm^-1^;

HRMS (APCI) for C_21_H_31_O_2_Si^+^ (M+H)^+^ : 343.20879; Found : 343.20880.

**Prodcut 23**: the title compound was prepared following the general procedure **E** using NiBr_2_ (4.4 mg, 0.02 mmol, 10 mol%), 4,4'-di-*tert*-butyl-2,2'-bipyridine (8.1 mg, 0.15 mmol, 15 mol%), 5-bromoisobenzofuran-1(3H)-one (42.6 mg, 0.2 mmol, 1 equiv.), Ir[dF(CF_3_)ppy]_2_(dtbpy)PF_6_ (2.3 mg, 0.002 mmol, 1 mol%), 2,6-lutidine (32.1 mg (⁓35 µL), 0.3 mmol, 1.5 equiv.) and *tert*-butyldimethyl((2-methylprop-1-en-1-yl)oxy)silane (112 mg, 0.6 mmol, 3 equiv.). Dissolved in the mixture solvent of benzene (1 mL) and THF (1 mL). After light irradiation with a 2 m blue LED strip at room temperature for 48 h, the crude product (91:9 *E*/*Z*) was purified by flash column chromatography to afford **23** as a colorless oil in 69% yield (*E*/*Z* 93:7, 44.1 mg).

**^1^H NMR (600 MHz, CDCl_3_, mixture of isomers *E*/*Z*, 93:7):** ***E* isomer** (major) δ 7.80 (d, *J* = 8.1 Hz, 1H), 7.33 (d, *J* = 7.9 Hz, 1H), 7.28 (s, 1H), 6.26 (s, 1H), 5.27 (s, 2H), 3.28 (s, 2H), 1.49 (s, 3H), 0.93 (s, 9H), 0.14 (s, 6H); ***Z* isomer** (minor) 7.29 (s, 1H), 6.20 (s, 1H), 5.26 (s, 2H), 3.51 (s, 2H), 1.46 (s, 3H) the remaining resonances are insufficiently resolved from those of the ***E* isomer** to be reported.

**^13^C NMR (151 MHz, CDCl_3_, mixture of isomers *E*/*Z*, 93:7):** ***E* isomer** (major) δ 171.2, 148.3, 147.2, 136.5, 129.9, 125.6, 123.9, 122.1, 115.5, 69.6, 40.7, 25.8, 18.4, 12.7, -5.1; ***Z* isomer** (minor) 135.4, 130.0, 125.6, 17.0, -5.1, the remaining resonances are insufficiently resolved from those of the ***E* isomer** to be reported.

IR (ATR) ν 2932, 2857, 1751, 1674, 1617, 1462, 1356, 1250, 1185, 1141, 1044, 999, 836, 776 cm^-1^;

HRMS (EI) for C_18_H_26_O_3_Si^+^ (M)^+^ : 318.16457; Found : 318.16458.

**Prodcut 24**: the title compound was prepared following the general procedure **E** using NiBr_2_ (4.4 mg, 0.02 mmol, 10 mol%), 4,4'-di-*tert*-butyl-2,2'-bipyridine (8.1 mg, 0.15 mmol, 15 mol%), 5-bromo-2-methylisoindoline-1,3-dione (48 mg, 0.2 mmol, 1 equiv.), Ir[dF(CF_3_)ppy]_2_(dtbpy)PF_6_ (2.3 mg, 0.002 mmol, 1 mol%), 2,6-lutidine (32.1 mg (⁓35 µL), 0.3 mmol, 1.5 equiv.) and *tert*-butyldimethyl((2-methylprop-1-en-1-yl)oxy)silane (112 mg, 0.6 mmol, 3 equiv.). Dissolved in the mixture solvent of benzene (1 mL) and THF (1 mL). After light irradiation with a 2 m blue LED strip at room temperature for 48 h, the crude product (92:8 *E*/*Z*) was purified by flash column chromatography to afford **24** as a colorless oil in 73% yield (*E*/*Z* 93:7, 50.5 mg).

**^1^H NMR (600 MHz, CDCl_3_, mixture of isomers *E*/*Z*, 93:7):** ***E* isomer** (major) δ 7.72 (d, *J* = 7.6 Hz, 1H), 7.64 (s, 1H), 7.49 (d, *J* = 7.7 Hz, 1H), 6.27 (d, *J* = 1.3 Hz, 1H), 3.28 (s, 2H), 3.15 (s, 3H), 1.47 (d, *J* = 1.5 Hz, 3H), 0.93 (s, 9H), 0.14 (s, 6H); ***Z* isomer** (minor) 6.21 (d, *J* = 1.5 Hz, 1H), 3.51 (s, 2H), 1.45 (d, *J* = 1.5 Hz, 3H) the remaining resonances are insufficiently resolved from those of the ***E* isomer** to be reported.

**^13^C NMR (151 MHz, CDCl_3_, mixture of isomers *E*/*Z*, 93:7):** ***E* isomer** (major) 168.8, 168.6, 148.3, 136.7, 134.2, 132.7, 130.3, 123.5, 123.2, 115.2, 40.7, 25.8, 24.0, 18.4, 12.6, -5.1; ***Z* isomer** (minor) 148.7, 135.6, 130.0, 123.6, 123.1, 114.1, 35.6, 24.0, 18.3, 17.0, -5.1 the remaining resonances are insufficiently resolved from those of the ***E* isomer** to be reported.

IR (ATR) ν 2932, 2858, 1769, 1709, 1620, 1433, 1379, 1252, 1190, 1154, 1007, 837, 781 cm^-1^;

HRMS (APCI) for C_19_H_28_O_3_NSi^+^ (M+H)^+^ : 346.18330; Found : 343.18388.

**Prodcut 25**: the title compound was prepared following the general procedure **E** using NiBr_2_ (4.4 mg, 0.02 mmol, 10 mol%), 4,4'-di-*tert*-butyl-2,2'-bipyridine (8.1 mg, 0.03 mmol, 15 mol%), *tert*-butyl 5-bromo-1*H*-indole-1-carboxylate (59.2 mg, 0.2 mmol, 1 equiv.), Ir[dF(CF_3_)ppy]_2_(dtbpy)PF_6_ (2.3 mg, 0.002 mmol, 1 mol%), 2,6-lutidine (32.1 mg (⁓35 µL), 0.3 mmol, 1.5 equiv.) and *tert*-butyldimethyl((2-methylprop-1-en-1-yl)oxy)silane (112 mg, 0.6 mmol, 3 equiv.). Dissolved in the mixture solvent of benzene (1 mL) and THF (1 mL). After light irradiation with a 2 m blue LED strip at room temperature for 48 h, the crude product (96:4 *E*/*Z*) was purified by flash column chromatography to afford **25** as a colorless oil in 59% yield (*E*/*Z* 96:4, 47.4 mg).

**^1^H NMR (600 MHz, CDCl_3_, mixture of isomers *E*/*Z*, 96:4):** ***E* isomer** (major) δ 8.04 (s, 1H), 7.57 (s, 1H), 7.36 (s, 1H), 7.14 (dd, *J* = 8.5, 1.7 Hz, 1H), 6.51 (d, *J* = 3.8 Hz, 1H), 6.28 (d, *J* = 1.0 Hz, 1H), 3.27 (s, 2H), 1.68 (s, 9H), 1.52 (d, *J* = 1.4 Hz, 3H), 0.96 (s, 9H), 0.17 (s, 6H); ***Z* isomer** (minor) 7.38 (s, 1H), 7.18 (dd, *J* = 8.6, 1.8 Hz, 1H), 6.28 (d, *J* = 1.6 Hz, 1H), 3.51 (s, 2H), 1.46 (d, *J* = 1.5 Hz, 3H) the remaining resonances are insufficiently resolved from those of the ***E* isomer** to be reported.

**^13^C NMR (151 MHz, CDCl_3_, mixture of isomers *E*/*Z*, 96:4): *E* isomer** (major) δ 150.0, 135.4, 135.1, 133.9, 130.8, 126.0, 125.3, 120.8, 117.4, 114.9, 107.3, 83.6, 40.2, 28.4, 25.9, 18.4, 12.7, -5.1.

IR (ATR) ν 2931, 2857, 1733, 1673, 1467, 1373, 1345, 1254, 1154, 1081, 1022, 838, 773, 724 cm^-1^;

HRMS (ESI) for C_23_H_35_O_3_NNaSi^+^ (M+Na)^+^ : 424.22784; Found : 424.22766.

**Product 26**: the title compound was prepared following the general procedure using NiBr_2_ (4.4 mg, 0.02 mmol, 10 mol%), 4,4'-di-*tert*-butyl-2,2'-bipyridine (8.1 mg, 0.03 mmol, 15 mol%), 4-bromo-1-tosyl-1*H*-pyrrolo[2,3-*b*]pyridine (70.2 mg, 0.2 mmol, 1 equiv.), Ir[dF(CF_3_)ppy]_2_(dtbpy)PF_6_ (2.3 mg, 0.002 mmol, 1 mol%), 2,6-lutidine (32.1 mg (⁓35 µL), 0.3 mmol, 1.5 equiv.) and *tert*-butyldimethyl((2-methylprop-1-en-1-yl)oxy)silane (112 mg, 0.6 mmol, 3 equiv.). Dissolved in the mixture solvent of benzene (1 mL) and THF (1 mL). After light irradiation with a 2 m blue LED strip at room temperature for 48 h, the crude product (97:3 *E*/*Z*) was purified by flash column chromatography to afford **26** as a white solid in 62% yield (95:5 *E*/*Z*, 56.6 mg).

**^1^H NMR (600 MHz, CDCl_3_,** **mixture of isomers *E*/*Z*, 95:5):** δ 8.33 (d, *J* = 5.0 Hz, 1H), 8.08 (d, *J* = 8.2 Hz, 2H), 7.69 (d, *J* = 4.0 Hz, 1H), 7.26 (d, *J* = 8.0 Hz, 2H), 6.98 (d, *J* = 4.9 Hz, 1H), 6.66 (d, *J* = 4.1 Hz, 1H), 6.24 (s, 1H), 3.37 (s, 2H), 2.36 (s, 3H), 1.48 (s, 3H), 0.92 (s, 9H), 0.13 (s, 6H).

**^13^C NMR (151 MHz, CDCl_3_, mixture of isomers *E*/*Z*, 95:5):** δ 147.4, 145.1, 145.0, 143.5, 136.6, 135.7, 129.7, 128.1, 125.7, 122.8, 119.0, 114.6, 103.8, 37.1, 25.8, 21.7, 12.9, -5.1.

IR (ATR) ν 2934, 2859, 1674, 1590, 1464, 1368, 1254, 1158, 1081, 1023, 839, 783, 727 cm^-1^;

HRMS (ESI) for C_24_H_33_O_3_N_2_SSi^+^ (M+H)^+^ : 457.19757; Found : 457.19760.

**Product 27**: the title compound was prepared following the general procedure using NiBr_2_ (4.4 mg, 0.02 mmol, 10 mol%), 4,4'-di-*tert*-butyl-2,2'-bipyridine (8.1 mg, 0.15 mmol, 15 mol%), 6-bromo-2-methylquinoline (44.4 mg, 0.2 mmol, 1 equiv.), Ir[dF(CF_3_)ppy]_2_(dtbpy)PF_6_ (2.3 mg, 0.002 mmol, 1 mol%), 2,6-lutidine (32.1 mg (⁓35 µL), 0.3 mmol, 1.5 equiv.) and *tert*-butyldimethyl((2-methylprop-1-en-1-yl)oxy)silane (112 mg, 0.6 mmol, 3 equiv.). Dissolved in the mixture solvent of benzene (1 mL) and THF (1 mL). After light irradiation with a 2 m blue LED strip at room temperature for 48 h, the crude product (92:8 *E*/*Z*) was purified by flash column chromatography to afford **27** as a white solid in 51% yield (95:5 *E*/*Z*, 33.5 mg).

**^1^H NMR (600 MHz, CDCl_3_, mixture of isomers *E*/*Z*, 95:5):** ***E* isomer** (major) δ 7.96 (d, *J* = 8.4 Hz, 1H), 7.92 (d, *J* = 8.4 Hz, 1H), 7.57 – 7.47 (m, 2H), 7.24 (d, *J* = 8.4 Hz, 1H), 6.28 (s, 1H), 3.33 (s, 2H), 2.72 (s, 3H), 1.52 (s, 3H), 0.95 (s, 9H), 0.15 (s, 6H).

**^13^C NMR (151 MHz, CDCl_3_, mixture of isomers *E*/*Z*, 95:5):** ***E* isomer** (major) δ 158.3, 147.0, 138.2, 136.0, 135.9, 131.0, 128.5, 126.5, 126.4, 122.0, 116.5, 40.3, 25.8, 25.4, 18.4, 12.8, -5.1.

IR (ATR) ν 2953, 2856, 1671, 1601, 1467, 1389, 1253, 1219, 1190, 1147, 834, 777 cm^-1^;

HRMS (ESI) for C_20_H_30_ONaSi^+^ (M+Na)^+^ : 328.20912; Found : 328.20874.

**Prodcut 28**: the title compound was prepared following the general procedure using NiBr_2_ (4.4 mg, 0.02 mmol, 10 mol%), 4,4'-di-*tert*-butyl-2,2'-bipyridine (8.1 mg, 0.15 mmol, 15 mol%), 5-bromo-2-(trifluoromethyl)pyridine (45.2 mg, 0.2 mmol, 1 equiv.), Ir[dF(CF_3_)ppy]_2_(dtbpy)PF_6_ (2.3 mg, 0.002 mmol, 1 mol%), 2,6-lutidine (32.1 mg (⁓35 µL), 0.3 mmol, 1.5 equiv.) and *tert*-butyldimethyl((2-methylprop-1-en-1-yl)oxy)silane (112 mg, 0.6 mmol, 3 equiv.). Dissolved in the mixture solvent of benzene (1 mL) and THF (1 mL). After light irradiation with a 2 m blue LED strip at room temperature for 48 h, the crude product (86:14 *E*/*Z*) was purified by flash column chromatography to afford **28** as a colorless oil in 71% yield (*E*/Z 88:12, 46.8 mg).

**^1^H NMR (600 MHz, CDCl_3_, mixture of isomers *E*/*Z*, 88:12):** ***E* isomer** (major) δ 8.58 – 8.51 (m, 1H), 7.65 (dd, *J* = 7.9, 2.0 Hz, 1H), 7.59 (d, *J* = 8.1 Hz, 1H), 6.27 (s, 1H), 3.24 (s, 2H), 1.50 (s, 3H), 0.93 (s, 9H), 0.14 (s, 6H); ***Z* isomer** (minor) 6.21 (s, 1H), 3.45 (s, 2H), 1.46 (s, 3H), the remaining resonances are insufficiently resolved from those of the ***E* isomer** to be reported.

**^13^C NMR (151 MHz, CDCl_3_, mixture of isomers *E*/*Z*, 88:12):** ***E* isomer** (major) δ 150.5, 146.3 (q, *J*_C-F_ = 34.0 Hz), 139.7, 137.3, 136.8, 121.9 (q, *J*_C-F_ = 273.8 Hz), 120.2 (q, *J*_C-F_ = 3.4 Hz), 114.7, 32.4, 25.8, 18.4, 12.6, -5.2; ***Z* isomer** (minor) 150.5, 140.1, 135.7, 113.6, 37.4, 18.3, 16.9, -5.1, the remaining resonances are insufficiently resolved from those of the ***E* isomer** to be reported.

**^19^F NMR (565 MHz, CDCl_3_):** ***E* isomer** (major) δ -67.7.

IR (ATR) ν 2932, 2859, 1673, 1467, 1397, 1335, 1255, 1140, 1085, 1027, 838, 780 cm^-1^;

HRMS (EI) for C_16_H_24_ONF_3_Si^+^ (M)^+^ : 331.15738; Found : 331.15749.

**Product 29**: the title compound was prepared following the general procedure using NiBr_2_ (4.4 mg, 0.02 mmol, 10 mol%), 4,4'-di-*tert*-butyl-2,2'-bipyridine (8.1 mg, 0.03 mmol, 15 mol%), methyl 2-(1-(4-bromobenzoyl)-5-methoxy-2-methyl-1H-indol-3-yl)acetate (83.3 mg, 0.2 mmol, 1 equiv.), Ir[dF(CF_3_)ppy]_2_(dtbpy)PF_6_ (2.3 mg, 0.002 mmol, 1 mol%), 2,6-lutidine (32.1 mg (⁓35 µL), 0.3 mmol, 1.5 equiv.) and *tert*-butyldimethyl((2-methylprop-1-en-1-yl)oxy)silane (112 mg, 0.6 mmol, 3 equiv.). Dissolved in the mixture solvent of benzene (1 mL) and THF (1 mL). After light irradiation with a 2 m blue LED strip at room temperature for 48 h, the crude product (94:6 *E*/*Z*) was purified by flash column chromatography to afford **29** as a light yellow oil in 72% yield (94:6 *E*/*Z*, 75.1 mg).

**^1^H NMR (400 MHz, CDCl_3_, mixture of isomers *E*/*Z*, 94:6):** ***E* isomer** (major) δ 7.64 (d, *J* = 7.8 Hz, 2H), 7.29 (d, *J* = 7.8 Hz, 2H), 6.96 (d, *J* = 2.6 Hz, 1H), 6.89 (d, *J* = 9.0 Hz, 1H), 6.65 (dd, *J* = 8.9, 2.4 Hz, 1H), 6.26 (s, 1H), 3.84 (s, 3H), 3.70 (s, 3H), 3.68 (s, 2H), 3.27 (s, 2H), 2.38 (s, 3H), 1.53 (s, 3H), 0.95 (s, 9H), 0.16 (s, 6H).

**^13^C NMR (101 MHz, CDCl_3_, mixture of isomers *E*/*Z*, 94:6):** ***E* isomer** (major) δ 171.6, 169.6, 155.9, 146.5, 136.2 (2C), 133.4, 131.1, 130.6, 130.0, 129.1, 115.9, 115.1, 112.0, 111.5, 101.2, 55.8, 52.2, 40.4, 30.3, 25.8, 18.4, 13.3, 12.7, -5.1.

IR (ATR) ν 2932, 2857, 1739, 1678, 1607, 1473, 1357, 1314, 1256, 1222, 1149, 1067, 1034, 914, 837, 782, 732 cm^-1^;

HRMS (ESI) for C_30_H_39_O_5_NNaSi^+^ (M+Na)^+^ : 544.24897; Found : 544.24829.

**Product 30**: the title compound was prepared following the general procedure using NiBr_2_ (4.4 mg, 0.02 mmol, 10 mol%), 4,4'-di-*tert*-butyl-2,2'-bipyridine (8.1 mg, 0.03 mmol, 15 mol%), 4-(5-(4-bromophenyl)-3-(trifluoromethyl)-1*H*-pyrazol-1-yl)benzenesulfonamide (89.3 mg, 0.2 mmol, 1 equiv.), Ir[dF(CF_3_)ppy]_2_(dtbpy)PF_6_ (2.3 mg, 0.002 mmol, 1 mol%), 2,6-lutidine (32.1 mg (⁓35 µL), 0.3 mmol, 1.5 equiv.) and *tert*-butyldimethyl((2-methylprop-1-en-1-yl)oxy)silane (112 mg, 0.6 mmol, 3 equiv.). Dissolved in the mixture solvent of benzene (1 mL) and THF (1 mL). After light irradiation with a 2 m blue LED strip at room temperature for 48 h, the crude product (94:6 *E*/*Z*) was purified by flash column chromatography to afford **30** as a colorless oil in 68% yield (94:6 *E*/*Z*, 75 mg).

**^1^H NMR (600 MHz, CDCl_3_, mixture of isomers *E*/*Z*, 94:6):** ***E* isomer** (major) δ 7.88 (d, *J* = 8.3 Hz, 2H), 7.45 (d, *J* = 8.2 Hz, 2H), 7.18 (d, *J* = 7.8 Hz, 2H), 7.13 (d, *J* = 7.8 Hz, 2H), 6.74 (s, 1H), 6.23 (s, 1H), 5.23 (s, 2H), 3.18 (s, 2H), 1.49 (s, 3H), 0.93 (s, 9H), 0.14 (s, 6H); ***Z* isomer** (minor) 6.19 (s, 1H), 3.42 (s, 2H), 1.45 (s, 3H), the remaining resonances are insufficiently resolved from those of the ***E* isomer** to be reported.

**^13^C NMR (151 MHz, CDCl_3_, mixture of isomers *E*/*Z*, 94:6):** ***E* isomer** (major) δ 145.4, 144.2 (q, *J*_C-F_ = 39.2 Hz), 142.8, 142.6, 141.5, 136.1, 129.5, 128.8, 127.6, 126.3, 125.6, 121.1 (q, *J*_C-F_ = 269.2 Hz), 115.9, 106.5, 40.1, 25.8, 18.4, 12.7, -5.1; ***Z* isomer** (minor) 35.0, 18.3, 17.1, -5.1, the remaining resonances are insufficiently resolved from those of the ***E* isomer** to be reported.

**^19^F NMR (565 MHz, CDCl_3_):** δ -62.41.

IR (ATR) ν 3268, 2937, 2861, 1673, 1468, 1341, 1238, 1148, 975, 910, 839, 782, 731 cm^-1^;

HRMS (ESI) for C_26_H_32_O_3_N_3_F_3_NaSSi^+^ (M+Na)^+^ : 574.17779; Found : 574.17712.

**Product 31**: the title compound was prepared following the general procedure using NiBr_2_ (4.4 mg, 0.02 mmol, 10 mol%), 4,4'-di-*tert*-butyl-2,2'-bipyridine (8.1 mg, 0.15 mmol, 15 mol%), ((3a*R*,5*R*,5a*S*,8a*S*,8b*R*)-2,2,7,7-tetramethyltetrahydro-5*H*-bis([1,3]dioxolo)[4,5-*b*:4',5'-*d*]pyran-5-yl)methyl 4-bromobenzoate (88.7 mg, 0.2 mmol, 1 equiv.), Ir[dF(CF_3_)ppy]_2_(dtbpy)PF_6_ (2.3 mg, 0.002 mmol, 1 mol%), 2,6-lutidine (32.1 mg (⁓35 µL), 0.3 mmol, 1.5 equiv.) and *tert*-butyldimethyl((2-methylprop-1-en-1-yl)oxy)silane (112 mg, 0.6 mmol, 3 equiv.). Dissolved in the mixture solvent of benzene (1 mL) and THF (1 mL). After light irradiation with a 2 m blue LED strip at room temperature for 48 h, the crude product (94:6 *E*/*Z*) was purified by flash column chromatography to afford **31** as a colorless oil in 71% yield (94:6 *E*/*Z*, 77.9 mg).

**^1^H NMR (600 MHz, CDCl_3_, mixture of isomers *E*/*Z*, 94:6):** ***E* isomer** (major) δ 7.95 (d, *J* = 8.2 Hz, 2H), 7.22 (d, *J* = 8.0 Hz, 2H), 6.22 (s, 1H), 5.56 (d, *J* = 4.9 Hz, 1H), 4.64 (dd, *J* = 7.8, 2.5 Hz, 1H), 4.51 (dd, *J* = 11.5, 4.9 Hz, 1H), 4.41 (dd, *J* = 11.4, 7.5 Hz, 1H), 4.35 – 4.31 (m, 2H), 4.21 – 4.15 (m, 1H), 3.21 (s, 2H), 1.51 (s, 3H), 1.48 (d, *J* = 1.5 Hz, 3H), 1.47 (s, 3H), 1.35 (s, 3H), 1.33 (s, 3H), 0.93 (s, 9H), 0.14 (s, 6H); ***Z* isomer** (minor) 7.25 (d, *J* = 8.3 Hz, 2H), 6.18 (s, 1H), 3.44 (s, 2H), the remaining resonances are insufficiently resolved from those of the ***E* isomer** to be reported.

**^13^C NMR (151 MHz, CDCl_3_, mixture of isomers *E*/*Z*, 94:6):** ***E* isomer** (major) δ 166.5, 146.4, 136.0, 129.8, 128.7, 128.0, 116.0, 109.7, 108.9, 96.4, 71.2, 70.8, 70.6, 66.3, 63.8, 40.4, 26.1 (2C), 25.8, 25.1, 24.6, 18.4, 12.7, -5.2; ***Z* isomer** (minor) 166.6, 146.9, 134.9, 128.8, 127.6, 114.9, 63.7, 35.2, 18.2, 16.9, -5.1, the remaining resonances are insufficiently resolved from those of the ***E* isomer** to be reported.

IR (ATR) ν 2932, 2859, 1719, 1674, 1610, 1463, 1378, 1257, 1208, 1164, 1103, 1069, 1005, 896, 838, 781 cm^-1^;

HRMS (EI) for C_29_H_44_O_8_Si^+^ (M)^+^ : 548.28000; Found : 548.28041.

**Product 32**: the title compound was prepared following the general procedure using NiBr_2_ (4.4 mg, 0.02 mmol, 10 mol%), 4,4'-di-*tert*-butyl-2,2'-bipyridine (8.1 mg, 0.15 mmol, 15 mol%), (3*S*,8*S*,9*S*,10*R*,13*S*,14*S*,17*S*)-17-acetyl-10,13-dimethyl-2,3,4,7,8,9,10,11,12,13,14,15,16,17-tetradecahydro-1*H*-cyclopenta[*a*]phenanthren-3-yl 4-bromobenzoate (99.9 mg, 0.2 mmol, 1 equiv.), Ir[dF(CF_3_)ppy]_2_(dtbpy)PF_6_ (2.3 mg, 0.002 mmol, 1 mol%), 2,6-lutidine (32.1 mg (⁓35 µL), 0.3 mmol, 1.5 equiv.) and *tert*-butyldimethyl((2-methylprop-1-en-1-yl)oxy)silane (112 mg, 0.6 mmol, 3 equiv.). Dissolved in the mixture solvent of benzene (1 mL) and THF (1 mL). After light irradiation with a 2 m blue LED strip at room temperature for 48 h, the crude product (94:6 *E*/*Z*) was purified by flash column chromatography to afford **32** as a colorless oil in 52% yield (*E*/Z 93:7, 62.3 mg).

**^1^H NMR (400 MHz, CDCl_3_, mixture of isomers *E*/*Z*, 93:7):** ***E* isomer** (major) δ 7.92 (d, *J* = 7.8 Hz, 2H), 7.20 (d, *J* = 8.1 Hz, 2H), 6.20 (s, 1H), 5.39 (s, 1H), 4.92 – 4.71 (m, 1H), 3.18 (s, 2H), 2.58 – 2.38 (m, 3H), 2.20 – 1.87 (m, 8H), 1.74 – 1.41 (m, 11H), 1.32 – 1.09 (m, 4H), 1.04 (s, 3H), 0.91 (s, 9H), 0.61 (s, 3H), 0.11 (s, 6H).

**^13^C NMR (101 MHz, CDCl_3_, mixture of isomers *E*/*Z*, 93:7):** ***E* isomer** (major) δ 209.6, 166.2, 146.2, 139.8, 136.0, 129.6, 128.8, 128.7, 128.7, 122.5, 116.1, 74.3, 63.8, 57.0, 50.0, 44.1, 40.4, 38.9, 38.3, 37.2, 36.8, 32.0, 31.9, 31.7, 28.0, 25.8, 24.6, 22.9, 21.2, 19.5, 18.4, 13.4, 12.7 -5.1.

IR (ATR) ν 2946, 2884, 1704, 1673, 1466, 1356, 1272, 1188, 1146, 1099, 1008, 840, 782 cm^-1^;

HRMS (APCI) for C_38_H_57_O_4_Si^+^ (M+H)^+^ : 605.40206; Found : 605.40266.

**Product 33**: the title compound was prepared following the general procedure using NiBr_2_ (4.4 mg, 0.02 mmol, 10 mol%), 4,4'-di-*tert*-butyl-2,2'-bipyridine (8.1 mg, 0.15 mmol, 15 mol%), 4-bromo-*N*-(2,6-dimethoxypyrimidin-4-yl)-*N*-methylbenzenesulfonamide (77.6 mg, 0.2 mmol, 1 equiv.), Ir[dF(CF_3_)ppy]_2_(dtbpy)PF_6_ (2.3 mg, 0.002 mmol, 1 mol%), 2,6-lutidine (32.1 mg (⁓35 µL), 0.3 mmol, 1.5 equiv.) and *tert*-butyldimethyl((2-methylprop-1-en-1-yl)oxy)silane (112 mg, 0.6 mmol, 3 equiv.). Dissolved in the mixture solvent of benzene (1 mL) and THF (1 mL). After light irradiation with a 2 m blue LED strip at room temperature for 48 h, the crude product (95:5 *E*/*Z*) was purified by flash column chromatography to afford **33** as a colorless oil in 62% yield (pure *E* isomer, 60.8 mg).

**^1^H NMR (600 MHz, CDCl_3_, *E* isomer only):** δ 7.68 (d, *J* = 8.1 Hz, 2H), 7.26 (d, *J* = 8.1 Hz, 2H), 6.63 (s, 1H), 6.20 (s, 1H), 3.91 (s, 3H), 3.83 (s, 3H), 3.43 (s, 3H), 3.19 (s, 2H), 1.44 (s, 3H), 0.91 (s, 9H), 0.12 (s, 6H). **^13^C NMR (151 MHz, CDCl_3_, *E* isomer only):** δ 172.6, 164.3, 161.5, 147.2, 136.5, 136.2, 129.4, 127.2, 115.3, 89.9, 54.8, 54.2, 40.2, 34.5, 25.8, 18.4, 12.7, -5.2. IR (ATR) ν 2932, 2859, 1674, 1577, 1468, 1357, 1191, 1151, 1023, 973, 920, 835, 779 cm^-1^; HRMS (EI) for C_23_H_35_O_5_N_3_SSi^+^ (M)^+^ : 493.20612; Found : 493.20653.

**Product 34**: the title compound was prepared following the general procedure using NiBr_2_ (4.4 mg, 0.02 mmol, 10 mol%), 4,4'-di-*tert*-butyl-2,2'-bipyridine (8.1 mg, 0.03 mmol, 15 mol%), methyl 4-bromobenzoate (43 mg, 0.2 mmol, 1 equiv.), Ir[dF(CF_3_)ppy]_2_(dtbpy)PF_6_ (2.3 mg, 0.002 mmol, 1 mol%), 2,6-lutidine (32.1 mg (⁓35 µL), 0.3 mmol, 1.5 equiv.), *tert*-butyl acrylate (51.3 mg, 0.4 mmol, 1.5 equiv.) and triisopropyl((2-methylprop-1-en-1-yl)oxy)silane (137 mg, 0.6 mmol, 3 equiv.). Dissolved in the mixture solvent of benzene (1 mL) and THF (1 mL). After light irradiation with a 2 m blue LED strip at room temperature for 43 h, the crude product (96:4 *E*/*Z*) was purified by flash column chromatography to afford **34** as a colorless oil in 73% yield (96:4 *E*/*Z*, 52.8mg).

**^1^H NMR (400 MHz, CDCl_3_, mixture of isomers *E*/*Z*, 96:4):** ***E* isomer** (major) δ 7.93 (d, *J* = 8.0 Hz, 2H), 7.22 (d, *J* = 8.0 Hz, 2H), 6.30 (s, 1H), 3.88 (s, 3H), 3.19 (s, 2H), 1.49 (s, 3H), 1.18 – 1.10 (m, 3H), 1.08 (s, 9H), 1.06 (s, 9H).

**^13^C NMR (101 MHz, CDCl_3_, mixture of isomers *E*/*Z*, 96:4):** ***E* isomer** (major) δ 167.1, 146.4, 136.4, 129.5, 128.6, 127.9, 115.0, 51.9, 40.2, 17.7, 12.5, 11.9.

IR (ATR) ν 2949, 1721, 1609, 1437, 1277, 1151, 1108, 1020, 756 cm^-1^;

HRMS (APCI) for C_21_H_35_O_3_Si^+^ (M+H)^+^ : 363.23500; Found : 363.23613.

**Product 35**: the title compound was prepared following the general procedure using NiBr_2_ (4.4 mg, 0.02 mmol, 10 mol%), 4,4'-di-*tert*-butyl-2,2'-bipyridine (8.1 mg, 0.03 mmol, 15 mol%), methyl 4-bromobenzoate (43 mg, 0.2 mmol, 1 equiv.), Ir[dF(CF_3_)ppy]_2_(dtbpy)PF_6_ (2.3 mg, 0.002 mmol, 1 mol%), 2,6-lutidine (32.1 mg (⁓35 µL), 0.3 mmol, 1.5 equiv.), *tert*-Butyl acrylate (51.3 mg, 0.4 mmol, 1.5 equiv.) and ((2-methylprop-1-en-1-yl)oxy)cyclopentane (97.3 mg, 0.6 mmol, 3 equiv.). Dissolved in the mixture solvent of benzene (1 mL) and THF (1 mL). After light irradiation with a 2 m blue LED strip at room temperature for 45 h, the crude product (94:6 *E*/*Z*) was purified by flash column chromatography to afford **35** as a colorless oil in 65% yield (97:3 *E*/*Z*, 35.9 mg).

**^1^H NMR (400 MHz, CDCl_3_, mixture of isomers *E*/*Z*, 97:3):** ***E* isomer** (major) δ 7.93 (d, *J* = 8.2 Hz, 2H), 7.23 (d, *J* = 8.2 Hz, 2H), 6.00 – 5.89 (m, 1H), 4.22 (tt, *J* = 5.2, 2.7 Hz, 1H), 3.88 (s, 3H), 3.20 (s, 2H), 1.80 – 1.65 (m, 6H), 1.61 – 1.49 (m, 2H), 1.46 (d, *J* = 1.3 Hz, 3H); ***Z* isomer** (minor) 3.39 (s, 2H), the remaining resonances are insufficiently resolved from those of the ***E* isomer** to be reported.

**^13^C NMR (101 MHz, CDCl_3_, mixture of isomers *E*/*Z*, 97:3):** ***E* isomer** (major) δ 167.3, 146.3, 141.2, 129.7, 128.8, 128.1, 113.5, 83.1, 52.1, 40.7, 32.8, 23.7, 13.1; the resonances of ***Z* isomer** are insufficiently resolved from those of the ***E* isomer** to be reported.

IR (ATR) ν 2953, 1720, 1680, 1609, 1435, 1276, 1177, 1148, 1107, 979, 836, 756 cm^-1^;

HRMS (ESI) for C_17_H_23_O_3_^+^ (M+H)^+^ : 275.16417; Found : 275.16528.

**Product 36**: the title compound was prepared following the general procedure using NiBr_2_ (4.4 mg, 0.02 mmol, 10 mol%), 4,4'-di-*tert*-butyl-2,2'-bipyridine (8.1 mg, 0.15 mmol, 15 mol%), methyl 4-bromobenzoate (43 mg, 0.2 mmol, 1 equiv.), Ir[dF(CF_3_)ppy]_2_(dtbpy)PF_6_ (2.3 mg, 0.002 mmol, 1 mol%), 2,6-lutidine (32.1 mg (⁓35 µL), 0.3 mmol, 1.5 equiv.), tert-Butyl acrylate (51.3 mg, 0.4 mmol, 1.5 equiv.) and ((2-methylprop-1-en-1-yl)oxy)benzene (88.9 mg, 0.6 mmol, 3 equiv.). Dissolved in the mixture solvent of benzene (1 mL) and THF (1 mL). After light irradiation with a 2 m blue LED strip at room temperature for 60 h, the crude product (90:10 *E*/*Z*) was purified by flash column chromatography to afford **36** as a colorless oil in 62% yield (93:7 *E*/*Z*, 35.1 mg).

**^1^H NMR (400 MHz, CDCl_3_, mixture of isomers *E*/*Z*, 93:7):** ***E* isomer** (major) δ 7.98 (d, *J* = 8.0 Hz, 2H), 7.36 – 7.25 (m, 4H), 7.08 – 6.93 (m, 3H), 6.36 (s, 1H), 3.90 (s, 3H), 3.36 (s, 2H), 1.64 (s, 3H).

**^13^C NMR (101 MHz, CDCl_3_, mixture of isomers *E*/*Z*, 93:7):** ***E* isomer** (major) δ 167.2, 157.6, 145.2, 137.6, 129.9, 129.7, 128.9, 128.4, 122.4, 119.7, 116.1, 52.2, 40.4, 13.3.

IR (ATR) ν 2947, 2914, 2328, 2082, 1931, 1719, 1594, 1489, 1435, 1277, 1231, 1106, 1018, 819, 754 cm^-1^;

HRMS (ESI) for C_18_H_18_O_3_Na^+^ (M+Na)^+^: 305.11482; Found : 305.11460.

**Product 37**: the title compound was prepared following the general procedure using NiBr_2_ (4.4 mg, 0.02 mmol, 10 mol%), 4,4'-di-*tert*-butyl-2,2'-bipyridine (8.1 mg, 0.03 mmol, 15 mol%), methyl 4-bromobenzoate (43 mg, 0.2 mmol, 1 equiv.), Ir[dF(CF_3_)ppy]_2_(dtbpy)PF_6_ (2.3 mg, 0.002 mmol, 1 mol%), 2,6-lutidine (32.1 mg (⁓35 µL), 0.3 mmol, 1.5 equiv.), *tert*-Butyl acrylate (51.3 mg, 0.4 mmol, 1.5 equiv.) and (1*S*,2*R*,4*S*)-1,7,7-trimethyl-2-((2-methylprop-1-en-1-yl)oxy)bicyclo[2.2.1]heptane (125 mg, 0.6 mmol, 3 equiv.). Dissolved in the mixture solvent of benzene (1 mL) and THF (1 mL). After light irradiation with a 2 m blue LED strip at room temperature for 43 h, the crude product (93:7 *E*/*Z*) was purified by flash column chromatography to afford **37** as a colorless oil in 71% yield (98:2 *E*/*Z*, 48.4mg).

**^1^H NMR (400 MHz, CDCl_3_, mixture of isomers *E*/*Z*, 98:2):** ***E* isomer** (major) δ 7.93 (d, *J* = 7.9 Hz, 2H), 7.23 (d, *J* = 7.9 Hz, 2H), 5.94 (s, 1H), 3.98 – 3.81 (m, 4H), 3.19 (s, 2H), 2.22 – 2.12 (m, 1H), 2.06 – 1.96 (m, 1H), 1.74 – 1.60 (m, 2H), 1.52 (s, 3H), 1.28 – 1.20 (m, 2H), 1.09 (dd, *J* = 13.3, 3.2 Hz, 1H), 0.89 – 0.82 (m, 9H).

**^13^C NMR (101 MHz, CDCl_3_, mixture of isomers *E*/*Z*, 98:2):** ***E* isomer** (major) δ 167.3, 146.4, 142.8, 129.7, 128.9, 128.1, 112.1, 86.8, 52.1, 49.6, 48.2, 45.1, 40.6, 36.5, 28.3, 26.9, 19.9, 19.0, 14.0, 13.3.

IR (ATR) ν 2954, 1720, 1680, 1609, 1435, 1276, 1177, 1147, 1106, 979, 836, 756cm^-1^;

HRMS (ESI) for C_22_H_31_O_3_^+^ (M+H)^+^ : 343.22677; Found : 343.22763.

**Product 38**: the title compound was prepared following the general procedure using NiBr_2_ (4.4 mg, 0.02 mmol, 10 mol%), 4,4'-di-*tert*-butyl-2,2'-bipyridine (8.1 mg, 0.15 mmol, 15 mol%), methyl 4-bromobenzoate (43 mg, 0.2 mmol, 1 equiv.), Ir[dF(CF_3_)ppy]_2_(dtbpy)PF_6_ (2.3 mg, 0.002 mmol, 1 mol%), 2,6-lutidine (32.1 mg (⁓35 µL), 0.3 mmol, 1.5 equiv.), tert-Butyl acrylate (51.3 mg, 0.4 mmol, 1.5 equiv.) and 1-ethoxy-2-methylprop-1-ene (60.1 mg, 0.6 mmol, 3 equiv.). Dissolved in the mixture solvent of benzene (1 mL) and THF (1 mL). After light irradiation with a 2 m blue LED strip at room temperature for 60 h, the crude product (92:8 *E*/*Z*) was purified by flash column chromatography to afford **38** as a colorless oil in 61% yield (only *E* isomer, 28.5 mg).

**^1^H NMR (400 MHz, CDCl_3_, only *E* isomer):** δ 7.93 (d, *J* = 7.9 Hz, 2H), 7.23 (d, *J* = 8.0 Hz, 2H), 5.94 (s, 1H), 3.88 (s, 3H), 3.77 (q, *J* = 7.1 Hz, 2H), 3.20 (s, 2H), 1.49 (s, 3H), 1.24 (t, *J* = 7.0 Hz, 3H).

**^13^C NMR (101 MHz, CDCl_3_, only *E* isomer):** δ 167.3, 146.2, 142.3, 129.7, 128.8, 128.1, 112.7, 67.5, 52.1, 40.6, 15.4, 13.0.

IR (ATR) ν 2977, 2907, 2326, 2080, 1928, 1720, 1609, 1435, 1277, 1182, 1109, 1020, 757 cm^-1^;

HRMS (ESI) for C_14_H_18_O_3_Na^+^ (M+Na)^+^ : 257.11482; Found : 257.11413.

**Product 39**: the title compound was prepared following the general procedure using NiBr_2_ (4.4 mg, 0.02 mmol, 10 mol%), 4,4'-di-*tert*-butyl-2,2'-bipyridine (8.1 mg, 0.15 mmol, 15 mol%), methyl 4-bromobenzoate (43 mg, 0.2 mmol, 1 equiv.), Ir[dF(CF_3_)ppy]_2_(dtbpy)PF_6_ (2.3 mg, 0.002 mmol, 1 mol%), 2,6-lutidine (32.1 mg (⁓35 µL), 0.3 mmol, 1.5 equiv.), *tert*-Butyl acrylate (51.3 mg, 0.4 mmol, 1.5 equiv.) and (1*S*,2*R*,4*R*)-1-isopropyl-4-methyl-2-((2-methylprop-1-en-1-yl)oxy)cyclohexane (126 mg, 0.6 mmol, 3 equiv.). Dissolved in the mixture solvent of benzene (1 mL) and THF (1 mL). After light irradiation with a 2 m blue LED strip at room temperature for 43 h, the crude product (89:11 *E*/*Z*) was purified by flash column chromatography to afford **39** as a colorless oil in 65% yield (96:4 *E*/*Z*, 45.1 mg).

**^1^H NMR (400 MHz, CDCl_3_, mixture of isomers *E*/*Z*, 96:4):** ***E* isomer** (major) δ 7.93 (d, *J* = 8.0 Hz, 2H), 7.22 (d, *J* = 7.9 Hz, 2H), 6.00 (s, 1H), 3.88 (s, 3H), 3.34 (td, *J* = 10.8, 4.3 Hz, 1H), 3.19 (s, 2H), 2.22 – 2.08 (m, 1H), 2.05 – 1.95 (m, 1H), 1.68 – 1.60 (m, 2H), 1.47 (s, 3H), 1.40 – 1.29 (m, 2H), 1.04 – 0.94 (m, 2H), 0.90 (t, *J* = 7.4 Hz, 6H), 0.87 – 0.80 (m, 2H), 0.77 (d, *J* = 7.0 Hz, 3H).

**^13^C NMR (101 MHz, CDCl_3_, mixture of isomers *E*/*Z*, 96:4):** ***E* isomer** (major) δ 167.3, 146.5, 141.7, 129.7, 128.9, 128.8, 128.0, 112.0, 81.4, 52.1, 48.0, 41.7, 40.7, 34.5, 31.7, 26.0, 23.6, 22.4, 21.0, 16.5, 13.2.

IR (ATR) ν 2852, 2869, 1720, 1610, 1439, 1277, 1179, 1139, 1107, 842 cm^-1^;

HRMS (EI) for C_22_H_32_O_3_^+^ (M)^+^: 344.23460; Found : 344.23475.

**Product 40**: the title compound was prepared following the general procedure using NiBr_2_ (4.4 mg, 0.02 mmol, 10 mol%), 4,4'-di-*tert*-butyl-2,2'-bipyridine (8.1 mg, 0.15 mmol, 15 mol%), methyl 4-bromobenzoate (43 mg, 0.2 mmol, 1 equiv.), Ir[dF(CF_3_)ppy]_2_(dtbpy)PF_6_ (2.3 mg, 0.002 mmol, 1 mol%), 2,6-lutidine (32.1 mg (⁓35 µL), 0.3 mmol, 1.5 equiv.), *tert*-Butyl acrylate (51.3 mg, 0.4 mmol, 1.5 equiv.), and (((2-methylprop-1-en-1-yl)oxy)methyl)benzene (97.3 mg, 0.6 mmol, 3 equiv.). Dissolved in the mixture solvent of benzene (1 mL) and THF (1 mL). After light irradiation with a 2 m blue LED strip at room temperature for 43 h, the crude product (90:10 *E*/*Z*) was purified by flash column chromatography to afford **40** as a colorless oil in 57% yield (95: 5 *E*/*Z*, 33.9 mg).

**^1^H NMR (400 MHz, CDCl_3_, mixture of isomers *E*/*Z*, 95:5):** ***E* isomer** (major) δ 7.94 (d, *J* = 8.3 Hz, 2H), 7.45 – 7.26 (m, 5H), 7.20 (d, *J* = 8.1 Hz, 2H), 6.05 (d, *J* = 1.6 Hz, 1H), 4.81 (s, 2H), 3.91 (s, 3H), 3.21 (s, 2H), 1.55 (d, *J* = 1.4 Hz, 3H); ***Z* isomer** (minor) 5.92 (d, *J* = 1.1 Hz, 1H), 4.75 (s, 2H), 3.49 (s, 2H) the remaining resonances are insufficiently resolved from those of the ***E* isomer** to be reported.

**^13^C NMR (101 MHz, CDCl_3_, mixture of isomers *E*/*Z*, 95:5):** ***E* isomer** (major) δ 167.4, 146.1, 142.2, 138.0, 129.8, 128.9, 128.7, 128.2, 128.1, 127.6, 113.8, 73.8, 52.2, 40.6, 13.3.

IR (ATR) ν 2913, 1718, 1609, 1436, 1276, 1178, 1109, 1018, 967, 835 cm^-1^;

HRMS (ESI) for C_19_H_21_O_3_^+^ (M+H)^+^ : 297.14852; Found : 297.14816.

**Product 41**: the title compound was prepared following the general procedure using NiBr_2_ (4.4 mg, 0.02 mmol, 10 mol%), 4,4'-di-*tert*-butyl-2,2'-bipyridine (8.1 mg, 0.15 mmol, 15 mol%), methyl 4-bromobenzoate (43 mg, 0.2 mmol, 1 equiv.), Ir[dF(CF_3_)ppy]_2_(dtbpy)PF_6_ (2.3 mg, 0.002 mmol, 1 mol%), 2,6-lutidine (32.1 mg (⁓35 µL), 0.3 mmol, 1.5 equiv.) and *tert*-butyldimethyl((3-methylbut-2-en-2-yl)oxy)silane (120 mg, 0.6 mmol, 3 equiv.). Dissolved in the mixture solvent of benzene (1 mL) and THF (1 mL). After light irradiation with a 2 m blue LED strip at room temperature for 43 h, the crude product was purified by flash column chromatography to afford **41** as a colorless oil in 78% yield (76: 24 *E*/*Z*, 51.9 mg).

**^1^H NMR (400 MHz, CDCl_3_, mixture of isomers *E*/*Z*, 76:24):** ***E* isomer** (major) δ 7.94 (d, *J* = 8.1 Hz, 2H), 7.21 (d, *J* = 8.1 Hz, 2H), 3.89 (s, 3H), 3.35 (s, 2H), .1.90 (s, 3H), 1.52 (s, 3H), 0.95 (s, 9H), 0.14 (s, 6H); ***Z* isomer** (minor) 3.88 (s, 3H), 3.46 (s, 2H), 1.90 (s, 3H), 1.47 (s, 3H), 0.92 (s, 9H), 0.14 (s, 6H) the remaining resonances are insufficiently resolved from those of the ***E* isomer** to be reported.

**^13^C NMR (101 MHz, CDCl_3_, mixture of isomers *E*/*Z*, 76:24):** ***E* isomer** (major) δ 167.3, 146.9, 143.2, 129.8, 128.5, 128.0, 112.0, 52.1, 39.4, 26.0, 18.9, 15.8, -3.6; ***Z* isomer** (minor) 167.4, 147.4, 142.3, 129.6, 128.8, 127.7, 110.9, 52.0, 37.2, 26.0, 18.6, 16.7, -3.4.

IR (ATR) ν 2961, 2931, 1718, 1659, 1608, 1464, 1436, 1260, 1178, 1106, 1070, 947, 915, 834 cm^-1^;

HRMS (ESI) for C_19_H_30_O_3_NaSi ^+^ (M+Na)^+^ : 357.18564; Found : 357.18584.

**Product 42**: the title compound was prepared following the general procedure using NiBr_2_ (4.4 mg, 0.02 mmol, 10 mol%), 4,4'-di-*tert*-butyl-2,2'-bipyridine (8.1 mg, 0.15 mmol, 15 mol%), methyl 4-bromobenzoate (43 mg, 0.2 mmol, 1 equiv.), Ir[dF(CF_3_)ppy]_2_(dtbpy)PF_6_ (2.3 mg, 0.002 mmol, 1 mol%), 2,6-lutidine (32.1 mg (⁓35 µL), 0.3 mmol, 1.5 equiv.) and *tert*-butyl((2,4-dimethylpent-2-en-3-yl)oxy)dimethylsilane (137 mg, 0.6 mmol, 3 equiv.). Dissolved in the mixture solvent of benzene (1 mL) and THF (1 mL). After light irradiation with a 2 m blue LED strip at room temperature for 48 h, the crude product was purified by flash column chromatography to afford **42** as a colorless oil in 67% yield (77:23 *E*/*Z*, 48.9 mg).

**^1^H NMR (600 MHz, CDCl_3_, mixture of isomers *E*/*Z*, 77:23):** ***E* isomer** (major) δ 7.95 (d, *J* = 8.2 Hz, 2H), 7.23 (d, *J* = 8.1 Hz, 2H), 3.90 (s, 3H), 3.41 (s, 2H), 2.94 – 2.85 (m, 1H), 1.52 (s, 3H), 1.06 (d, *J* = 6.6 Hz, 6H), 1.00 (s, 9H), 0.18 (s, 6H),; ***Z* isomer** (minor) 7.94 (d, *J* = 8.2 Hz, 2H), 7.21 (d, *J* = 8.1 Hz, 2H), 3.89 (s, 3H), 3.42 (s, 2H), 2.85 – 2.78 (m, 1H), 1.49 (s, 3H), 1.09 (d, *J* = 7.1 Hz, 6H), 0.93 (s, 9H), 0.14 (s, 6H).

**^13^C NMR (151 MHz, CDCl_3_, mixture of isomers *E*/*Z*, 77:23):** ***E* isomer** (major) δ 167.3, 152.0, 146.9, 129.8, 128.4, 109.4, 52.1, 39.2, 30.0, 26.6, 20.4, 19.3, 17.2, -2.8,; ***Z* isomer** (minor) 167.4, 151.1, 147.2, 129.7, 128.8, 109.1, 52.0, 38.1, 30.2, 26.4, 20.2, 19.1, 16.5, -2.8.

IR (ATR) ν 2961, 2931, 1720, 1659, 1608, 1464, 1439, 1260, 1183, 1106, 1070, 947, 915, 834, 767 cm^-1^;

HRMS (ESI) for C_21_H_34_O_3_NaSi^+^ (M+Na)^+^ : 385.21694; Found : 385.21686.

**Product 43**: the title compound was prepared following the general procedure using NiBr_2_ (4.4 mg, 0.02 mmol, 10 mol%), 4,4'-di-*tert*-butyl-2,2'-bipyridine (8.1 mg, 0.15 mmol, 15 mol%), methyl 4-bromobenzoate (43 mg, 0.2 mmol, 1 equiv.), Ir[dF(CF_3_)ppy]_2_(dtbpy)PF_6_ (2.3 mg, 0.002 mmol, 1 mol%), 2,6-lutidine (32.1 mg (⁓35 µL), 0.3 mmol, 1.5 equiv.) and *tert*-butyldimethyl((1-(*o*-tolyl)prop-1-en-1-yl)oxy)silane (mixture of isomers *E/Z*, 157 mg, 0.6 mmol, 3 equiv.). Dissolved in the mixture solvent of benzene (1 mL) and THF (1 mL). After light irradiation with a 2 m blue LED strip at room temperature for 43 h, the crude product was purified by flash column chromatography to afford **43** as a colorless oil in 74% yield (16:84 *E*/*Z*, 58.5 mg).

**^1^H NMR (400 MHz, CDCl_3_, mixture of isomers *E*/*Z*, 16:84):** ***Z* isomer** (major) δ 7.96 (d, *J* = 8.1 Hz, 2H), 7.35 (d, *J* = 8.0 Hz, 2H), 7.26 – 7.08 (m, 4H), 4.91 (t, *J* = 7.2 Hz, 1H), 3.89 (s, 3H), 3.62 (d, *J* = 7.2 Hz, 2H), 2.37 (s, 3H), 0.92 (s, 9H), -0.18 (s, 6H); ***E* isomer** (minor) 7.93 (d, *J* = 8.0 Hz, 2H), 5.20 (t, *J* = 7.8 Hz, 1H), 3.88 (s, 3H), 3.21 (d, *J* = 7.8 Hz, 2H), 2.33 (s, 3H), 0.87 (s, 9H), -0.01 (s, 6H) the remaining resonances are insufficiently resolved from those of the ***E* isomer** to be reported.

**^13^C NMR (101 MHz, CDCl_3_, mixture of isomers *E*/*Z*, 16:84):** ***Z* isomer** (major) δ 167.3, 151.3, 147.7, 139.4, 136.6, 130.2, 129.8, 129.2, 128.5, 128.3, 128.2, 127.8, 125.5, 110.3, 52.1, 31.9, 25.8, 20.2, 18.3, -4.5; ***E* isomer** (minor) 167.3, 152.1, 147.6, 137.1, 136.9, 130.4, 129.4, 128.3, 125.3, 108.1, 33.8, 25.7, 19.8, 18.2, -4.4, the remaining resonances are insufficiently resolved from those of the ***E* isomer** to be reported.

IR (ATR) ν 2952, 2932, 2858, 1722, 1656, 1609, 1436, 1277, 1182, 1107, 1029, 834, 775 cm^-1^;

HRMS (ESI) for C_24_H_33_O_3_Si^+^ (M+H)^+^ : 397.21935; Found : 397.21901.

**Product 44**: the title compound was prepared following the general procedure using NiBr_2_ (4.4 mg, 0.02 mmol, 10 mol%), 4,4'-di-*tert*-butyl-2,2'-bipyridine (8.1 mg, 0.15 mmol, 15 mol%), methyl 4-bromobenzoate (43 mg, 0.2 mmol, 1 equiv.), Ir[dF(CF_3_)ppy]_2_(dtbpy)PF_6_ (2.3 mg, 0.002 mmol, 1 mol%), 2,6-lutidine (32.1 mg (⁓35 µL), 0.3 mmol, 1.5 equiv.) and *tert-*butyldimethyl((2,4,4-trimethylpent-2-en-3-yl)oxy)silane (145 mg, 0.6 mmol, 3 equiv.). Dissolved in the mixture solvent of benzene (1 mL) and THF (1 mL). After light irradiation with a 2 m blue LED strip at room temperature for 43 h, the crude product (15:85 *E*/*Z*) was purified by flash column chromatography to afford **44** as a colorless oil in 39% yield (2:98 *E*/*Z*, 29.3 mg).

**^1^H NMR (400 MHz, CDCl_3_, mixture of isomers *E*/*Z*, 2:98):** ***Z* isomer** (major) δ 7.93 (d, *J* = 8.0 Hz, 2H), 7.20 (d, *J* = 7.9 Hz, 2H), 3.89 (s, 3H), 3.39 (s, 2H), 1.54 (s, 3H), 1.22 (s, 9H), 0.94 (s, 9H), 0.16 (s, 6H).

**^13^C NMR (101 MHz, CDCl_3_, mixture of isomers *E*/*Z*, 2:98):** ***Z* isomer** (major) δ 167.4, 153.0, 147.6, 129.7, 128.9, 127.7, 111.4, 52.1, 40.9, 36.8, 30.3, 26.5, 19.1, 18.2, -2.2.

IR (ATR) ν 2953, 2860, 1634, 1610, 1467, 1436, 1274, 1177, 1120, 1020, 942, 837, 773, 710 cm^-1^;

HRMS (ESI) for C_22_H_36_O_3_NaSi^+^ (M+Na)^+^ : 399.23259; Found : 399.23232.

**Prodcut 45**: the title compound was prepared following the general procedure using NiBr_2_ (4.4 mg, 0.02 mmol, 10 mol%), 4,4'-di-*tert*-butyl-2,2'-bipyridine (8.1 mg, 0.15 mmol, 15 mol%), methyl 4-bromobenzoate (43 mg, 0.2 mmol, 1 equiv.), Ir[dF(CF_3_)ppy]_2_(dtbpy)PF_6_ (2.3 mg, 0.002 mmol, 1 mol%), 2,6-lutidine (32.1 mg (⁓35 µL), 0.3 mmol, 1.5 equiv.) and *tert*-butyl((4,4-dimethylpent-2-en-3-yl)oxy)dimethylsilane (pure *Z*, 137 mg, 0.6 mmol, 3 equiv.). Dissolved in the mixture solvent of benzene (1 mL) and THF (1 mL). After light irradiation with a 2 m blue LED strip at room temperature for 43 h, the crude product (2:98 *E*/*Z*) was purified by flash column chromatography to afford **45** as a colorless oil in 62% yield (2:98 *E*/*Z*, 44.8 mg).

**^1^H NMR (400 MHz, CDCl_3_, mixture of isomers *E*/*Z*, 2:98):** δ ***Z* isomer** (major) 7.94 (d, *J* = 8.2 Hz, 2H), 7.23 (d, *J* = 7.9 Hz, 2H), 4.66 (t, *J* = 7.0 Hz, 1H), 3.88 (s, 3H), 3.39 (d, *J* = 6.9 Hz, 2H), 1.09 (s, 9H), 0.97 (s, 9H), 0.17 (s, 6H).

**^13^C NMR (101 MHz, CDCl_3_, mixture of isomers *E*/*Z*, 2:98):** δ ***Z* isomer** (major) 167.3, 159.6, 148.0, 129.8, 128.4, 127.8, 101.3, 52.1, 36.9, 32.3, 29.1, 26.6, 19.4, -2.6.

IR (ATR) ν 3393, 2956, 2859, 1656, 1608, 1466, 1437, 1278, 1172, 1107, 973, 828, 776 cm^-1^;

HRMS (ESI) for C_21_H_34_O_3_NaSi^+^ (M+Na)^+^ : 385.21694; Found : 385.21663.

**Product 46**: the title compound^10^ was prepared following the general procedure using NiBr_2_ (4.4 mg, 0.02 mmol, 10 mol%), 4,4'-di-*tert*-butyl-2,2'-bipyridine (8.1 mg, 0.03 mmol, 15 mol%), methyl 4-bromobenzoate (43 mg, 0.2 mmol, 1 equiv.), Ir[dF(CF_3_)ppy]_2_(dtbpy)PF_6_ (2.3 mg, 0.002 mmol, 1 mol%), 2,6-lutidine (32.1 mg (⁓35 µL), 0.3 mmol, 1.5 equiv.) and 2,4,4-trimethylpent-2-ene (67.3 mg, 0.6 mmol, 3 equiv.). Dissolved in the mixture solvent of benzene (1 mL) and THF (1 mL). After light irradiation with a 2 m blue LED strip at room temperature for 45 h, the crude product (97:3 *E*/*Z*) was purified by flash column chromatography to afford **46** as a colorless oil in 66% yield (97:3 *E*/*Z*, 32.8 mg).

**^1^H NMR (400 MHz, CDCl_3_, mixture of isomers *E*/*Z*, 97:3):** ***E* isomer** (major) δ 7.95 (d, *J* = 8.1 Hz, 2H), 7.24 (d, *J* = 8.0 Hz, 2H), 5.36 (s, 1H), 3.90 (s, 3H), 3.27 (s, 2H), 1.61 (d, *J* = 1.3 Hz, 3H), 1.13 (s, 9H); ***Z* isomer** (minor) 5.44 (s, 1H), 3.60 (s, 2H) the remaining resonances are insufficiently resolved from those of the ***E* isomer** to be reported.

**^13^C NMR (101 MHz, CDCl_3_, mixture of isomers *E*/*Z*, 97:3):** ***E* isomer** (major) δ 167.3, 146.5, 138.3, 132.4, 129.7, 128.8, 128.1, 52.1, 48.3, 32.4, 31.2, 17.0; the resonances of ***Z* isomer** are insufficiently resolved from those of the ***E* isomer** to be reported.

**Product 47**: the title compound^11^ was prepared following the general procedure using NiBr_2_ (8.8 mg, 0.04 mmol, 20 mol%), 6,6′-dimethyl-2,2′-bipyridine (7.4 mg, 0.04 mmol, 20 mol%), methyl 4-bromobenzoate (43 mg, 0.2 mmol, 1 equiv.), Ir[dF(CF_3_)ppy]_2_(dtbpy)PF_6_ (2.3 mg, 0.002 mmol, 1 mol%), 2,4,6-collidine (72.7 mg (⁓80 µL), 0.6 mmol, 3.0 equiv.) and cyclopentene (40.9 mg, 0.6 mmol, 3 equiv.). Dissolved in the mixture solvent of dioxane (1 mL) and THF (1 mL). After light irradiation with a 2 m blue LED strip at room temperature for 60 h, the crude product was purified by flash column chromatography to afford **47** as a colorless oil in 72% yield (29.3 mg).

**^1^H NMR (400 MHz, CDCl_3_):** δ 7.94 (d, *J* = 8.1 Hz, 2H), 7.23 (d, *J* = 8.1 Hz, 2H), 5.96 (dd, *J* = 5.6, 2.6 Hz, 1H), 5.74 (dd, *J* = 5.4, 2.4 Hz, 1H), 3.97 – 3.83 (m, 4H), 2.58 – 2.33 (m, 3H), 1.79 – 1.64 (m, 1H).

**^13^C NMR (101 MHz, CDCl_3_):** δ 167.3, 152.1, 133.6, 132.8, 129.9, 128.1, 127.3, 52.1, 51.4, 33.7, 32.6.

**Product 48**: the title compound^12^ was prepared following the general procedure using NiBr_2_ (8.8 mg, 0.04 mmol, 20 mol%), 6,6′-dimethyl-2,2′-bipyridine (7.4 mg, 0.04 mmol, 20 mol%), methyl 4-bromobenzoate (43 mg, 0.2 mmol, 1 equiv.), Ir[dF(CF_3_)ppy]_2_(dtbpy)PF_6_ (2.3 mg, 0.002 mmol, 1 mol%), 2,4,6-collidine (72.7 mg (⁓80 µL), 0.6 mmol, 3.0 equiv.) and cyclohexene (49.3 mg, 0.6 mmol, 3 equiv.). Dissolved in the mixture solvent of dioxane (1 mL) and THF (1 mL). After light irradiation with a 2 m blue LED strip at room temperature for 60 h, the crude product was purified by flash column chromatography to afford **48** as a colorless oil in 74% yield (32 mg). **^1^H NMR (600 MHz, CDCl_3_):** δ 7.97 (d, *J* = 8.0 Hz, 2H), 7.28 (d, *J* = 8.0 Hz, 2H), 5.93 (dd, *J* = 10.4, 3.0 Hz, 1H), 5.69 (d, *J* = 9.2 Hz, 1H), 3.90 (s, 3H), 3.46 (dd, *J* = 5.7, 2.8 Hz, 1H), 2.16 – 1.95 (m, 3H), 1.80 – 1.70 (m, 1H), 1.70 – 1.58 (m, 1H), 1.58 – 1.51 (m, 1H)., **^13^C NMR (151 MHz, CDCl_3_):** 167.3, 152.3, 129.8, 129.4, 129.1, 128.1, 127.9, 52.1, 42.0, 32.5, 25.1, 21.2.

**Prodcut 49**: the title compound^13^ was prepared following the general procedure using NiBr_2_•glyme (6.2 mg, 0.02 mmol, 10 mol%), 4,4'-di-*tert*-butyl-2,2'-bipyridine (8.1 mg, 0.03 mmol, 15 mol%), methyl 4-bromobenzoate (43 mg, 0.2 mmol, 1 equiv.), Ir[dF(CF_3_)ppy]_2_(dtbpy)PF_6_ (4.5 mg, 0.004 mmol, 2 mol%), 2,6-lutidine (32.1 mg (⁓35 µL), 0.3 mmol, 1.5 equiv.) and trans-β-Methylstyrene (118 mg, 1 mmol, 5 equiv.). Dissolved in benzene (2 mL). After light irradiation with a 2 m blue LED strip at room temperature for 96 h, the solvent was removed carefully under high vacuum and exchanged to 1 mL THF. After removing THF, 1 equiv. PhSSPh (43.7 mg, 0.2 mmol, 1 equiv.) in THF (2 mL) was added. The mixture was stirred under 2 m blue LED strip for 1 h. The crude product (93:7 *E*/*Z*) was purified by flash column chromatography to afford **49** as a colorless oil in 53% yield (93:7 *E*/*Z*, 26.8 mg).

**^1^H NMR (400 MHz, CDCl_3_, mixture of isomers *E*/*Z*, 93:7):** ***E* isomer** (major) δ 7.99 (d, *J* = 8.0 Hz, 2H), 7.47 – 7.27 (m, 6H), 7.27 – 7.18 (m, 1H), 6.47 (d, *J* = 15.8 Hz, 1H), 6.34 (dt, *J* = 15.7, 6.7 Hz, 1H), 3.91 (s, 3H), 3.61 (d, *J* = 6.9 Hz, 2H).

**^13^C NMR (101 MHz, CDCl_3_, mixture of isomers *E*/*Z***, **93:7):** ***E* isomer** (major) δ 167.1, 145.6, 137.2, 131.8, 129.8, 128.7, 128.5, 128.2, 128.0, 127.3, 126.1, 52.0, 39.3.

**Prodcut 50**: the title compound was prepared following the general procedure using NiBr_2_•glyme (6.2 mg, 0.02 mmol, 10 mol%), 4,4'-di-*tert*-butyl-2,2'-bipyridine (8.1 mg, 0.03 mmol, 15 mol%), methyl 4-bromobenzoate (43 mg, 0.2 mmol, 1 equiv.), Ir[dF(CF_3_)ppy]_2_(dtbpy)PF_6_ (4.5 mg, 0.004 mmol, 2 mol%), 2,6-lutidine (32.1 mg (⁓35 µL), 0.3 mmol, 1.5 equiv.) and methyl eugenol (178 mg, 1 mmol, 5 equiv.). Dissolved in benzene (2 mL). After light irradiation with a 2 m blue LED strip at room temperature for 96 h, the solvent was removed carefully under high vacuum and exchanged to 1 mL THF. After removing THF, 1 equiv. PhSSPh (43.7 mg, 0.2 mmol, 1 equiv.) in THF (2 mL) was added. The mixture was stirred under 2 m blue LED strip for 1 h. The crude product (95:5 *E*/*Z*) was purified by flash column chromatography to afford **50** as a colorless oil in 50% yield (only *E* isomer, 31.4 mg).

**^1^H NMR (400 MHz, CDCl_3_, only *E* isomer):** δ 7.98 (d, *J* = 7.9 Hz, 2H), 7.32 (d, *J* = 7.9 Hz, 2H), 6.95 – 6.86 (m, 2H), 6.80 (d, *J* = 8.1 Hz, 1H), 6.40 (d, *J* = 15.7 Hz, 1H), 6.19 (dt, *J* = 15.2, 6.9 Hz, 1H), 3.90 (s, 3H), 3.89 – 3.84 (m, 6H), 3.58 (d, *J* = 6.8 Hz, 2H).

**^13^C NMR (101 MHz, CDCl_3_, only *E* isomer):** δ 167.2, 149.1, 148.7, 145.9, 131.5, 130.4, 129.9, 128.8, 128.3, 126.2, 119.3, 111.2, 108.7, 56.0, 55.9, 52.1, 39.4.

HRMS (ESI) for C_19_H_20_O_4_Na^+^ (M+Na)^+^ : 335.1254; Found : 335.1251.

**Prodcut 51**: the title compound was prepared following the general procedure using NiBr_2_ (8.8 mg, 0.04 mmol, 20 mol%), 6,6′-dimethyl-2,2′-bipyridine (7.4 mg, 0.04 mmol, 20 mol%), methyl 3-bromobenzoate (43 mg, 0.2 mmol, 1 equiv.), Ir[dF(CF_3_)ppy]_2_(dtbpy)PF_6_ (2.3 mg, 0.002 mmol, 1 mol%), 2,4,6-collidine (72.7 mg (⁓80 µL), 0.6 mmol, 3.0 equiv.) and *tert*-butyldimethyl((2-methylprop-1-en-1-yl)oxy)silane (112 mg, 0.6 mmol, 3 equiv.). Dissolved in the mixture solvent of dioxane (1 mL) and THF (1 mL). After light irradiation with a 2 m blue LED strip at room temperature for 72 h, the crude product (10:90 *E*/*Z*) was purified by flash column chromatography to afford **51** as a colorless oil in 45% yield (only *Z*, 28.8 mg).

**^1^H NMR (400 MHz, CDCl_3_, only *Z* isomer):** δ 7.89 (d, *J* = 1.6 Hz, 1H), 7.85 (d, *J* = 7.5 Hz, 1H), 7.39 (d, *J* = 7.6 Hz, 1H), 7.32 (t, *J* = 7.6 Hz, 1H), 6.19 (s, 1H), 3.90 (s, 3H), 3.45 (s, 2H), 1.45 (d, *J* = 1.4 Hz, 3H), 0.95 (s, 9H), 0.17 (s, 6H).

**^13^C NMR (101 MHz, CDCl_3_, only *Z* isomer):** δ 167.5, 141.5, 134.8, 133.5, 130.2, 130.0, 128.3, 127.1, 115.3, 52.1, 35.0, 25.8, 18.3, 17.0, -5.1.

IR (ATR) ν 2953, 2931, 2857, 1724, 1675, 1438, 1277, 1188, 1140, 995, 838, 780 cm^-1^;

HRMS (ESI) for C_18_H_28_O_3_NaSi^+^ (M+Na)^+^ : 343.16999; Found : 343.16954.

**Prodcut 52**: the title compound was prepared following the general procedure using NiBr_2_ (8.8 mg, 0.04 mmol, 20 mol%), 6,6′-dimethyl-2,2′-bipyridine (7.4 mg, 0.04 mmol, 20 mol%), methyl 4-bromobenzoate (43 mg, 0.2 mmol, 1 equiv.), Ir[dF(CF_3_)ppy]_2_(dtbpy)PF_6_ (2.3 mg, 0.002 mmol, 1 mol%), 2,4,6-collidine (72.7 mg (⁓80 µL), 0.6 mmol, 3.0 equiv.) and triisopropyl((2-methylprop-1-en-1-yl)oxy)silane (137 mg, 0.6 mmol, 3 equiv.). Dissolved in the mixture solvent of dioxane (1 mL) and THF (1 mL). After light irradiation with a 2 m blue LED strip at room temperature for 60 h, the crude product (8:92 *E*/*Z*) was purified by flash column chromatography to afford **52** as a colorless oil in 73% yield (only *Z* isomer, 52.7 mg).

**^1^H NMR (400 MHz, CDCl_3_, only *Z* isomer):** δ 7.94 (d, *J* = 8.4 Hz, 2H), 7.28 (d, *J* = 8.2 Hz, 2H), 6.28 (d, *J* = 1.7 Hz, 1H), 3.89 (s, 3H), 3.48 (s, 2H), 1.44 (d, *J* = 1.5 Hz, 3H), 1.22 – 1.13 (m, 3H), 1.10 (s, 18H); 1.13 – 1.07 (m, 18H).

**^13^C NMR (101 MHz, CDCl_3_, only *Z* isomer):** δ 167.2, 146.8, 135.2, 129.5, 128.7, 127.5, 113.9, 51.9, 35.1, 17.8, 16.8, 11.9.

IR (ATR) ν 2945, 2866, 1722, 1673, 1461, 1436, 1276, 1183, 1142, 1016, 880, 815 cm^-1^;

HRMS (ESI) for C_21_H_34_O_3_NaSi^+^ (M+Na)^+^ : 385.21694; Found : 385.21656.

**Product 53**: the title compound was prepared following the general procedure using NiBr_2_ (8.8 mg, 0.04 mmol, 20 mol%), 6,6′-dimethyl-2,2′-bipyridine (7.4 mg, 0.04 mmol, 20 mol%), 1-bromo-4-(trifluoromethyl)benzene (45 mg, 0.2 mmol, 1 equiv.), Ir[dF(CF_3_)ppy]_2_(dtbpy)PF_6_ (2.3 mg, 0.002 mmol, 1 mol%), 2,4,6-collidine (72.7 mg (⁓80 µL), 0.6 mmol, 3.0 equiv.) and triisopropyl((2-methylprop-1-en-1-yl)oxy)silane (137 mg, 0.6 mmol, 3 equiv.). Dissolved in the mixture solvent of dioxane (1 mL) and THF (1 mL). After light irradiation with a 2 m blue LED strip at room temperature for 72 h, the crude product (9:91 *E*/*Z*) was purified by flash column chromatography to afford **53** as a colorless oil in 63% yield (only *Z* isomer, 46.8 mg).

**^1^H NMR (400 MHz, CDCl_3_, only *Z* isomer):** δ 7.52 (d, *J* = 7.9 Hz, 2H), 7.32 (d, *J* = 8.0 Hz, 2H), 6.29 (d, *J* = 1.6 Hz, 1H), 3.49 (s, 2H), 1.45 (d, *J* = 1.5 Hz, 3H), 1.24 – 1.15 (m, 3H), 1.13 – 1.07 (m, 18H).

**^13^C NMR (101 MHz, CDCl_3_, only *Z* isomer):** δ 145.6, 135.5, 129.1, 128.1, 125.2, 124.6, 114.1, 35.1, 17.9, 17.0, 12.1.

**^19^F NMR (376.3 MHz, CDCl_3_, only *Z* isomer):** δ -62.2.

IR (ATR) ν 2944, 2867, 1673, 1463, 1323, 1160, 1125, 1066, 1015, 881, 855, 815 cm^-1^;

HRMS (ESI) for C_20_H_31_OF_3_NaSi^+^ (M+Na)^+^ : 395.19885; Found : 395.19812.

**Product 54**: the title compound was prepared following the general procedure using NiBr_2_ (8.8 mg, 0.04 mmol, 20 mol%), 6,6′-dimethyl-2,2′-bipyridine (7.4 mg, 0.04 mmol, 20 mol%), (4-bromophenyl)(phenyl)methanone (52.2 mg, 0.2 mmol, 1 equiv.), Ir[dF(CF_3_)ppy]_2_(dtbpy)PF_6_ (2.3 mg, 0.002 mmol, 1 mol%), 2,4,6-collidine (72.7 mg (⁓80 µL), 0.6 mmol, 3.0 equiv.) and triisopropyl((2-methylprop-1-en-1-yl)oxy)silane (137 mg, 0.6 mmol, 3 equiv.). Dissolved in the mixture solvent of dioxane (1 mL) and THF (1 mL). After light irradiation with a 2 m blue LED strip at room temperature for 72 h, the crude product (11:89 *E*/*Z*) was purified by flash column chromatography to afford **54** as a colorless oil in 72% yield (only *Z* isomer, 53.0 mg).

**^1^H NMR (400 MHz, CDCl_3_, only *Z* isomer):** δ 7.80 (d, *J* = 7.2 Hz, 2H), 7.73 (d, *J* = 8.1 Hz, 2H), 7.57 (t, *J* = 7.4 Hz, 1H), 7.47 (t, *J* = 7.6 Hz, 2H), 7.33 (d, *J* = 8.0 Hz, 2H), 6.31 (s, 1H), 3.52 (s, 2H), 1.48 (s, 3H), 1.25 – 1.14 (m, 3H), 1.13 – 1.08 (m, 18H).

**^13^C NMR (101 MHz, CDCl_3_, only *Z* isomer):** δ 196.7, 146.7, 138.2, 135.5, 135.2, 132.2, 130.4, 130.1, 128.8, 128.3, 114.1, 35.3, 17.9, 17.9, 17.0, 12.1.

IR (ATR) ν 2943, 2866, 2323, 2106, 1918, 1659, 1603, 1459, 1311, 1276, 1182, 1142, 997, 922, 881, 816, 737 cm^-1^;

HRMS (ESI) for C_26_H_37_O_2_Si^+^ (M+H)^+^ : 409.25573; Found : 409.25501.

**Product 55**: the title compound was prepared following the general procedure using NiBr_2_ (8.8 mg, 0.04 mmol, 20 mol%), 6,6′-dimethyl-2,2′-bipyridine (7.4 mg, 0.04 mmol, 20 mol%), 1-(4-bromophenyl)ethan-1-one (39.8 mg, 0.2 mmol, 1 equiv.), Ir[dF(CF_3_)ppy]_2_(dtbpy)PF_6_ (2.3 mg, 0.002 mmol, 1 mol%), 2,4,6-collidine (72.7 mg (⁓80 µL), 0.6 mmol, 3.0 equiv.) and triisopropyl((2-methylprop-1-en-1-yl)oxy)silane (137 mg, 0.6 mmol, 3 equiv.). Dissolved in the mixture solvent of dioxane (1 mL) and THF (1 mL). After light irradiation with a 2 m blue LED strip at room temperature for 60 h, the crude product (10:90 *E*/*Z*) was purified by flash column chromatography to afford **55** as a colorless oil in 63% yield (3:97 *E*/*Z*, 43.7 mg).

**^1^H NMR (400 MHz, CDCl_3_, mixture of isomers *E*/*Z*, 3:97):** ***Z* isomer** (major) δ 7.85 (d, *J* = 8.1 Hz, 2H), 7.28 (d, *J* = 8.1 Hz, 2H), 6.27 (s, 1H), 3.47 (s, 2H), 2.56 (s, 3H), 1.42 (d, *J* = 1.5 Hz, 3H), 1.22 – 1.12 (m, 3H), 1.10 – 1.05 (m, 18H).

**^13^C NMR (101 MHz, CDCl_3_, mixture of isomers *E*/*Z*, 3:97):** ***Z* isomer** (major) δ 198.1, 147.4, 135.5, 135.0, 129.0, 128.5, 114.0, 35.2, 26.7, 17.9, 17.0, 12.1.

IR (ATR) ν 2942, 2866, 1679, 1605, 1462, 1358, 1265, 1183, 1142, 1013, 881, 816 cm^-1^;

HRMS (ESI) for C_21_H_34_O_2_NaSi^+^ (M+Na)^+^ : 369.22203; Found : 369.22160.

**Product 56**: the title compound was prepared following the general procedure using NiBr_2_ (8.8 mg, 0.04 mmol, 20 mol%), 6,6′-dimethyl-2,2′-bipyridine (7.4 mg, 0.04 mmol, 20 mol%), 4-bromobenzonitrile (36.4 mg, 0.2 mmol, 1 equiv.), Ir[dF(CF_3_)ppy]_2_(dtbpy)PF_6_ (2.3 mg, 0.002 mmol, 1 mol%), 2,4,6-collidine (72.7 mg (⁓80 µL), 0.6 mmol, 3.0 equiv.) and triisopropyl((2-methylprop-1-en-1-yl)oxy)silane (137 mg, 0.6 mmol, 3 equiv.). Dissolved in the mixture solvent of dioxane (1 mL) and THF (1 mL). After light irradiation with a 2 m blue LED strip at room temperature for 70 h, the crude product (18:82 *E*/*Z*) was purified by flash column chromatography to afford **56** as a colorless oil in 64% yield (only *Z* isomer, 42.3 mg).

**^1^H NMR (400 MHz, CDCl_3_, only *Z* isomer):** δ 7.54 (d, *J* = 8.2 Hz, 2H), 7.30 (d, *J* = 8.1 Hz, 2H), 6.29 (d, *J* = 1.6 Hz, 1H), 3.47 (s, 2H), 1.44 (d, *J* = 1.5 Hz, 3H), 1.22 – 1.12 (m, 3H), 1.11 – 1.05 (m, 18H).

**^13^C NMR (101 MHz, CDCl_3_, only *Z* isomer):** δ 147.2, 135.9, 132.1, 129.6, 119.4, 113.4, 109.5, 35.4, 17.9, 17.0, 12.0.

IR (ATR) ν 2943, 2866, 2228, 1672, 1607, 1462, 1242, 1184, 1142, 1069, 1012, 881, 856, 813 cm^-1^;

HRMS (ESI) for C_20_H_31_ONNaSi^+^ (M+Na)^+^ : 352.20671; Found : 352.20627.

**Product 57**: the title compound was prepared following the general procedure using NiBr_2_ (8.8 mg, 0.04 mmol, 20 mol%), 6,6′-dimethyl-2,2′-bipyridine (7.4 mg, 0.04 mmol, 20 mol%), 4-bromobenzenesulfonamide (47.2 mg, 0.2 mmol, 1 equiv.), Ir[dF(CF_3_)ppy]_2_(dtbpy)PF_6_ (2.3 mg, 0.002 mmol, 1 mol%), 2,4,6-collidine (72.7 mg (⁓80 µL), 0.6 mmol, 3.0 equiv.) and triisopropyl((2-methylprop-1-en-1-yl)oxy)silane (137 mg, 0.6 mmol, 3 equiv.). Dissolved in the mixture solvent of dioxane (1 mL) and THF (1 mL). After light irradiation with a 2 m blue LED strip at room temperature for 66 h, the crude product (10:90 *E*/*Z*) was purified by flash column chromatography to afford **57** as a colorless oil in 56% yield (9:91 *E*/*Z*, 42.8 mg).

**^1^H NMR (400 MHz, CDCl_3_, mixture of isomers *E*/*Z*, 9:91):** ***Z* isomer** (major) δ 7.81 (d, *J* = 7.9 Hz, 2H), 7.34 (d, *J* = 8.0 Hz, 2H), 6.29 (s, 1H), 5.05 (s, 2H), 3.48 (s, 2H), 1.44 (s, 3H), 1.23 – 1.13 (m, 3H), 1.11 – 1.06 (m, 18H); ***E* isomer** (minor) 7.30 (d, *J* = 8.0 Hz, 2H), 6.34 (s, 1H), 5.07 (s, 2H), 3.22 (s, 2H), 1.50 (s, 3H) the remaining resonances are insufficiently resolved from those of the ***Z* isomer** to be reported.

**^13^C NMR (101 MHz, CDCl_3_, mixture of isomers *E*/*Z*, 9:91):** ***Z* isomer** (major) δ 147.1, 139.3, 135.7, 129.5, 126.5, 126.5, 113.7, 35.1, 17.9, 16.9, 12.1.

IR (ATR) ν 3268, 2942, 2866, 1672, 1462, 1328, 1156, 1012, 914, 882, 816 cm^-1^;

HRMS (ESI) for C_19_H_33_O_3_NNaSSi^+^ (M+Na)^+^ : 406.18426; Found : 406.18393.

**Product 58**: the title compound was prepared following the general procedure using NiBr_2_ (4.4 mg, 0.02 mmol, 20 mol%), 6,6′-dimethyl-2,2′-bipyridine (3.7 mg, 0.02 mmol, 20 mol%), 2-bromonaphthalene (20.7 mg, 0.1 mmol, 1 equiv.), Ir[dF(CF_3_)ppy]_2_(dtbpy)PF_6_ (1.2 mg, 0.001 mmol, 1 mol%), LiBr (8.7 mg, 0.1 mmol, 1 equiv.), 2,4,6-collidine (18.2 mg (⁓20 µL), 0.15 mmol, 1.5 equiv.) and triisopropyl((2-methylprop-1-en-1-yl)oxy)silane (68.5 mg, 0.3 mmol, 3 equiv.). Dissolved in the mixture solvent of dioxane (0.5 mL) and THF (0.5 mL). After light irradiation with a 2 m blue LED strip at room temperature for 72 h, the crude product (9:91 *E*/*Z*) was purified by flash column chromatography to afford **58** as a colorless oil in 51% yield (only *Z* isomer, 35.9 mg; this result is based on two individual runs).

**^1^H NMR (400 MHz, CDCl_3_, only *Z* isomer):** δ 7.88 – 7.72 (m, 3H), 7.67 (s, 1H), 7.51 – 7.35 (m, 3H), 6.34 (s, 1H), 3.63 (s, 2H), 1.50 (s, 3H), 1.28 – 1.20 (m, 3H), 1.18 – 1.12 (m, 18H).

**^13^C NMR (101 MHz, CDCl_3_, only *Z* isomer):** δ 138.9, 135.0, 133.7, 132.1, 127.9, 127.8, 127.7, 127.5, 126.9, 125.8, 125.0, 115.0, 35.3, 18.0, 17.0, 12.2.

IR (ATR) ν 2942, 2865, 1673, 1462, 1245, 1184, 1151, 1012, 882, 811, 748 cm^-1^;

HRMS (ESI) for C_23_H_34_ONaSi^+^ (M+Na)^+^ : 377.22711; Found : 377.22658.

**Product 59**: the title compound was prepared following the general procedure using NiBr_2_ (4.4 mg, 0.02 mmol, 20 mol%), 6,6′-dimethyl-2,2′-bipyridine (3.7 mg, 0.02 mmol, 20 mol%), 4-bromo-1,1'-biphenyl (23.3 mg, 0.1 mmol, 1 equiv.), Ir[dF(CF_3_)ppy]_2_(dtbpy)PF_6_ (1.2 mg, 0.001 mmol, 1 mol%), LiBr (8.7 mg, 0.1 mmol, 1 equiv.), 2,4,6-collidine (18.2 mg (⁓20 µL), 0.15 mmol, 1.5 equiv.) and triisopropyl((2-methylprop-1-en-1-yl)oxy)silane (68. 5 mg, 0.3 mmol, 3 equiv.). Dissolved in the mixture solvent of dioxane (0.5 mL) and THF (0.5 mL). After light irradiation with a 2 m blue LED strip at room temperature for 72 h, the crude product (10:90 *E*/*Z*) was purified by flash column chromatography to afford **59** as a colorless oil in 57% yield (only *Z* isomer, 43.4 mg; this result is based on two individual runs).

**^1^H NMR (400 MHz, CDCl_3_, only *Z* isomer):** δ 7.62 (d, *J* = 7.2 Hz, 2H), 7.53 (d, *J* = 8.2 Hz, 2H), 7.45 (t, *J* = 7.6 Hz, 2H), 7.33 (dd, *J* = 10.9, 7.7 Hz, 3H), 6.32 (s, 1H), 3.52 (s, 2H), 1.52 (d, *J* = 1.4 Hz, 3H), 1.27 – 1.19 (m, 3H), 1.18 – 1.12 (m, 18H).

**^13^C NMR (101 MHz, CDCl_3_, only *Z* isomer):** δ 148.4, 138.2, 134.7, 128.6, 125.2, 115.3, 34.6, 31.6, 18.0, 17.1, 12.2.

IR (ATR) ν 2942, 2865, 2087, 1672, 1462, 1380, 1242, 1183, 1141, 1069, 1006, 881, 818 cm^-1^;

HRMS (ESI) for C_25_H_36_ONaSi^+^ (M+Na)^+^ : 403.24276; Found : 403.24191.

**Product 60**: the title compound was prepared following the general procedure using NiBr_2_ (4.4 mg, 0.02 mmol, 20 mol%), 6,6′-dimethyl-2,2′-bipyridine (3.7 mg, 0.02 mmol, 20 mol%), 1-bromo-4-(tert-butyl)benzene (21.3 mg, 0.1 mmol, 1 equiv.), Ir[dF(CF_3_)ppy]_2_(dtbpy)PF_6_ (1.2 mg, 0.001 mmol, 1 mol%), LiBr (8.7 mg, 0.1 mmol, 1 equiv.), 2,4,6-collidine (18.2 mg (⁓20 µL), 0.15 mmol, 1.5 equiv.) and triisopropyl((2-methylprop-1-en-1-yl)oxy)silane (68.5 mg, 0.3 mmol, 3 equiv.). Dissolved in the mixture solvent of dioxane (0.5 mL) and THF (0.5 mL). After light irradiation with a 2 m blue LED strip at room temperature for 72 h, the crude product (10:90 *E*/*Z*) was purified by flash column chromatography to afford **60** as a colorless oil in 40% yield (only *Z* isomer, 28.8 mg; this result is based on two individual runs).

**^1^H NMR (400 MHz, CDCl_3_, only *Z* isomer):** δ 7.29 (d, *J* = 8.2 Hz, 2H), 7.16 (d, *J* = 8.0 Hz, 2H), 6.26 (s, 1H), 3.42 (s, 2H), 1.45 (d, *J* = 1.4 Hz, 3H), 1.32 (s, 9H), 1.23 – 1.15 (m, 3H), 1.15 – 1.06 (m, 18H).

**^13^C NMR (101 MHz, CDCl_3_, only *Z* isomer):** δ 148.4, 138.2, 134.7, 128.6, 125.2, 115.3, 34.6, 31.6, 18.0, 17.1, 12.2.

IR (ATR) ν 2950, 2866, 1673, 1510, 1462, 1365, 1243, 1184, 1141, 1013, 919, 881, 818 cm^-1^;

HRMS (ESI) for C_23_H_40_ONaSi^+^ (M+Na)^+^ : 383.27406; Found : 383.27357.

**Product 61**: the title compound was prepared following the general procedure using NiBr_2_ (4.4 mg, 0.02 mmol, 20 mol%), 6,6′-dimethyl-2,2′-bipyridine (3.7 mg, 0.02 mmol, 20 mol%), 1-bromo-3-methoxybenzene (18.7 mg, 0.1 mmol, 1 equiv.), Ir[dF(CF_3_)ppy]_2_(dtbpy)PF_6_ (1.2 mg, 0.001 mmol, 1 mol%), LiBr (8.7 mg, 0.1 mmol, 1 equiv.), 2,4,6-collidine (18.2 mg (⁓20 µL), 0.15 mmol, 1.5 equiv.) and triisopropyl((2-methylprop-1-en-1-yl)oxy)silane (68.5 mg, 0.3 mmol, 3 equiv.). Dissolved in the mixture solvent of dioxane (0.5 mL) and THF (0.5 mL). After light irradiation with a 2 m blue LED strip at room temperature for 72 h, the crude product (10:90 *E*/*Z*) was purified by flash column chromatography to afford **61** as a colorless oil in 44% yield (only *Z* isomer, 29.3 mg; this result is based on two individual runs).

**^1^H NMR (400 MHz, CDCl_3_, only *Z* isomer):** δ 7.18 (t, *J* = 7.8 Hz, 1H), 6.87 – 6.76 (m, 2H), 6.73 (dd, *J* = 8.2, 2.6 Hz, 1H), 6.27 (s, 1H), 3.78 (s, 3H), 3.43 (s, 2H), 1.45 (d, *J* = 1.5 Hz, 3H), 1.24 – 1.15 (m, 3H), 1.13 – 1.09 (m, 18H).

**^13^C NMR (101 MHz, CDCl_3_, only *Z* isomer):** δ 159.7, 143.0, 134.9, 129.1, 121.5, 115.0, 114.3, 111.4, 55.2, 35.2, 18.0, 17.0, 12.1.

IR (ATR) ν 2943, 2866, 1674, 1597, 1461, 1257, 1184, 1142, 1051, 996, 881, 816, 778, 741 cm^-1^;

HRMS (ESI) for C_20_H_34_O_2_NaSi^+^ (M+Na)^+^ : 357.22203; Found : 357.22139.

**Prodcut 62**: the title compound was prepared following the general procedure using NiBr_2_ (8.8 mg, 0.04 mmol, 20 mol%), 6,6′-dimethyl-2,2′-bipyridine (7.4 mg, 0.04 mmol, 20 mol%), 1-(3-bromophenyl)ethan-1-one (39.8 mg, 0.2 mmol, 1 equiv.), Ir[dF(CF_3_)ppy]_2_(dtbpy)PF_6_ (2.3 mg, 0.002 mmol, 1 mol%), 2,4,6-collidine (72.7 mg (⁓80 µL), 0.6 mmol, 3.0 equiv.) and triisopropyl((2-methylprop-1-en-1-yl)oxy)silane (137 mg, 0.6 mmol, 3 equiv.). Dissolved in the mixture solvent of dioxane (1 mL) and THF (1 mL). After light irradiation with a 2 m blue LED strip at room temperature for 72 h, the crude product (8:92 *E*/*Z*) was purified by flash column chromatography to afford **62** as a colorless oil in 53% yield (only *Z* isomer, 36.4 mg).

**^1^H NMR (400 MHz, CDCl_3_, only *Z* isomer):** δ 7.79 (s, 1H), 7.75 (d, *J* = 7.7 Hz, 1H), 7.40 (d, *J* = 7.6 Hz, 1H), 7.33 (t, *J* = 7.6 Hz, 1H), 6.27 (s, 1H), 3.47 (s, 2H), 2.56 (s, 3H), 1.43 (d, *J* = 1.4 Hz, 3H), 1.22 – 1.12 (m, 3H), 1.11 – 1.06 (m, 18H). **^13^C NMR (101 MHz, CDCl_3_, only *Z* isomer):** δ 198.5, 141.8, 137.3, 135.3, 133.7, 128.8, 128.5, 125.9, 114.4, 35.0, 26.8, 17.9, 17.0, 12.1.

IR (ATR) ν 2943, 2866, 1684, 1462, 1436, 1357, 1267, 1184, 1142, 1070, 997, 881, 816 cm^-1^;

HRMS (ESI) for C_21_H_34_O_2_NaSi^+^ (M+Na)^+^ : 369.22203; Found : 369.22157.

**Prodcut 63**: the title compound was prepared following the general procedure using NiBr_2_ (8.8 mg, 0.04 mmol, 20 mol%), 6,6′-dimethyl-2,2′-bipyridine (7.4 mg, 0.04 mmol, 20 mol%), 5-bromo-2-(trifluoromethyl)pyridine (45.2 mg, 0.2 mmol, 1 equiv.), Ir[dF(CF_3_)ppy]_2_(dtbpy)PF_6_ (2.3 mg, 0.002 mmol, 1 mol%), 2,4,6-collidine (72.7 mg (⁓80 µL), 0.6 mmol, 3.0 equiv.) and triisopropyl((2-methylprop-1-en-1-yl)oxy)silane (137 mg, 0.6 mmol, 3 equiv.). Dissolved in the mixture solvent of dioxane (1 mL) and THF (1 mL). After light irradiation with a 2 m blue LED strip at room temperature for 60 h, the crude product (26:74 *E*/*Z*) was purified by flash column chromatography to afford **63** as a colorless oil in 76% yield (26:74 *E*/*Z*, 56.4 mg).

**^1^H NMR (600 MHz, CDCl_3_, mixture of isomers *E*/*Z*, 26:74):** ***Z* isomer** (major) δ 8.56 (s), 7.67 (d, *J* = 8.2 Hz, 1H), 7.61 – 7.54 (m, 1H), 6.30 (s, 1H), 3.48 (s, 2H), 1.45 (s, 3H), 1.20 – 1.14 (m, 3H), 1.10 – 1.06 (m, 18H); ***E* isomer** (minor) 8.53 (s), 7.64 (d, *J* = 8.0 Hz, 1H), 6.36 (s, 1H), 3.24 (s, 2H), 1.51 (s, 3H), the remaining resonances are insufficiently resolved from those of the ***Z* isomer** to be reported.

**^13^C NMR (151 MHz, CDCl_3_, mixture of isomers *E*/*Z*, 26:74):** ***Z* isomer** (major) δ 150.6, 145.9 (q, *J*_C-F_ = 34.5 Hz), 140.2, 137.3, 136.2, 121.9 (q, *J*_C-F_ = 274.3 Hz), 120.2 (q, *J*_C-F_ = 2.2 Hz), 112.9, 37.4, 32.3, 17.9, 12.1; ***E* isomer** (minor) 150.5, 139.8, 137.3, 113.8, 16.9, 12.1 the remaining resonances are insufficiently resolved from those of the ***Z* isomer** to be reported.

**^19^F NMR (376.3 MHz, CDCl_3_, mixture of isomers *E*/*Z*, 26:74):** ***Z* isomer** (major) δ -67.68; ***E* isomer** (minor) -67.71.

IR (ATR) ν 2945, 2868, 1673, 1463, 1335, 1138, 1084, 1019, 881, 812 cm^-1^;

HRMS (ESI) for C_19_H_31_ONF_3_Si^+^ (M+H)^+^ : 374.21215; Found : 374.21151.

**Product 52**: the title compound was prepared following the general procedure using NiBr_2_ (8.8 mg, 0.04 mmol, 20 mol%), 6,6′-dimethyl-2,2′-bipyridine (7.4 mg, 0.04 mmol, 20 mol%), methyl 4-bromobenzoate (43 mg, 0.2 mmol, 1 equiv.), Ir[dF(CF_3_)ppy]_2_(dtbpy)PF_6_ (2.3 mg, 0.002 mmol, 1 mol%), 2,4,6-collidine (72.7 mg (⁓80 µL), 0.6 mmol, 3.0 equiv.) and triisopropyl((2-methylallyl)oxy)silane (137 mg, 0.6 mmol, 3 equiv.). Dissolved in the mixture solvent of dioxane (1 mL) and THF (1 mL). After light irradiation with a 2 m blue LED strip at room temperature for 72 h, the crude product (10:90 *E*/*Z*) was purified by flash column chromatography to afford **52** as a colorless oil in 61% yield (only *Z* isomer, 44.3 mg).

**^1^H NMR (400 MHz, CDCl_3_, only *Z* isomer):** δ 7.94 (d, *J* = 8.4 Hz, 2H), 7.28 (d, *J* = 8.2 Hz, 2H), 6.28 (d, *J* = 1.7 Hz, 1H), 3.89 (s, 3H), 3.48 (s, 2H), 1.44 (d, *J* = 1.5 Hz, 3H), 1.22 – 1.13 (m, 3H), 1.10 (s, 18H); 1.13 – 1.07 (m, 18H).

**^13^C NMR (101 MHz, CDCl_3_, only *Z* isomer):** δ 167.2, 146.8, 135.2, 129.5, 128.7, 127.5, 113.9, 51.9, 35.1, 17.8, 16.8, 11.9.

IR (ATR) ν 2945, 2866, 1722, 1673, 1461, 1436, 1276, 1183, 1142, 1016, 880, 815 cm^-1^;

HRMS (ESI) for C_21_H_34_O_3_NaSi^+^ (M+Na)^+^ : 385.21694; Found : 385.21656.

**Product 64**: the title compound was prepared following the general procedure using NiBr_2_ (4.4 mg, 0.02 mmol, 20 mol%), 6,6′-dimethyl-2,2′-bipyridine (3.7 mg, 0.02 mmol, 20 mol%), methyl 4-bromobenzoate (21.5 mg, 0.1 mmol, 1 equiv.), Ir[dF(CF_3_)ppy]_2_(dtbpy)PF_6_ (1.2 mg, 0.001 mmol, 1 mol%), LiBr (8.7 mg, 0.1 mmol, 1 equiv.), 2,4,6-collidine (18.2 mg (⁓20 µL), 0.15 mmol, 1.5 equiv.) and 1-allyl-2-methylbenzene (39.7 mg, 0.3 mmol, 3 equiv.). Dissolved in the mixture solvent of dioxane (0.5 mL) and THF (0.5 mL). After light irradiation with a 2 m blue LED strip at room temperature for 72 h, the crude product (15:85 *E*/*Z*) was purified by flash column chromatography to afford **64** as a yellow oil in 47% yield (only *Z* isomer, 24.8 mg; this result is based on two individual runs, however, it contains ⁓8% unidentified isomer as determined by ^1^H NMR).

**^1^H NMR (400 MHz, CDCl_3_, only *Z* isomer):** δ 7.95 (d, *J* = 7.9 Hz, 2H), 7.28 – 7.10 (m, 6H), 6.63 (d, *J* = 11.3 Hz, 1H), 5.88 (dt, *J* = 11.3, 7.4 Hz, 1H), 3.89 (s, 3H), 3.54 (d, *J* = 7.4 Hz, 2H), 2.28 (s, 3H).

**^13^C NMR (101 MHz, CDCl_3_, only *Z* isomer):** δ 167.2, 146.5, 136.5, 136.2, 130.1, 129.9, 129.6, 128.9, 128.5, 127.4, 125.6, 52.1, 34.6, 20.1.

IR (ATR) ν 2949, 1719, 1608, 1434, 1277, 1180, 1106, 1018, 966, 851, 754 cm^-1^;

HRMS (ESI) for C_18_H_18_O_2_Na^+^ (M+Na)^+^ : 289.11989; Found : 289.11990.

**Product 65**: the title compound was prepared following the general procedure using NiBr_2_ (8.8 mg, 0.04 mmol, 20 mol%), 6,6′-dimethyl-2,2′-bipyridine (7.4 mg, 0.04 mmol, 20 mol%), methyl 3-bromobenzoate (43 mg, 0.2 mmol, 1 equiv.), Ir[dF(CF_3_)ppy]_2_(dtbpy)PF_6_ (2.3 mg, 0.002 mmol, 1 mol%), 2,4,6-collidine (72.7 mg (⁓80 µL), 0.4 mmol, 2.0 equiv.) and 9-(2-methylallyl)-9*H*-carbazole (133 mg, 0.6 mmol, 3 equiv.). Dissolved in the mixture solvent of dioxane (1 mL) and THF (1 mL). After light irradiation with a 2 m blue LED strip at room temperature for 60 h, the crude product was purified by flash column chromatography to afford **65** as a colorless oil in 84% yield (23:77 *E*/*Z*, 60.1 mg).

**^1^H NMR (400 MHz, CDCl_3_,** **mixture of isomers E/Z, 23:77):** δ 8.27 – 8.03 (m, 3.54H_E+Z_), 7.91 (d, J = 7.9 Hz, 0.46H*_E_*), 7.60 – 7.45 (m, 3.54H*_E_*_+_*_Z_*), 7.42 (d, *J* = 8.2 Hz, 0.46H*_E_*), 7.39 – 7.23 (m, 3.54H*_E_*_+_*_Z_*), 7.09 (d, *J* = 8.0 Hz, 0.46H*_E_*), 6.72 (s, 0.77H*_Z_*), 6.62 (s, 0.23H*_E_*), 3.99 (s, 2.31H*_Z_*), 3.92 (s, 0.69H*_E_*), 3.72 (s, 1.54H*_Z_*), 3.40 (s, 0.46*_E_*), 1.95 (s, 0.69H*_E_*), 1.58 (s, 2.31H*_Z_*).

**^13^C NMR (101 MHz, CDCl_3_, mixture of isomers *E*/*Z*, 23:77):** ***E* isomer** (major) δ 167.1, 144.5, 140.6, 139.3, 130.1, 129.0, 128.8, 125.9, 123.2, 120.4, 120.1, 119.7, 110.0, 52.2, 43.0, 16.8; ***Z* isomer** (minor) 167.0, 143.9, 140.9, 140.2, 129.8, 129.0, 128.3, 126.1, 123.3, 120.5, 119.9, 119.2, 110.0, 52.1, 38.2, 19.6.

IR (ATR) ν 2922, 1713, 1604, 1478, 1451, 1277, 1237, 1177, 1107, 1016, 747 cm^-1^;

HRMS (ESI) for C_24_H_21_O_2_NNa^+^ (M+Na)^+^ : 378.14645; Found : 378.14653.

**Product 70**: the title compound was prepared following the general procedure using NiBr_2_ (4.4 mg, 0.02 mmol, 10 mol%), 4,4'-di-*tert*-butyl-2,2'-bipyridine (8.1 mg, 0.15 mmol, 15 mol%), methyl 4-bromobenzoate (43 mg, 0.2 mmol, 1 equiv.), Ir[dF(CF_3_)ppy]_2_(dtbpy)PF_6_ (2.3 mg, 0.002 mmol, 1 mol%), 2,6-lutidine (32.1 mg (⁓35 µL), 0.3 mmol, 1.5 equiv.) and 2-methylprop-1-en-1-yl acetate (68.5 mg, 0.6 mmol, 3 equiv.). Dissolved in the benzene (2 mL). After light irradiation with a 2 m blue LED strip at room temperature for 60 h, the crude product was purified by flash column chromatography to afford **70** as a colorless oil in 74% yield (77:23 *E*/*Z*, 36.9 mg).

**^1^H NMR (600 MHz, CDCl_3_, mixture of isomers *E*/*Z*, 77:23):** ***E* isomer** (major) δ 7.96 (d, *J* = 8.4 Hz, 2H), 7.25 (d, *J* = 7.8 Hz, 2H), 7.07 (d, *J* = 1.4 Hz, 1H), 3.90 (s, 3H), 3.32 (s, 2H), 2.15 (s, 3H), 1.58 (d, *J* = 1.5 Hz, 3H); ***Z* isomer** (minor) 7.02 (d, *J* = 1.7 Hz, 1H), 3.51 (s, 2H), 2.16 (s, 3H), 1.56 (d, *J* = 1.6 Hz, 3H) the remaining resonances are insufficiently resolved from those of the ***E* isomer** to be reported.

**^13^C NMR (151 MHz, CDCl_3_, mixture of isomers *E*/*Z*, 77:23):** ***E* isomer** (major) δ 168.3, 167.2, 144.6, 131.9, 129.9, 128.9, 128.6, 120.4, 52.2, 40.5, 20.9, 13.7; ***Z* isomer** (minor) 144.8, 131.2, 129.9, 128.8, 128.4, 120.0, 36.0, 29.9, 17.4 the remaining resonances are insufficiently resolved from those of the ***E* isomer** to be reported.

IR (ATR) ν 1749, 1720, 1610, 1435, 1371, 1278, 1216, 1100, 1020, 917, 826 cm^-1^;

HRMS (ESI) for C_14_H_16_O_4_Na^+^ (M+Na)^+^: 271.09408; Found : 271.09328.

**Product 72**: the title compound was prepared following the general procedure using NiBr_2_ (4.4 mg, 0.02 mmol, 10 mol%), 4,4'-di-*tert*-butyl-2,2'-bipyridine (8.1 mg, 0.15 mmol, 15 mol%), methyl 4-bromobenzoate (43 mg, 0.2 mmol, 1 equiv.), Ir[dF(CF_3_)ppy]_2_(dtbpy)PF_6_ (2.3 mg, 0.002 mmol, 1 mol%), 2,6-lutidine (32.1 mg (⁓35 µL), 0.3 mmol, 1.5 equiv.) and (*Z*)-tert-butyldimethyl(prop-1-en-1-yloxy)silane (103 mg, 0.6 mmol, 3 equiv.). Dissolved in the mixture solvent of benzene (1 mL) and THF (1 mL). After light irradiation with a 2 m blue LED strip at room temperature for 48 h, the crude product was purified by flash column chromatography to afford **72** as a colorless oil in 58% yield (35:65 *E*/*Z*, 35.6 mg).

**^1^H NMR (400 MHz, CDCl_3_, mixture of isomers *E*/*Z*, 35:65):** δ ***Z* isomer** (major) 7.92 (d, *J* = 8.2 Hz, 2H), 7.26 (d, *J* = 8.2 Hz, 2H), 6.31 (dt, *J* = 6.0, 1.5 Hz, 1H), 4.64 (td, *J* = 7.4, 5.7 Hz, 1H), 3.88 (s, 3H), 3.47 (d, *J* = 7.4 Hz, 2H), 0.92 (s, 9H), 0.14 (s, 6H); ***E* isomer** (minor) 5.12 (dt, *J* = 11.9, 7.6 Hz, 1H), 3.88 (s, 3H), 3.26 (d, *J* = 7.4 Hz, 2H), 0.91 (s, 9H), 0.13 (s, 6H), the remaining resonances are insufficiently resolved from those of the ***E* isomer** to be reported.

**^13^C NMR (101 MHz, CDCl_3_, mixture of isomers *E*/*Z*, 35:65):** δ ***Z* isomer** (major) 167.4, 147.7, 139.9, 129.8, 128.5, 127.7, 108.0, 52.1, 30.2, 25.8, 18.4, -5.2; ***E* isomer** (minor) 167.3, 147.2, 142.1, 129.8, 128.4, 128.0, 109.3, 52.1, 33.8, 25.8, 18.5, -5.1.

IR (ATR) ν 2951, 2932, 2857, 1722, 1656, 1465, 1434, 1277, 1175, 1107, 1018, 837, 782 cm^-1^;

HRMS (ESI) for C_17_H_26_O_3_NaSi^+^ (M+Na)^+^ : 329.15434; Found : 329.15333.

**Product 75**: the title compound was prepared following the general procedure using NiBr_2_ (4.4 mg, 0.02 mmol, 10 mol%), 4,4'-di-*tert*-butyl-2,2'-bipyridine (8.1 mg, 0.15 mmol, 15 mol%), 1-bromo-4-(*tert*-butyl)benzene (42.6 mg, 0.2 mmol, 1 equiv.), Ir[dF(CF_3_)ppy]_2_(dtbpy)PF_6_ (2.3 mg, 0.002 mmol, 1 mol%), 2,6-lutidine (32.1 mg (⁓35 µL), 0.3 mmol, 1.5 equiv.) and *tert*-butyl((3-(4-(tert-butyl)phenyl)-2-methylprop-1-en-1-yl)oxy)dimethylsilane (191 mg, mixture of *E*/*Z* 1:1, 0.6 mmol, 3 equiv.). Dissolved in the mixture solvent of benzene (1 mL) and THF (1 mL). After light irradiation with a 2 m blue LED strip at room temperature for 60 h, the crude product was purified by flash column chromatography to afford **75** as a colorless oil in 43% yield (38.5 mg).

**^1^H NMR (400 MHz, CDCl_3_):** ***Z* isomer** (major) δ 7.30 (t, *J* = 7.8 Hz, 4H), 7.11 (dd, *J* = 10.6, 8.0 Hz, 4H), 6.32 (s, 1H), 3.29 (s, 2H), 3.06 (s, 2H), 1.33 (s, 18H), 0.97 (s, 9H), 0.19 (s, 6H).

**^13^C NMR (101 MHz, CDCl_3_):** δ ***Z* isomer** (major) 148.6, 148.2, 137.9, 137.5, 135.8, 128.6, 128.5, 125.0, 125.0, 120.1, 36.6, 34.3, 34.3, 31.6, 31.5, 25.7, 18.2, -5.2.

IR (ATR) ν 2957, 2861, 1666, 1511, 1466, 1363, 1256, 1193, 1138, 1015, 838, 779 cm^-1^;

HRMS (EI) for C_30_H_46_OSi^+^ (M)^+^ : 450.33125; Found : 450.33117.

**Product 76**: the title compound was prepared following the general procedure using NiBr_2_ (4.4 mg, 0.02 mmol, 10 mol%), 4,4'-di-*tert*-butyl-2,2'-bipyridine (8.1 mg, 0.15 mmol, 15 mol%), 1-bromo-4-(*tert*-butyl)benzene (42.6 mg, 0.2 mmol, 1 equiv.), Ir[dF(CF_3_)ppy]_2_(dtbpy)PF_6_ (2.3 mg, 0.002 mmol, 1 mol%), 2,6-lutidine (32.1 mg (⁓35 µL), 0.3 mmol, 1.5 equiv.) and *tert*-butyl((3-(4-(tert-butyl)phenyl)-2-methylprop-1-en-1-yl)oxy)dimethylsilane (191 mg, mixture of *E*/*Z* 1:1, 0.6 mmol, 3 equiv.). Dissolved in the benzene (2 mL). After light irradiation with a 2 m blue LED strip at room temperature for 60 h, the crude product was purified by flash column chromatography to afford **76** as a colorless oil in 51% yield (46 mg).

**^1^H NMR (400 MHz, CDCl_3_, mixture of isomers *E*/*Z*, 85:15):** ***E* isomer** (major) δ 7.93 (d, *J* = 8.1 Hz, 2H), 7.26 (d, *J* = 8.2 Hz, 2H), 7.18 (d, *J* = 8.0 Hz, 2H), 7.04 (d, *J* = 8.1 Hz, 2H), 6.31 (s, 1H), 3.91 (s, 3H), 3.26 (s, 2H), 3.13 (s, 2H), 1.31 (s, 9H), 0.96 (s, 9H), 0.18 (s, 6H); ***Z* isomer** (minor) 6.35 (s, 1H), 3.36 (s, 2H), 3.04 (s, 2H), 0.95 (s, 9H) the remaining resonances are insufficiently resolved from those of the ***E* isomer** to be reported.

**^13^C NMR (101 MHz, CDCl_3_, mixture of isomers *E*/*Z*, 85:15):** ***E* isomer** (major) δ 167.3, 148.6, 146.4, 137.5, 136.7, 129.7, 129.1, 128.7, 128.0, 125.2, 119.5, 52.1, 37.5, 34.5, 31.9, 31.6, 25.9, 18.4, -5.0.

IR (ATR) ν 2955, 2859, 1723, 1666, 1609, 1465, 1434, 1364, 1275, 1191, 1139, 1108, 1018, 837, 781 cm^-1^; HRMS (ESI) for C_28_H_40_O_3_NaSi^+^ (M+Na)^+^ : 475.26389; Found : 475.26324.

**Product 77**: the title compound was prepared following the general procedure using NiBr_2_ (8.8 mg, 0.04 mmol, 20 mol%), 6,6′-dimethyl-2,2′-bipyridine (7.4 mg, 0.04 mmol, 20 mol%), 1-bromo-4-(*tert*-butyl)benzene (42.6 mg, 0.2 mmol, 1 equiv.), Ir[dF(CF_3_)ppy]_2_(dtbpy)PF_6_ (2.3 mg, 0.002 mmol, 1 mol%), 2,4,6-collidine (72.7 mg (⁓80 µL), 0.4 mmol, 2.0 equiv.) and *tert*-butyl((3-(4-(tert-butyl)phenyl)-2-methylprop-1-en-1-yl)oxy)dimethylsilane (191 mg, mixture of *E*/*Z* 1:1, 0.6 mmol, 3 equiv.). Dissolved in the dioxane (2 mL). After light irradiation with a 2 m blue LED strip at room temperature for 60 h, the crude product was purified by flash column chromatography to afford **77** as a colorless oil in 57% yield (51.5 mg).

**^1^H NMR (400 MHz, CDCl_3_, mixture of isomers *E*/*Z*, 18:82):** ***Z* isomer** (major) δ 7.91 (d, *J* = 8.1 Hz, 2H), 7.27 (d, *J* = 8.2 Hz, 2H), 7.20 (d, *J* = 8.2 Hz, 2H), 7.03 (d, *J* = 8.2 Hz, 2H), 6.35 (s, 1H), 3.89 (s, 3H), 3.36 (s, 2H), 3.04 (s, 2H), 1.31 (s, 9H), 0.95 (s, 9H), 0.19 (s, 6H); ***E* isomer** (minor) 6.32 (s, 1H), 3.26 (s, 2H), 3.13 (s, 2H), 0.95 (s, 9H) the remaining resonances are insufficiently resolved from those of the ***Z* isomer** to be reported.

**^13^C NMR (101 MHz, CDCl_3_, mixture of isomers *E*/*Z*, 18:82):** ***Z* isomer** (major) δ 167.4, 149.0, 146.9, 137.1, 136.7, 129.6, 129.0, 128.6, 127.7, 125.2, 119.0, 52.1, 52.0, 36.9, 34.5, 32.5, 31.6, 25.8, 18.4, -5.0.

IR (ATR) ν 2955, 2859, 1722, 1665, 1609, 1465, 1434, 1363, 1275, 1192, 1139, 1108, 1018, 838 cm^-1^;

HRMS (ESI) for C_28_H_40_O_3_NaSi^+^ (M+Na)^+^ : 475.26389; Found : 475.26227.

**Product 78**:^14^ the title compound was prepared following the general procedure **E** using NiCl_2_ dtbbpy•4H_2_O (4.7 mg, 0.01 mmol, 10 mol%), 1-(4-bromophenyl)ethan-1-one (19.9 mg, 0.1 mmol, 1 equiv.), Ir[dF(CF_3_)ppy]_2_(dtbpy)PF_6_ (1.1 mg, 0.001 mmol, 1 mol%), 2,6-lutidine (16.1 mg (⁓17 µL), 0.15 mmol, 1.5 equiv.) and *tert*-butyldimethyl((2-methyl-1-phenylprop-1-en-1-yl)oxy)silane (131 mg, 0.5 mmol, 5 equiv.). Dissolved in the mixture solvent of benzene (2 mL). After light irradiation with a 2 m blue LED strip at room temperature for 40 h. The crude product was next subjected to HCl (2M) and the process of deprotection was monitored by TLC. The mixture was purified by flash column chromatography to afford **78** as a white solid in 41% yield (11.0 mg).

**^1^H NMR (600 MHz, CDCl_3_):** δ 7.91 (d, *J* = 7.9 Hz, 2H), 7.86 (d, *J* = 8.1 Hz, 2H), 7.55 (t, *J* = 7.4 Hz, 1H), 7.45 (t, *J* = 7.7 Hz, 2H), 7.29 (d, *J* = 7.7 Hz, 2H), 3.78 (h, *J* = 7.0 Hz, 1H), 3.23 (dd, *J* = 13.6, 6.7 Hz, 1H), 2.78 (dd, *J* = 13.8, 7.3 Hz, 1H), 2.56 (s, 3H), 1.22 (d, *J* = 6.7 Hz, 3H).

**^13^C NMR (151 MHz, CDCl_3_):** δ 203.1, 197.7, 145.8, 136.2, 135.4, 133.1, 129.3, 128.7, 128.5, 128.2, 42.4, 39.2, 26.5, 17.7.

**Product 80**: the title compound was prepared following the general procedure **E** using NiCl_2_ dtbbpy•4H_2_O (9.4 mg, 0.02 mmol, 10 mol%), 1-(4-bromophenyl)ethan-1-one (39.8 mg, 0.2 mmol, 1 equiv.), Ir[dF(CF_3_)ppy]_2_(dtbpy)PF_6_ (2.3 mg, 0.002 mmol, 1 mol%), 2,6-lutidine (32.1 mg (⁓35 µL), 0.3 mmol, 1.5 equiv.) and trimethyl((2-methylprop-1-en-1-yl)oxy)silane (86.6 mg, 0.6 mmol, 3 equiv.). Dissolved in the mixture solvent of benzene (2 mL). After light irradiation with a 2 m blue LED strip at room temperature for 48 h. The crude product was next subjected to HCl (2M) and the process of deprotection was monitored by TLC. The mixture was purified by flash column chromatography to afford **80** as a white solid in 43% yield (17.7 mg).

**^1^H NMR (400 MHz, CDCl_3_):** δ 7.89 (d, *J* = 7.9 Hz, 2H), 7.28 (d, *J* = 8.1 Hz, 2H), 3.12 (dd, *J* = 12.8, 6.2 Hz, 1H), 2.87 – 2.70 (m, 2H), 2.59 (s, 3H), 1.20 (d, *J* = 6.6 Hz, 3H).

**^13^C NMR (101 MHz, CDCl_3_):** δ 198.0, 181.4, 144.9, 135.7, 129.4, 128.7, 41.0, 39.3, 26.7, 16.8.

HRMS (ESI) for C_12_H_14_O_3_Na^+^ (M+Na)^+^ : 229.08352; Found : 229.08381.

**Product 81**: the title compound was prepared following the general procedure of Simmon-Smith cyclopropanation using (E)-tert-butyl((3-(4-(tert-butyl)phenyl)-2-methylprop-1-en-1-yl)oxy)dimethylsilane. The mixture was purified by flash column chromatography to afford **81** as a white solid in 91% yield (60.5 mg, 99:1 dr).

**^1^H NMR (400 MHz, CDCl_3_):** δ 7.32 (d, *J* = 8.3 Hz, 2H), 7.13 (d, *J* = 8.1 Hz, 2H), 3.28 (dd, *J* = 6.6, 3.3 Hz, 1H), 2.56 (d, *J* = 14.2 Hz, 1H), 2.39 (d, *J* = 14.2 Hz, 1H), 1.34 (s, 9H), 1.09 (s, 3H), 0.92 (s, 9H), 0.67 (t, *J* = 6.1 Hz, 1H), 0.30 (dd, *J* = 5.6, 3.3 Hz, 1H), 0.10 (s, 3H), 0.07 (s, 3H).

**^13^C NMR (101 MHz, CDCl_3_):** δ 148.8, 136.9, 129.0, 125.1, 57.2, 43.8, 34.5, 31.6, 26.1, 21.6, 20.2, 18.3, 16.6, -4.8, -4.9.

IR (ATR) ν 2956, 2859, 1789, 1513, 1464, 1367, 1253, 1155, 1091, 1009, 895, 834, 776 cm^-1^;

HRMS (ESI) for C_21_H_36_OSiNa^+^ (M+Na)^+^ : 355.24276; Found : 355.24254.

**Product 82**: the title compound was prepared following the general procedure of Simmon-Smith cyclopropanation using (Z)-tert-butyl((3-(4-(tert-butyl)phenyl)-2-methylprop-1-en-1-yl)oxy)dimethylsilane. The mixture was purified by flash column chromatography to afford **82** as a white solid in 98% yield (65.2 mg, only one isomer).

**^1^H NMR (600 MHz, CDCl_3_):** δ 7.33 (d, *J* = 8.2 Hz, 2H), 7.27 (d, *J* = 8.2 Hz, 2H), 3.21 (dd, *J* = 5.6, 3.6 Hz, 1H), 2.80 – 2.68 (m, 2H), 1.35 (s, 9H), 0.96 (s, 9H), 0.87 (s, 3H), 0.55 – 0.50 (m, 2H), 0.18 (s, 3H), 0.16 (s, 3H).

**^13^C NMR (151 MHz, CDCl_3_):** δ 148.4, 138.2, 129.2, 125.0, 57.9, 37.9, 34.5, 31.6, 26.0, 22.2, 21.7, 21.2, 18.3, -4.7, -4.8.

IR (ATR) ν 2955, 2860, 2323, 1795, 1513, 1463, 1369, 1253, 1186, 1146, 893, 837, 776 cm^-1^;

HRMS (ESI) for C_21_H_36_OSiNa^+^ (M+Na)^+^ : 355.24276; Found : 355.24206.


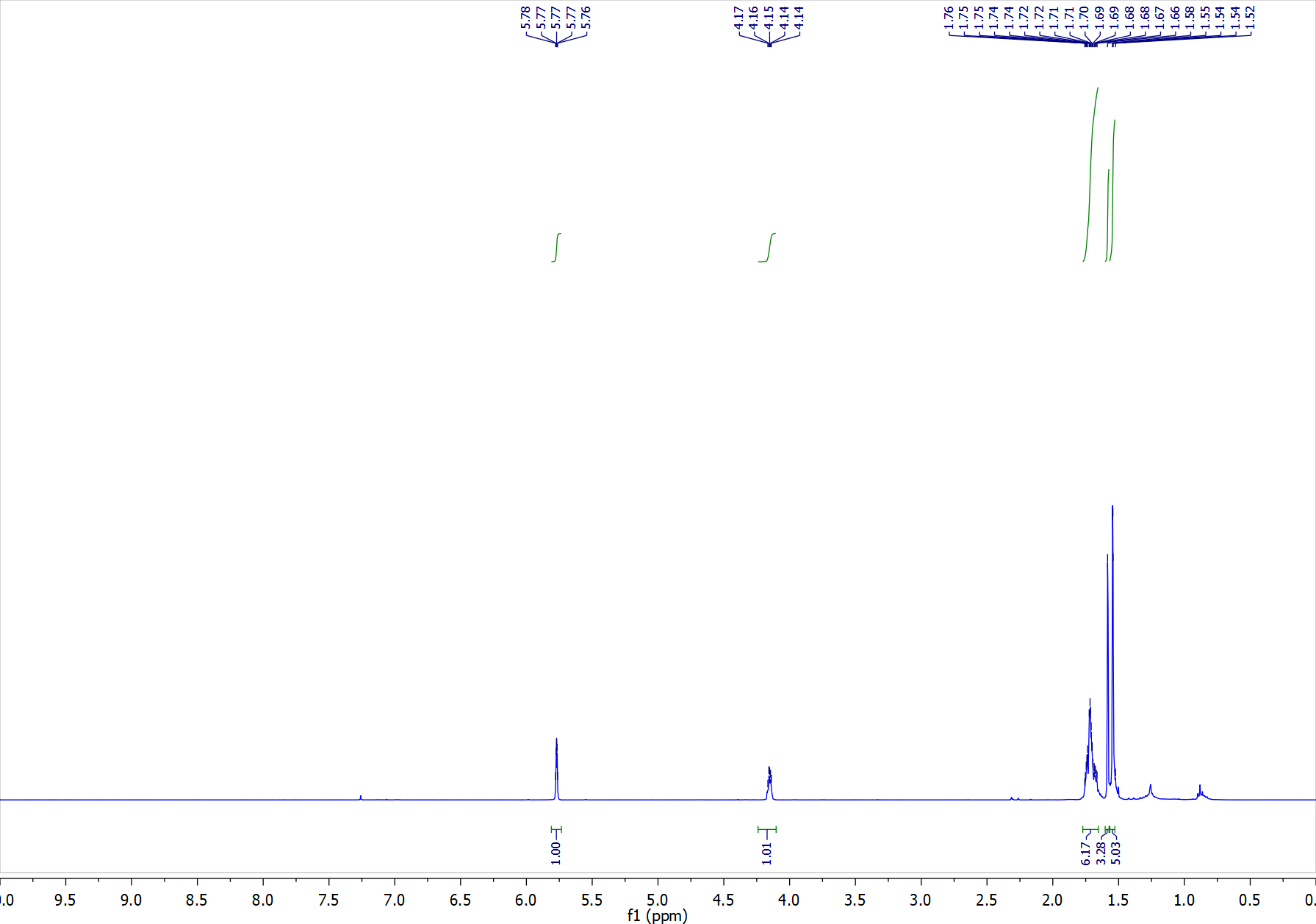


**Supplementary Figure 25.** ^1^H NMR spectrum of **compound 3**


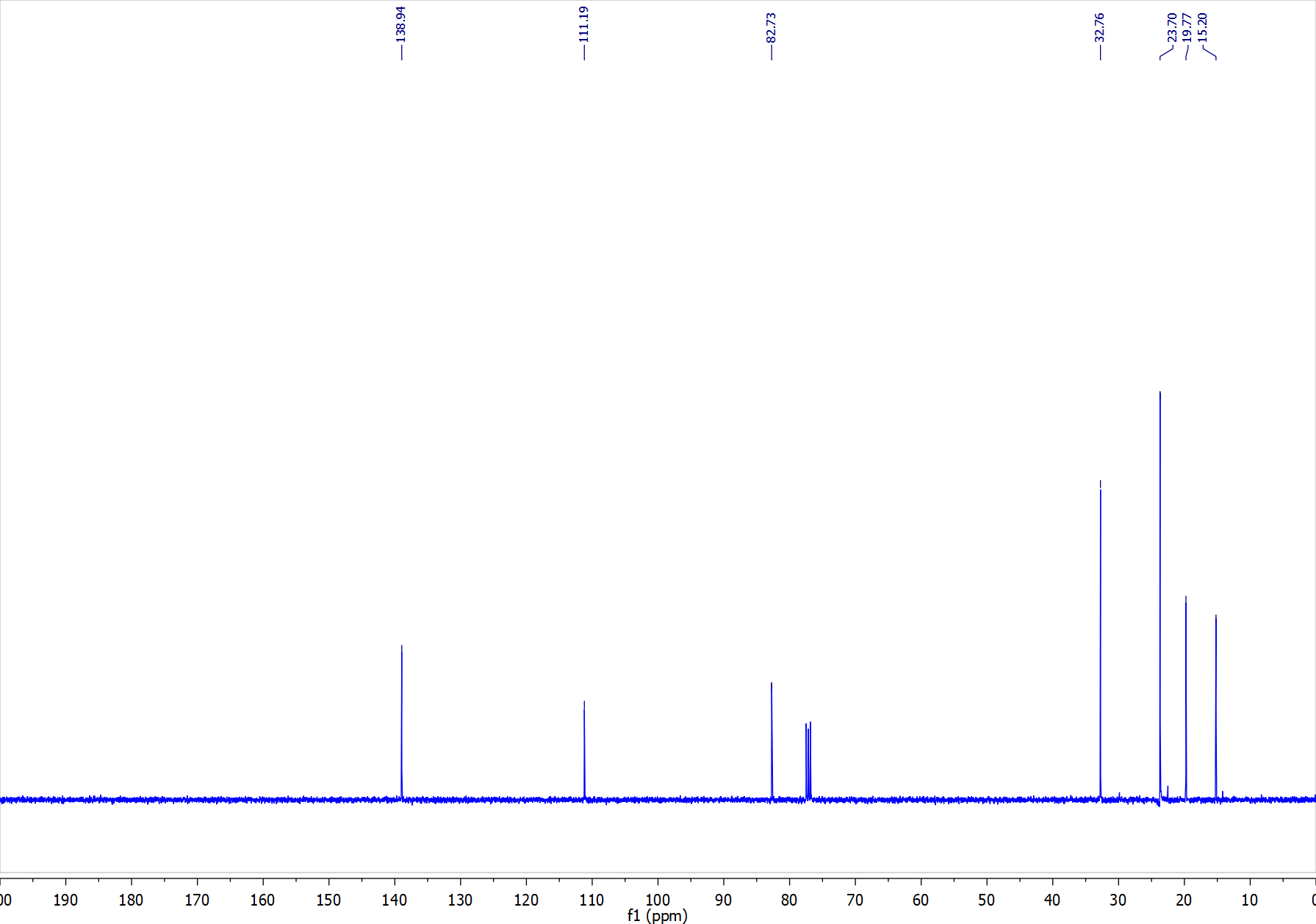
 **Supplementary Figure 26.** ^13^C NMR spectrum of **compound 3**


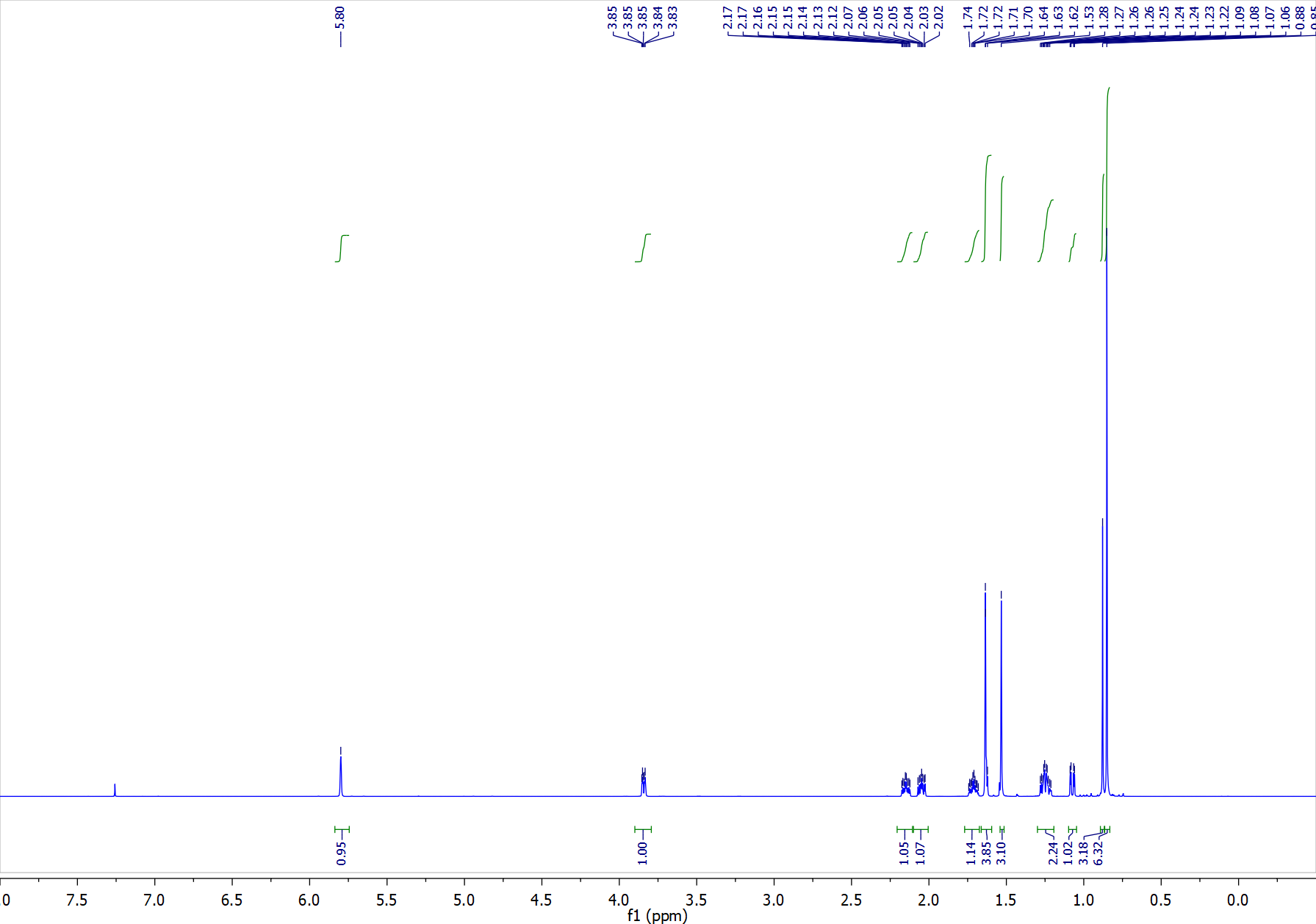
 **Supplementary Figure 27.** ^1^H NMR spectrum of **compound 5**


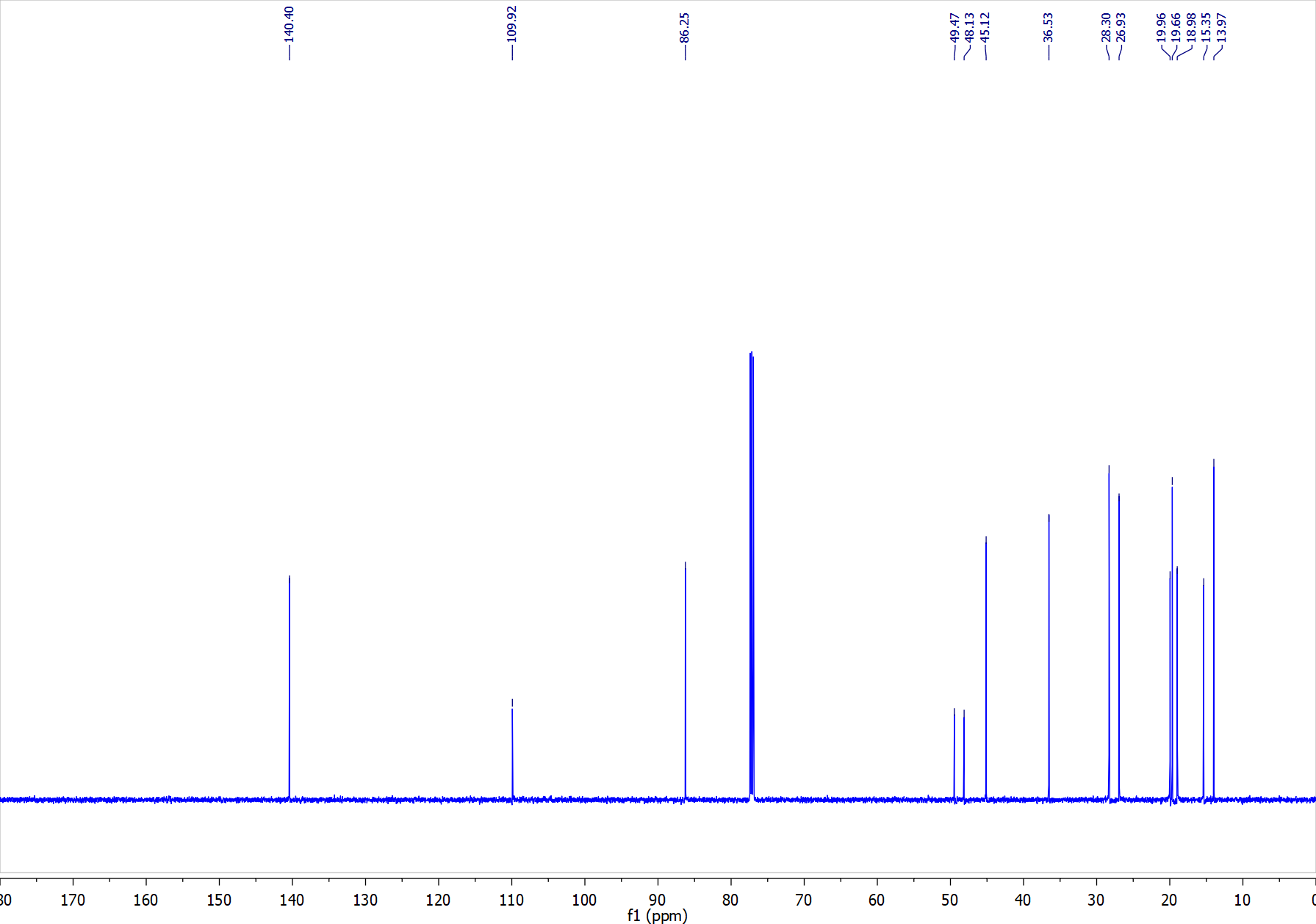
**Supplementary Figure 28.** ^13^C NMR spectrum of **compound 5**


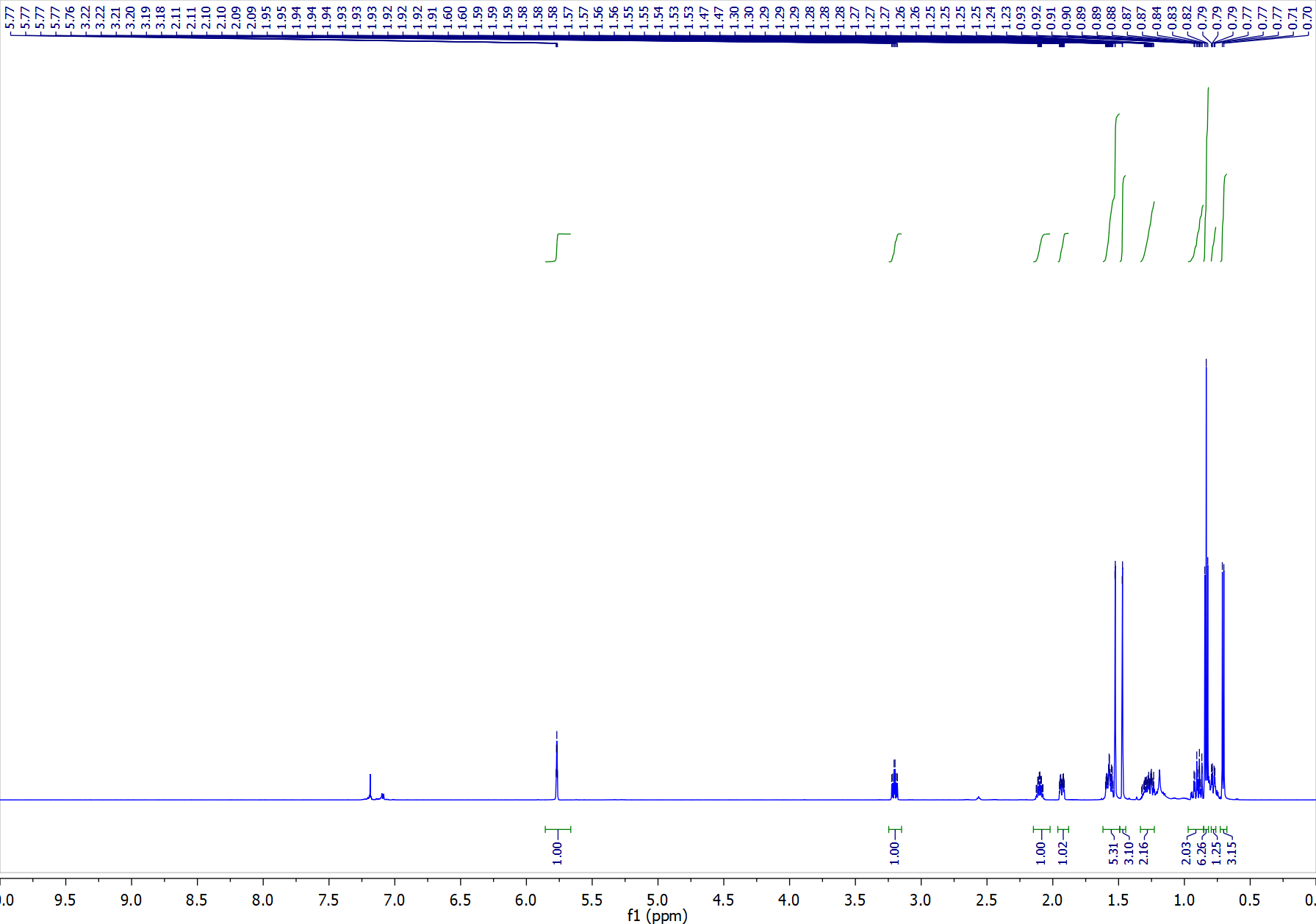
 **Supplementary Figure 29.** ^1^H NMR spectrum of **compound 7**


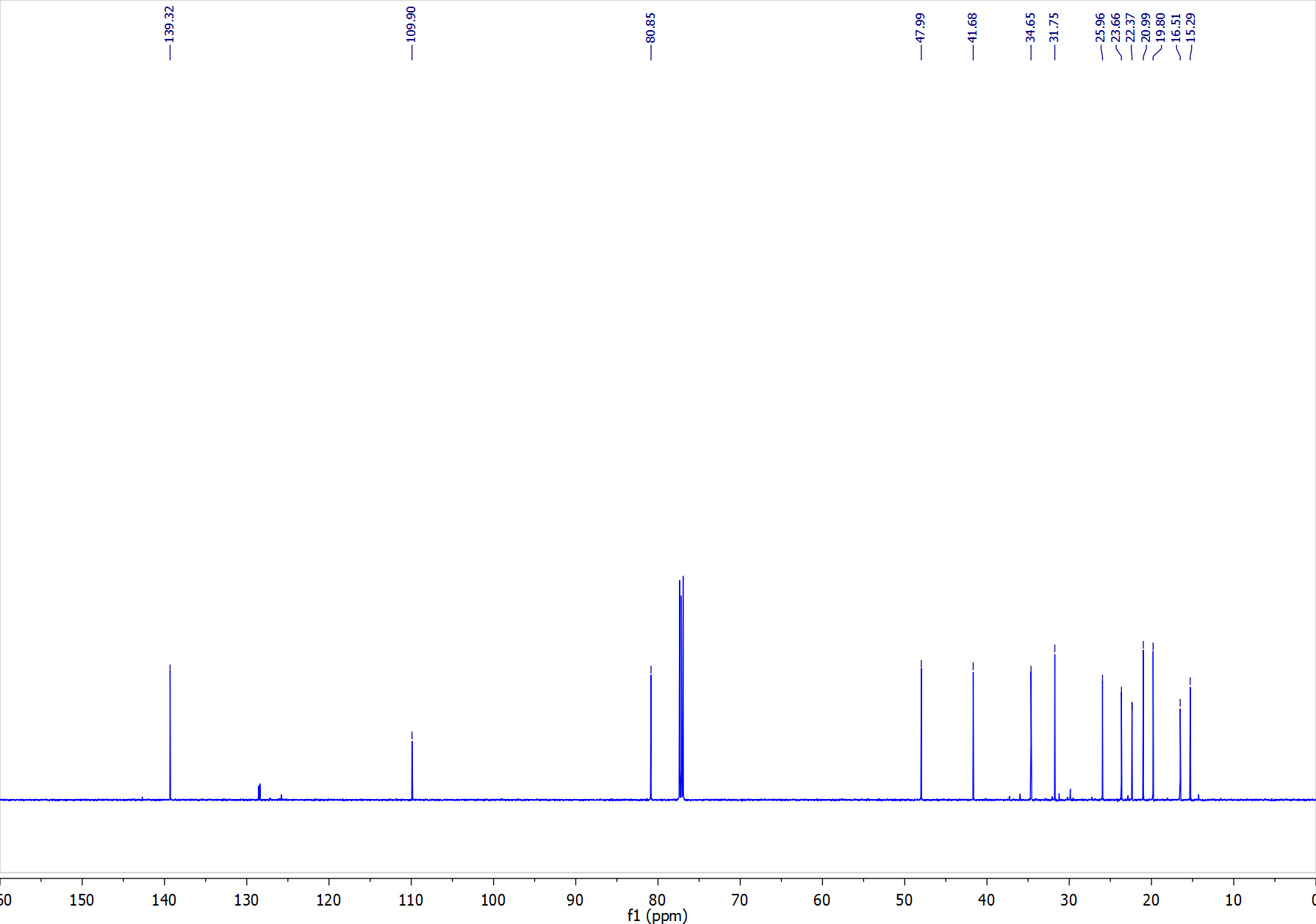


**Supplementary Figure 30.** ^13^C NMR spectrum of **compound 7**


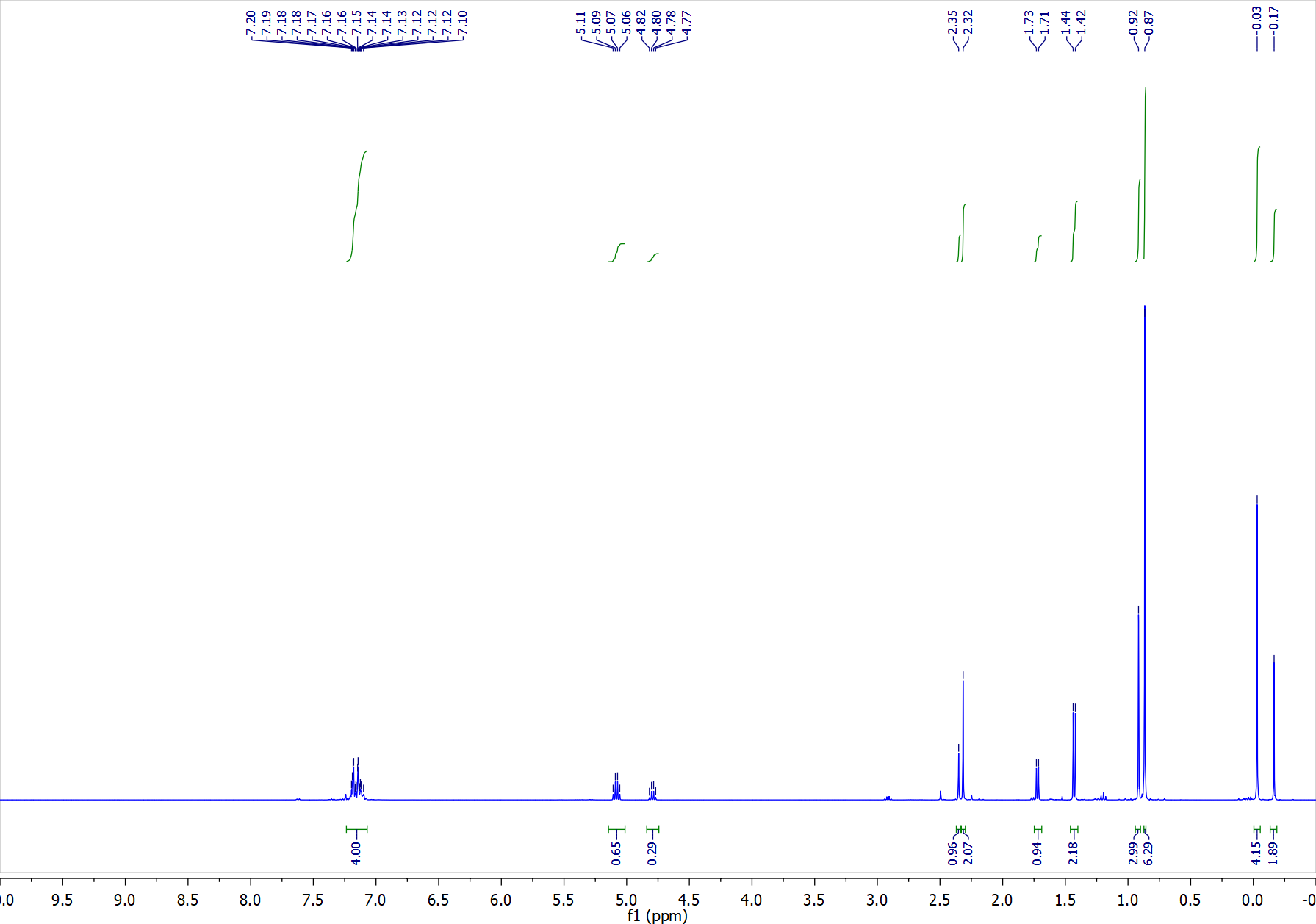
 **Supplementary Figure 31.** ^1^H NMR spectrum of **compound 11**


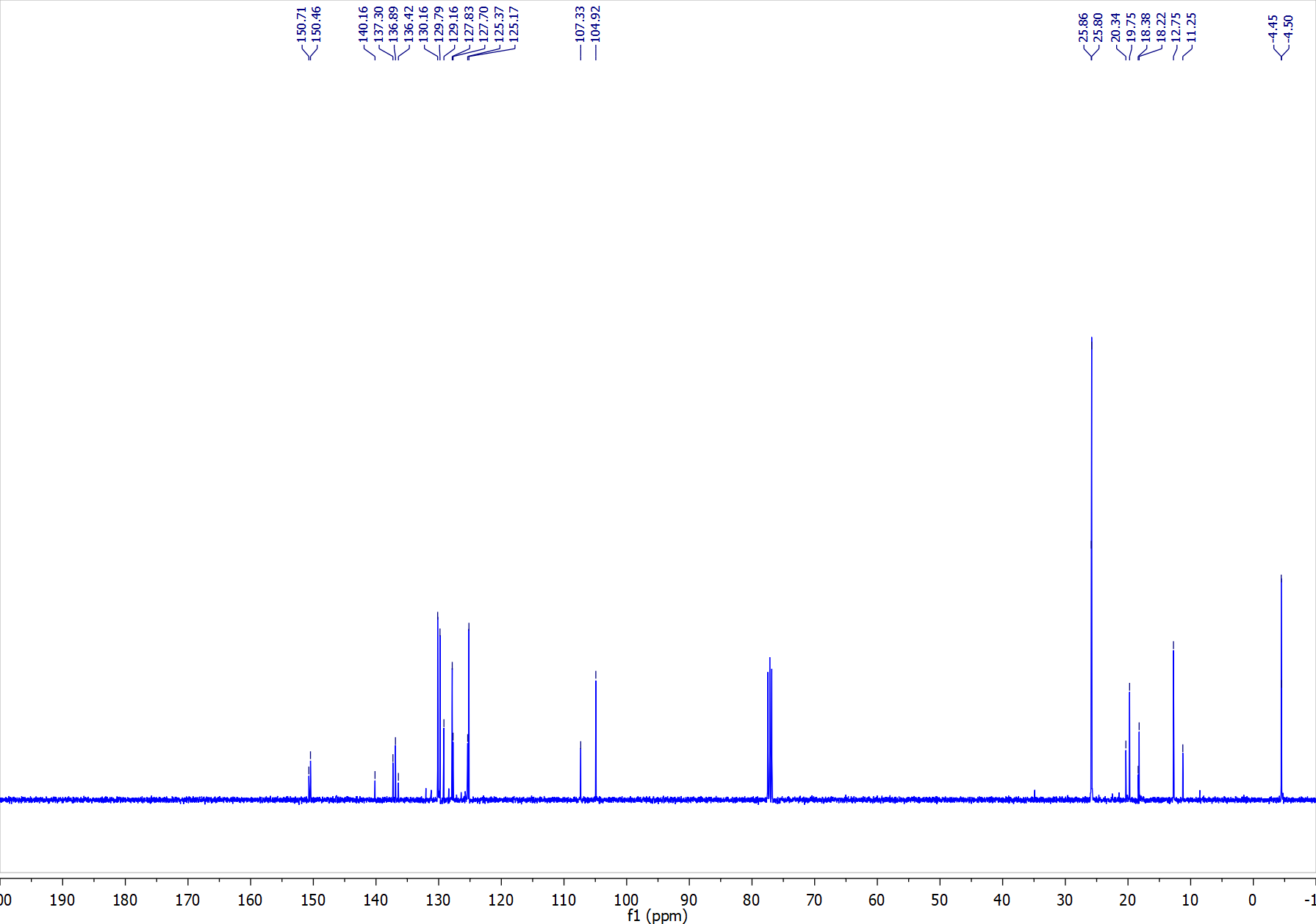
**Supplementary Figure 32.** ^13^C NMR spectrum of **compound 11**


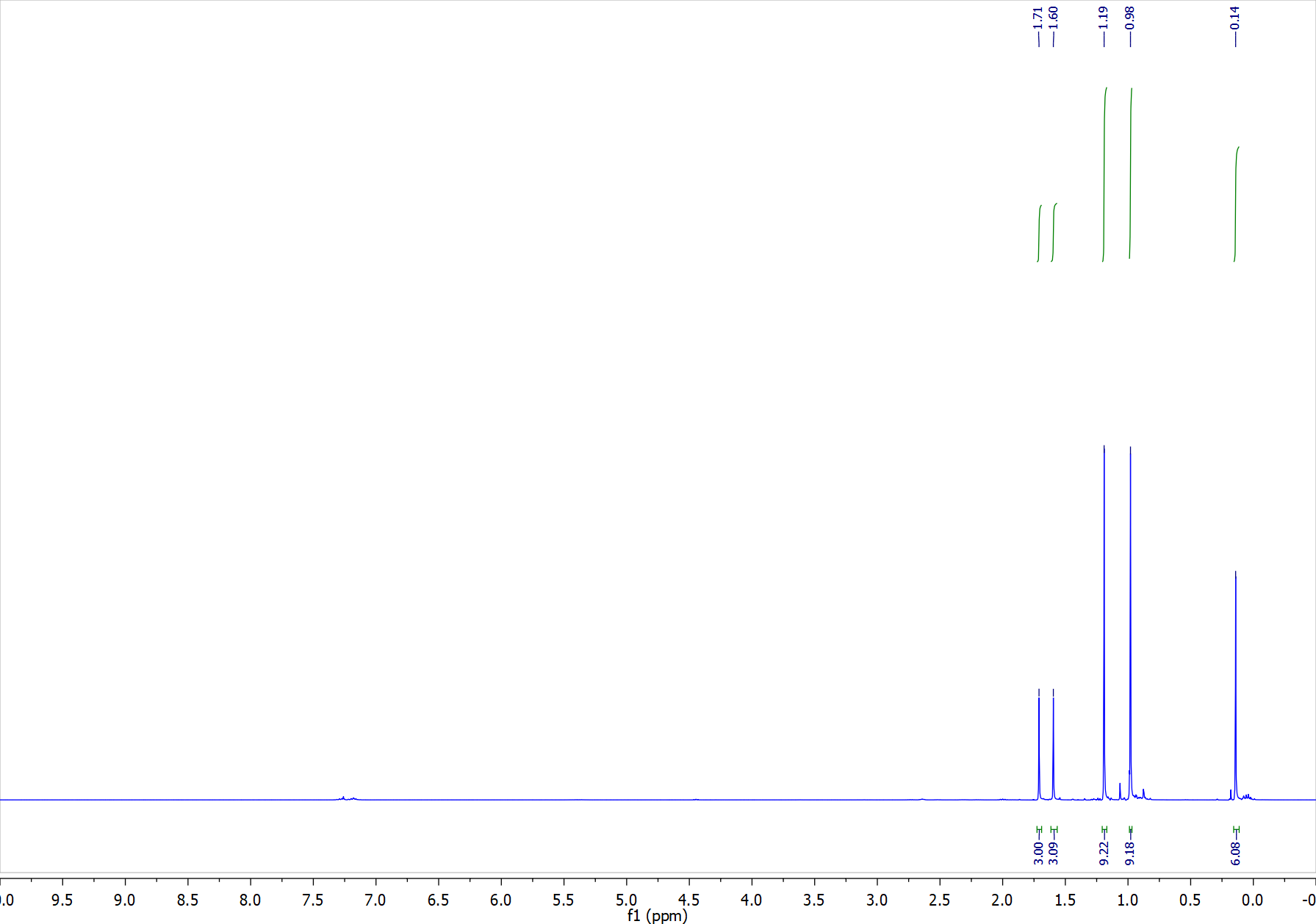
 **Supplementary Figure 33.** ^1^H NMR spectrum of **compound 12**


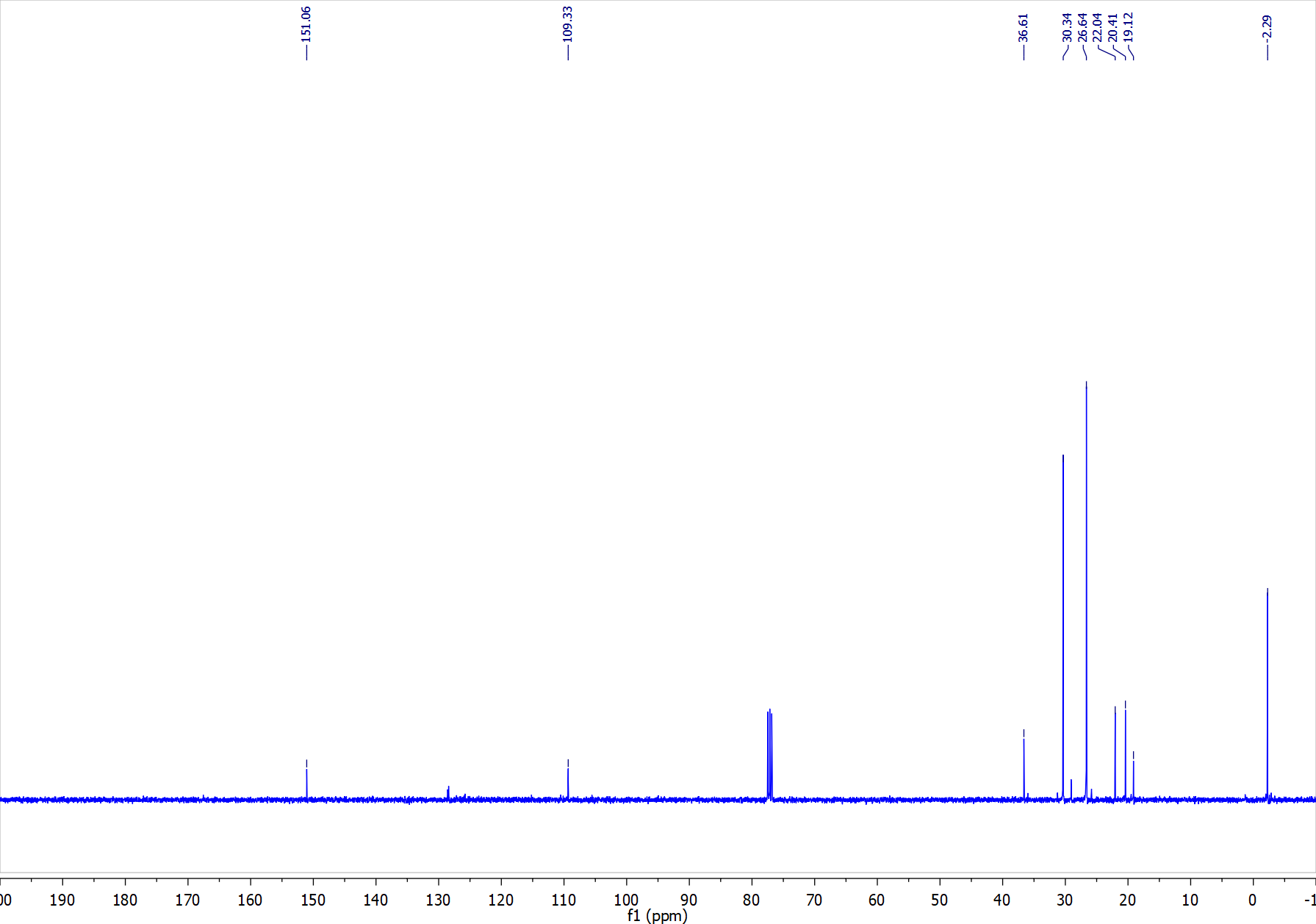
**Supplementary Figure 34.** ^13^C NMR spectrum of **compound 12**


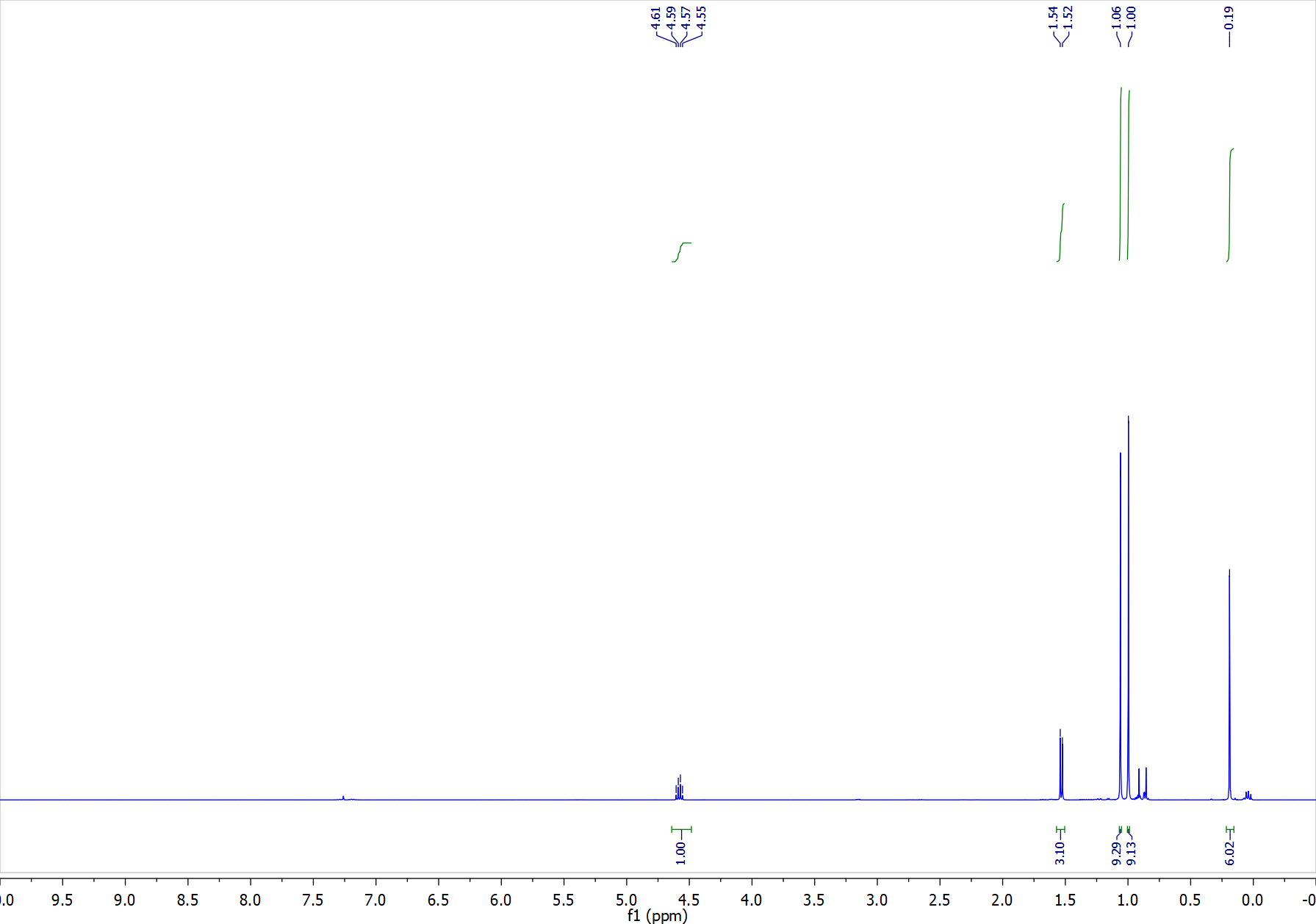
 **Supplementary Figure 35.** ^1^H NMR spectrum of **compound 13**


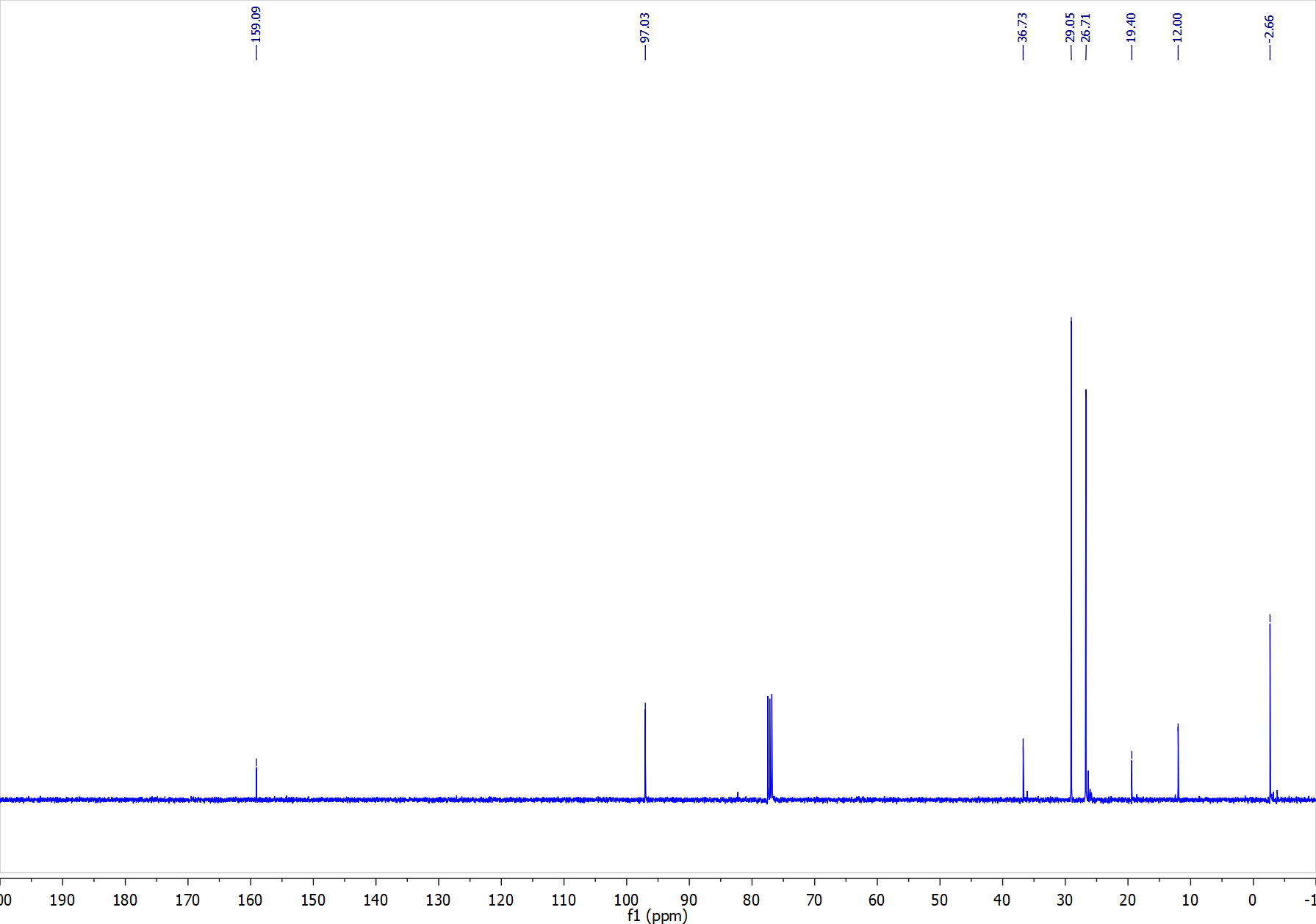
**Supplementary Figure 36.** ^13^C NMR spectrum of **compound 13**


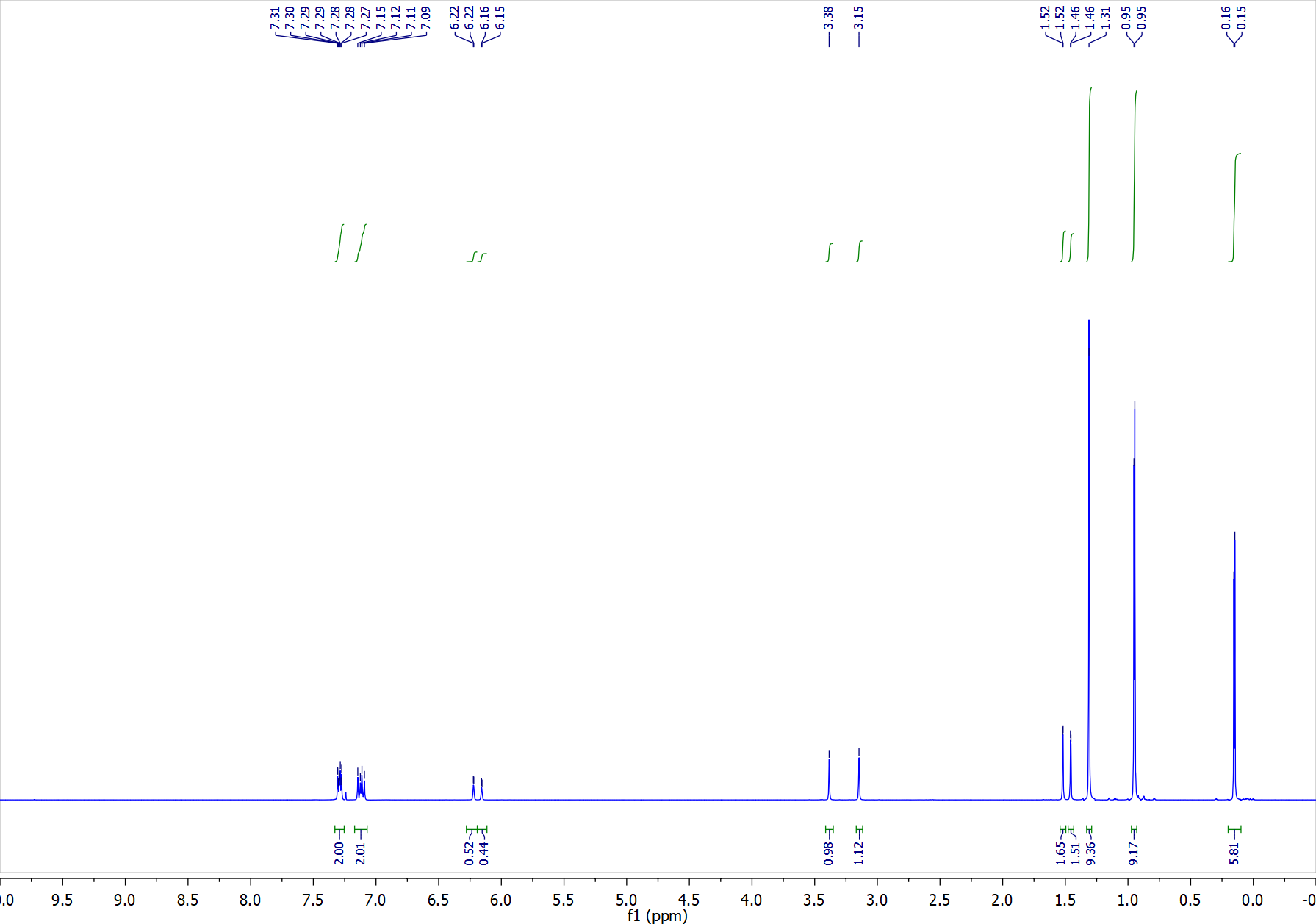
**Supplementary Figure 37.** ^1^H NMR spectrum of **compound 23**


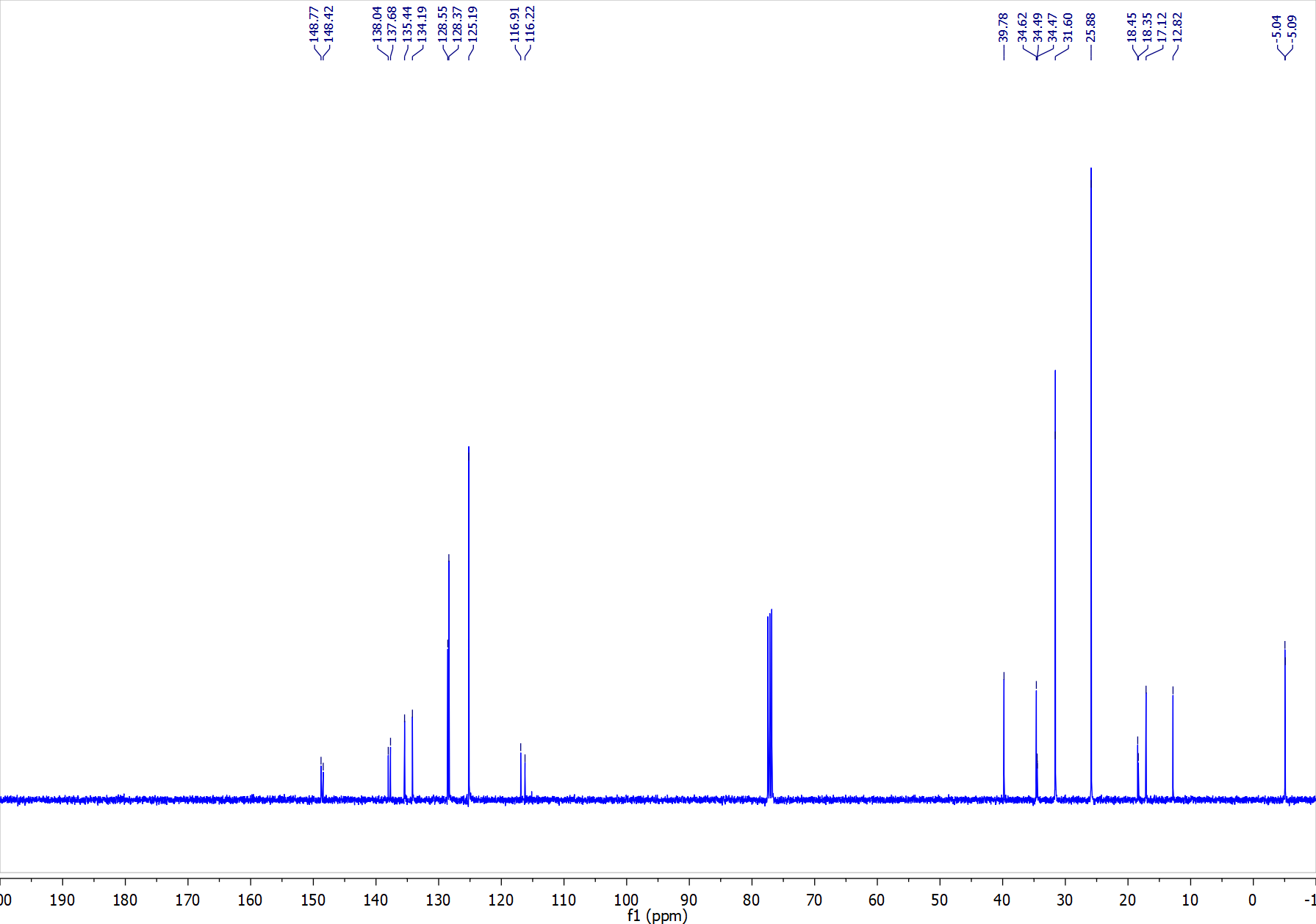


**Supplementary Figure 38.** ^13^C NMR spectrum of **compound 23**


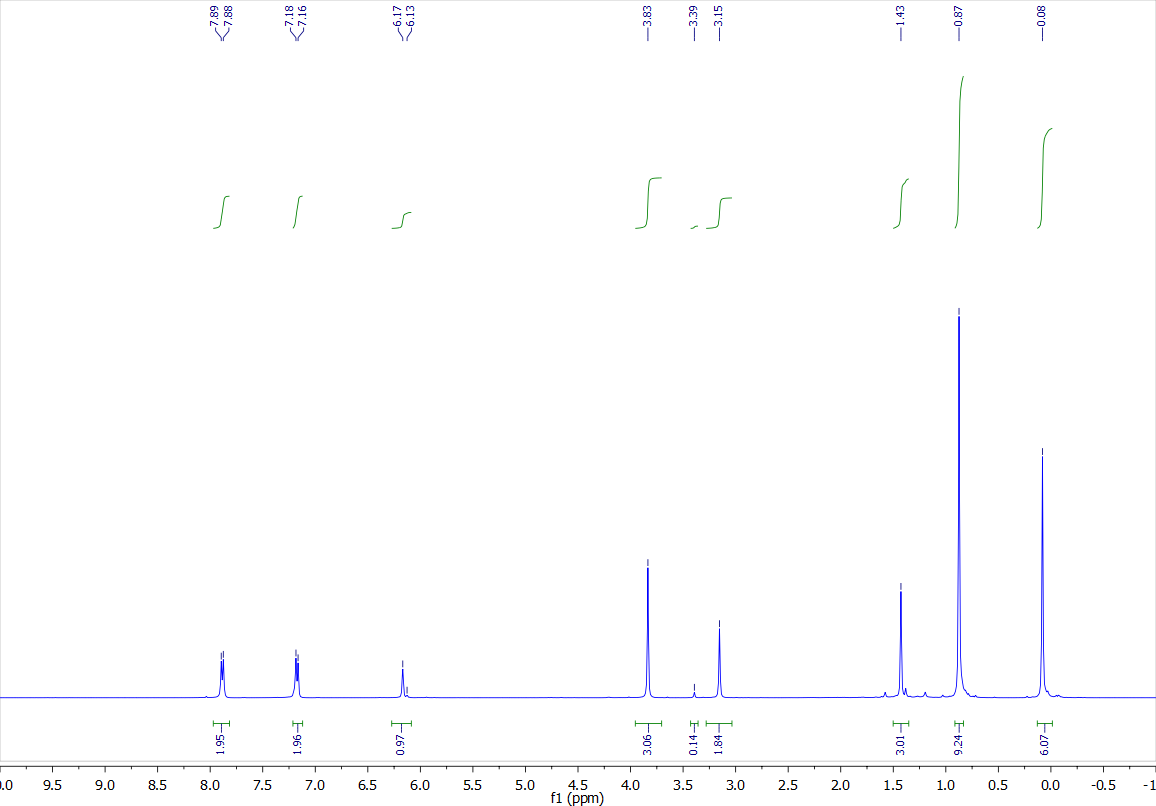
 **Supplementary Figure 39.** ^1^H NMR spectrum of **3**


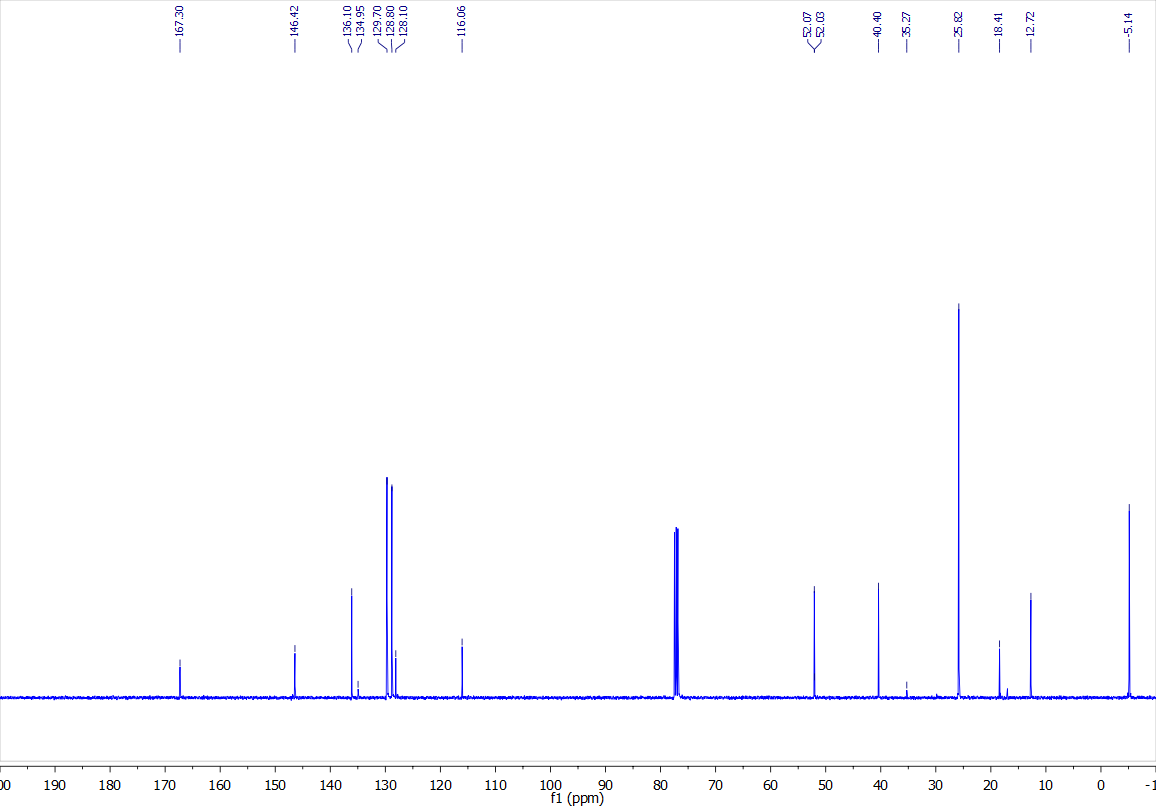
 **Supplementary Figure 40.** ^13^C NMR spectrum of **3**


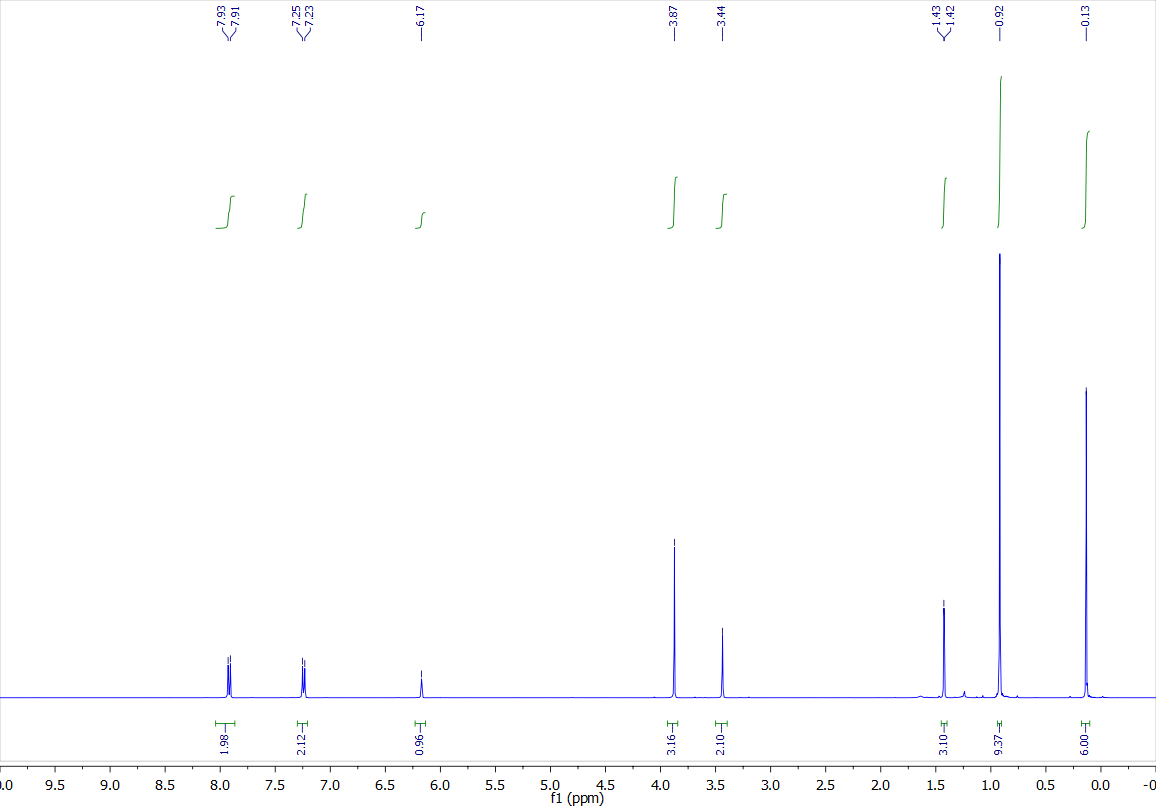
 **Supplementary Figure 41.** ^1^H NMR spectrum of **4**


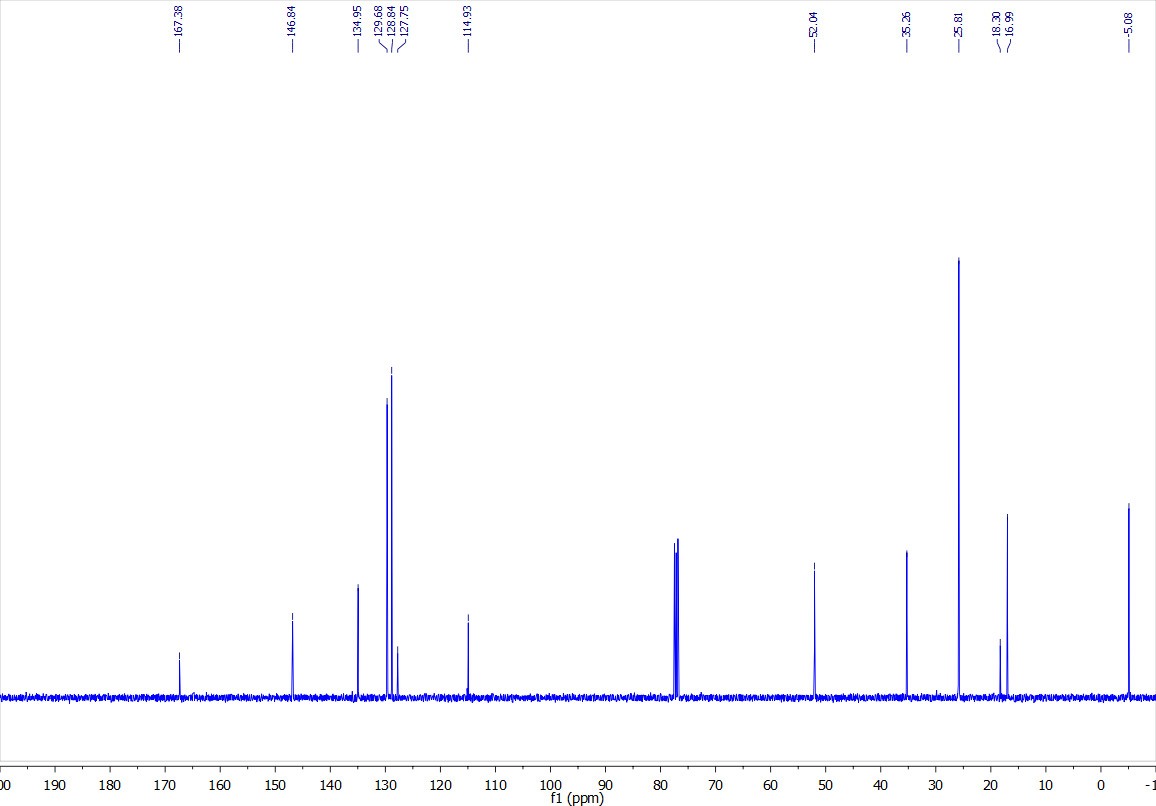
 **Supplementary Figure 42.** ^13^C NMR spectrum of **4**


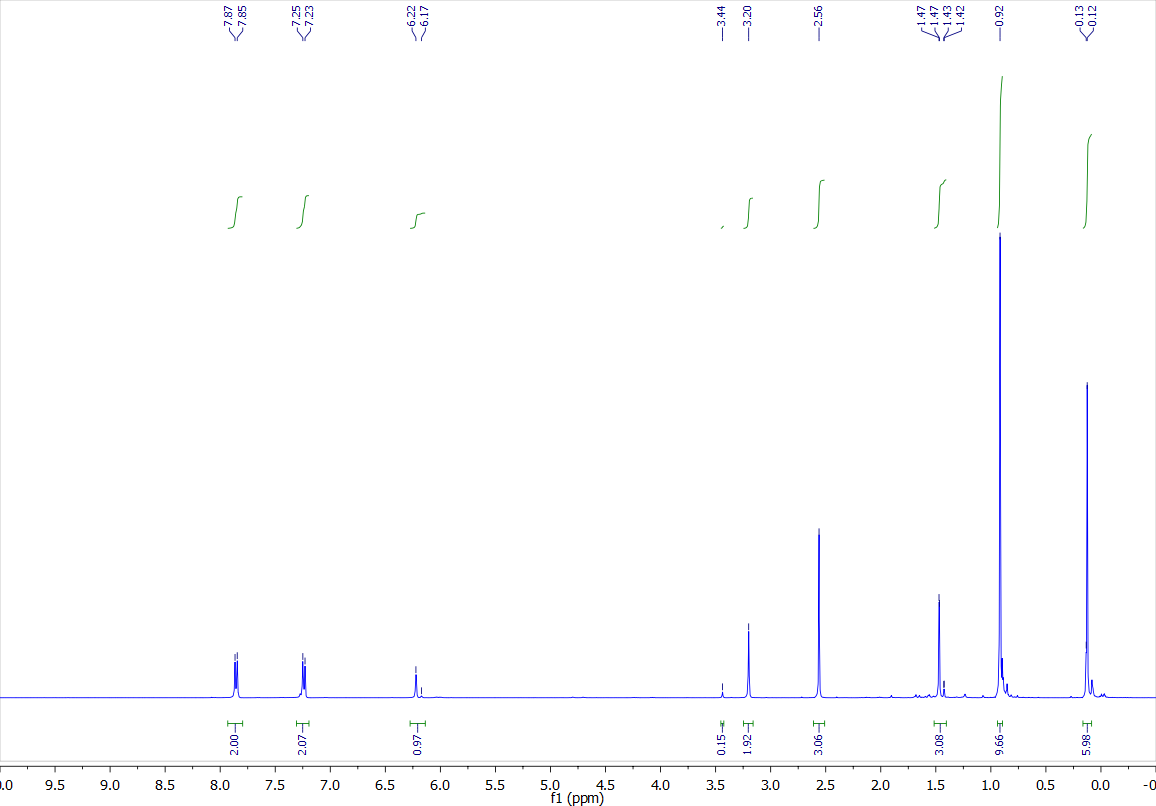
 **Supplementary Figure 43.** ^1^H NMR spectrum of **5**


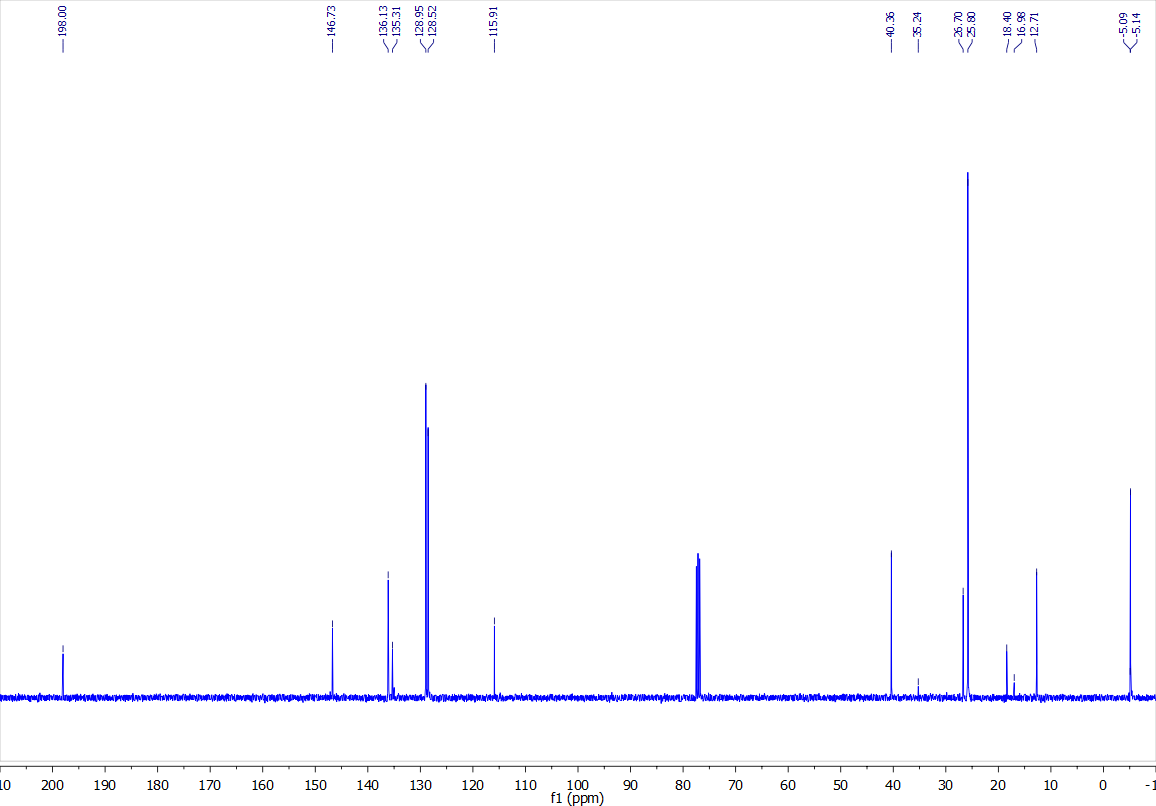
**Supplementary Figure 44.** ^13^C NMR spectrum of **5**


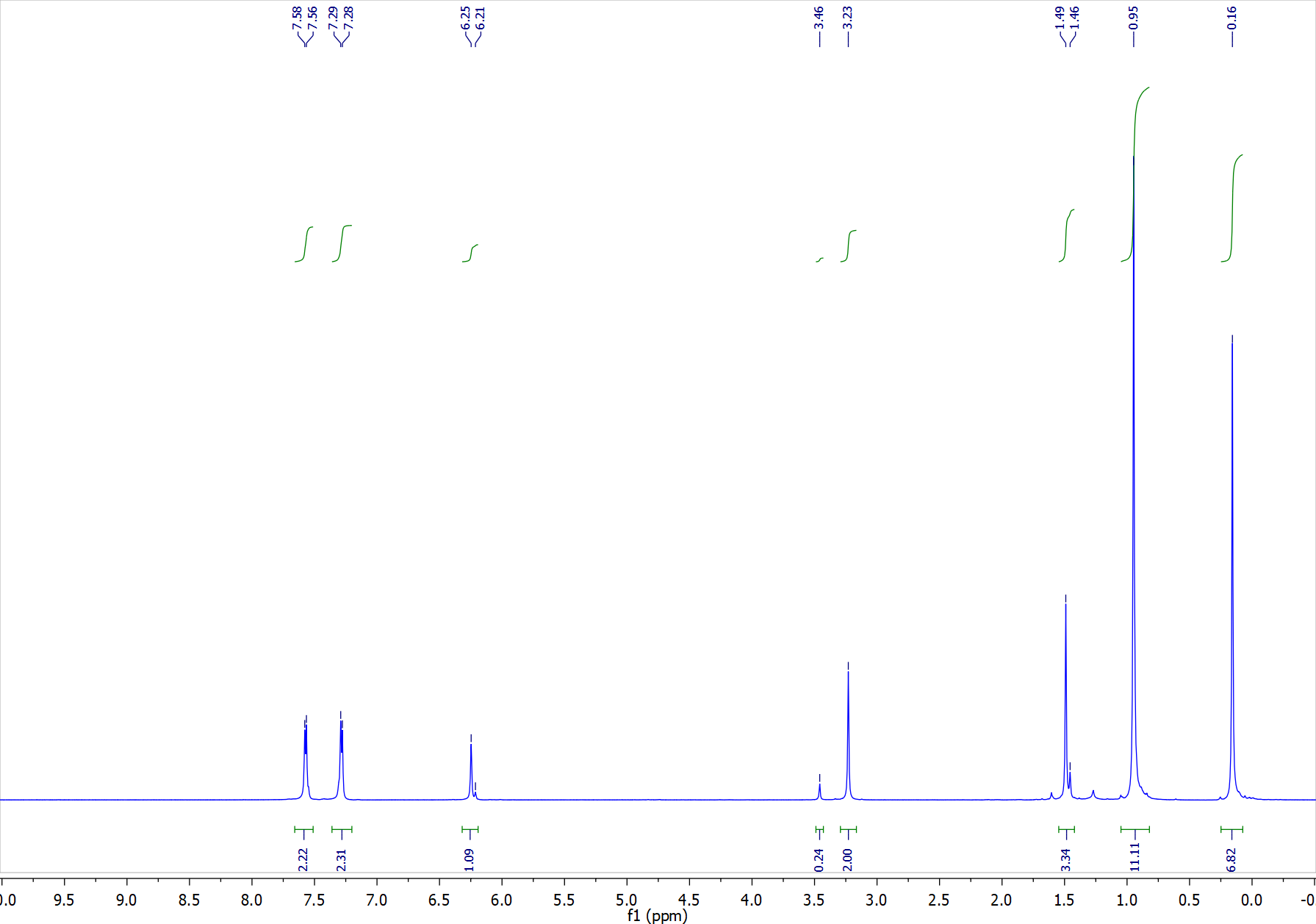
 **Supplementary Figure 45.** ^1^H NMR spectrum of **6**


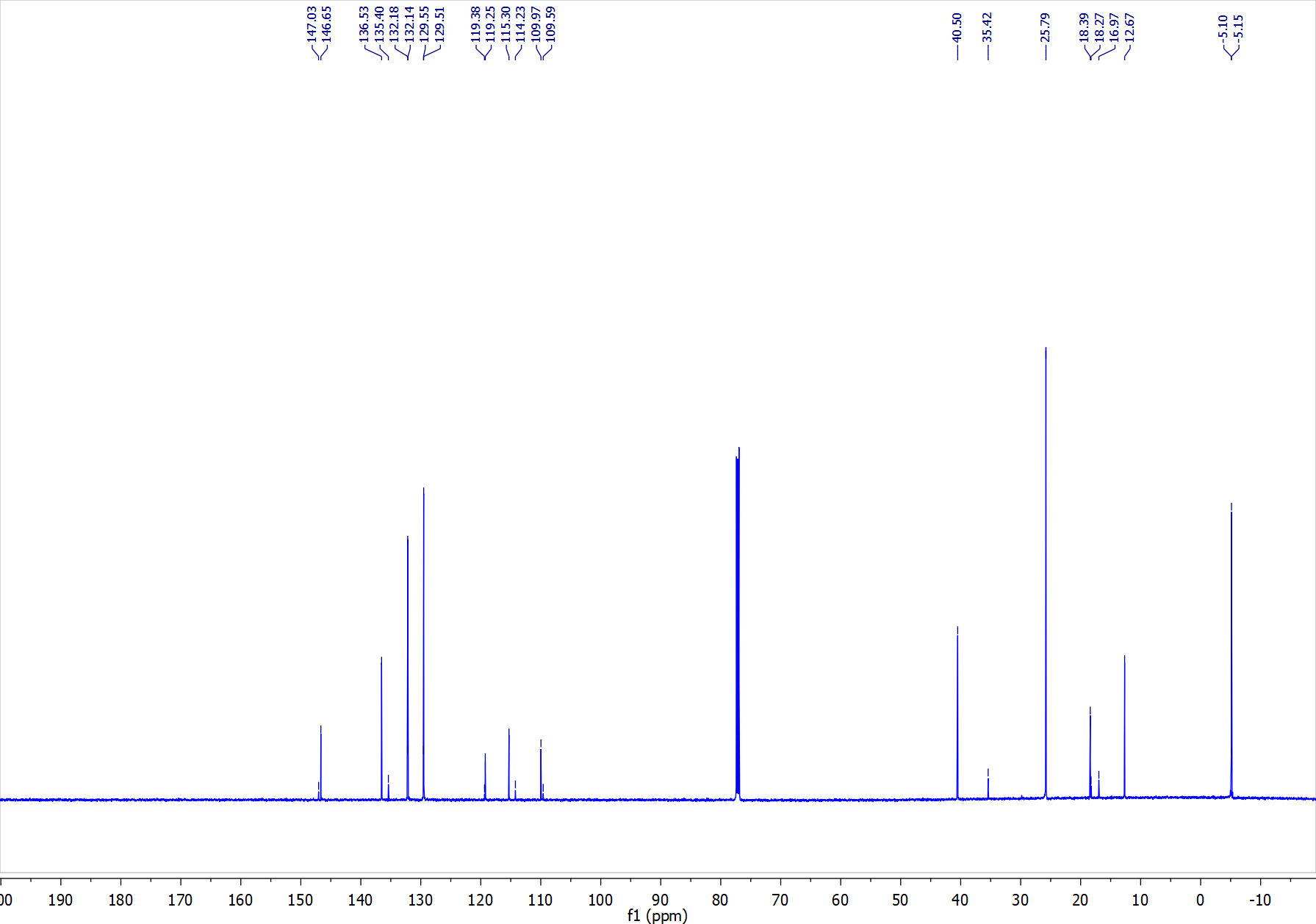
 **Supplementary Figure 46.** ^13^C NMR spectrum of **6**


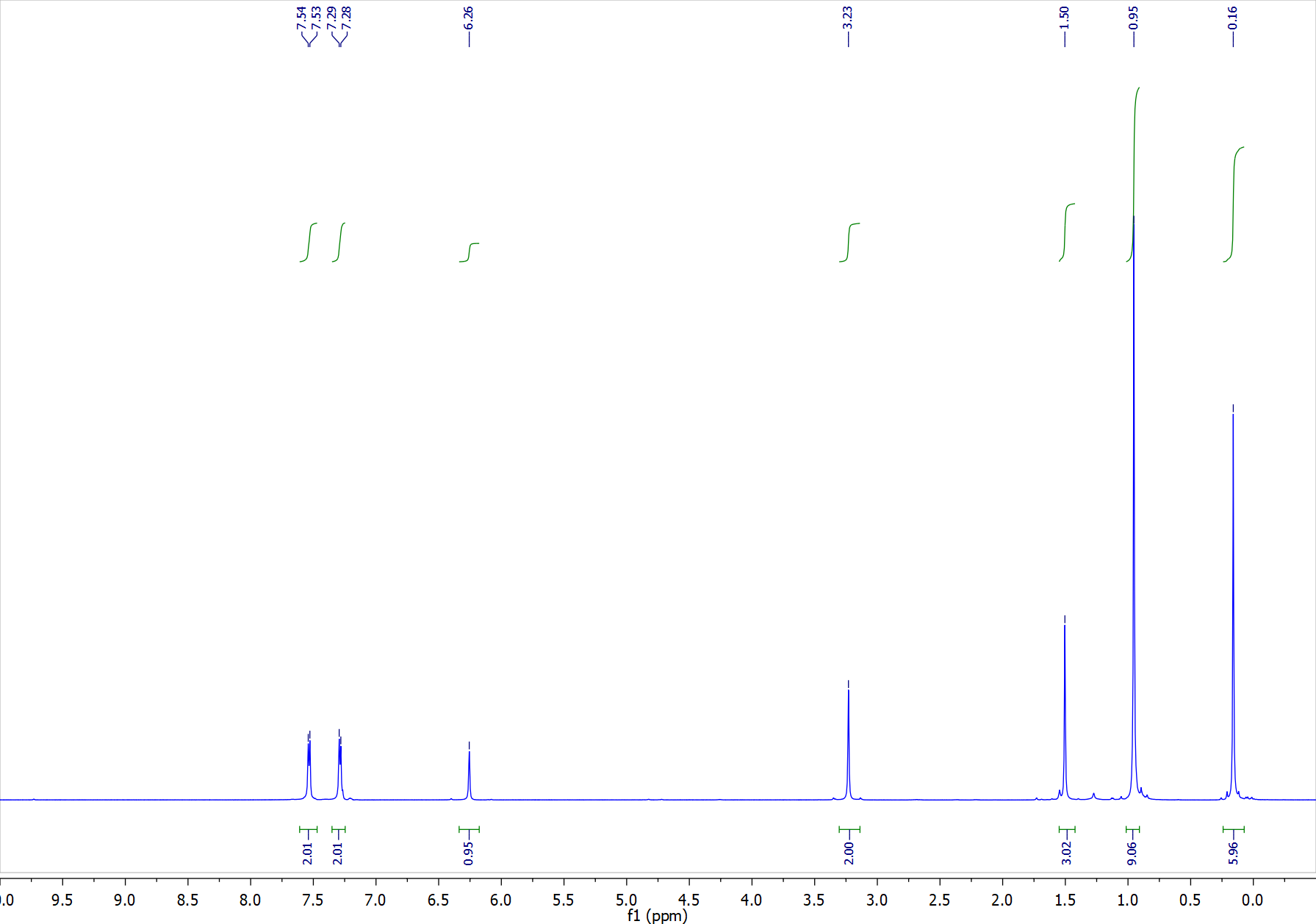
 **Supplementary Figure 47.** ^1^H NMR spectrum of **7**


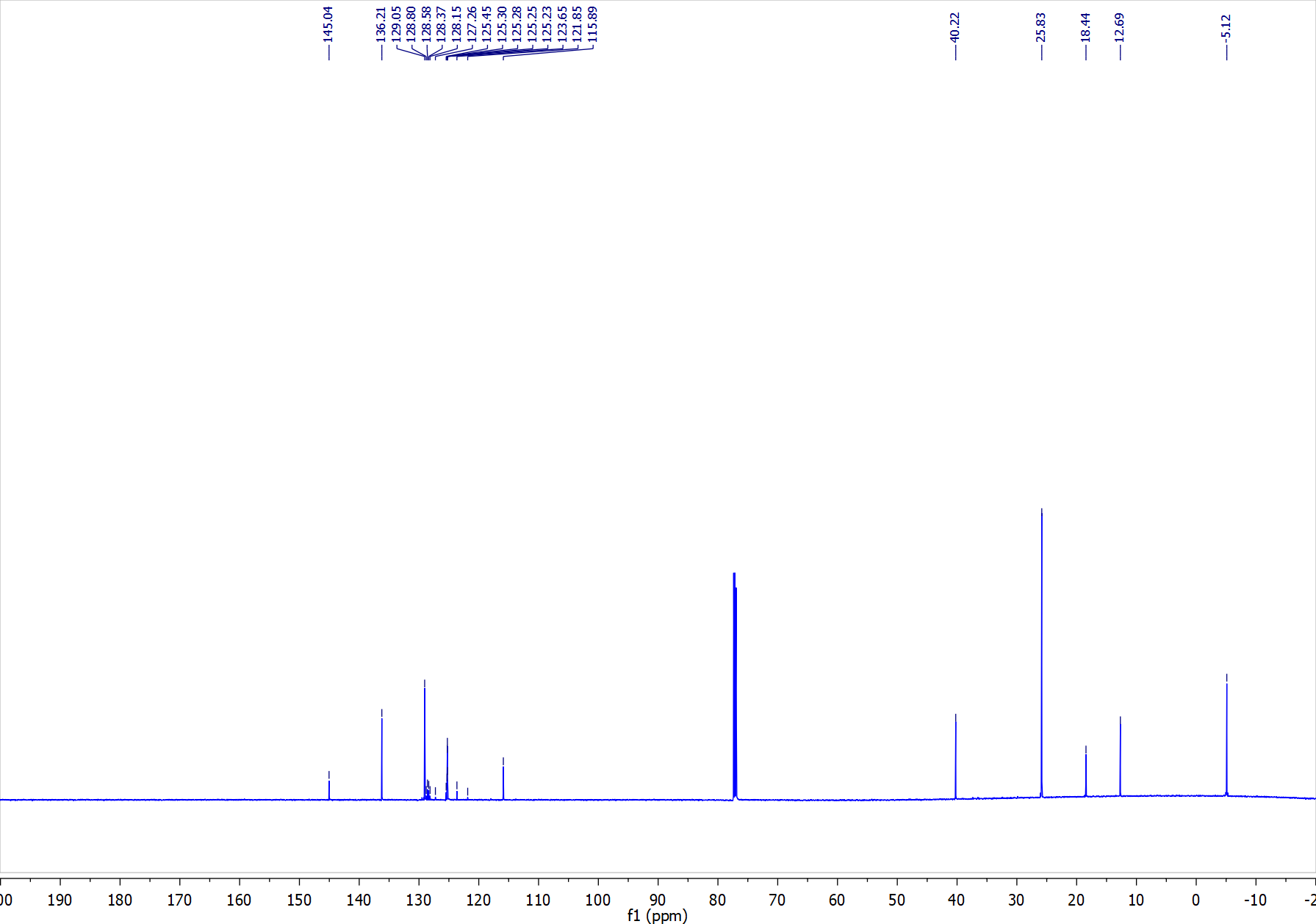
**Supplementary Figure 48.** ^13^C NMR spectrum of **7**


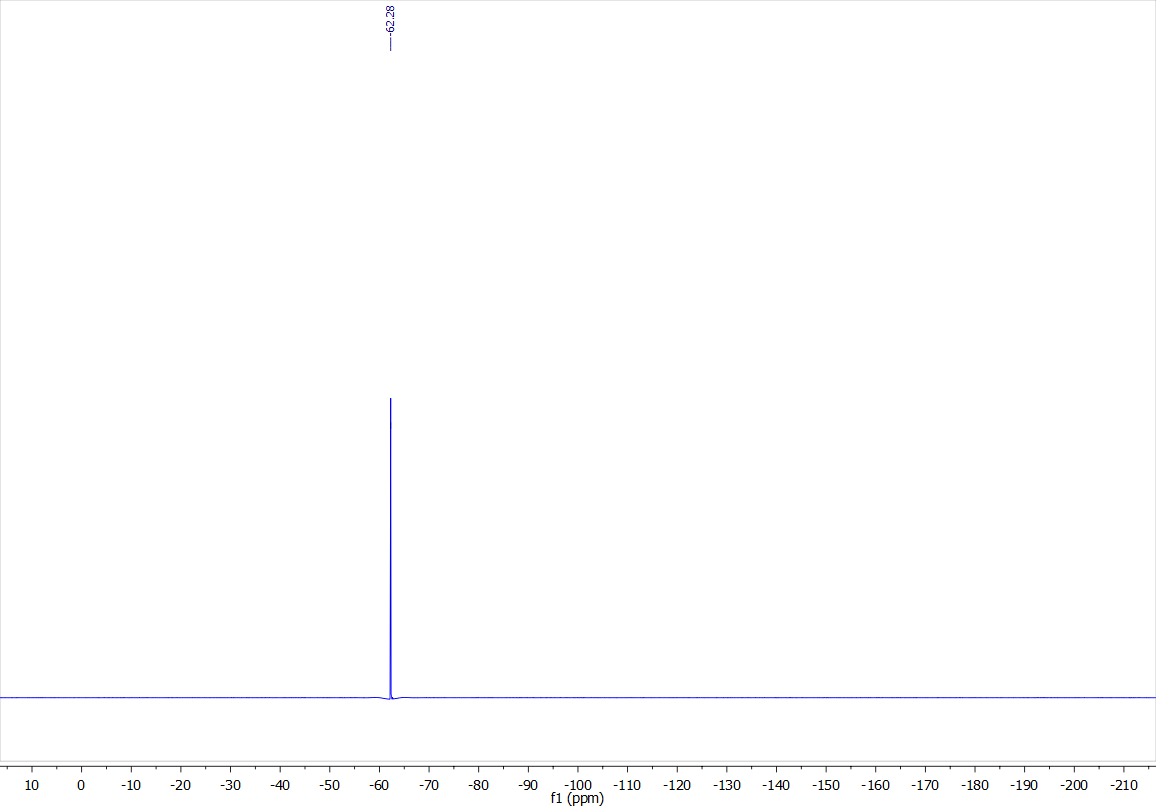
 **Supplementary Figure 49.** ^19^F NMR spectrum of **7**


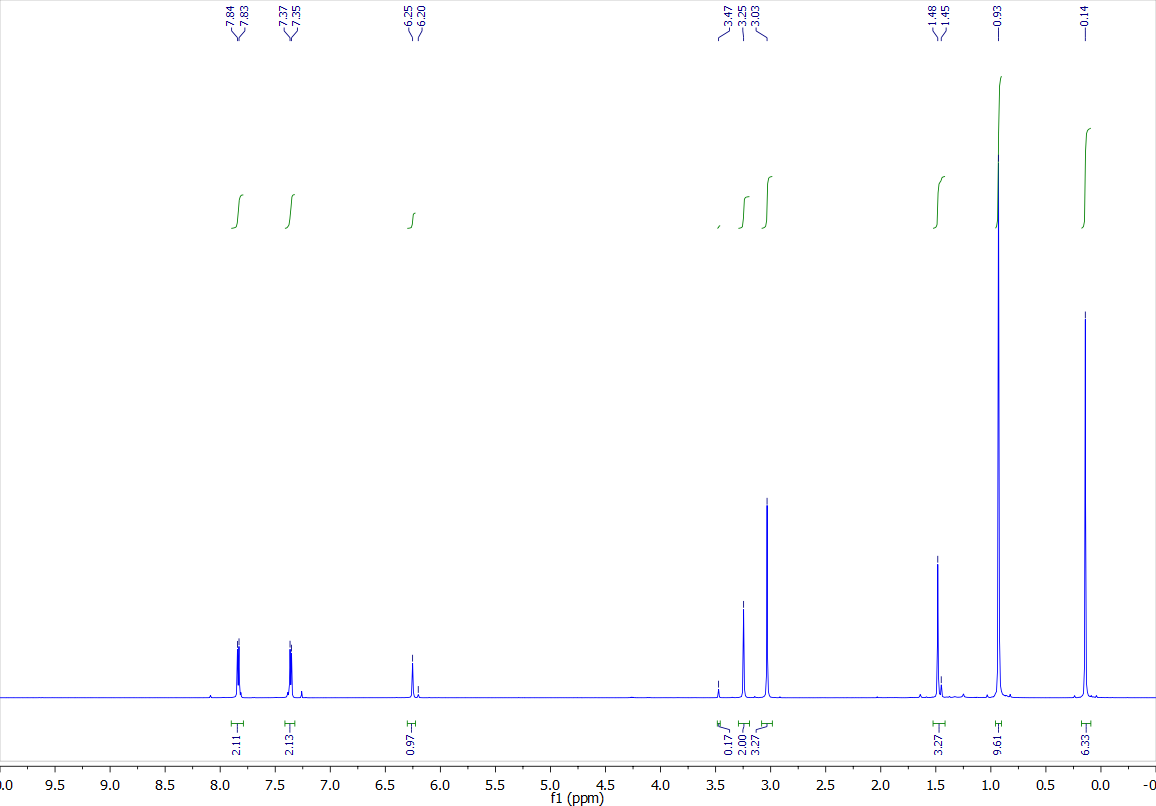
 **Supplementary Figure 50.** ^1^H NMR spectrum of **8**


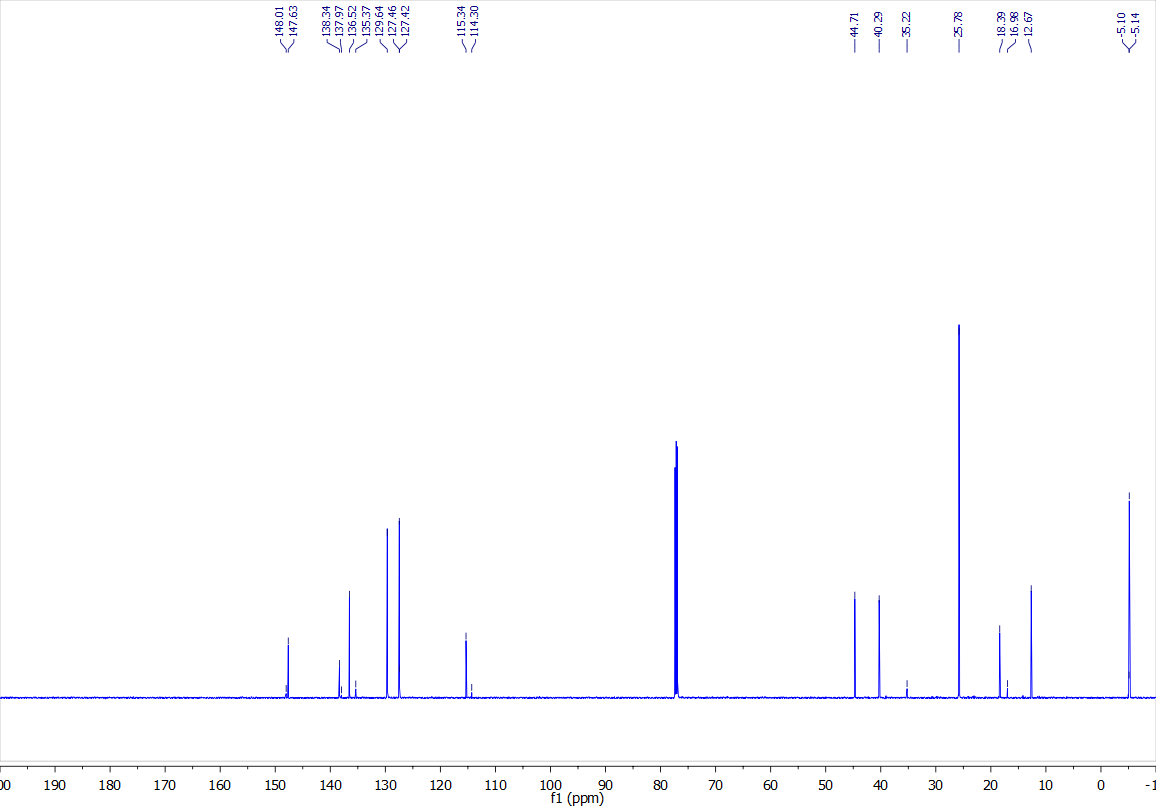
**Supplementary Figure 51.** ^13^C NMR spectrum of **8**


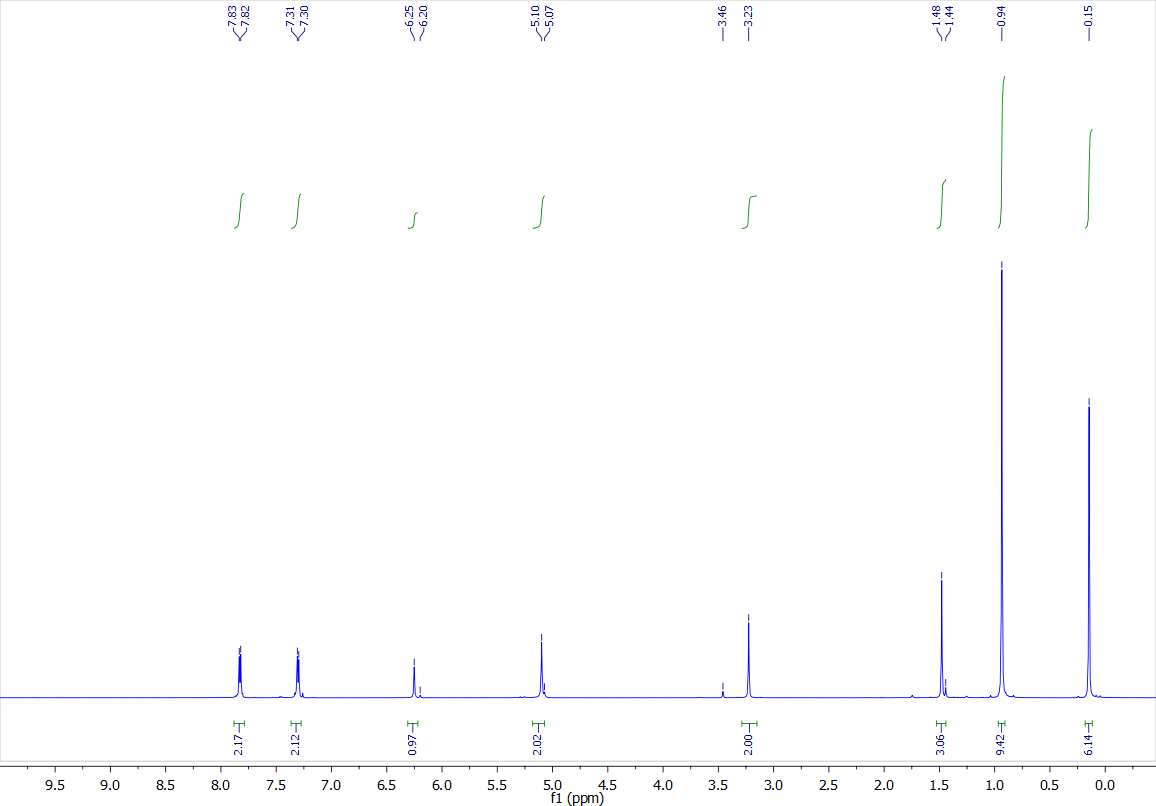
 **Supplementary Figure 52.** ^1^H NMR spectrum of **9**


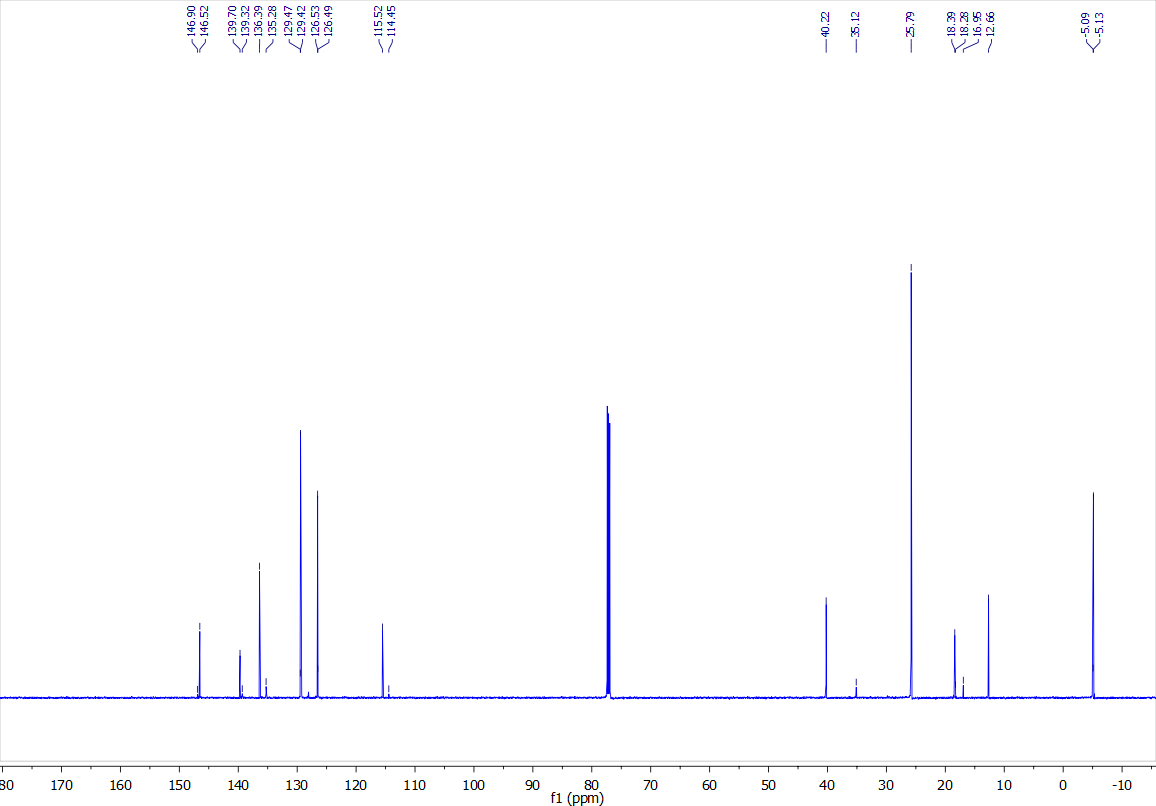
 **Supplementary Figure 53.** ^13^C NMR spectrum of **9**


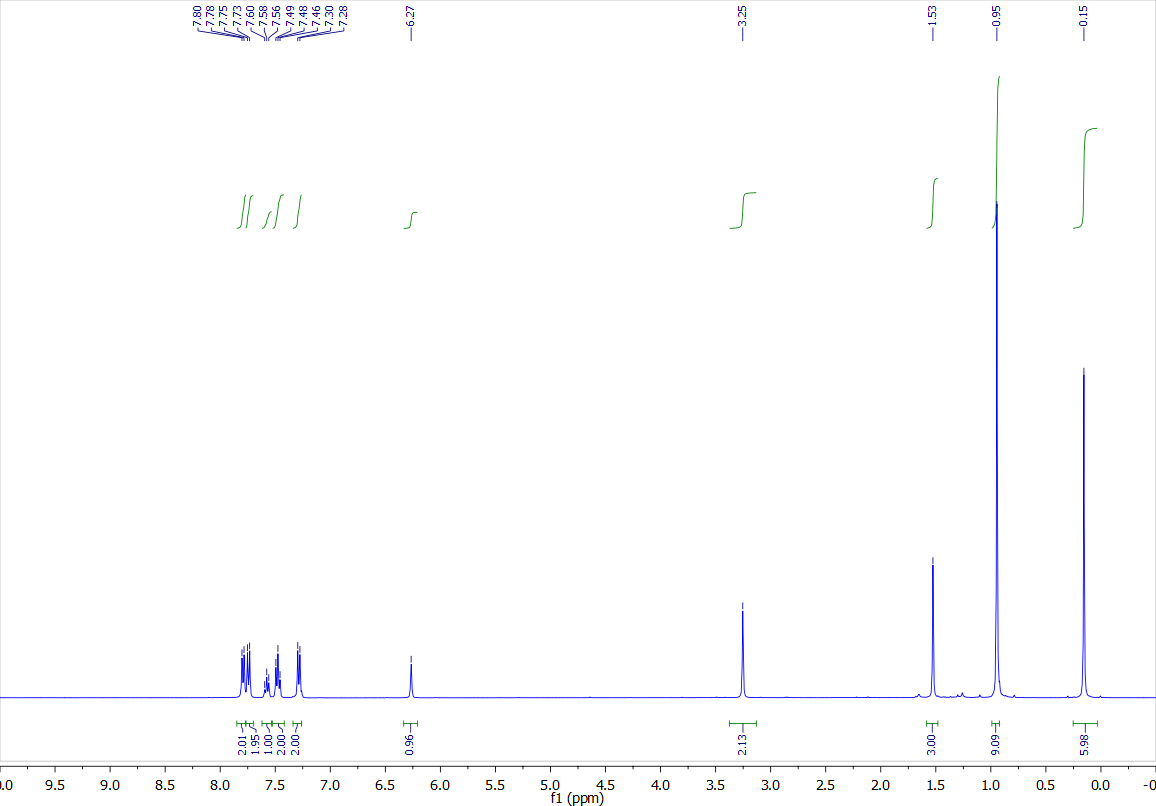
**Supplementary Figure 54.** ^1^H NMR spectrum of **10**


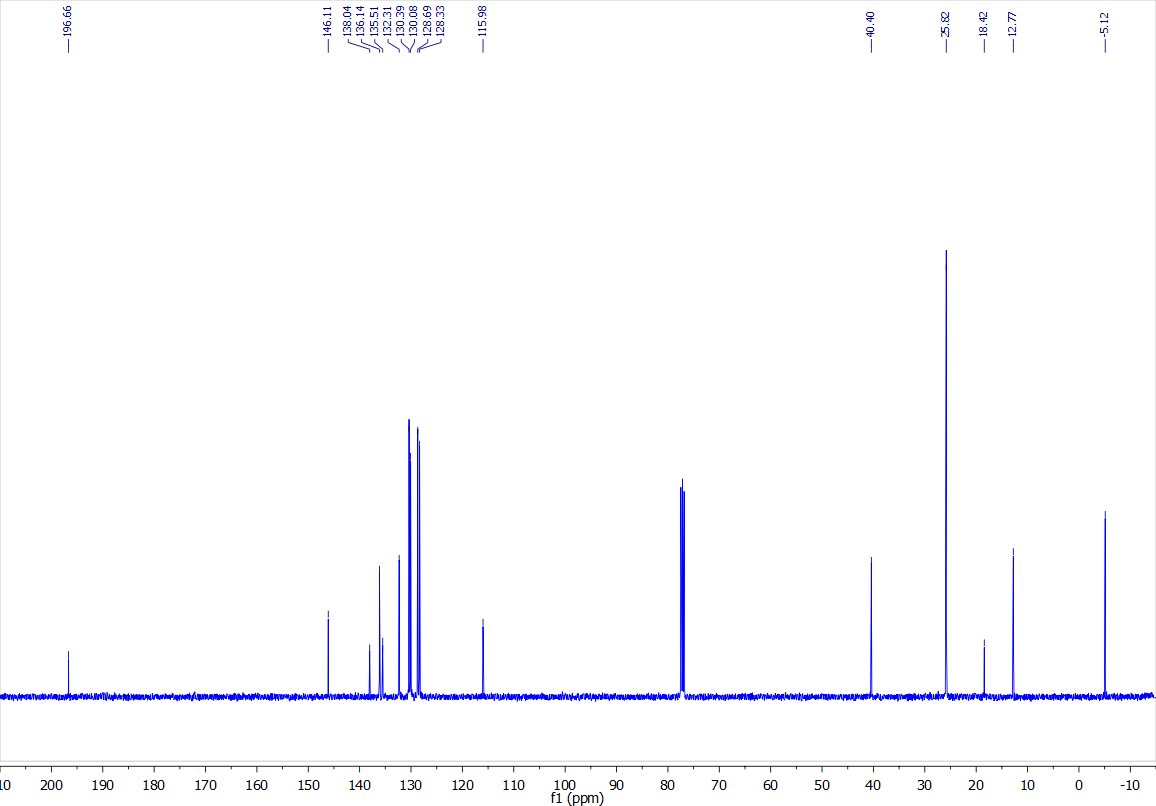
 **Supplementary Figure 55.** ^13^C NMR spectrum of **10**


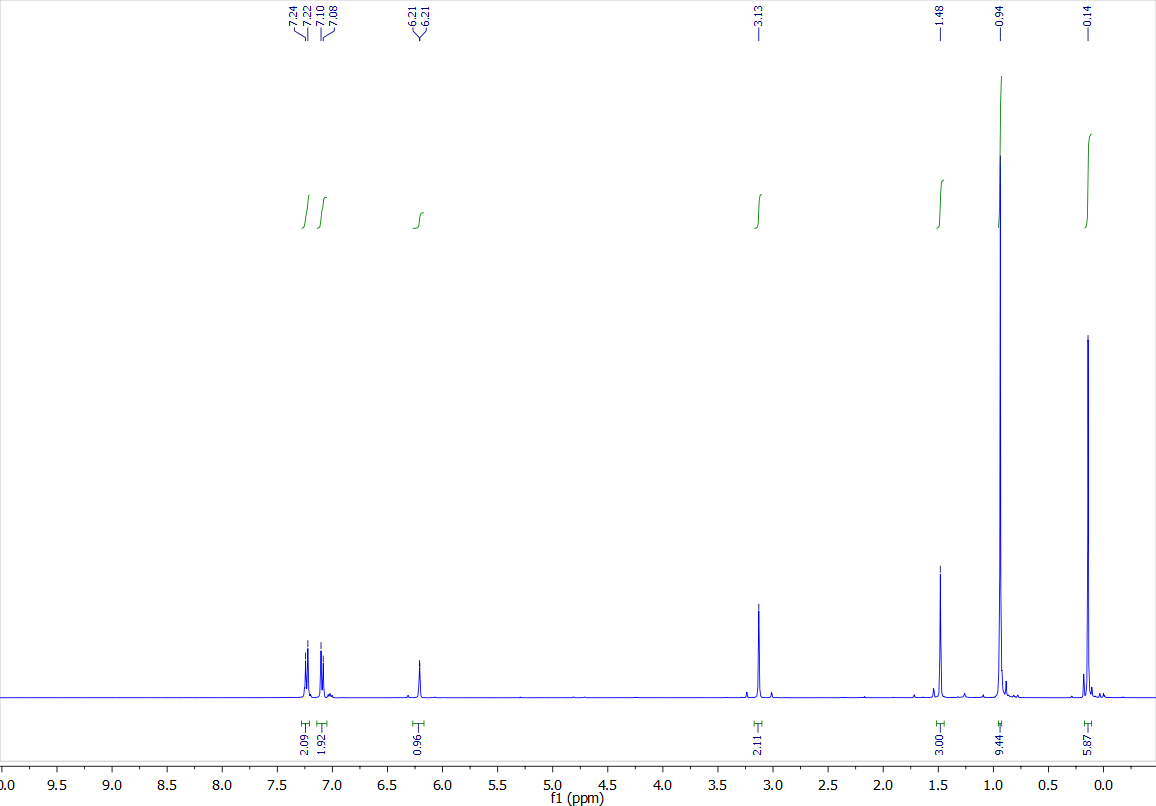
**Supplementary Figure 56.** ^1^H NMR spectrum of **11**
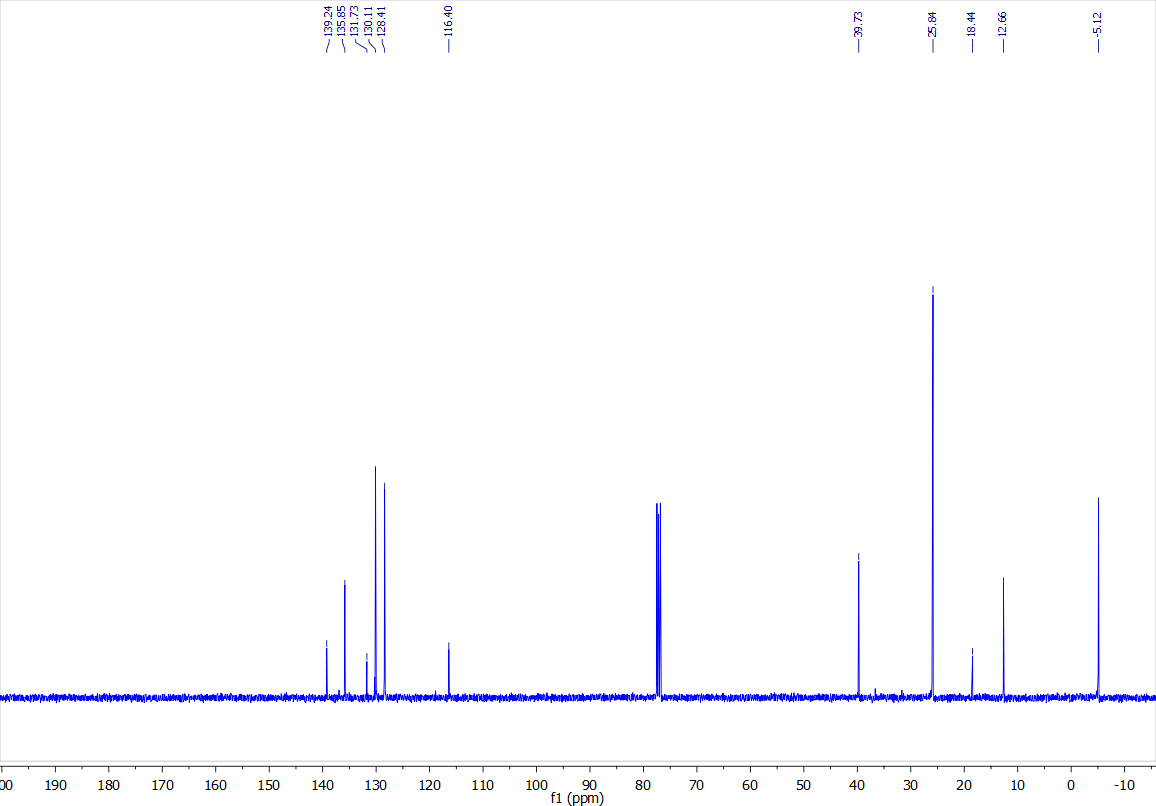
 **Supplementary Figure 57.** ^13^C NMR spectrum of **11**


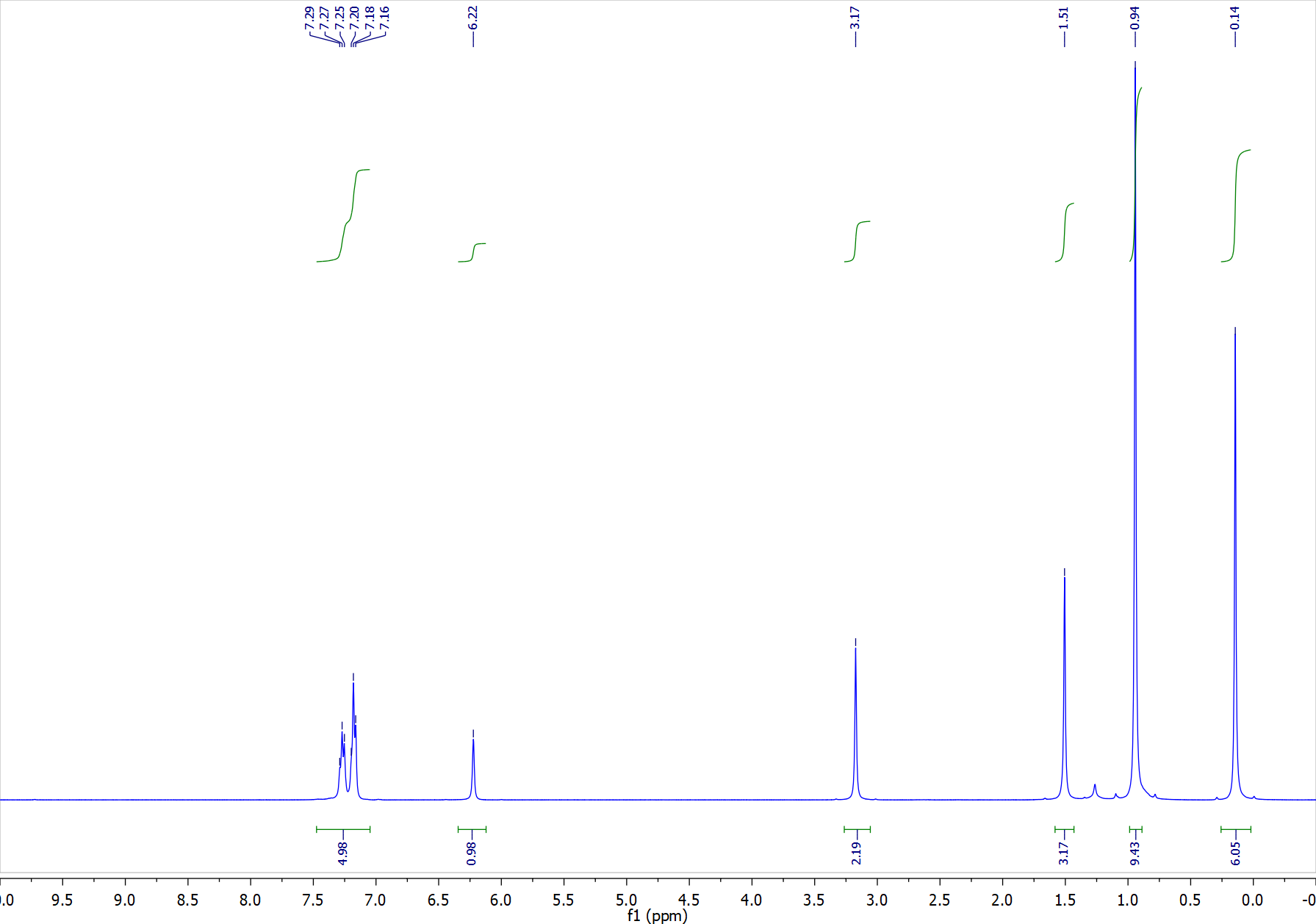
**Supplementary Figure 58.** ^1^H NMR spectrum of **12**
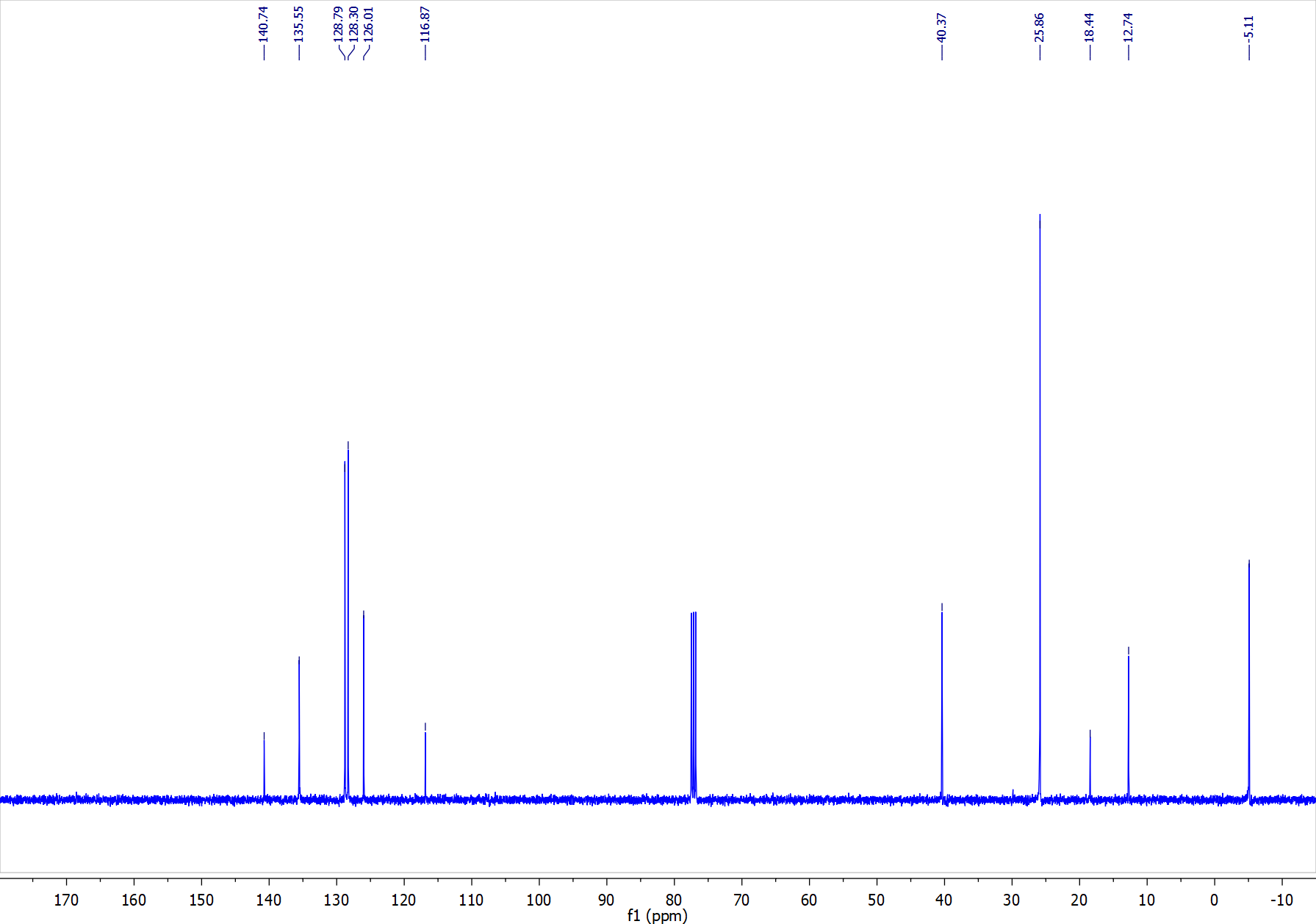
 **Supplementary Figure 59.** ^13^C NMR spectrum of **12**


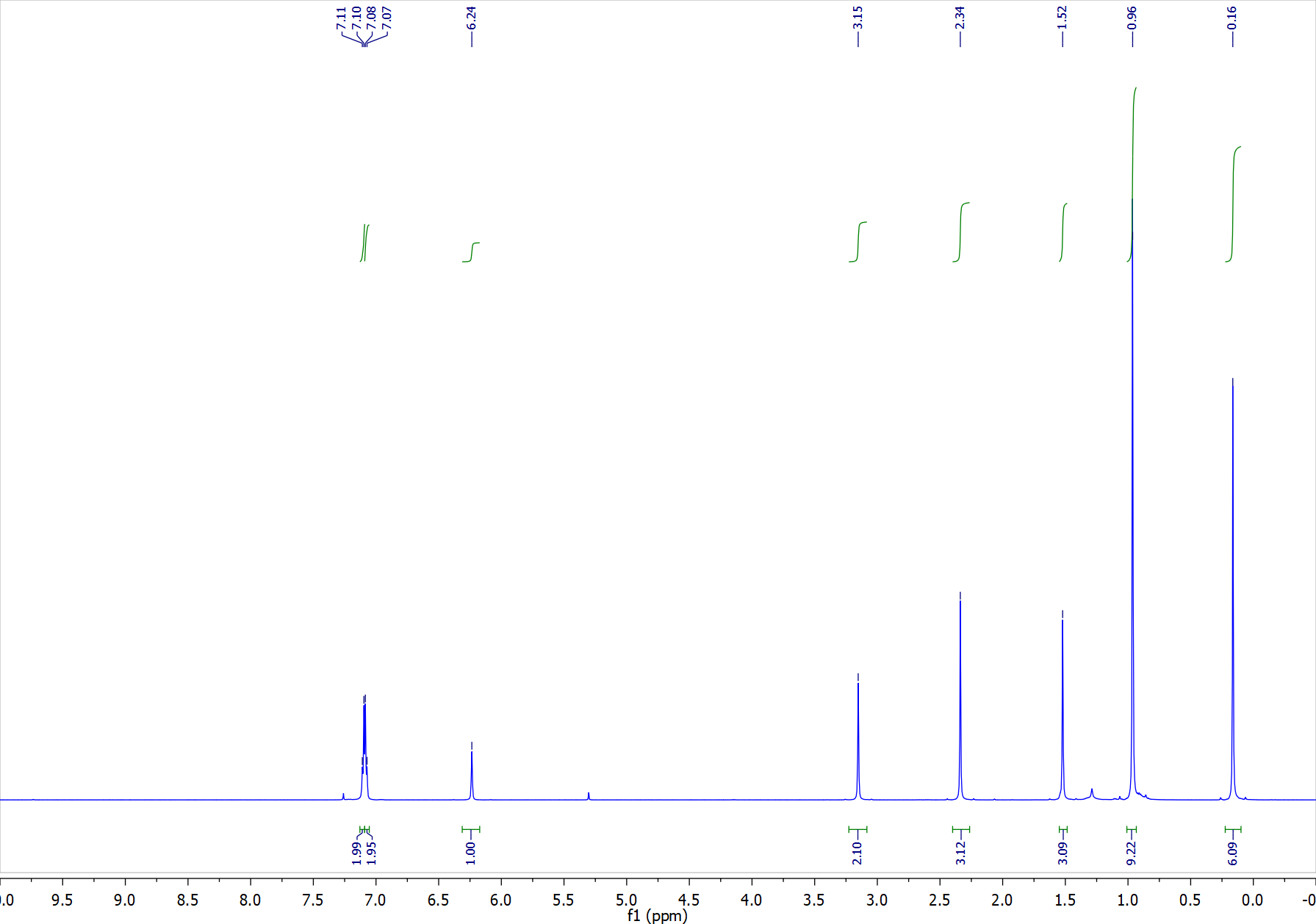
**Supplementary Figure 60.** ^1^H NMR spectrum of **13**
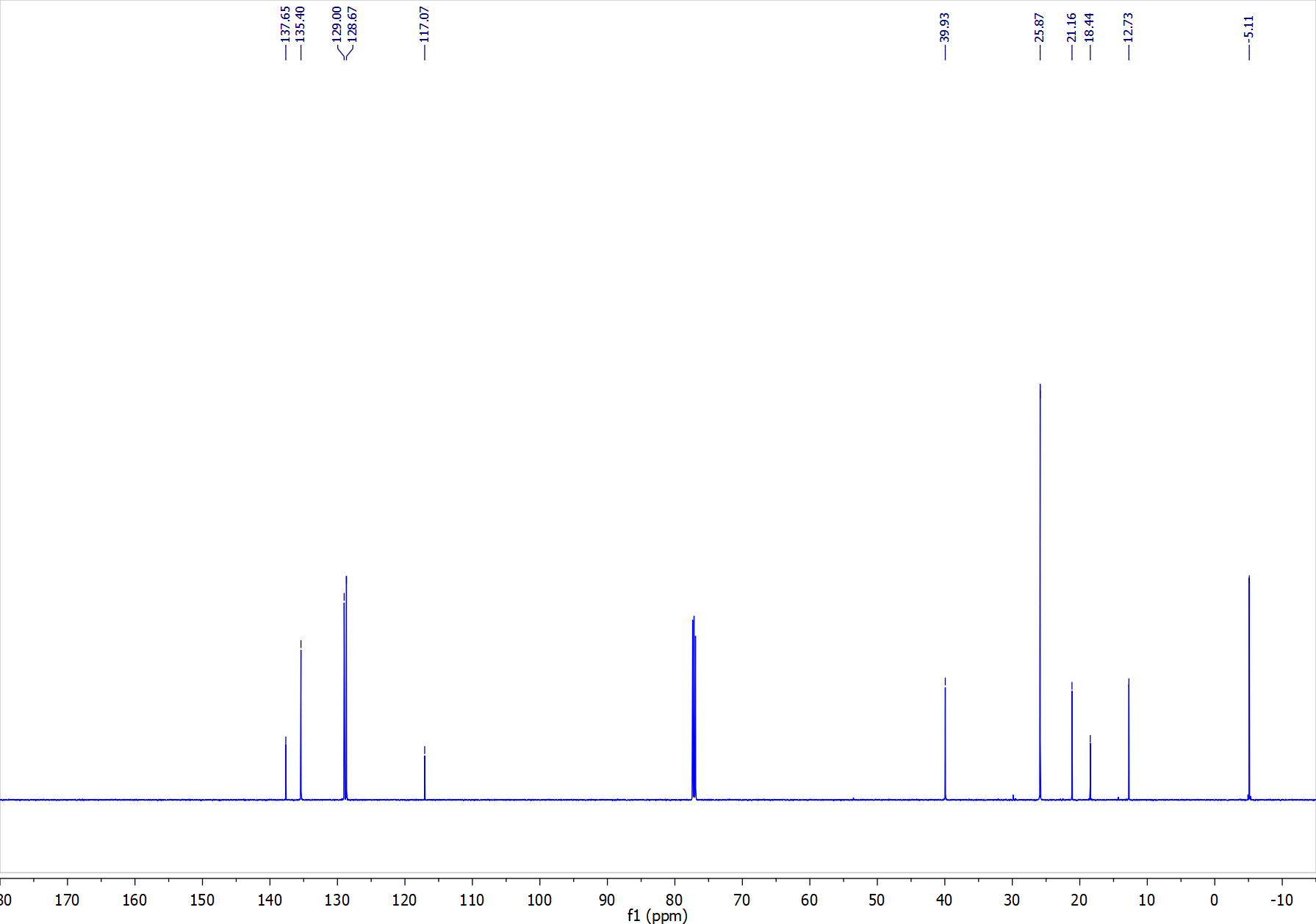
 **Supplementary Figure 61.** ^13^C NMR spectrum of **13**


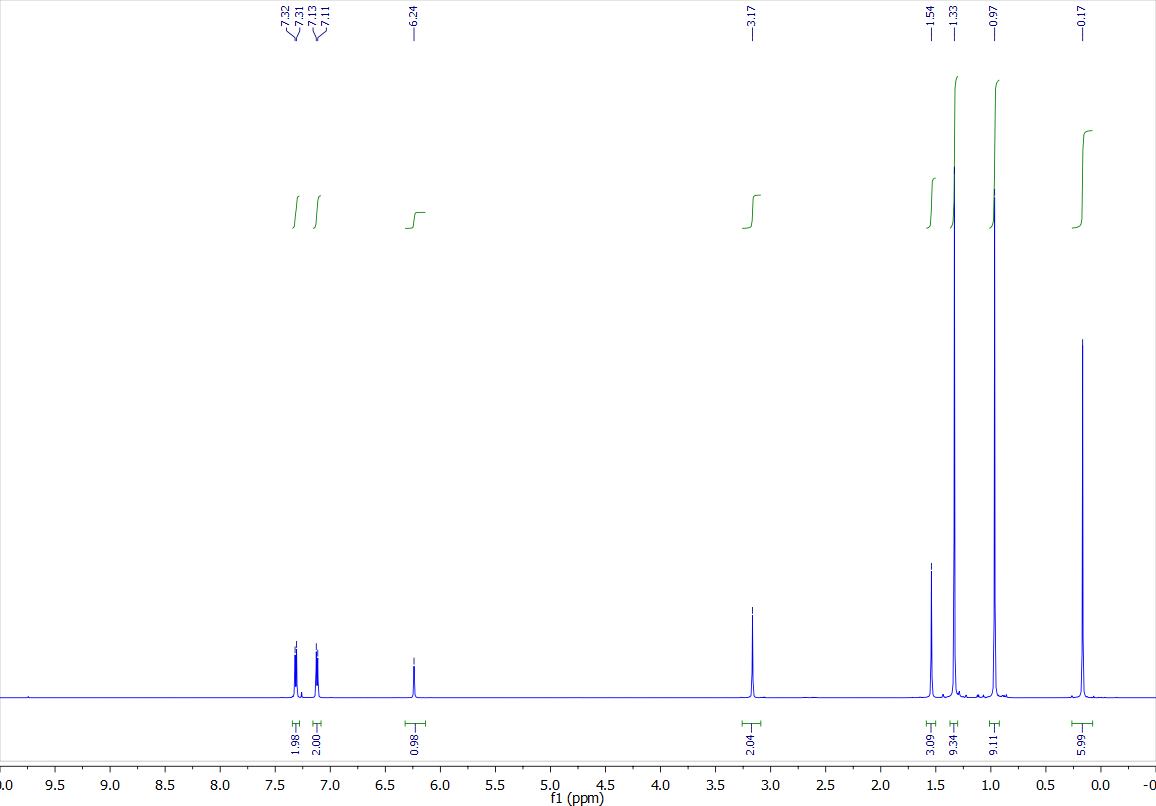
**Supplementary Figure 62.** ^1^H NMR spectrum of **14**
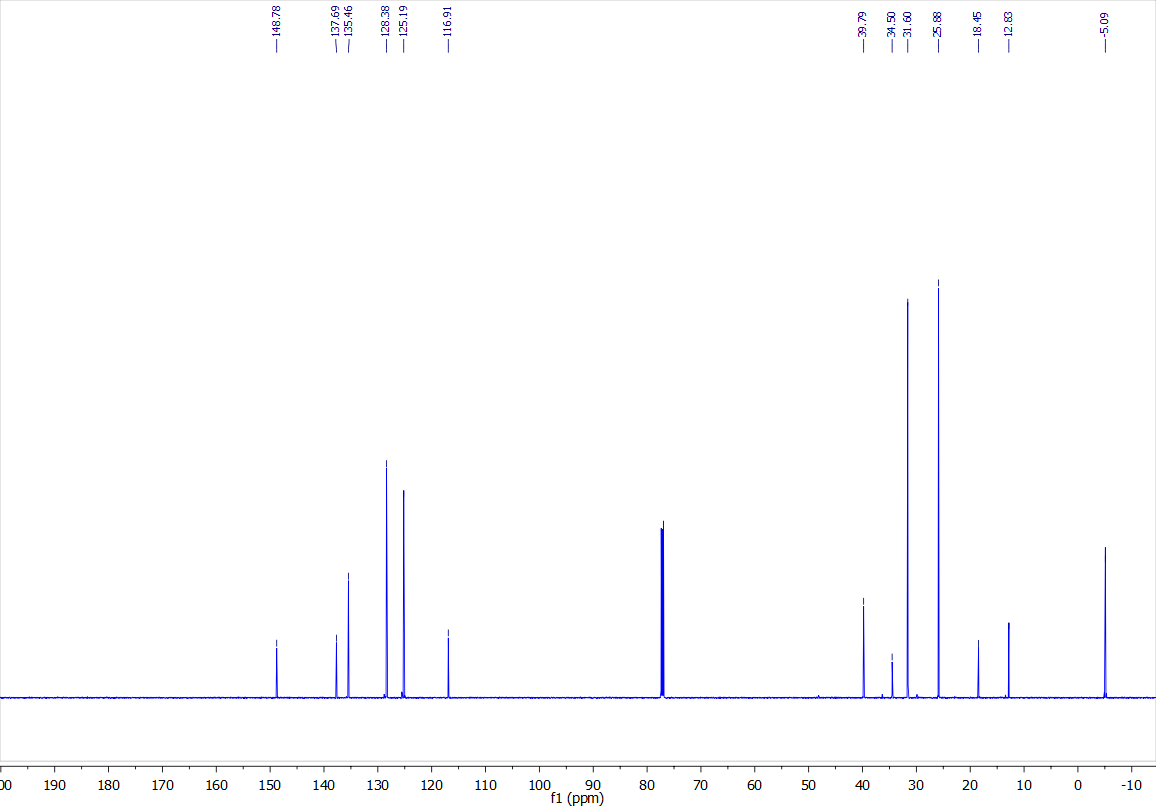
 **Supplementary Figure 63.** ^13^C NMR spectrum of **14**


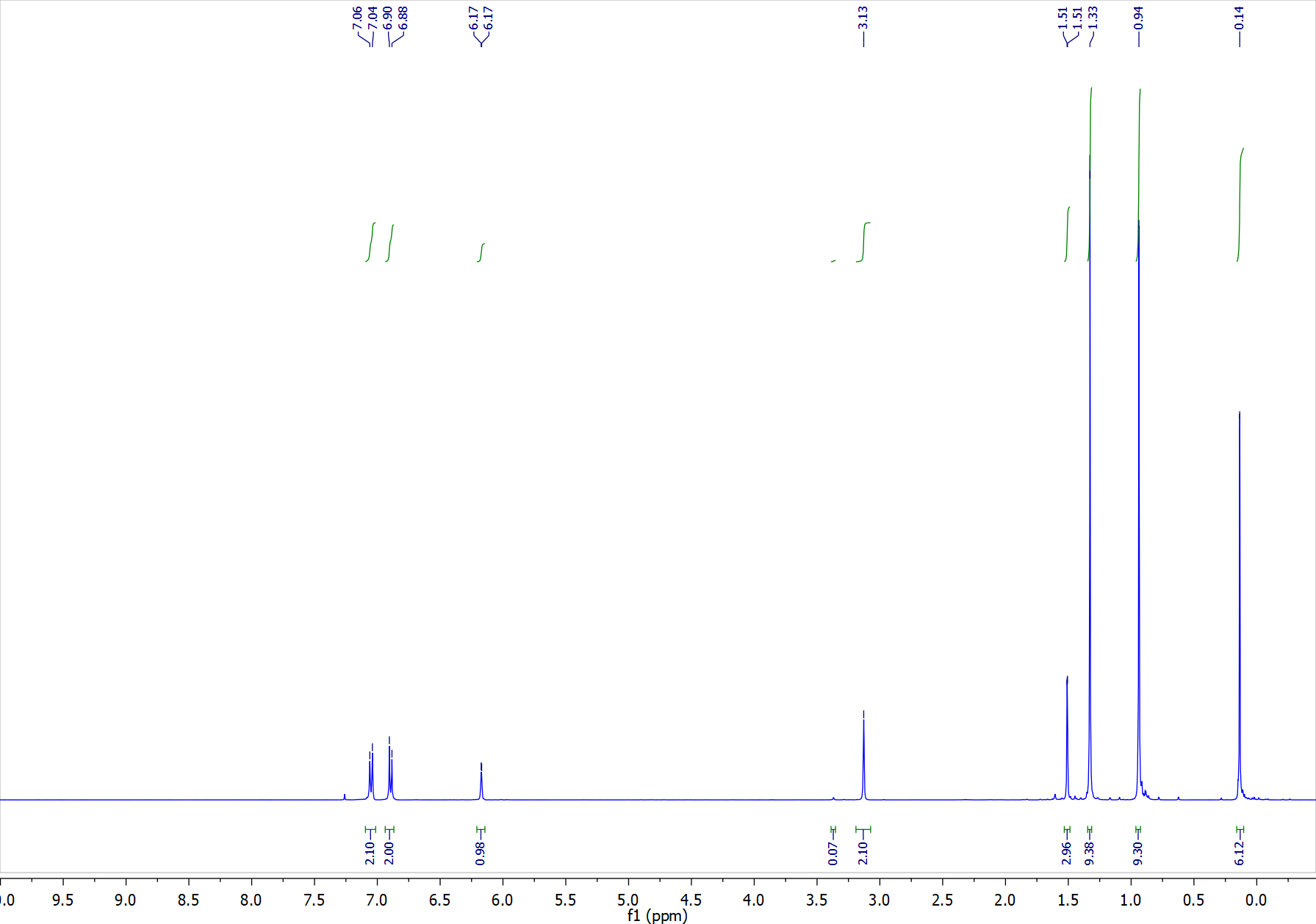
**Supplementary Figure 64.** ^1^H NMR spectrum of **15**
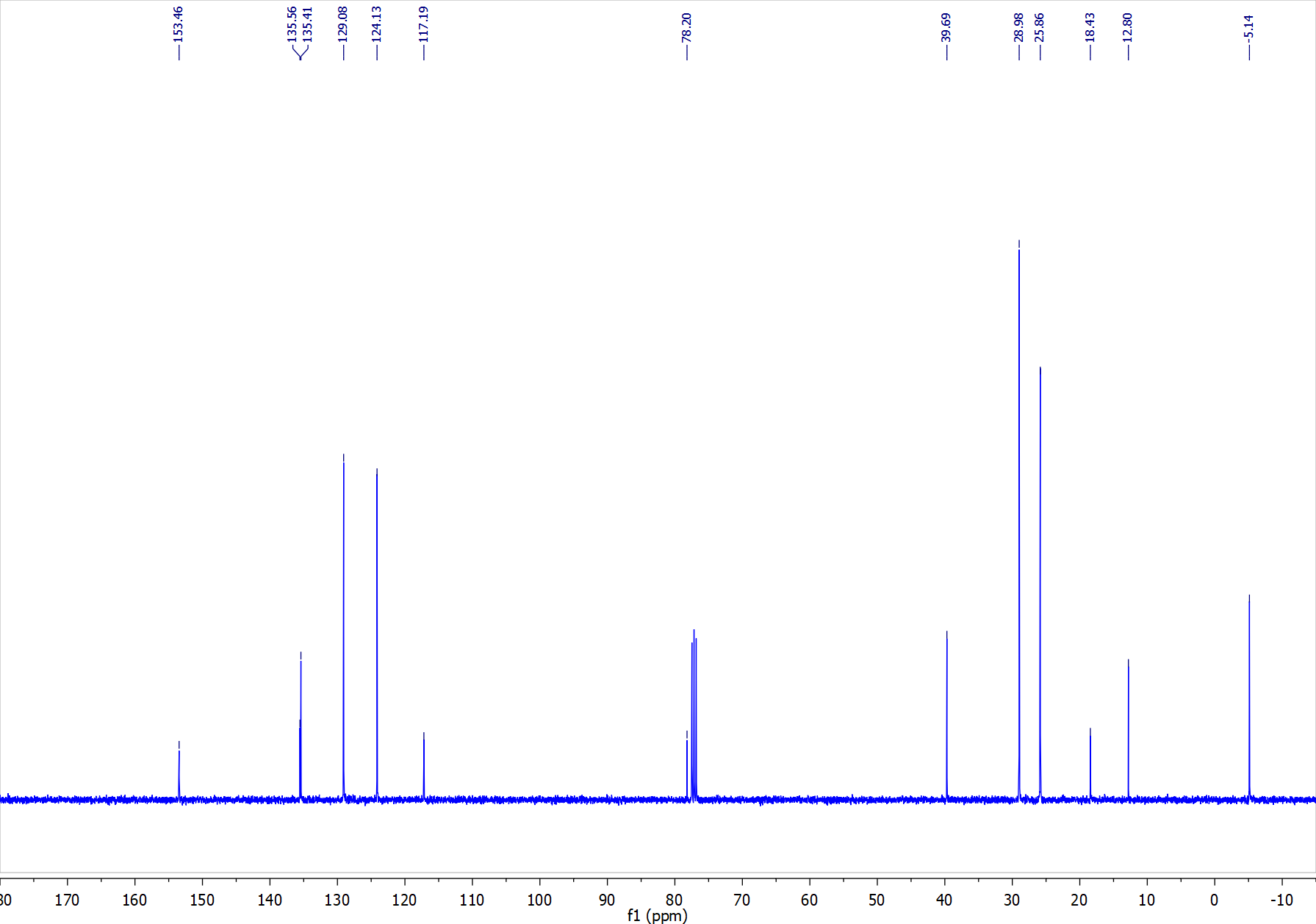
 **Supplementary Figure 65.** ^13^C NMR spectrum of **15**


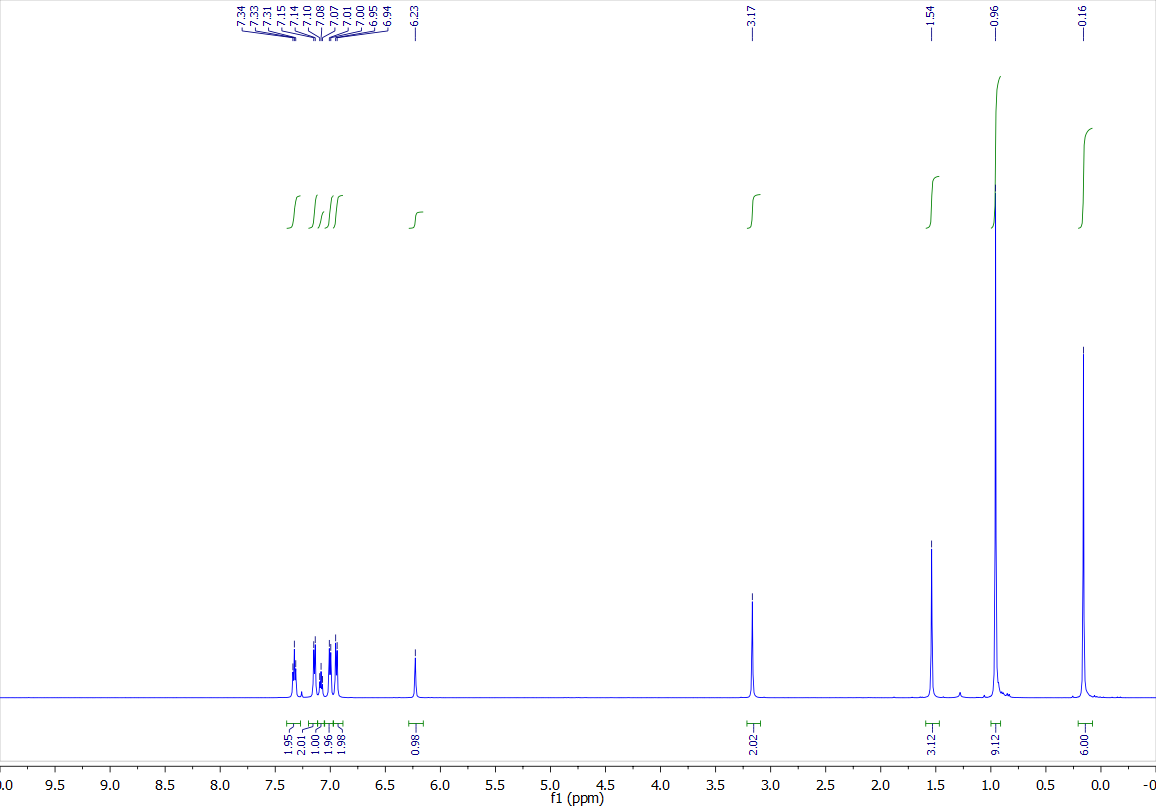
**Supplementary Figure 66.** ^1^H NMR spectrum of **16**
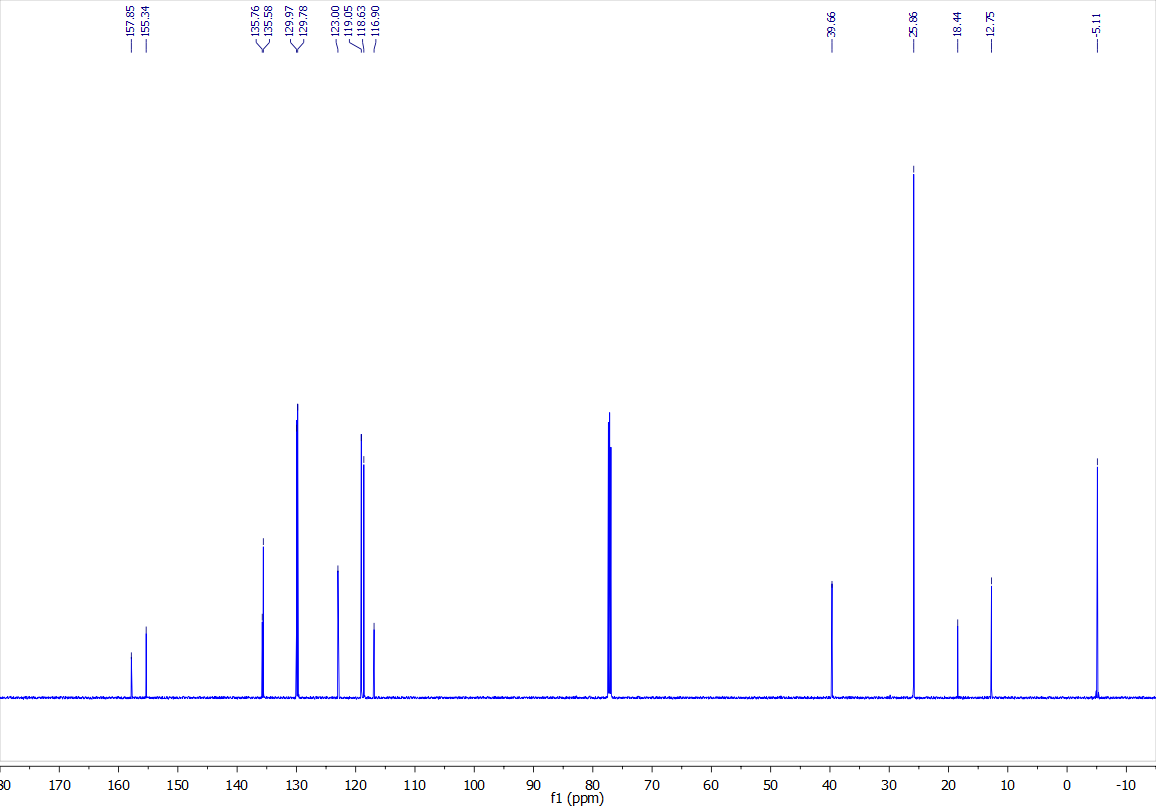
 **Supplementary Figure 67.** ^13^C NMR spectrum of **16**


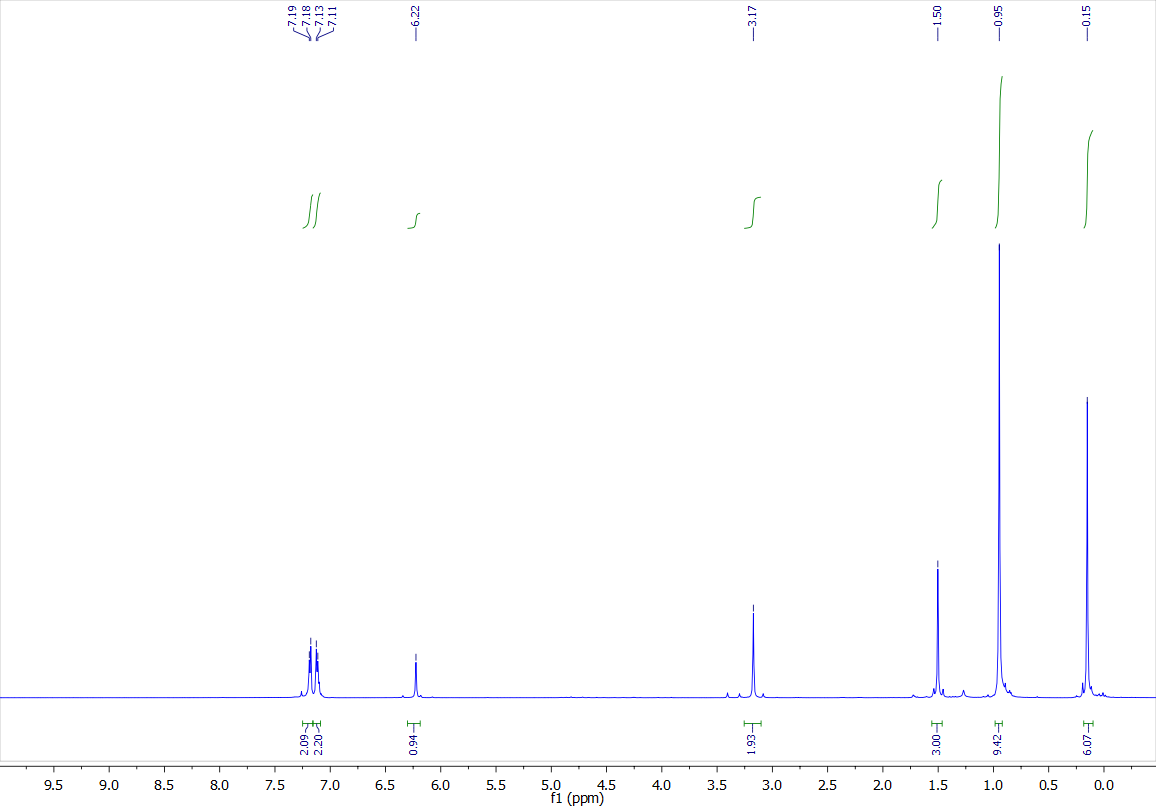
**Supplementary Figure 68.** ^1^H NMR spectrum of **17**
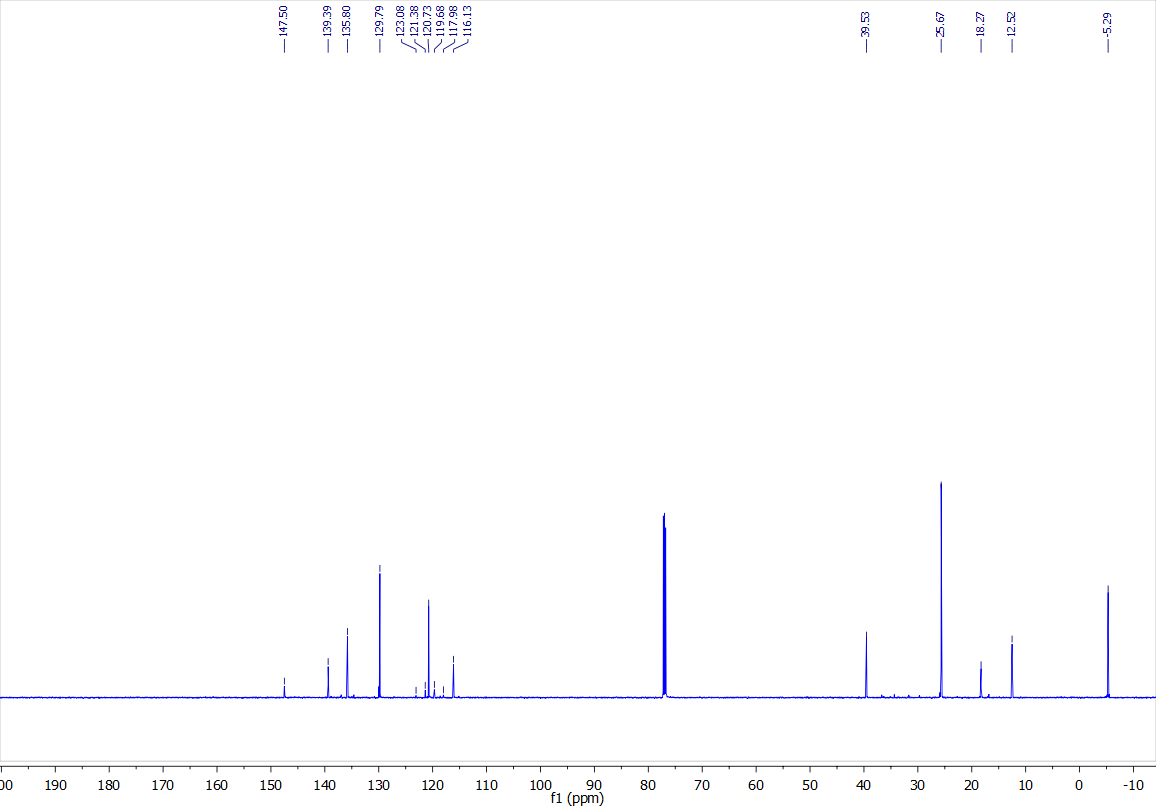
 **Supplementary Figure 69.** ^13^C NMR spectrum of **17**


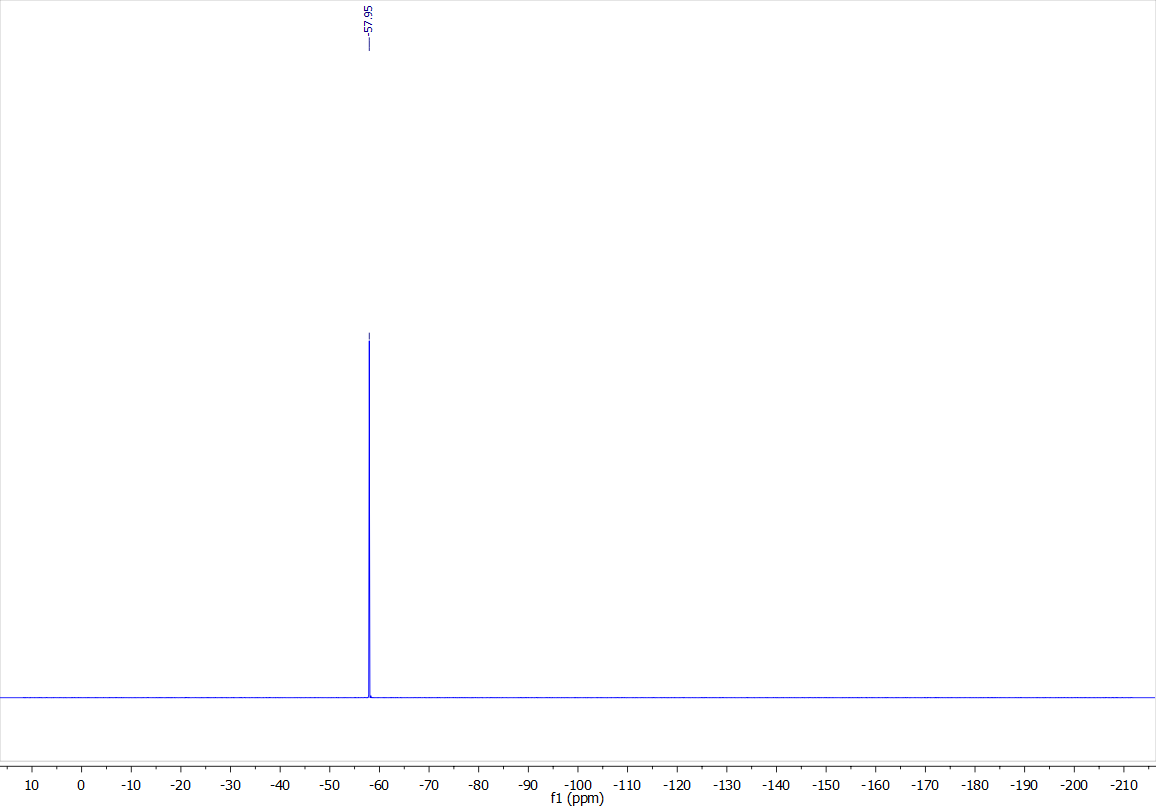
**Supplementary Figure 70.** ^19^F NMR spectrum of **17**


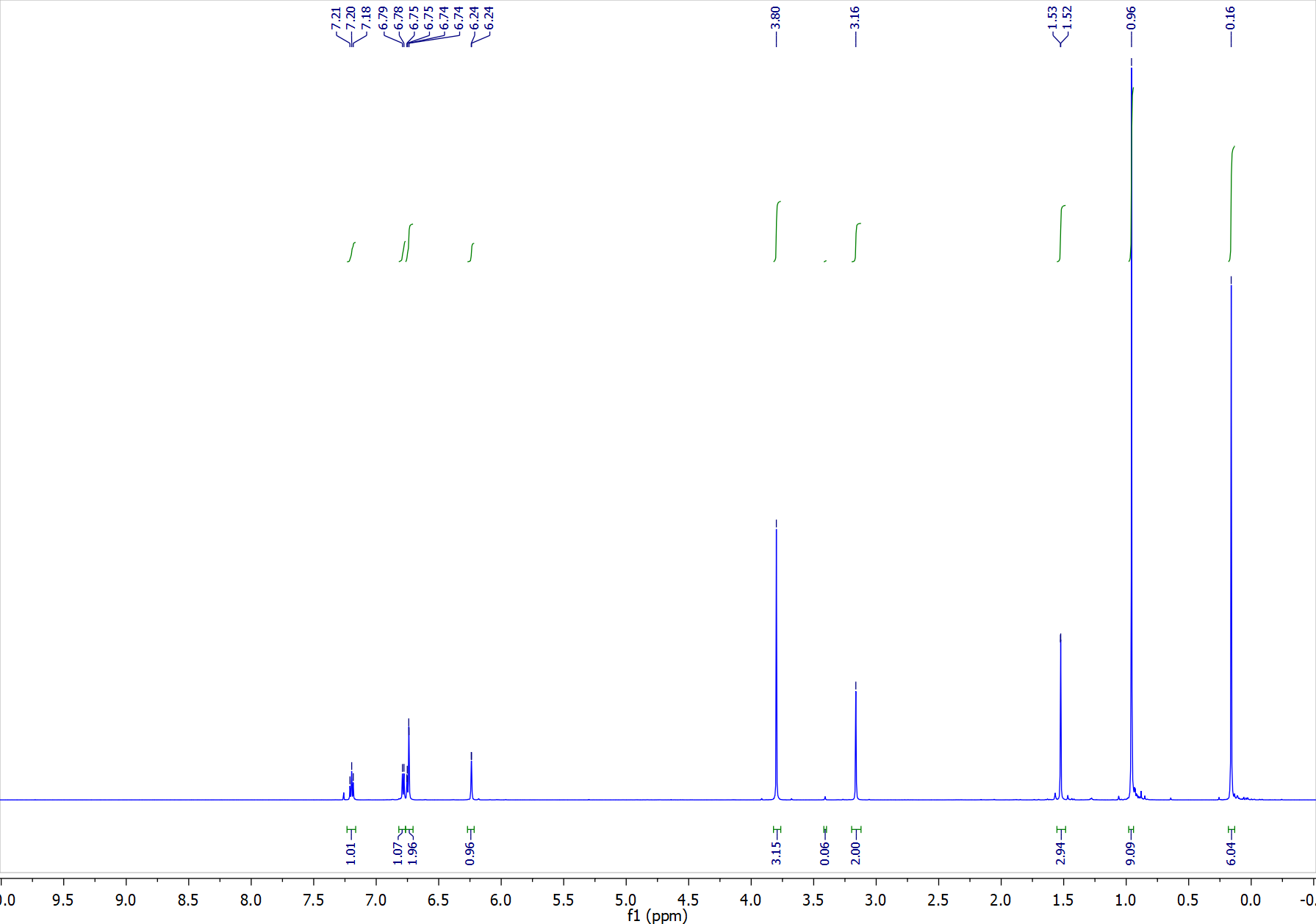
**Supplementary Figure 71.** ^1^H NMR spectrum of **18**
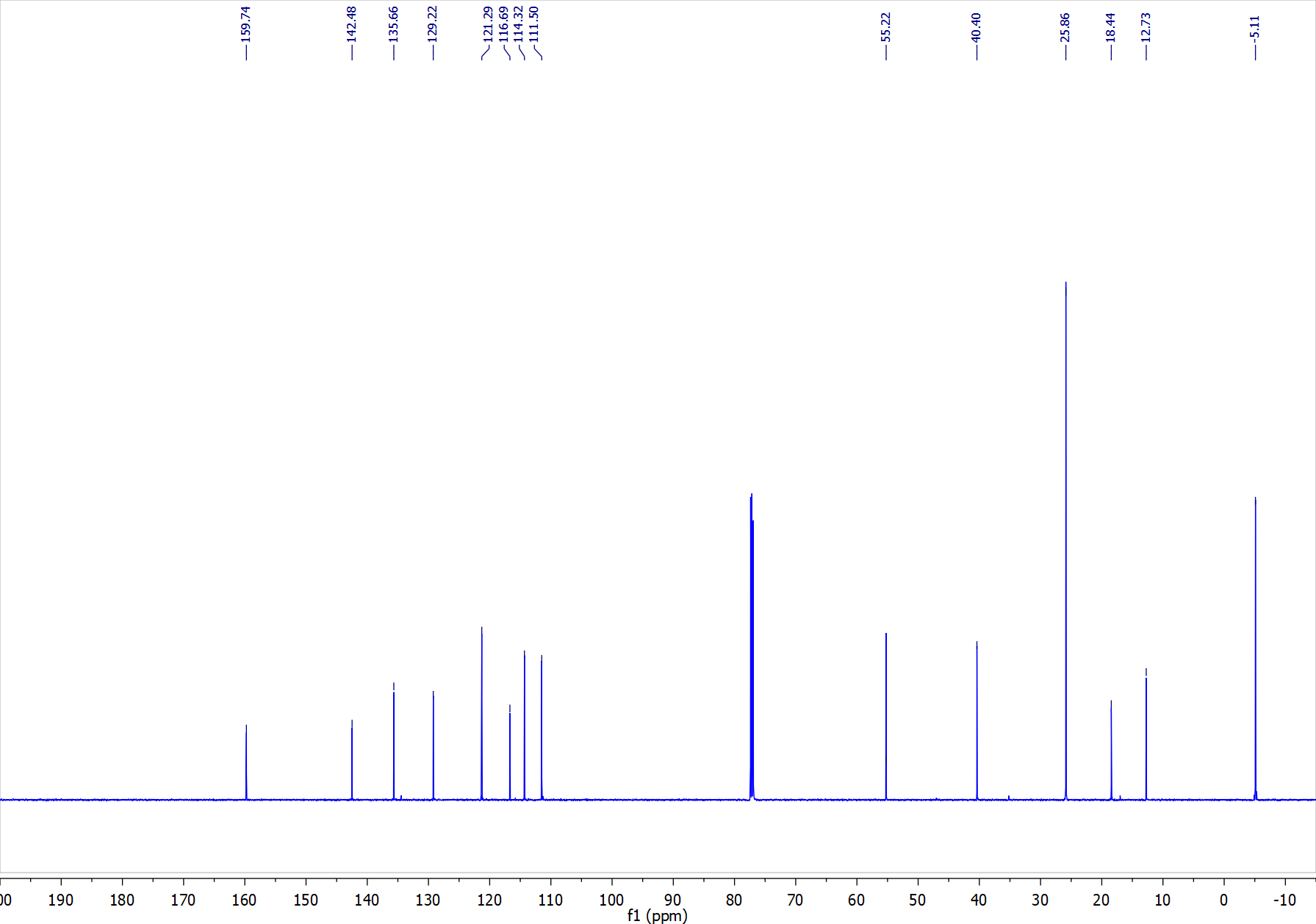
 **Supplementary Figure 72.** ^13^C NMR spectrum of **18**


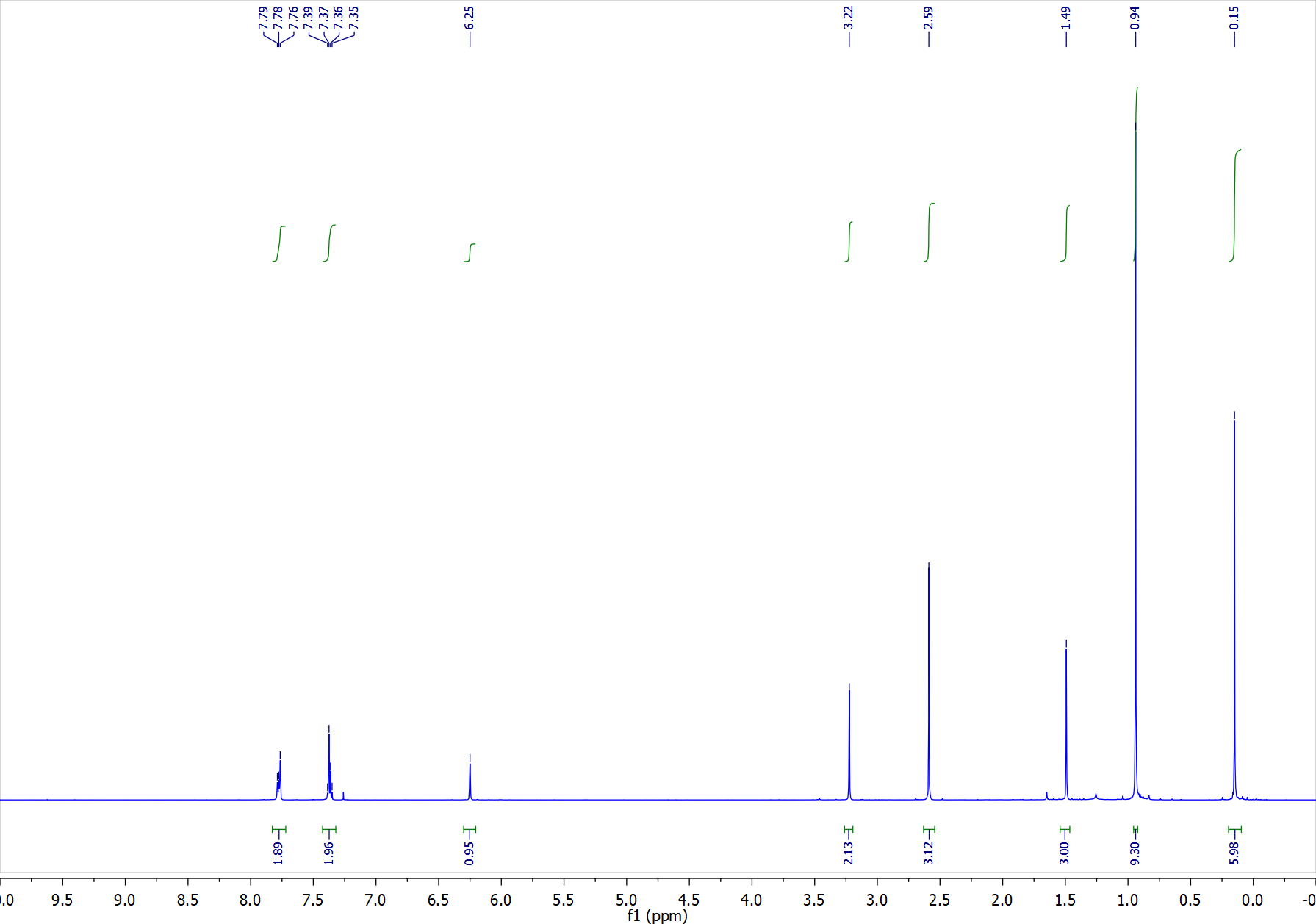
**Supplementary Figure 73.** ^1^H NMR spectrum of **19**
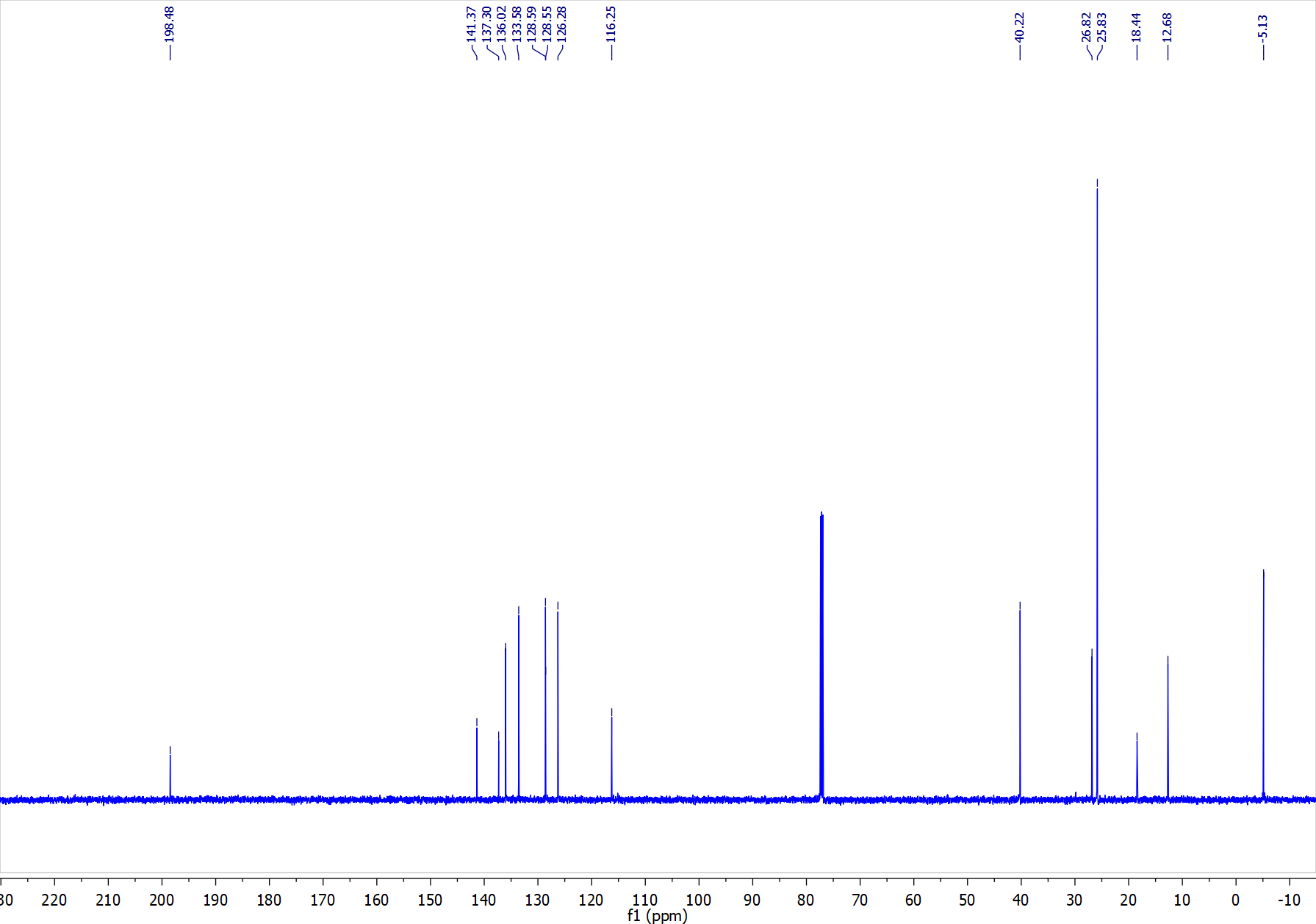
 **Supplementary Figure 74.** ^13^C NMR spectrum of **19**


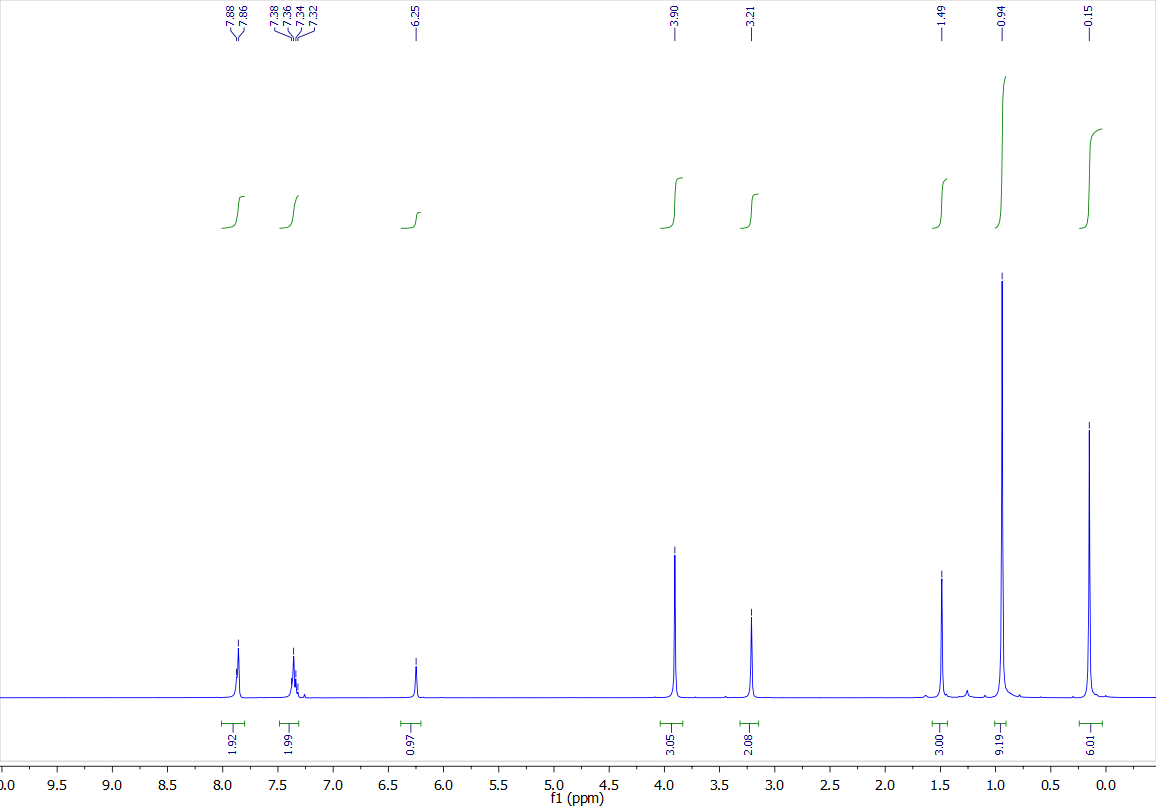
**Supplementary Figure 75.** ^1^H NMR spectrum of **20**
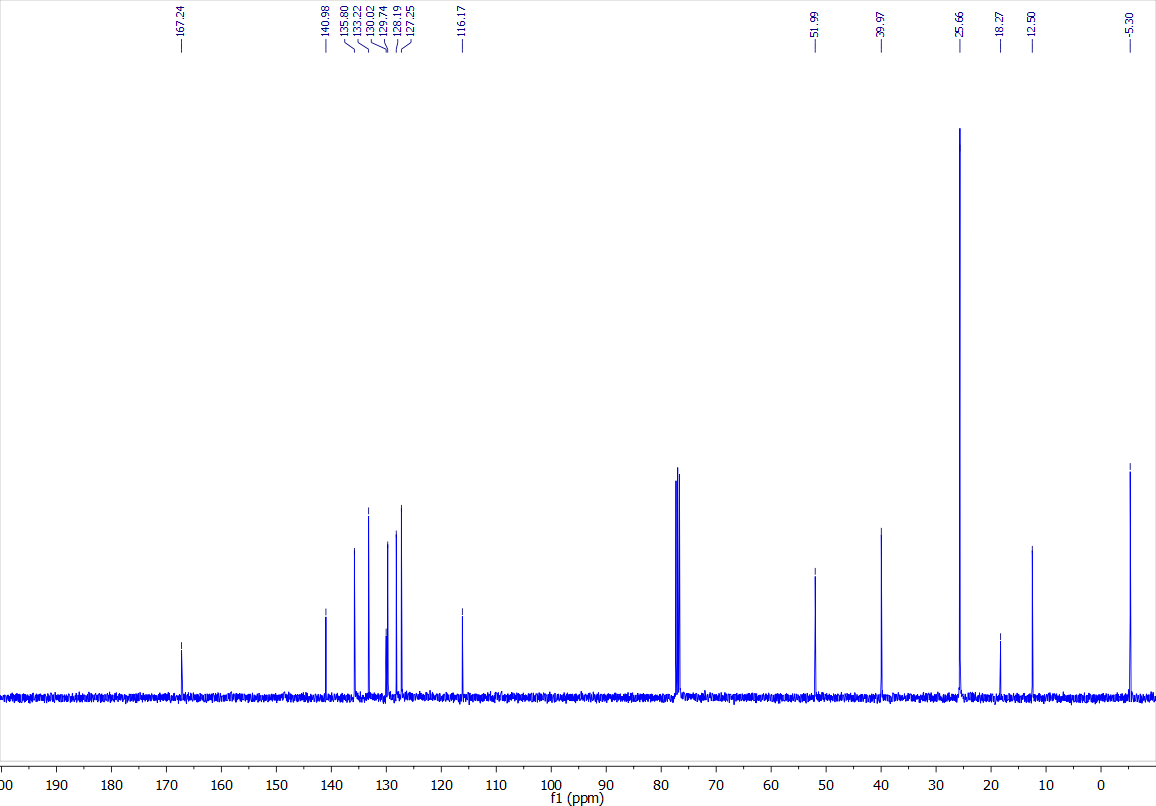
 **Supplementary Figure 76.** ^13^C NMR spectrum of **20**


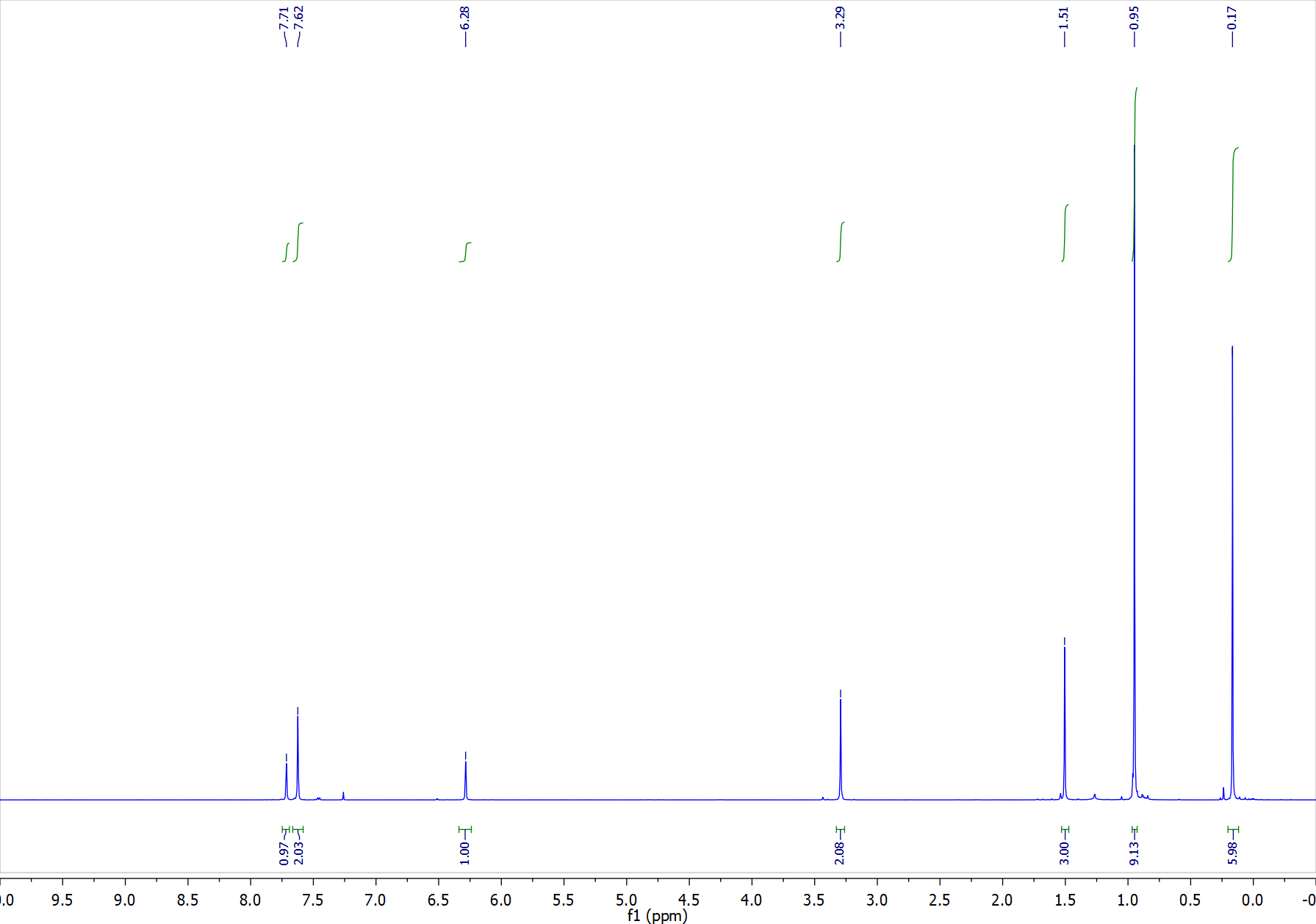
**Supplementary Figure 77.** ^1^H NMR spectrum of **21**


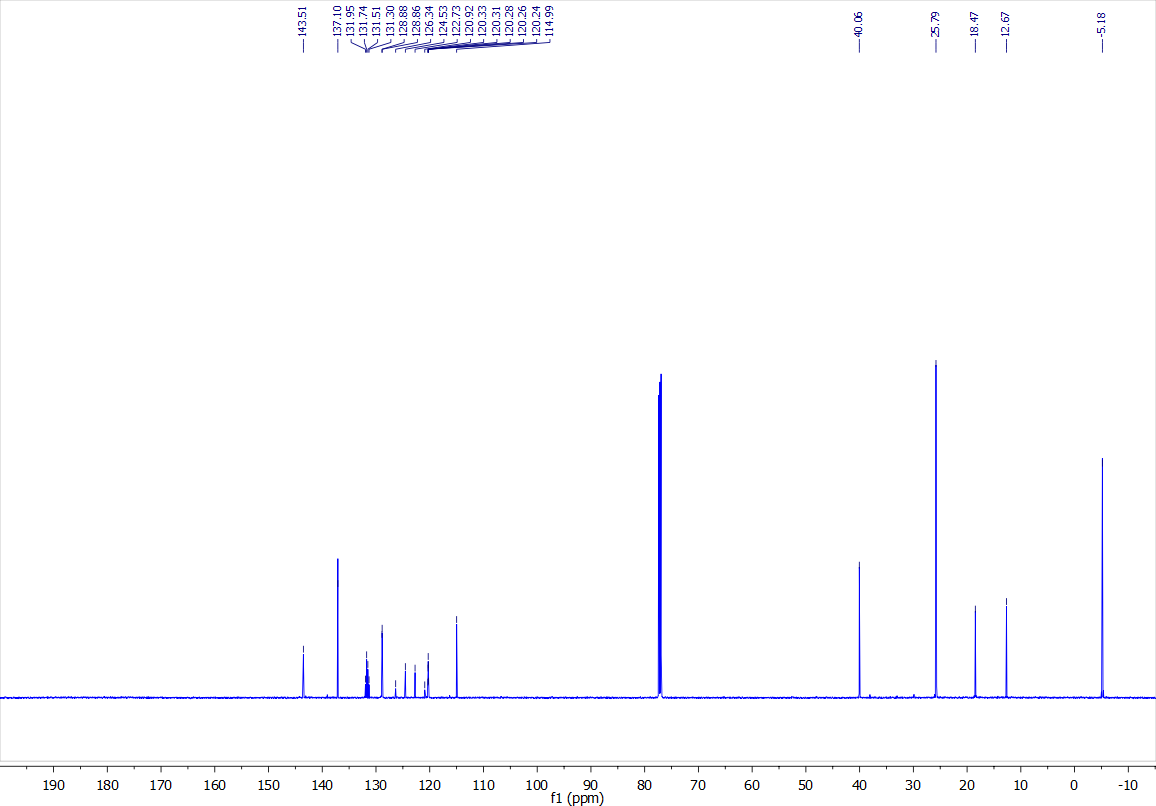
**Supplementary Figure 78.** ^13^C NMR spectrum of **21**
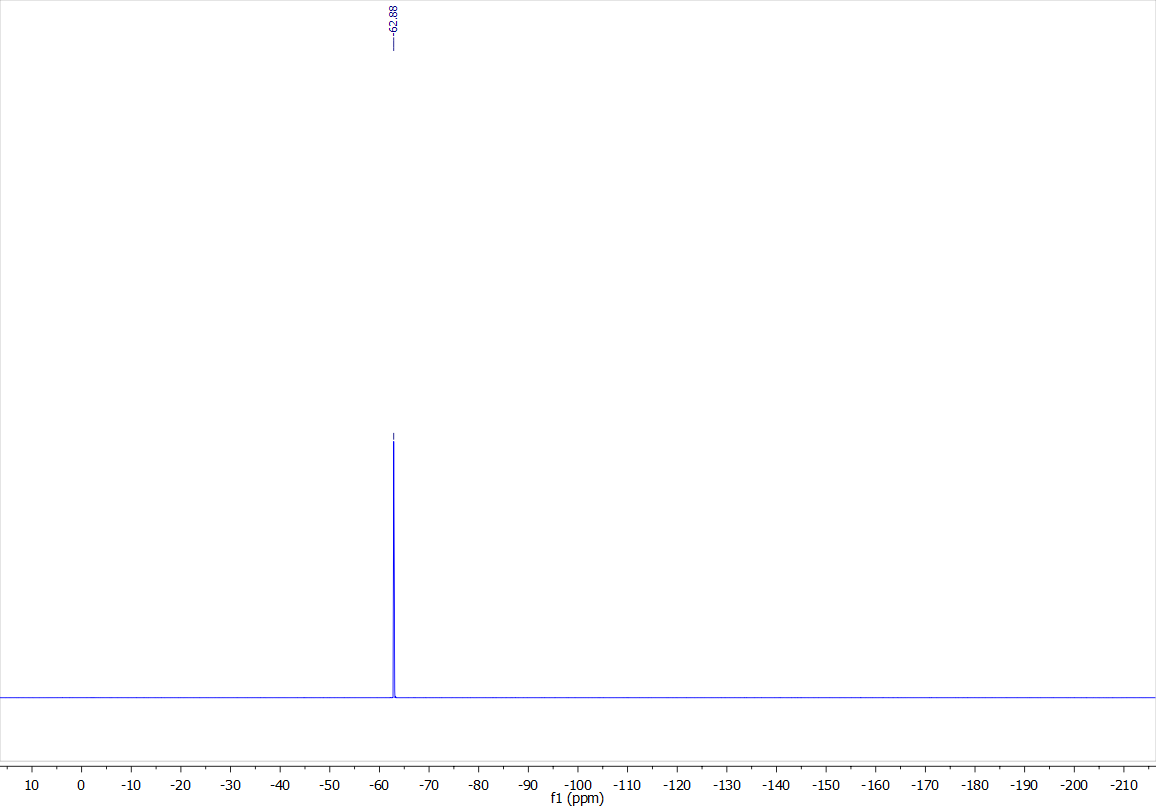
 **Supplementary Figure 79.** ^19^F NMR spectrum of **2****1**


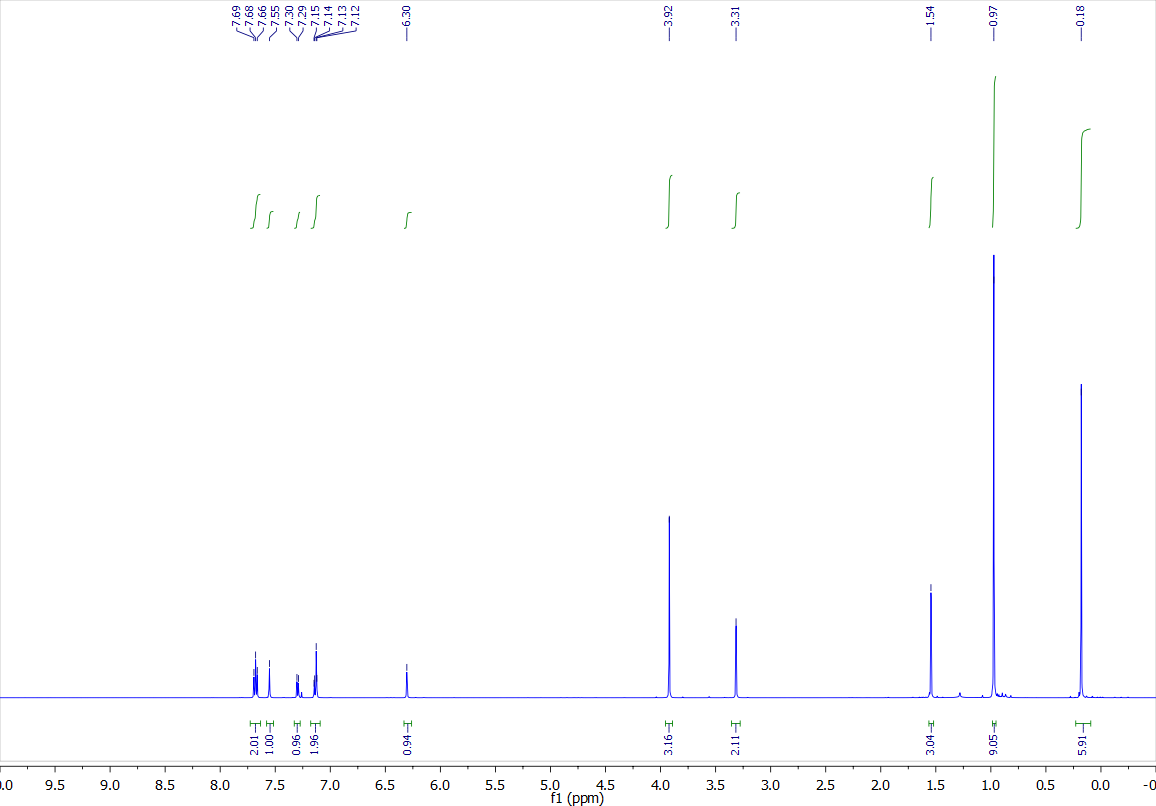
**Supplementary Figure 80.** ^1^H NMR spectrum of **22**
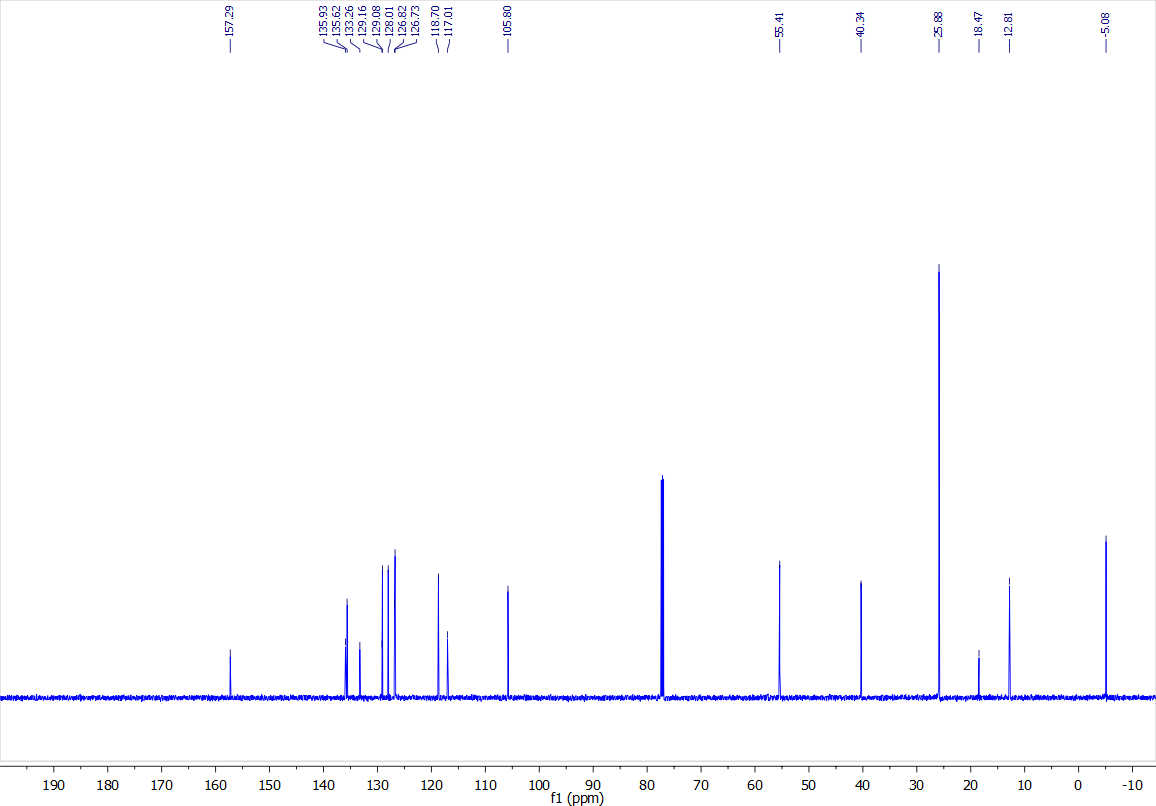
 **Supplementary Figure 81.** ^13^C NMR spectrum of **22**


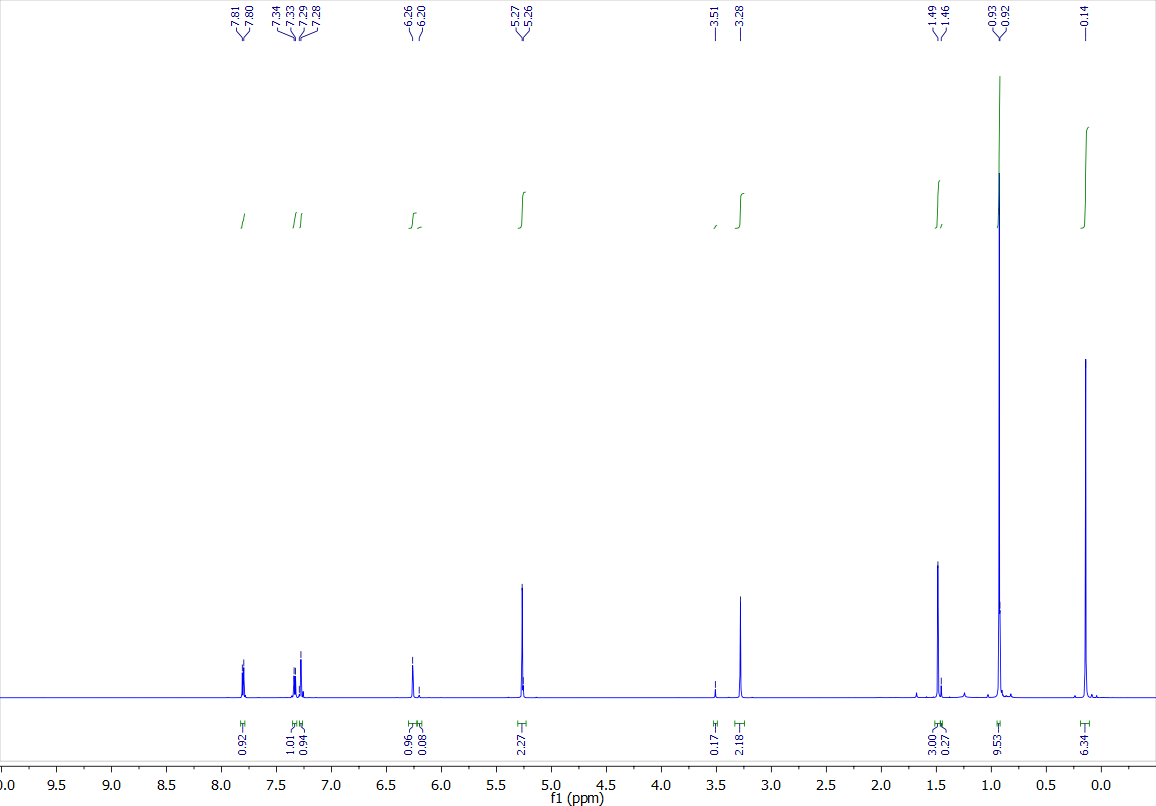
**Supplementary Figure 82.** ^1^H NMR spectrum of **23**
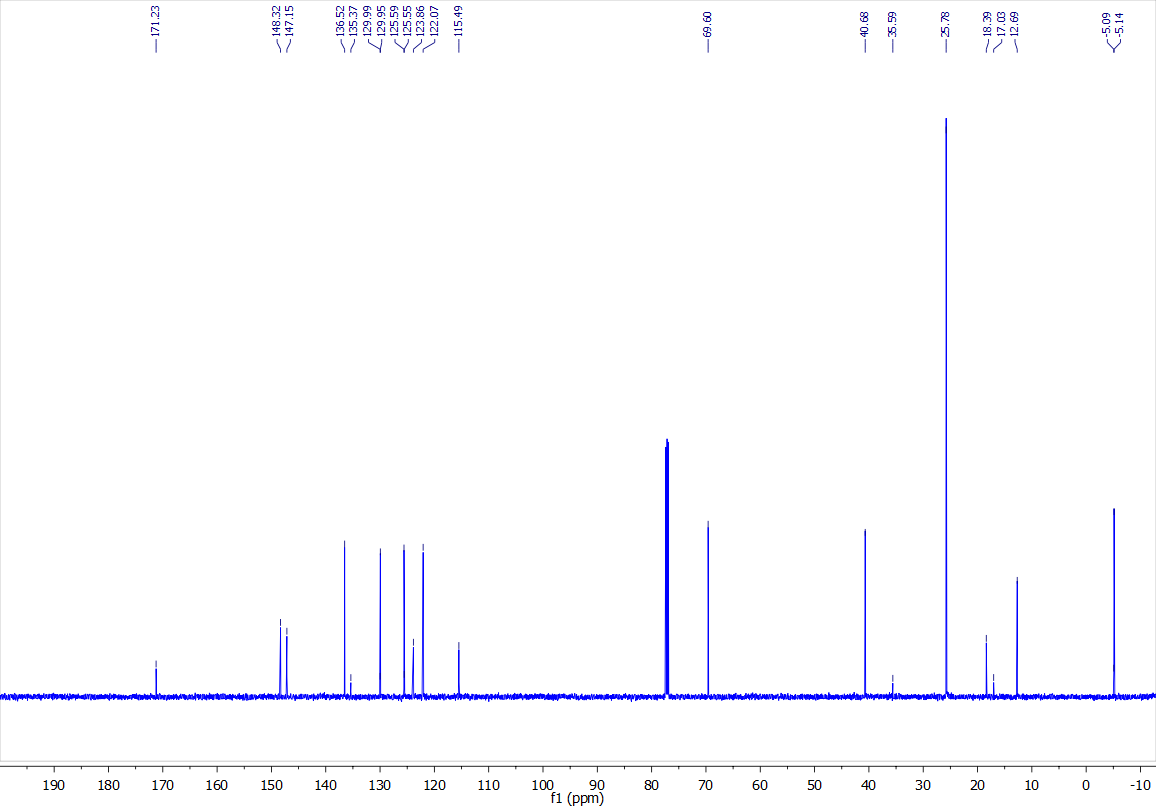
 **Supplementary Figure 83.** ^13^C NMR spectrum of **23**


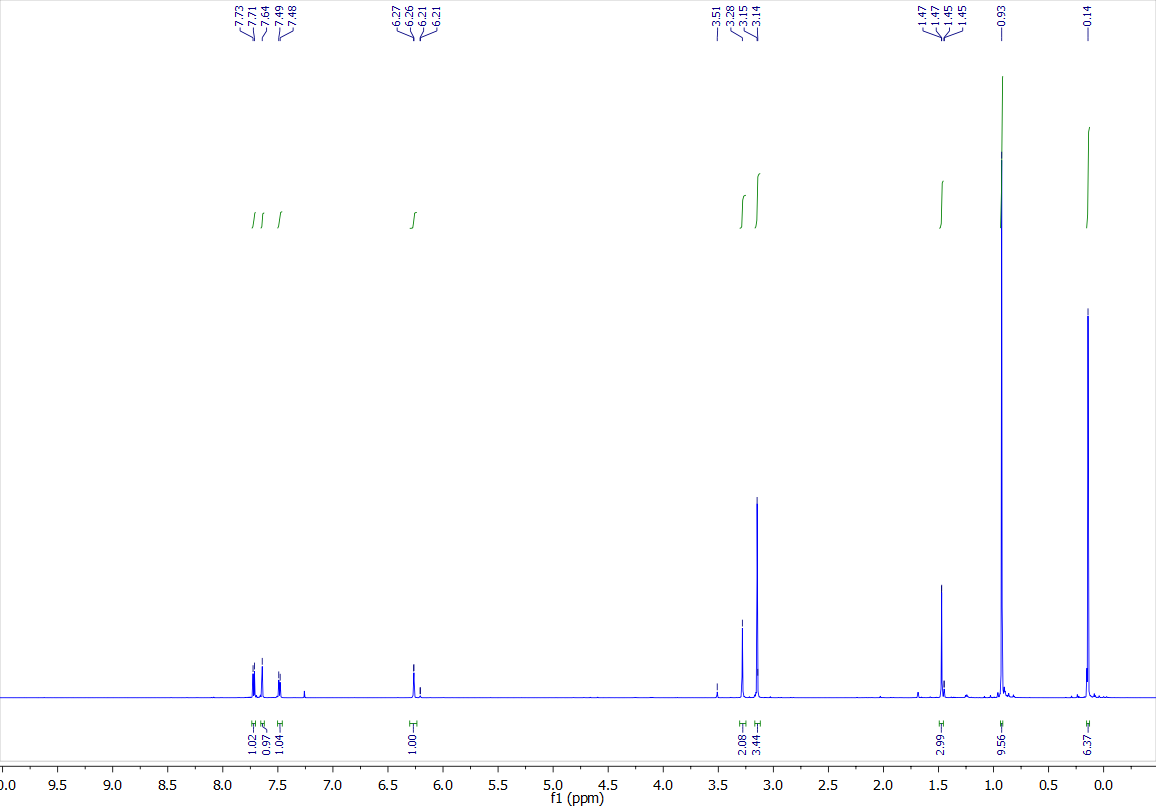
**Supplementary Figure 84.** ^1^H NMR spectrum of **24**
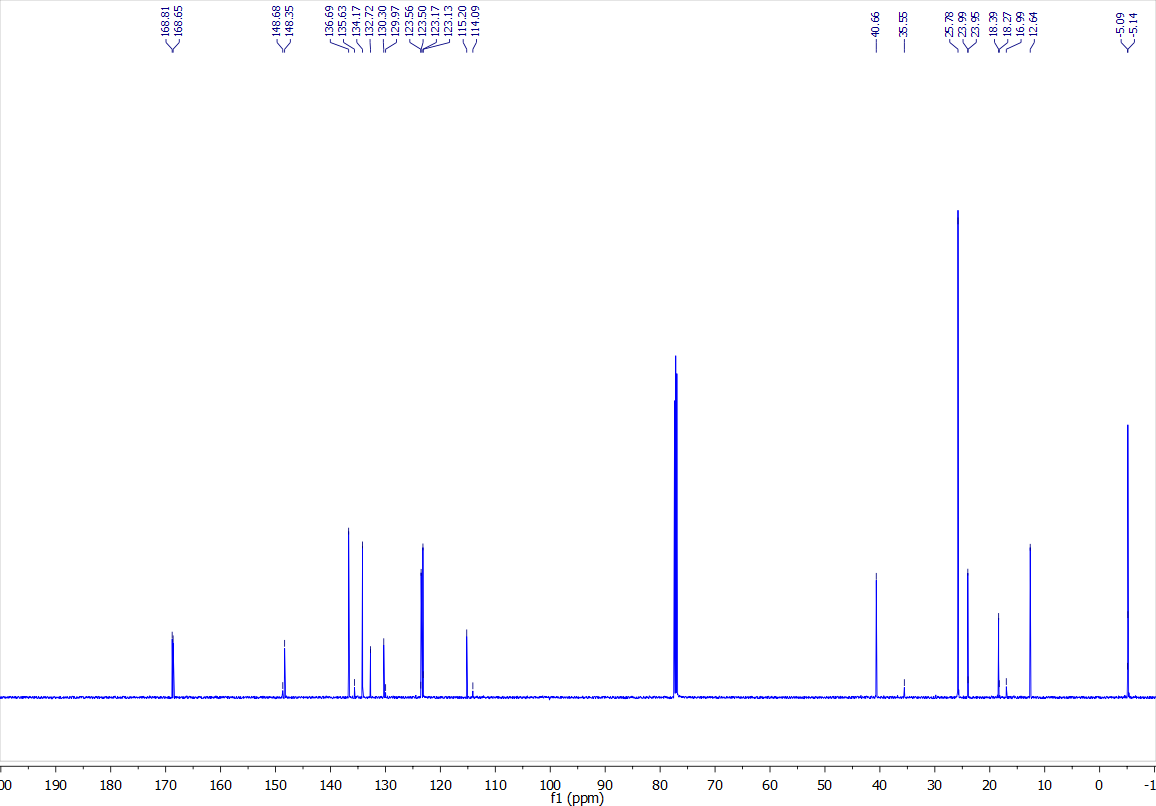
 **Supplementary Figure 85.** ^13^C NMR spectrum of **24**


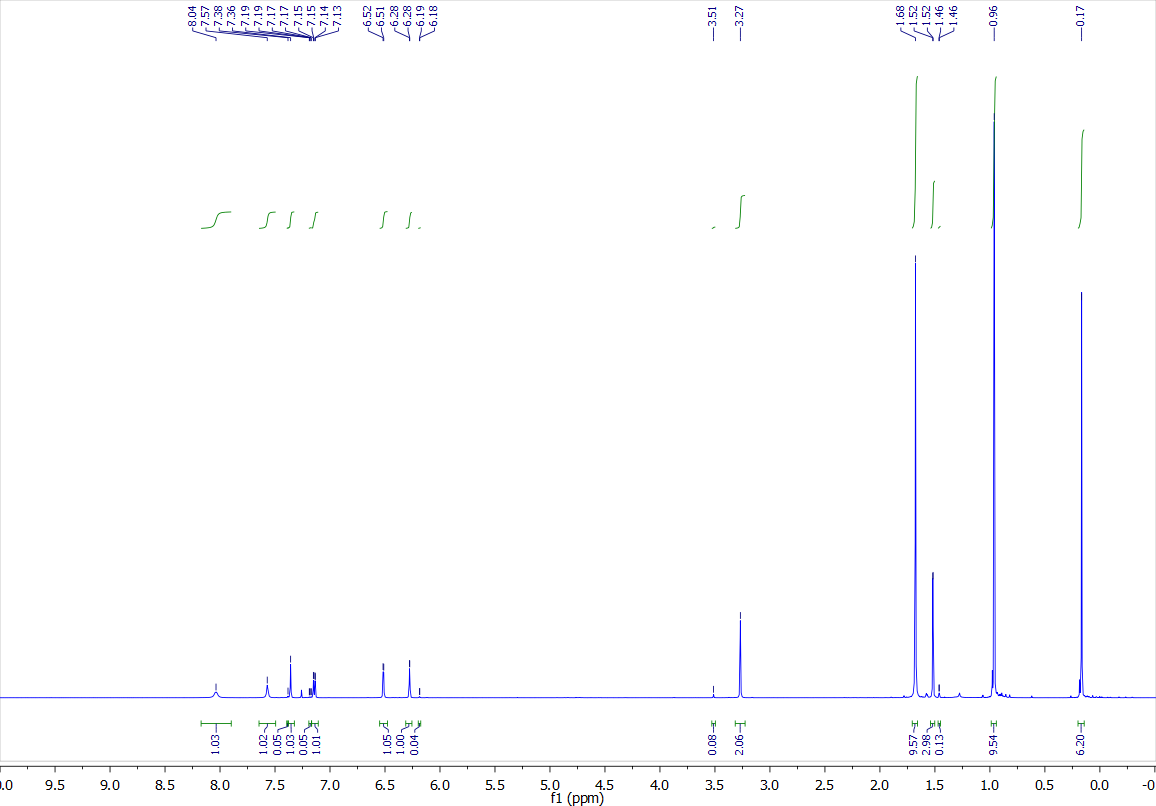
**Supplementary Figure 86.** ^1^H NMR spectrum of **25**
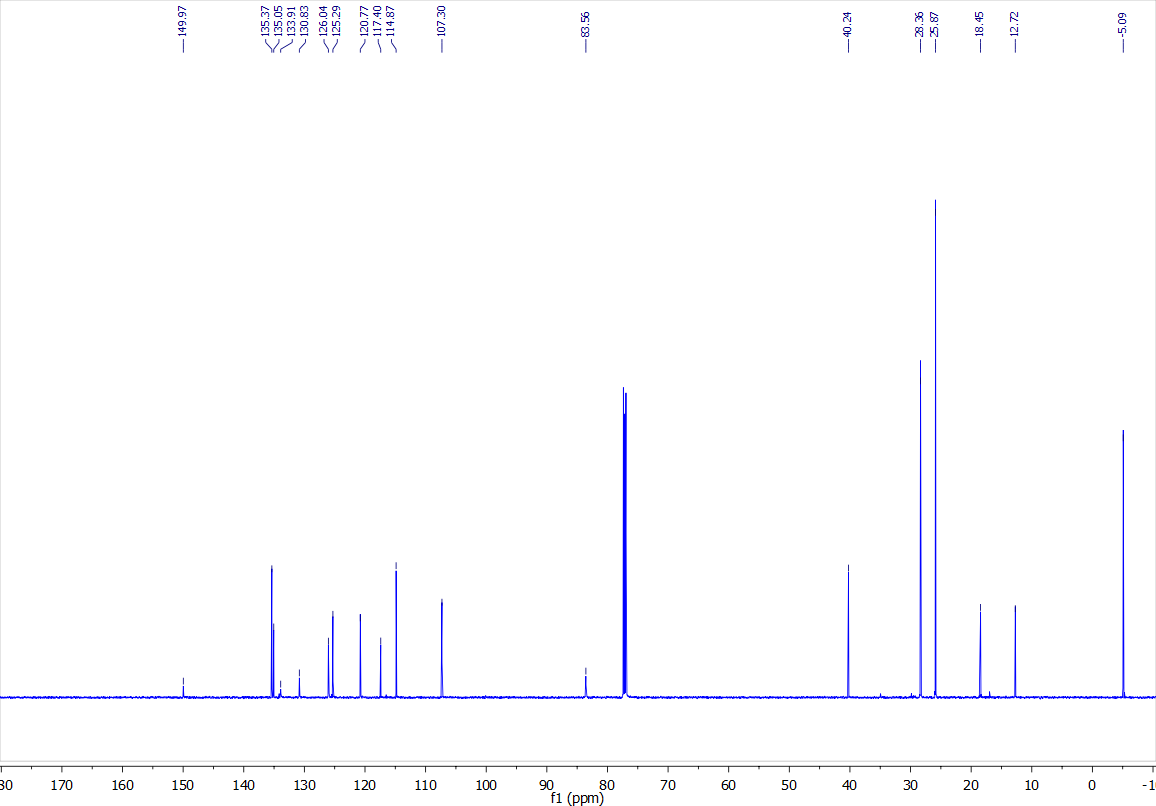
 **Supplementary Figure 87.** ^13^C NMR spectrum of **25**


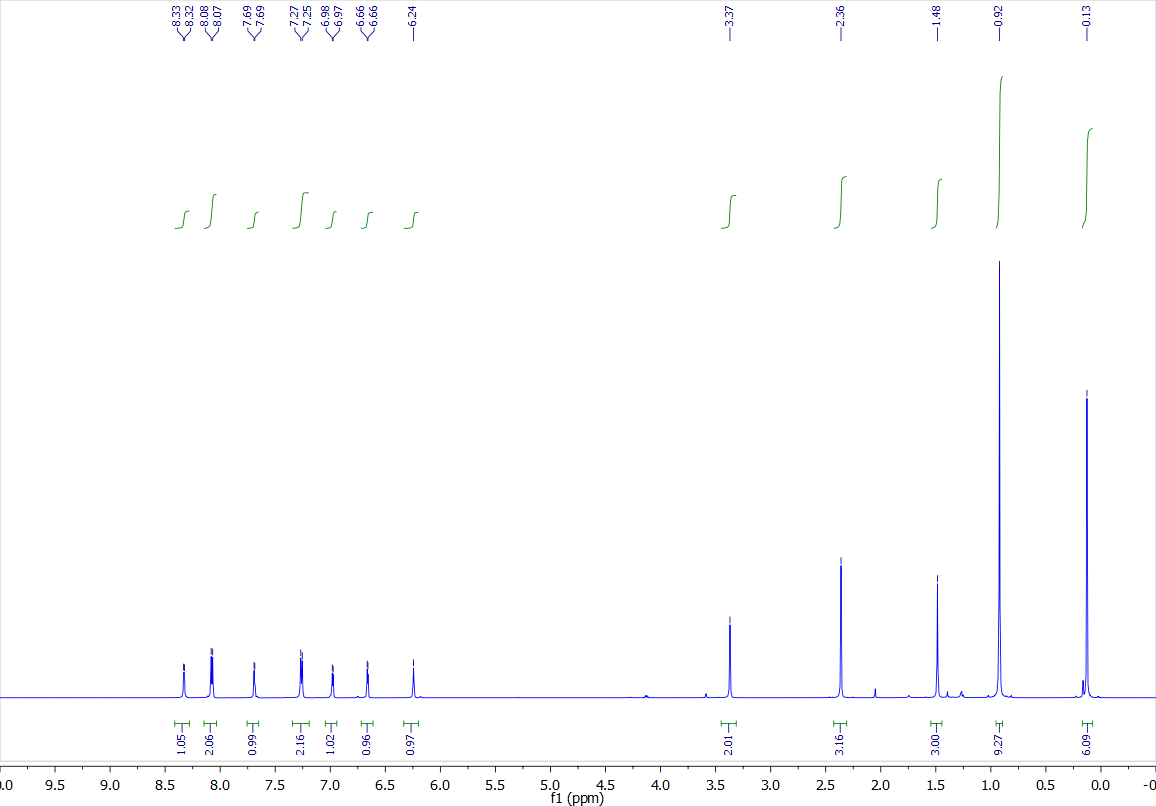
**Supplementary Figure 88.** ^1^H NMR spectrum of **26**
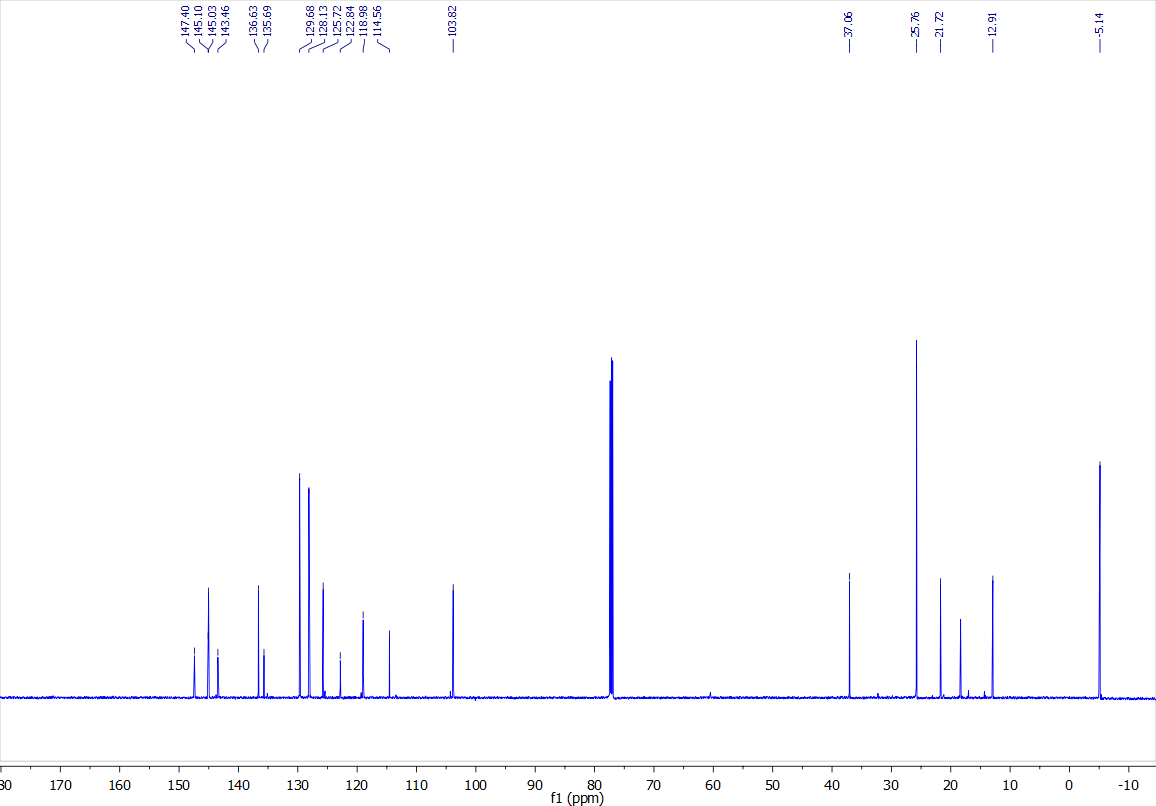
 **Supplementary Figure 89.** ^13^C NMR spectrum of **26**


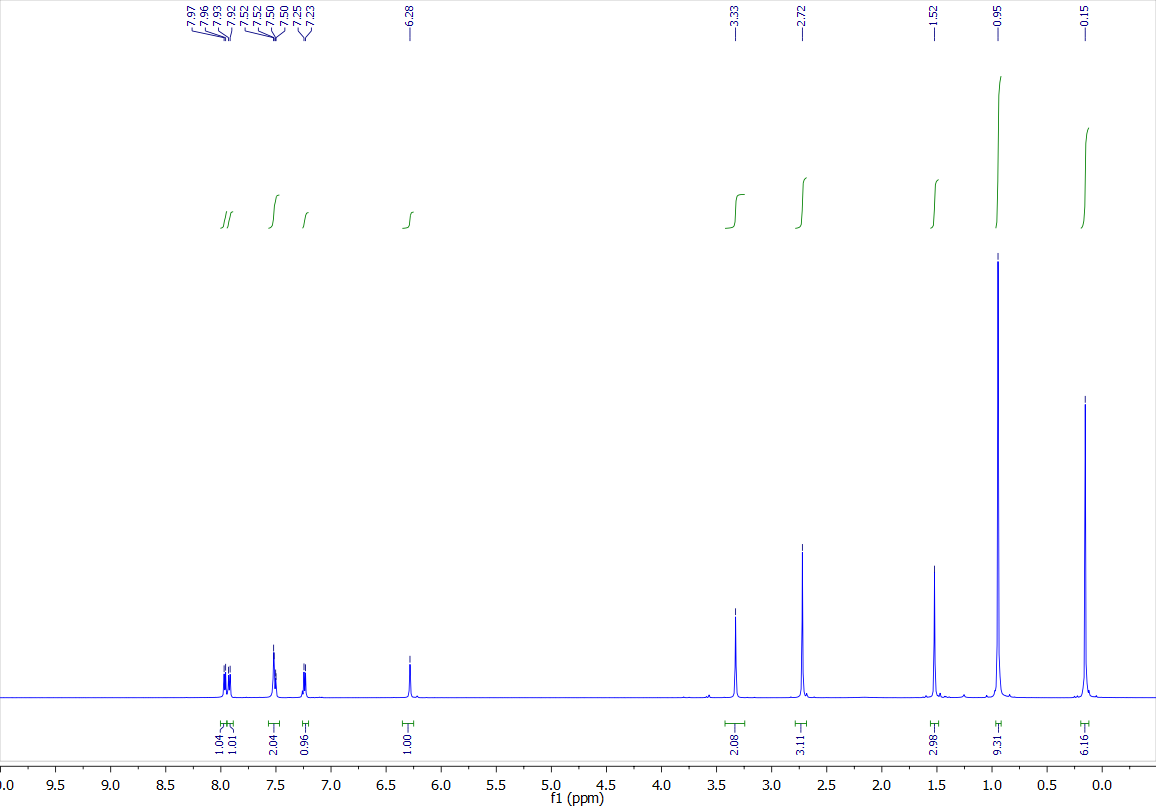
**Supplementary Figure 90.** ^1^H NMR spectrum of **27**
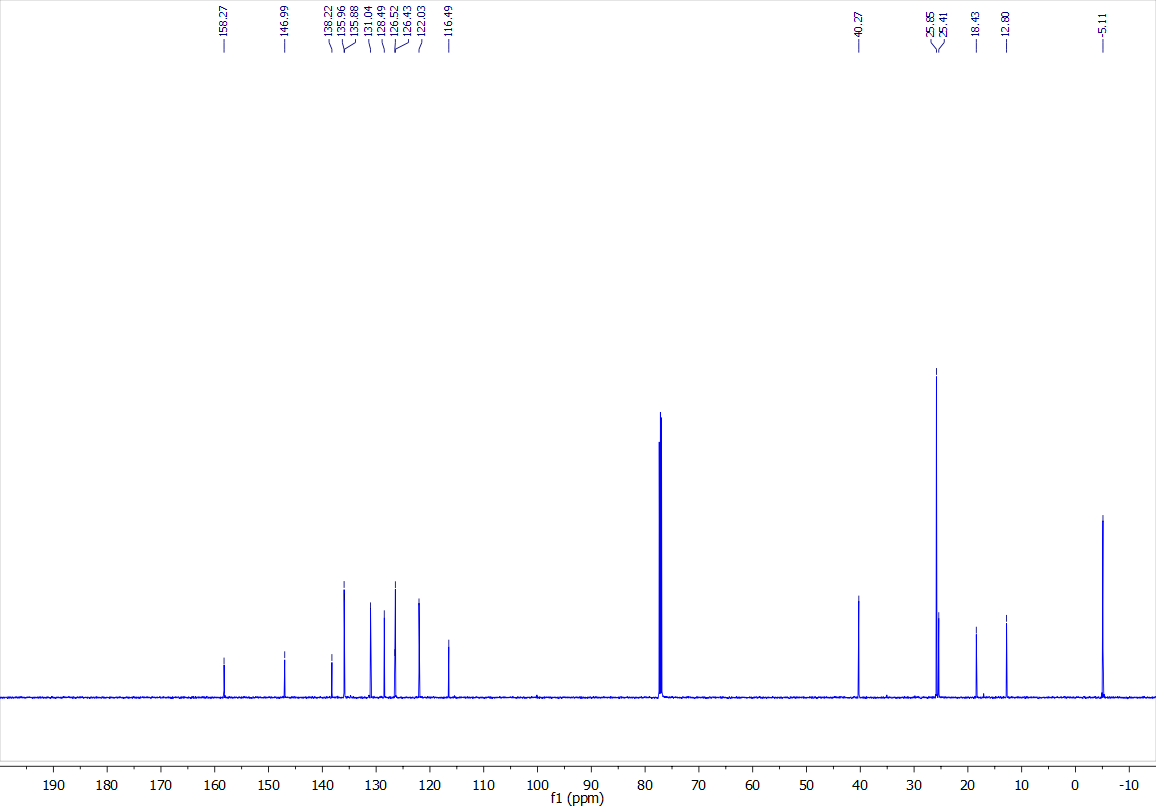
 **Supplementary Figure 91.** ^13^C NMR spectrum of **27**


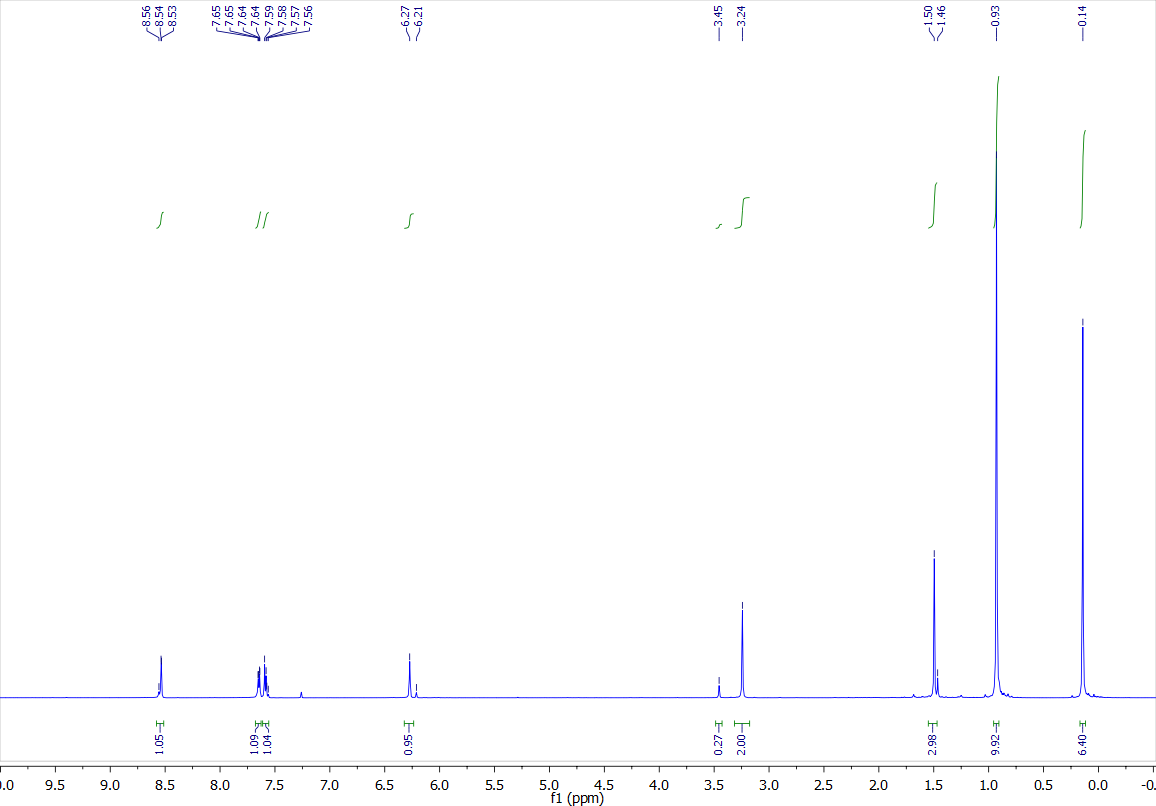
**Supplementary Figure 92.** ^1^H NMR spectrum of **28**
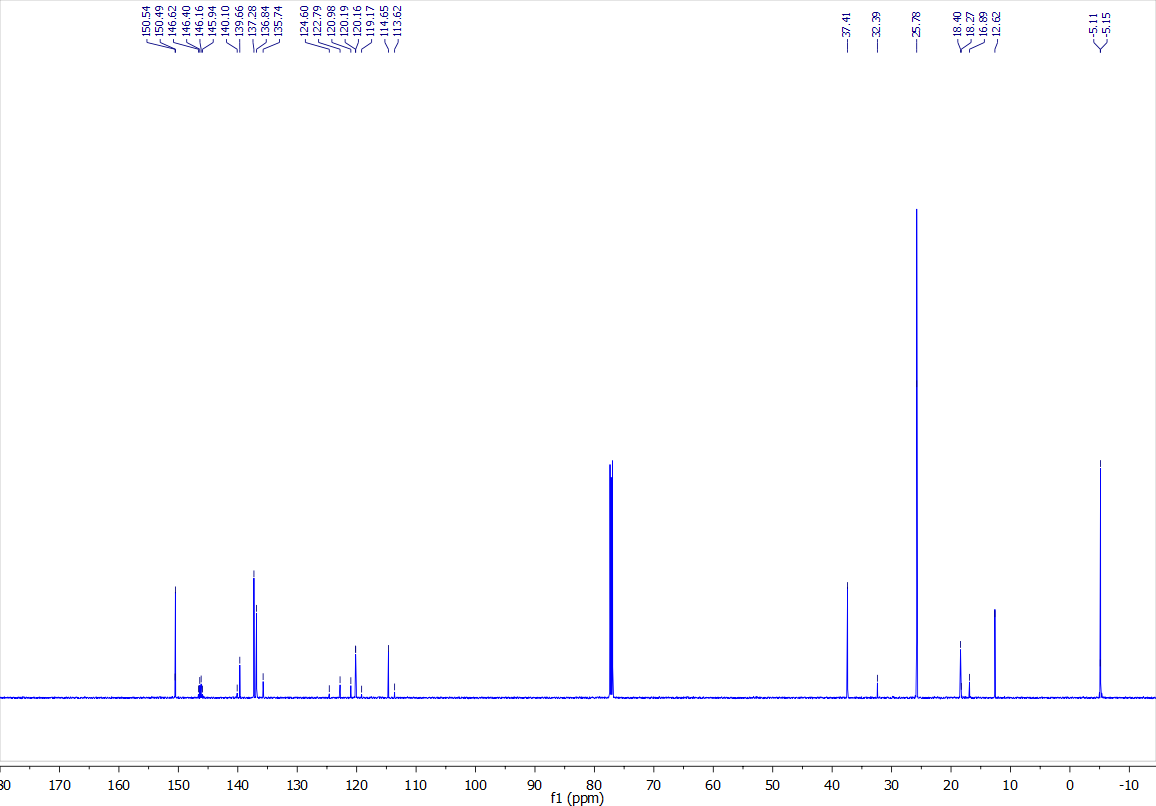


**Supplementary Figure 93.** ^13^C NMR spectrum of **28**
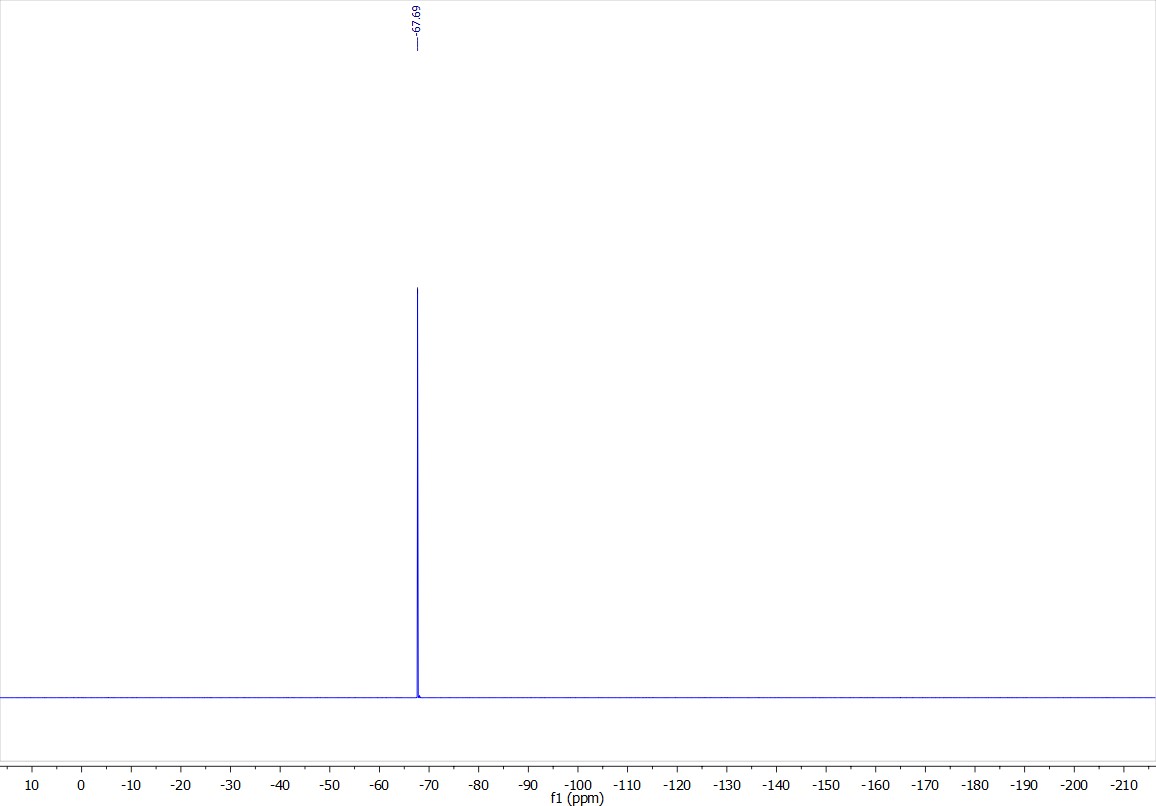
 **Supplementary Figure 94.** ^19^F NMR spectrum of **28**


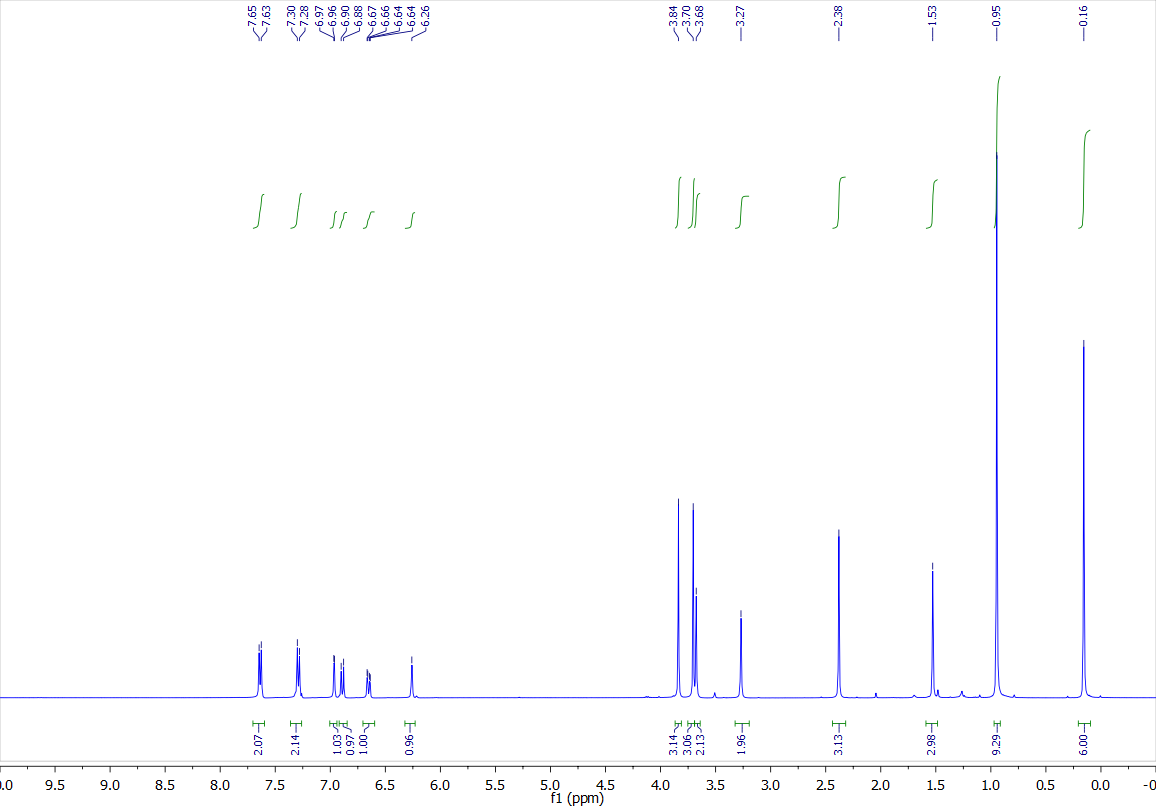
**Supplementary Figure 95.** ^1^H NMR spectrum of **29**
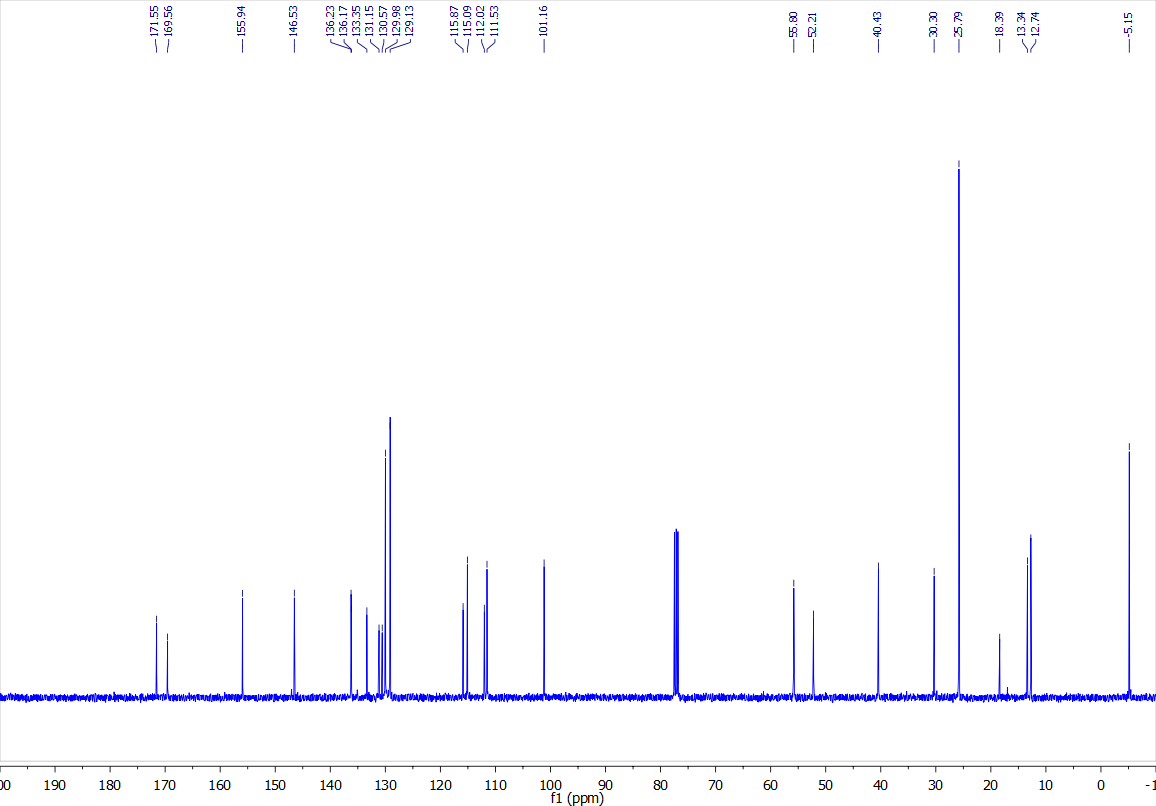
 **Supplementary Figure 96.** ^13^C NMR spectrum of **29**


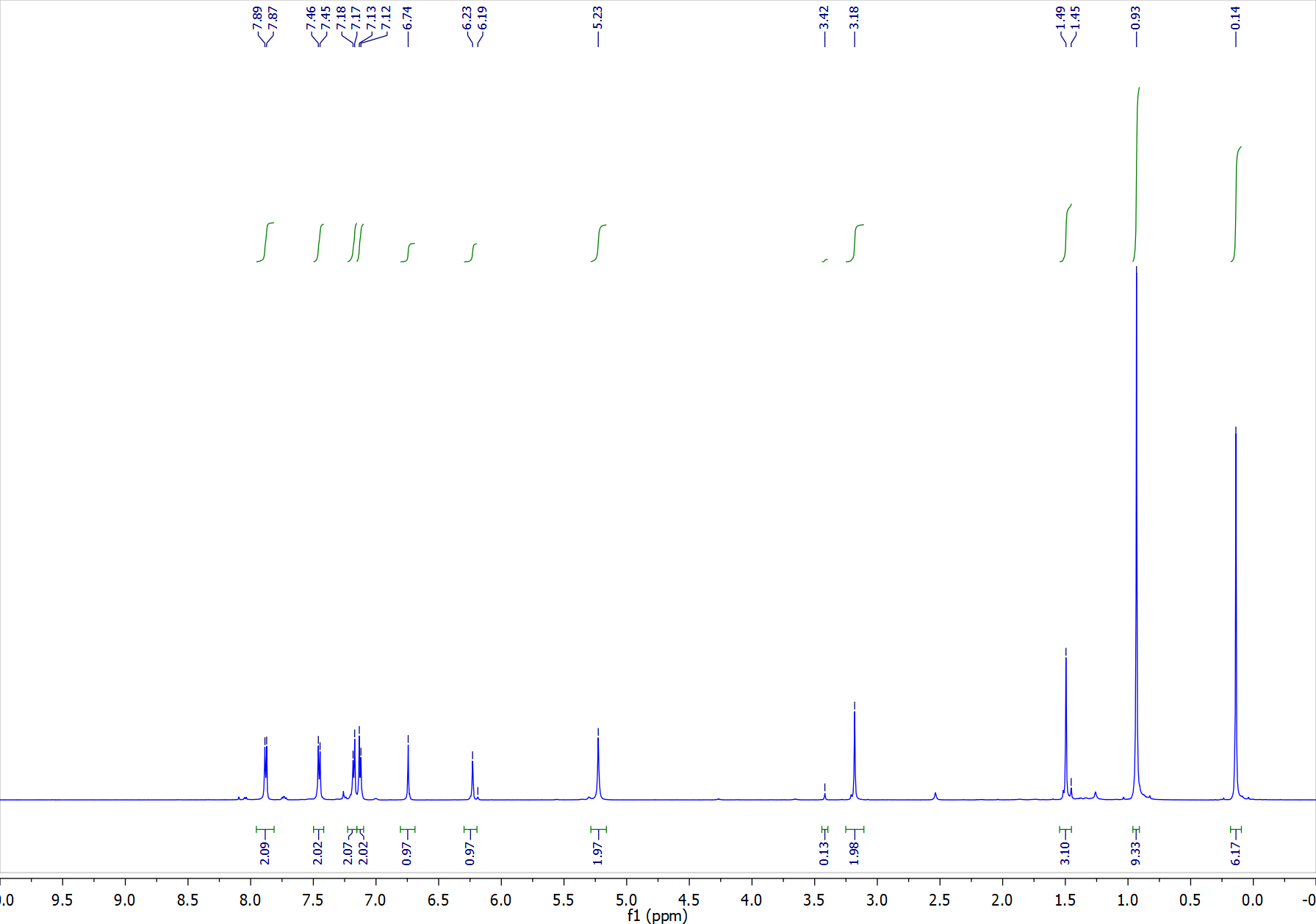
**Supplementary Figure 97.** ^1^H NMR spectrum of **30**
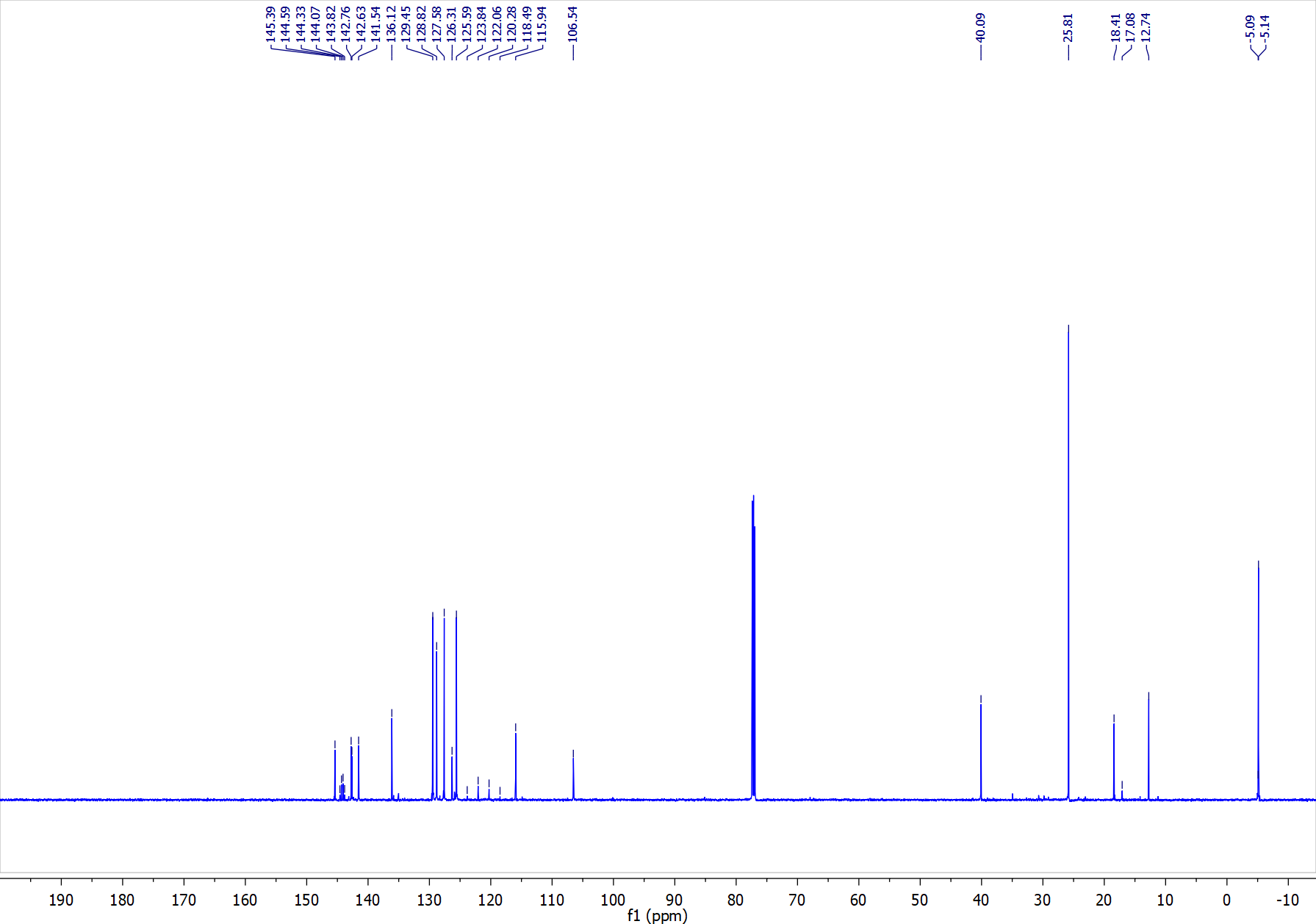
 **Supplementary Figure 98.** ^13^C NMR spectrum of **30**
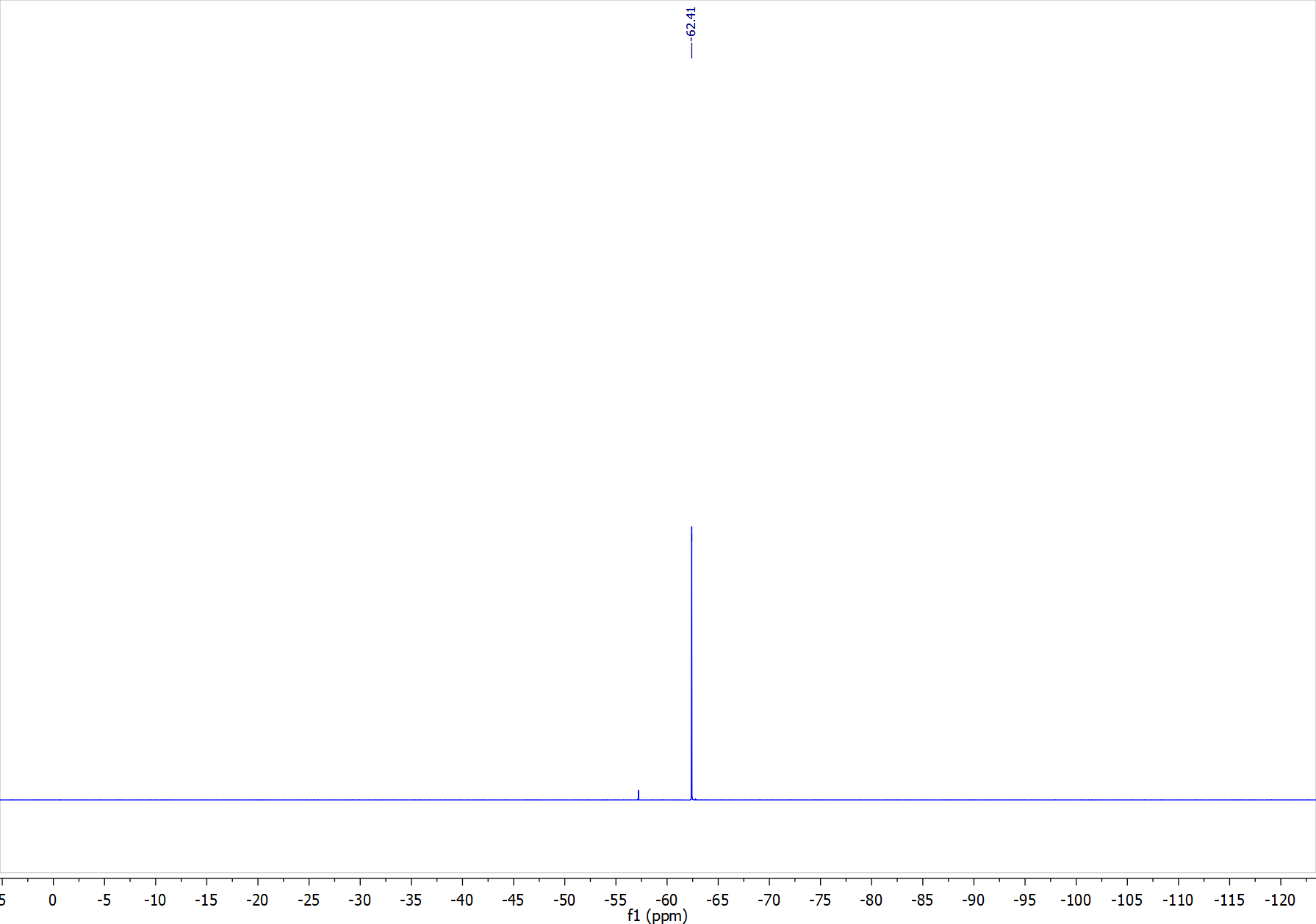
 **Supplementary Figure 99.** ^19^F NMR spectrum of **30**


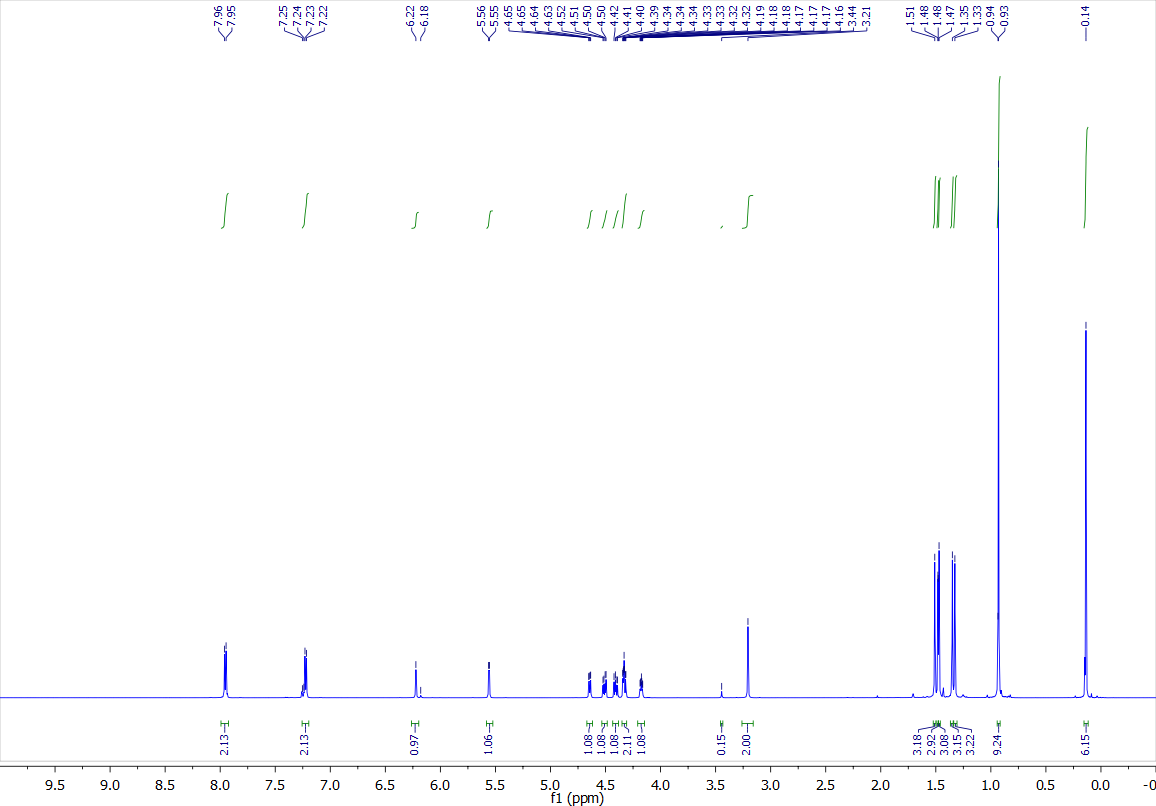
**Supplementary Figure 100.** ^1^H NMR spectrum of **31**


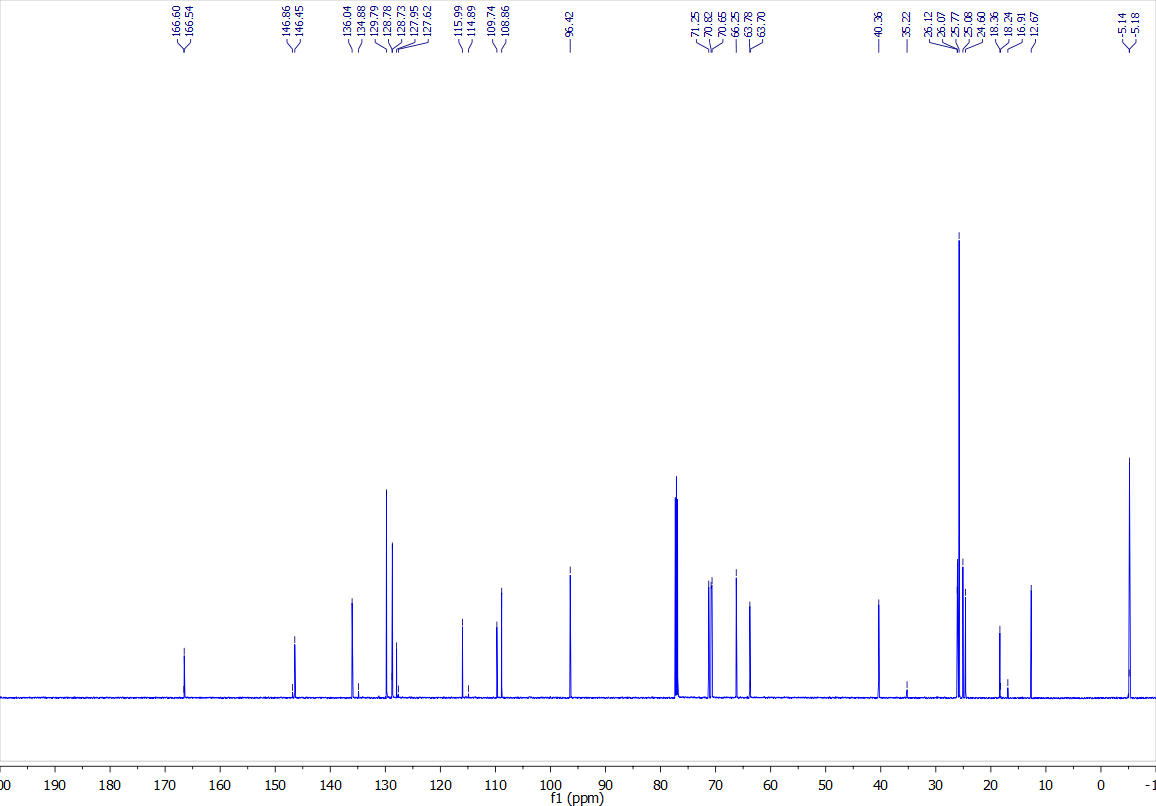
**Supplementary Figure 101.** ^13^C NMR spectrum of **31**


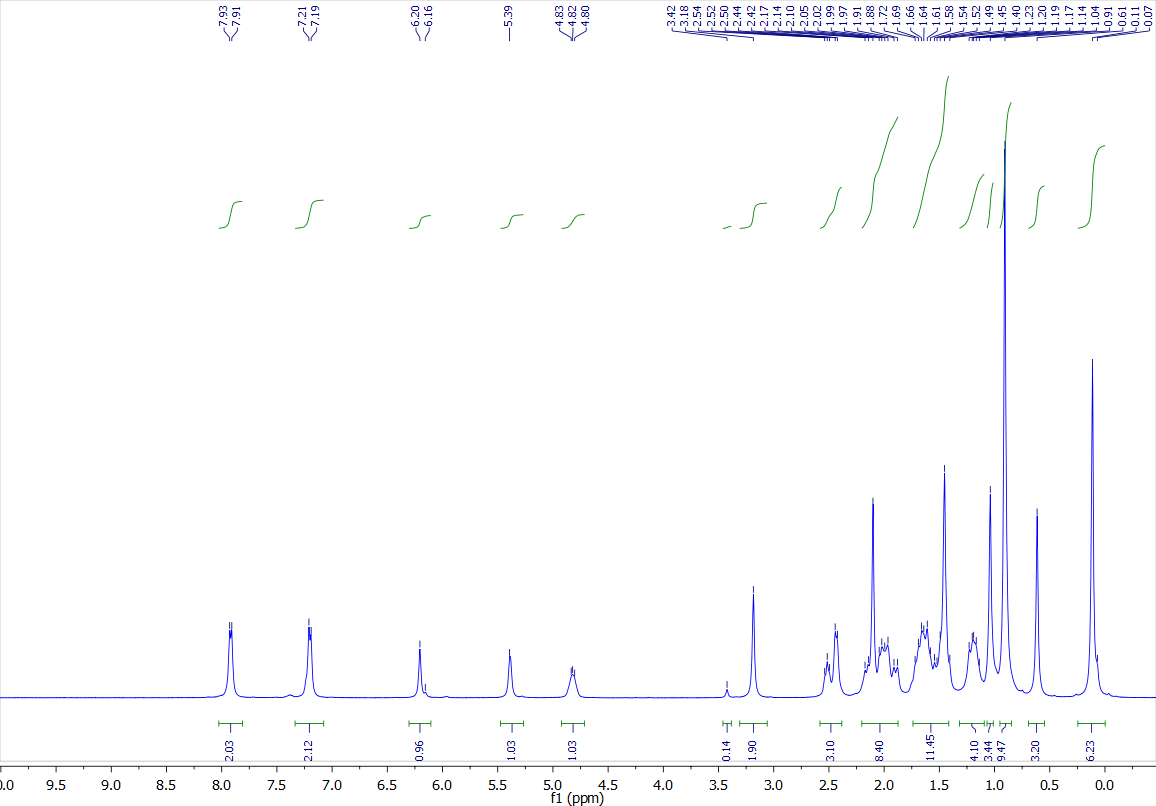
**Supplementary Figure 102.** ^1^H NMR spectrum of **32**
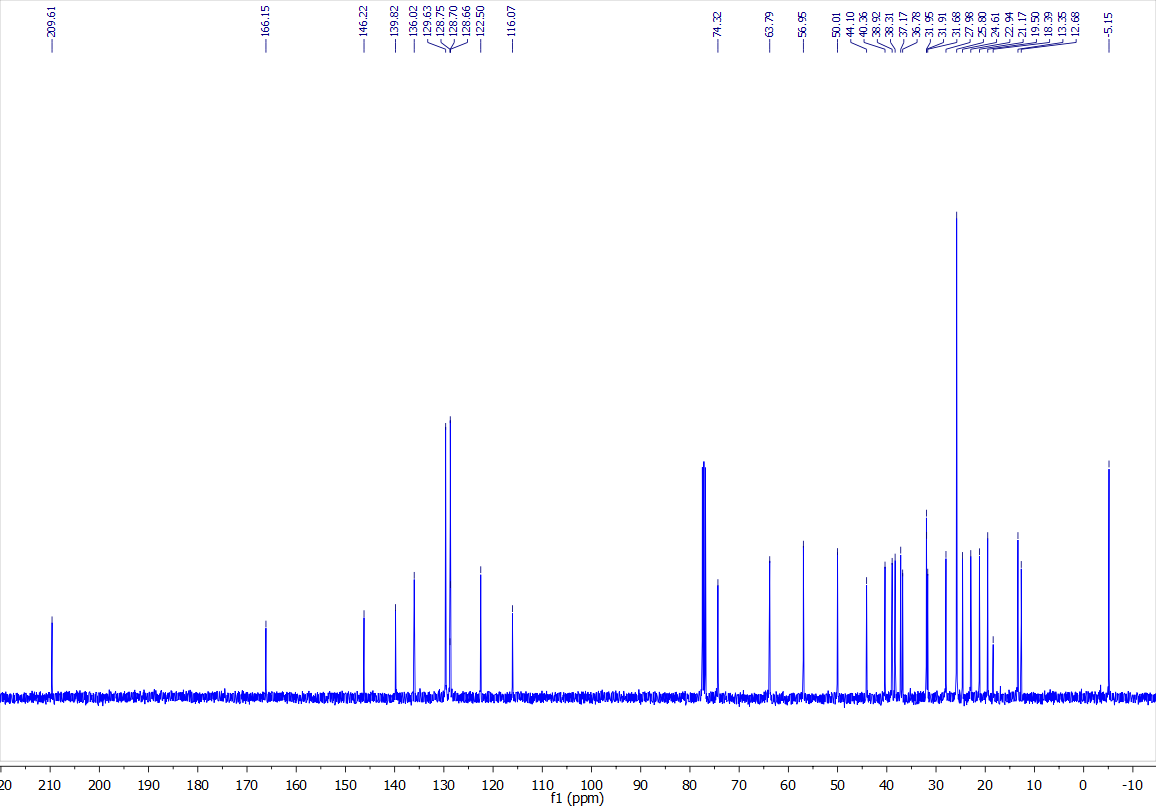
 **Supplementary Figure 103.** ^13^C NMR spectrum of **32**


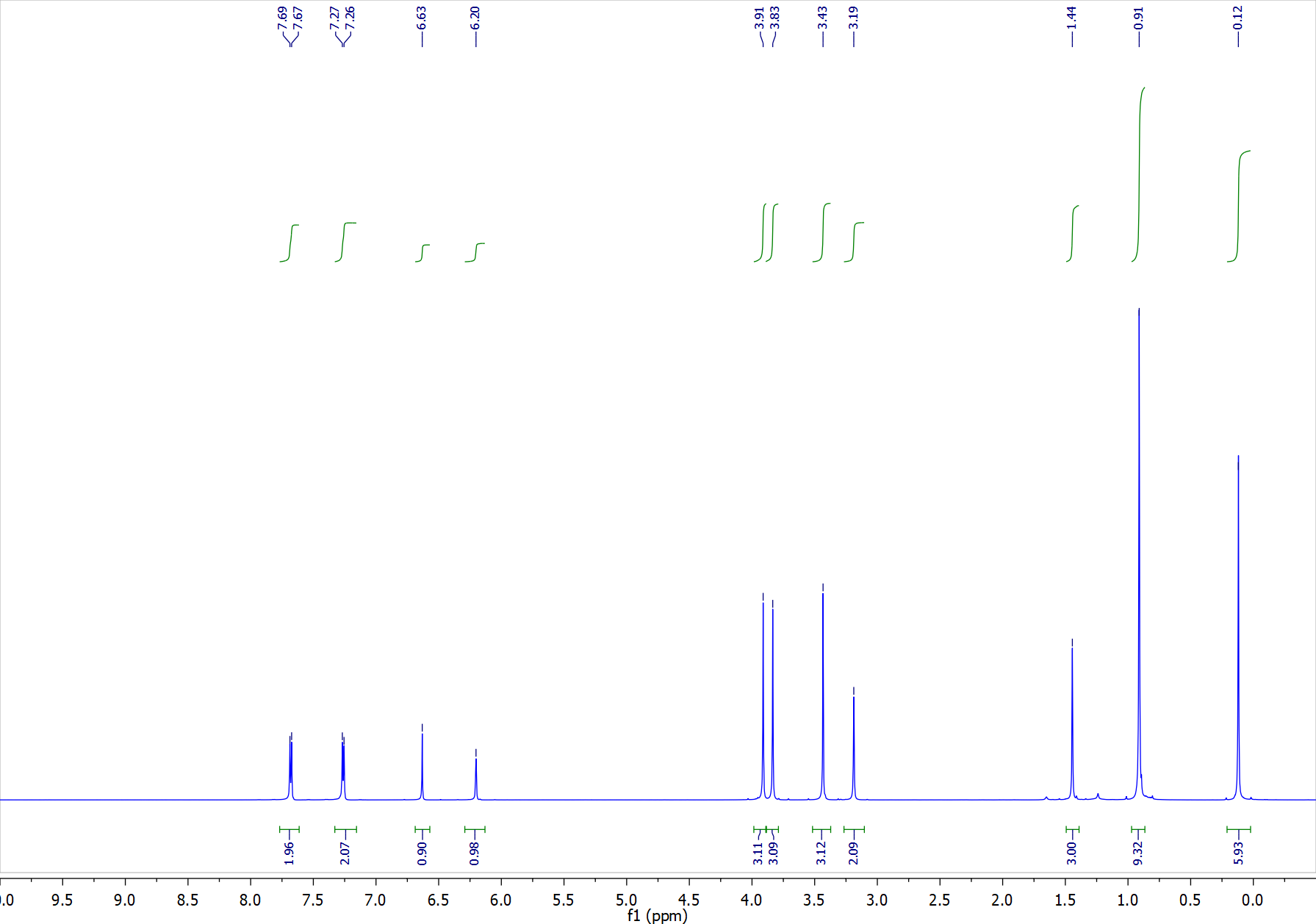
**Supplementary Figure 104.** ^1^H NMR spectrum of **33**
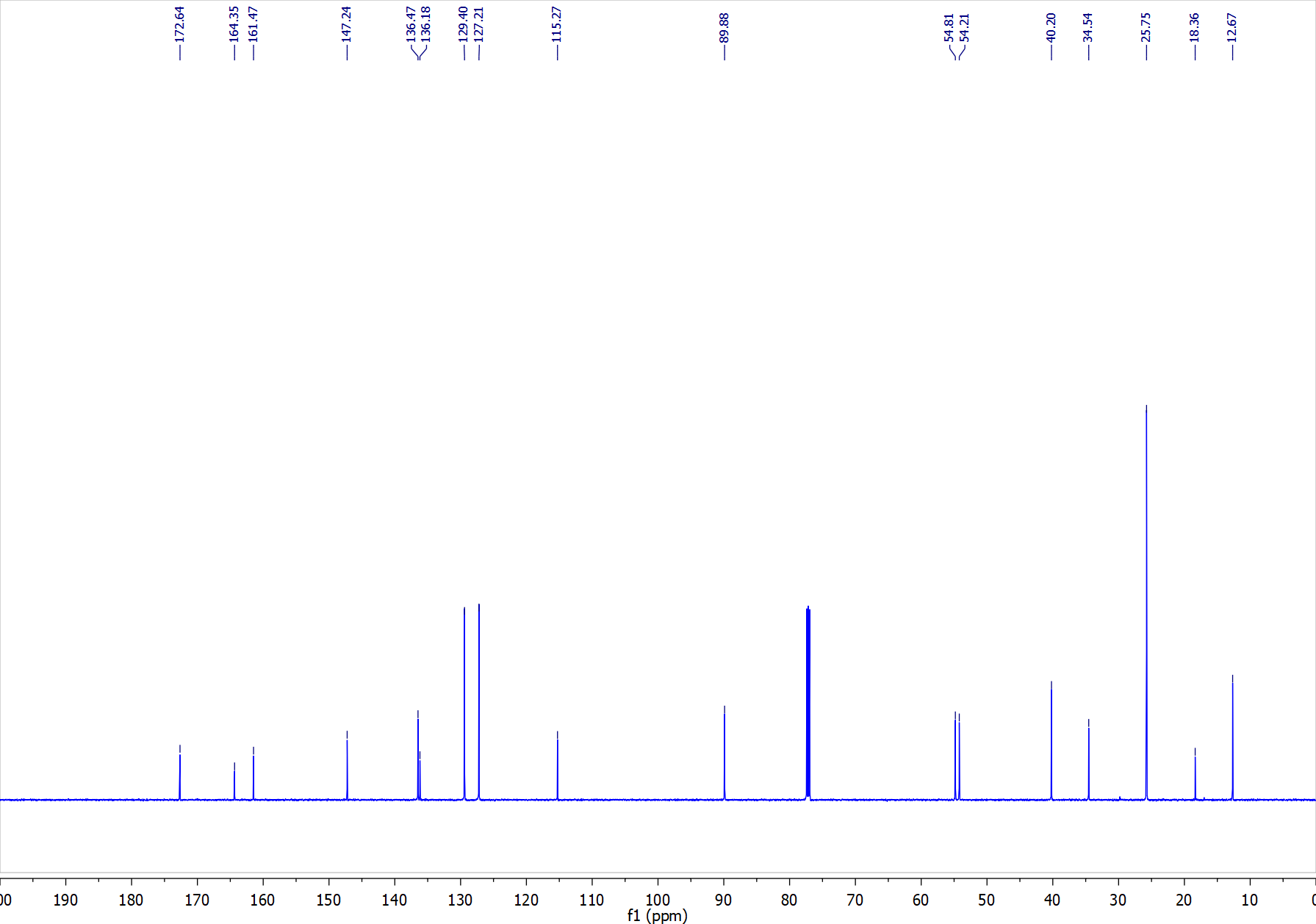
 **Supplementary Figure 105.** ^13^C NMR spectrum of **33**


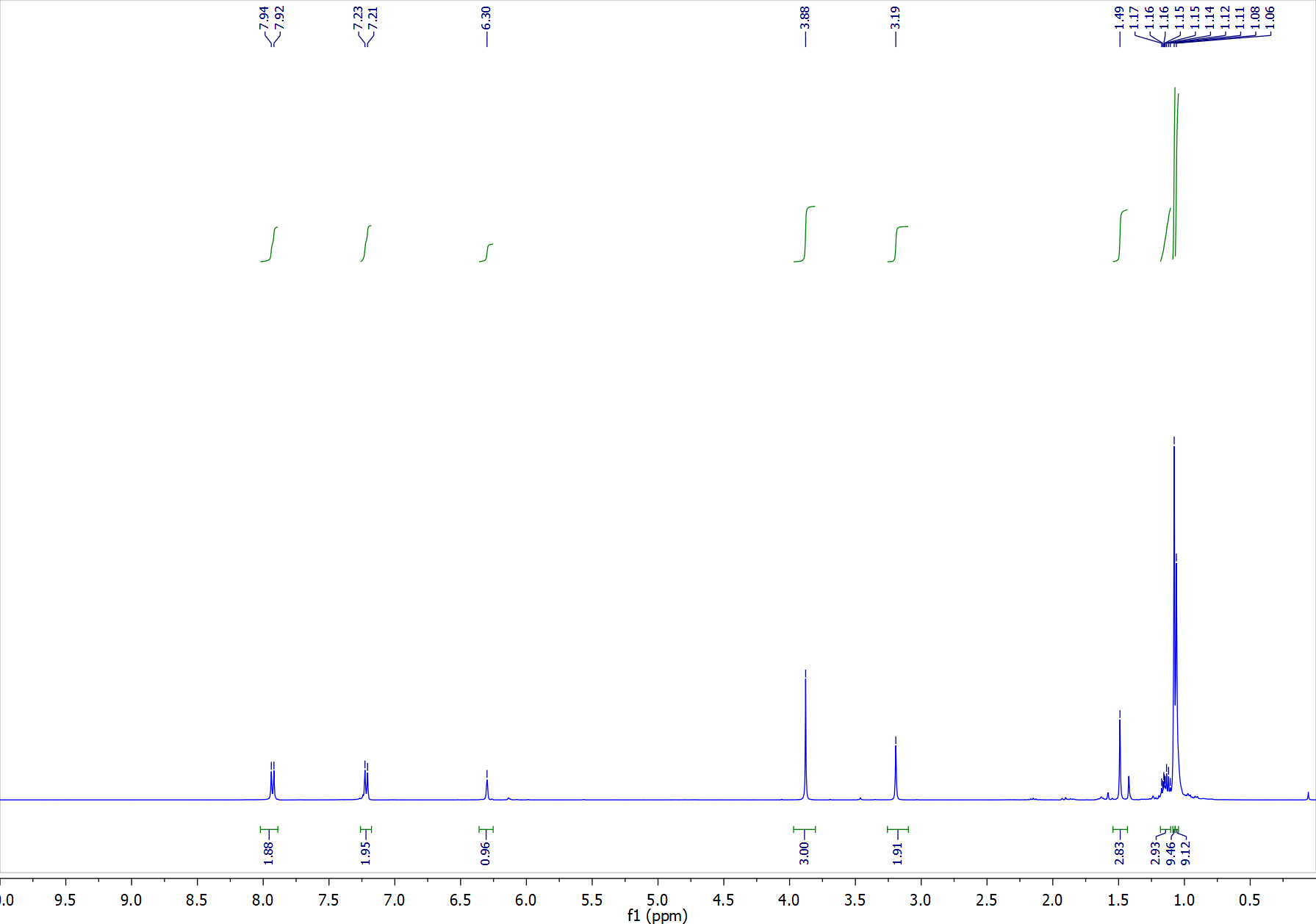
**Supplementary Figure 106.** ^1^H NMR spectrum of **34**
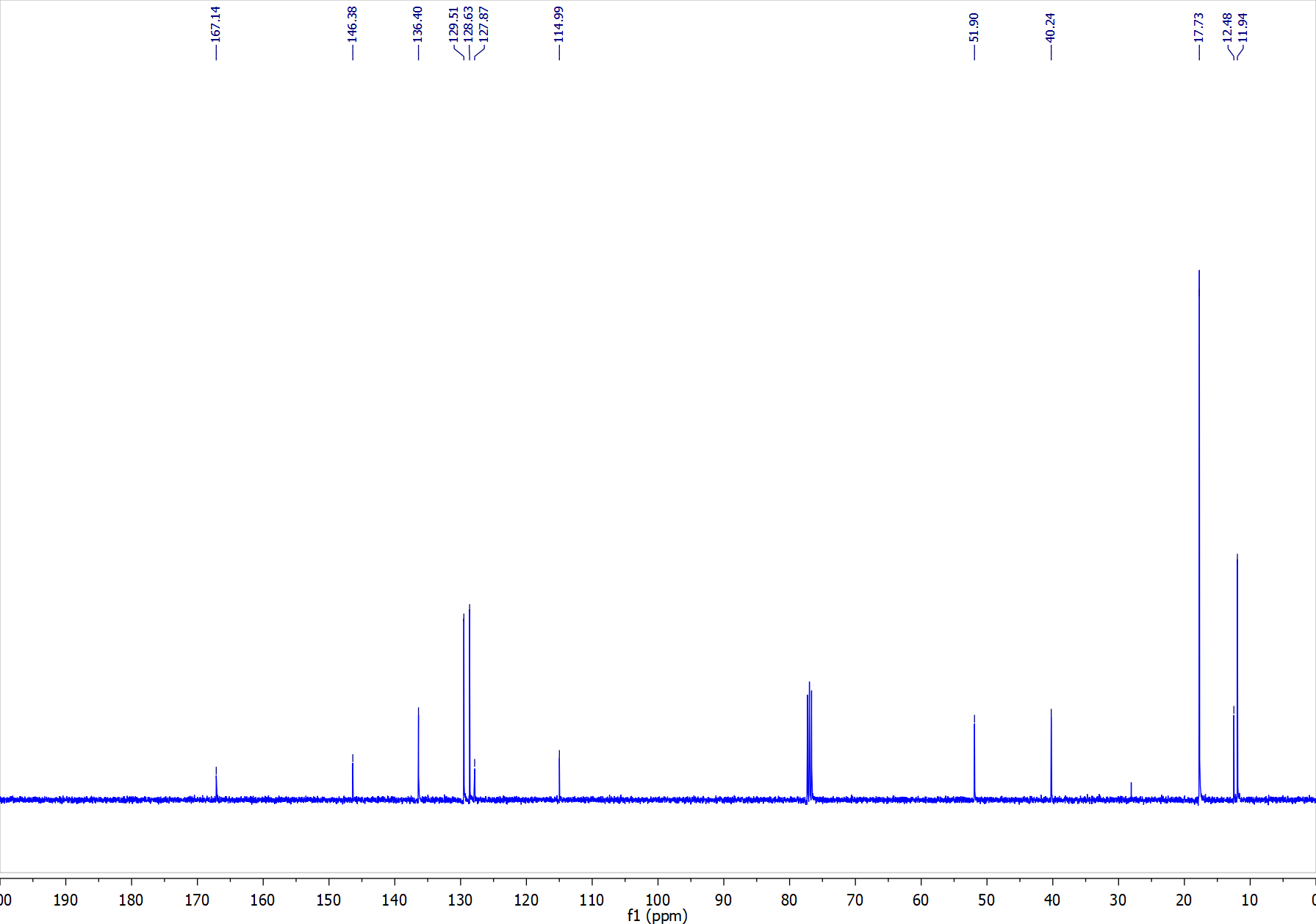
 **Supplementary Figure 107.** ^13^C NMR spectrum of **34**


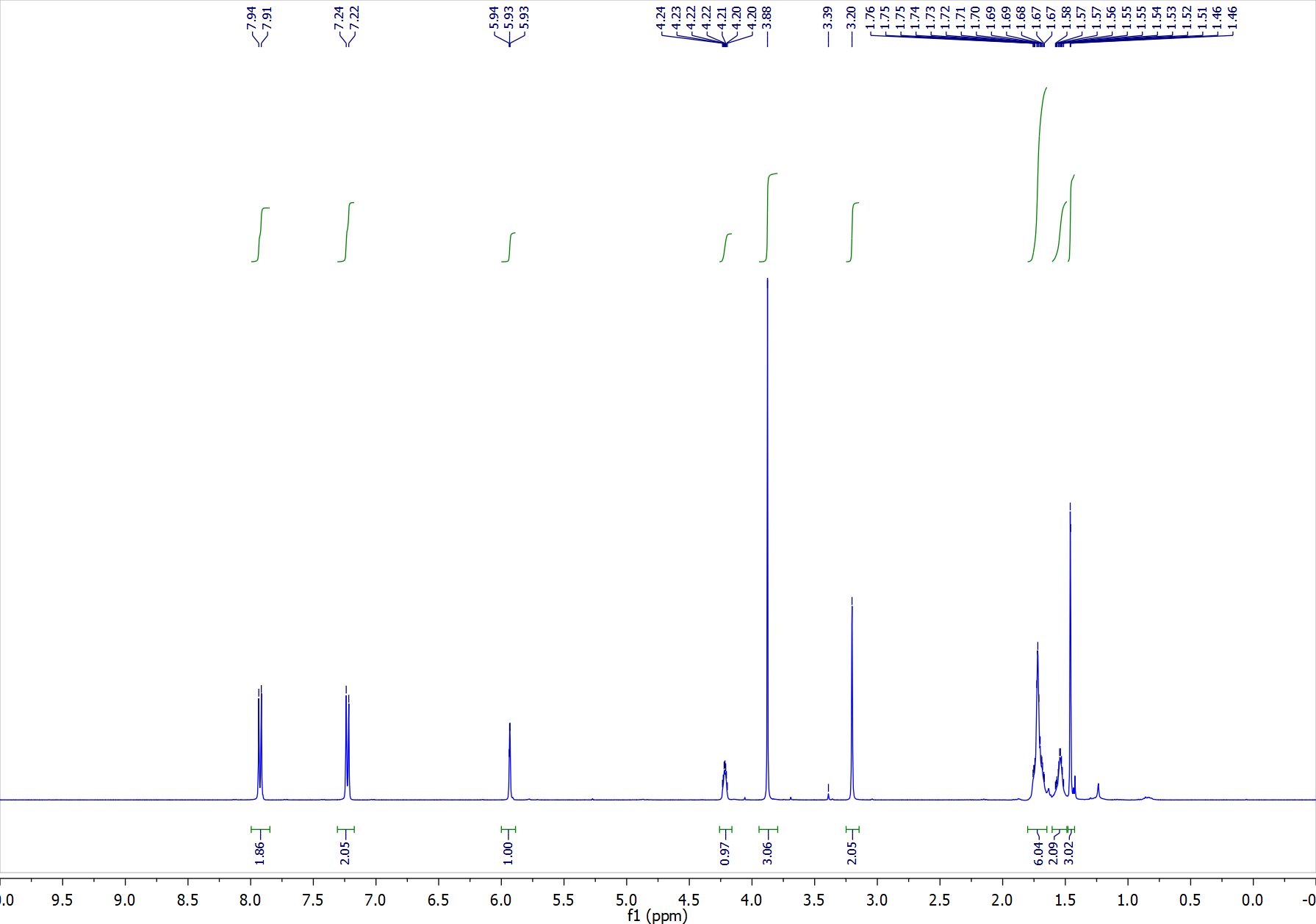
**Supplementary Figure 108.** ^1^H NMR spectrum of **35**


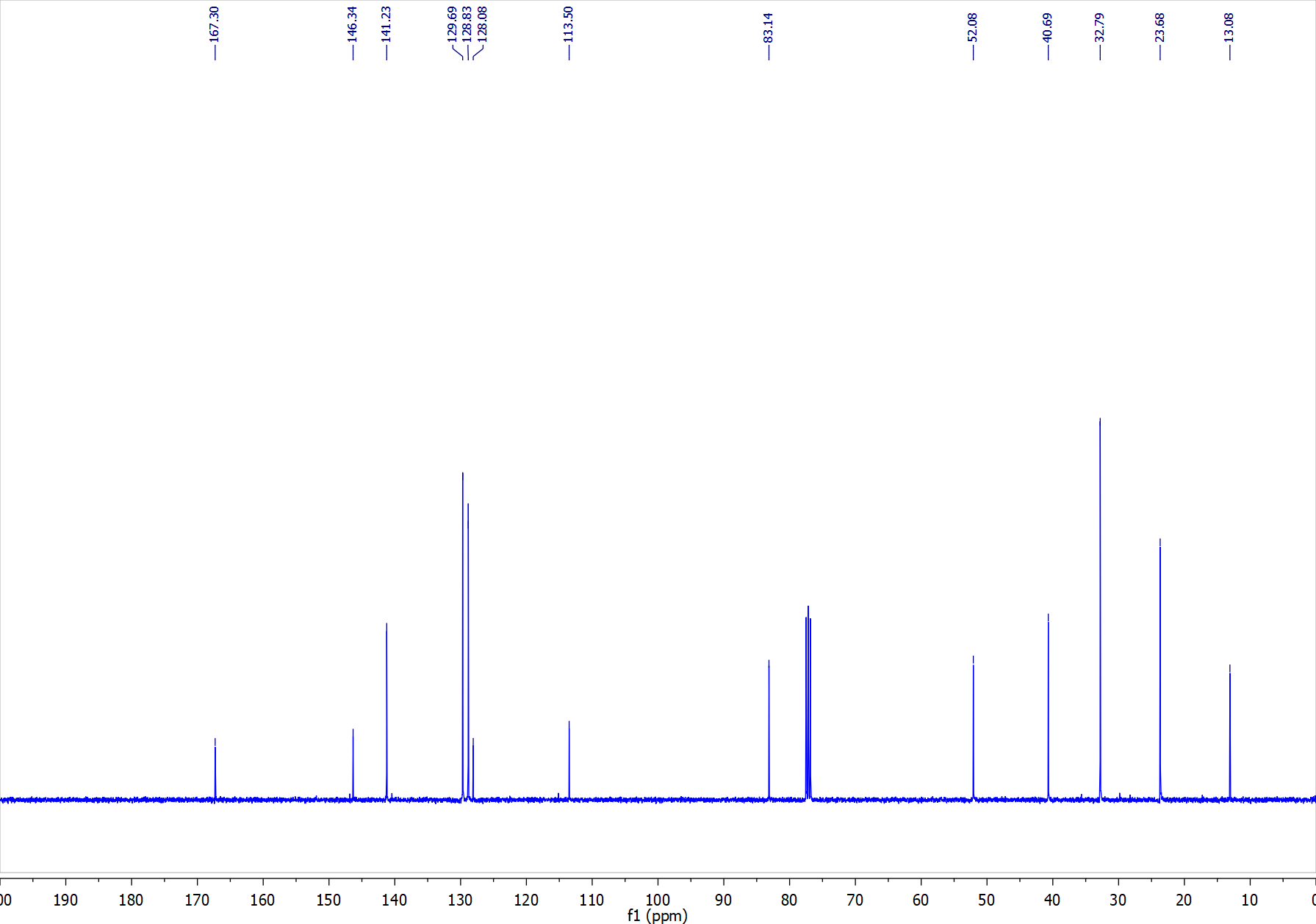
**Supplementary Figure 109.** ^13^C NMR spectrum of **35**


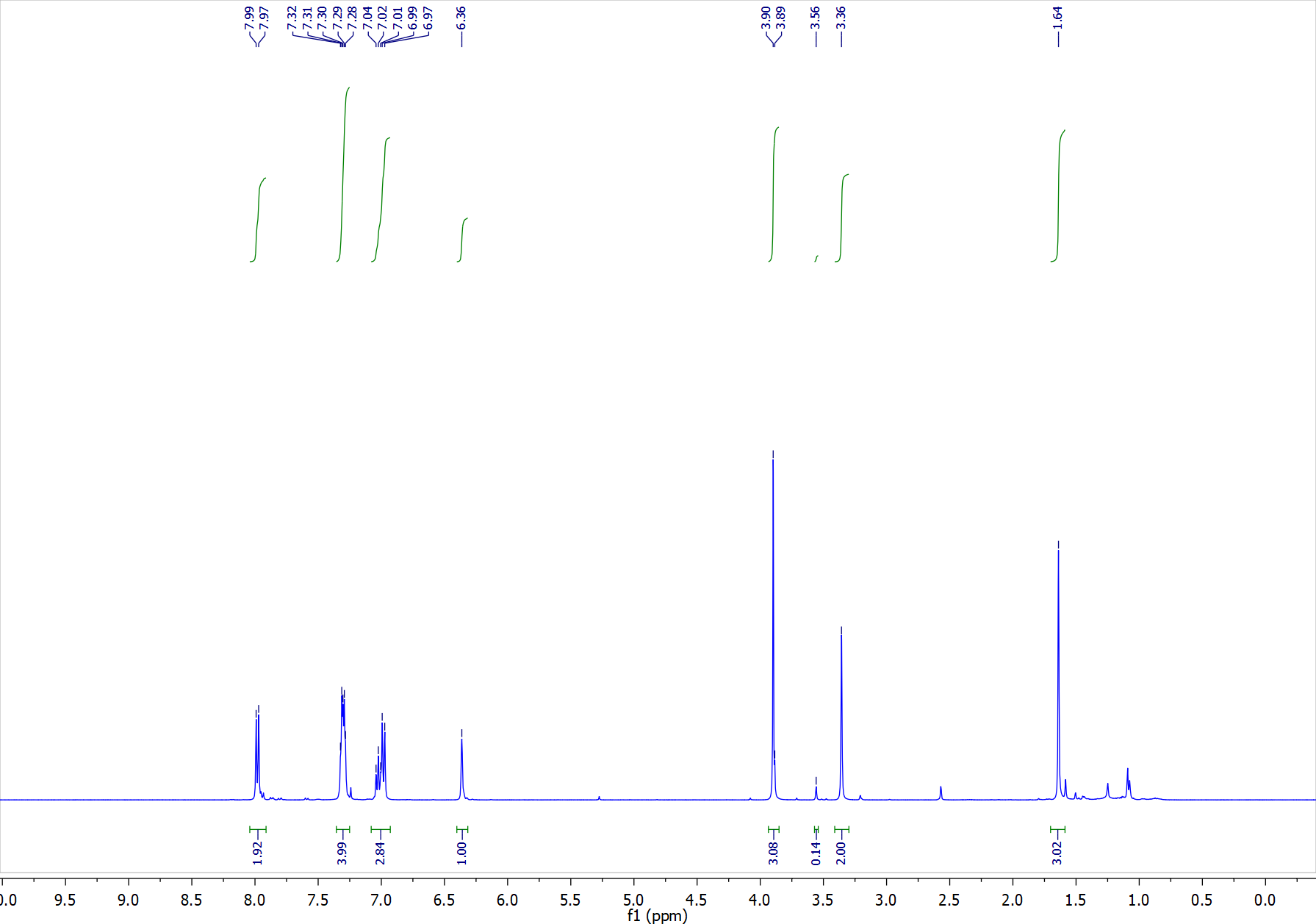
 **Supplementary Figure 110.** ^1^H NMR spectrum of **36**


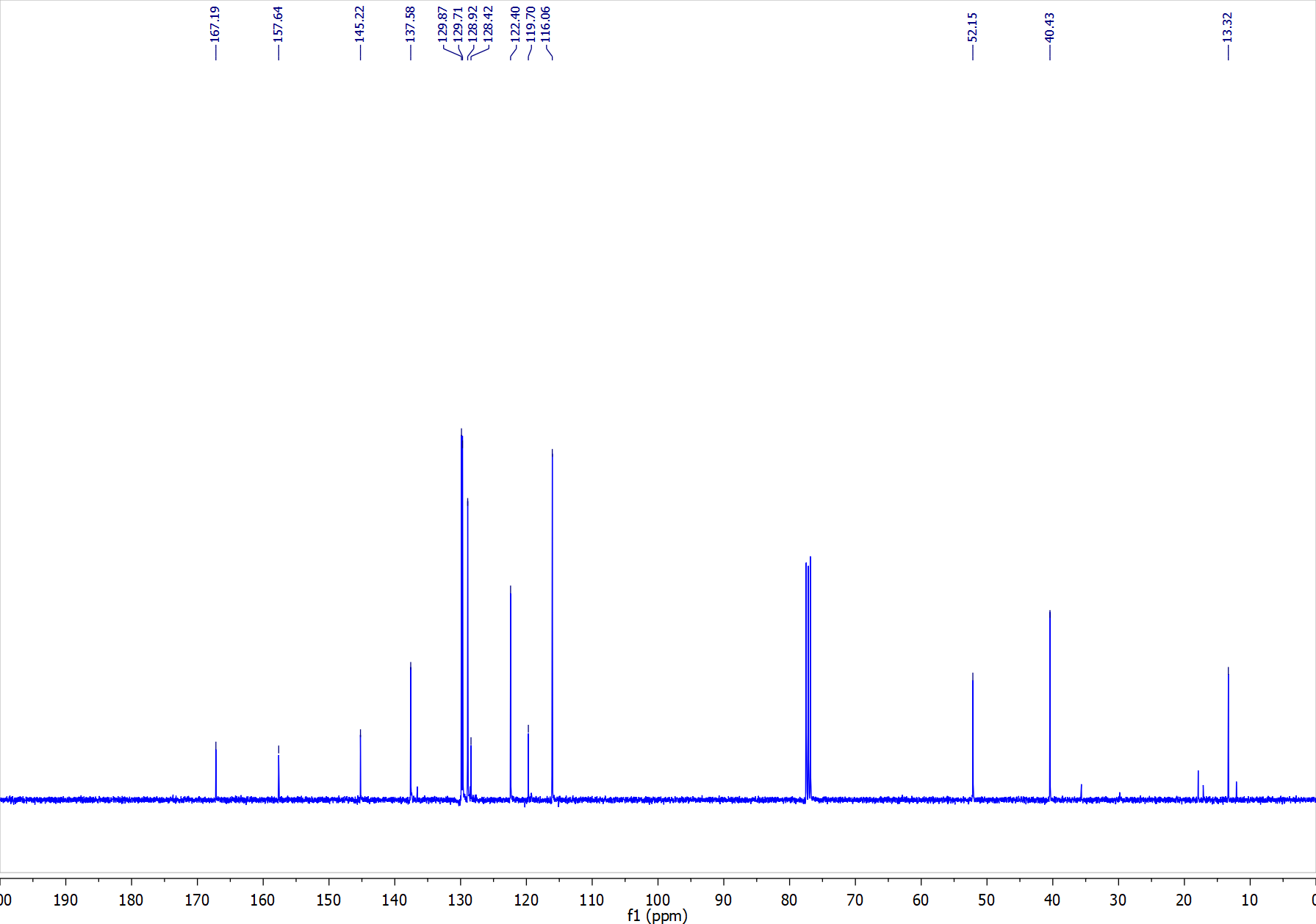
 **Supplementary Figure 111.** ^13^C NMR spectrum of **36**


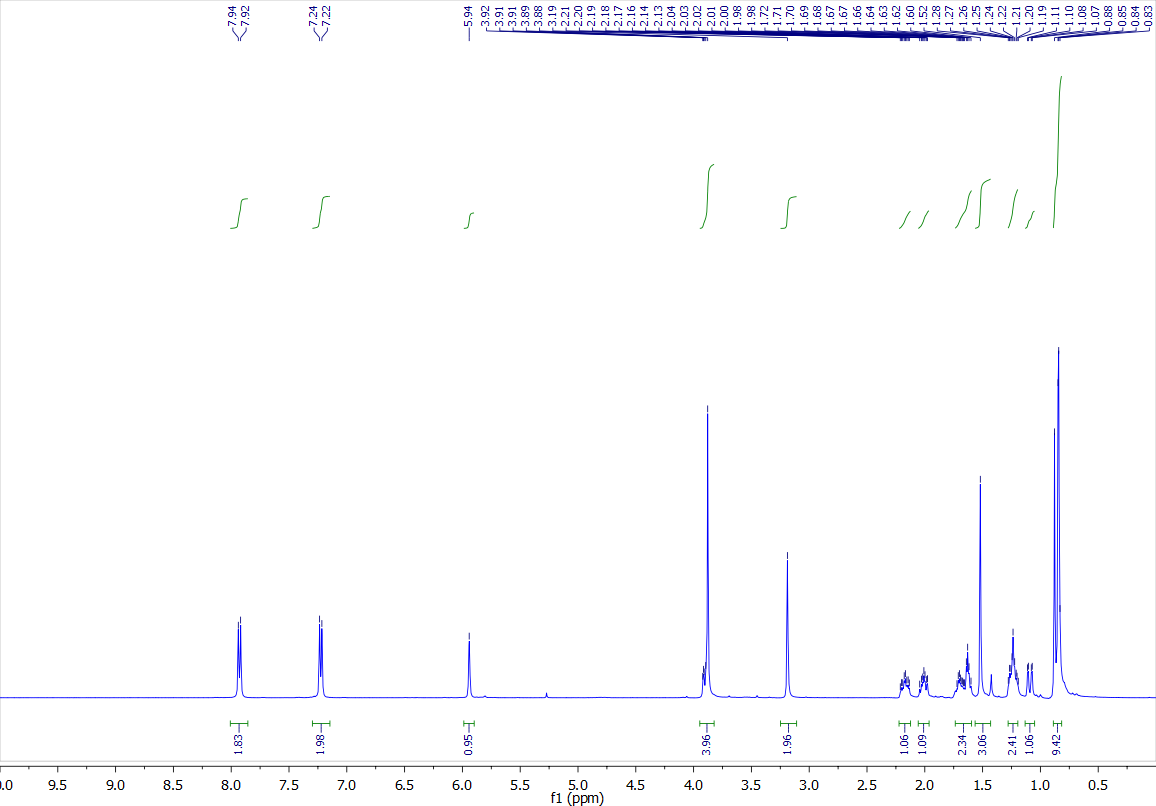
**Supplementary Figure 112.** ^1^H NMR spectrum of **37**


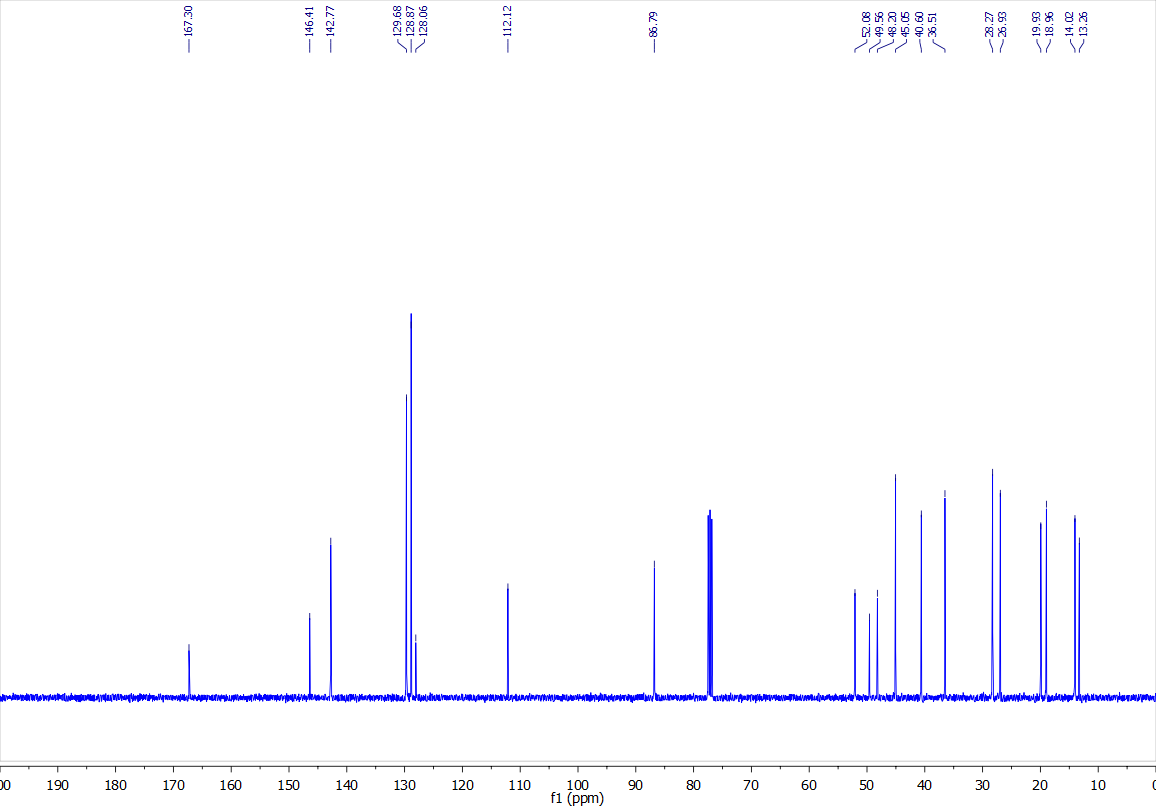
**Supplementary Figure 113.** ^13^C NMR spectrum of **37**


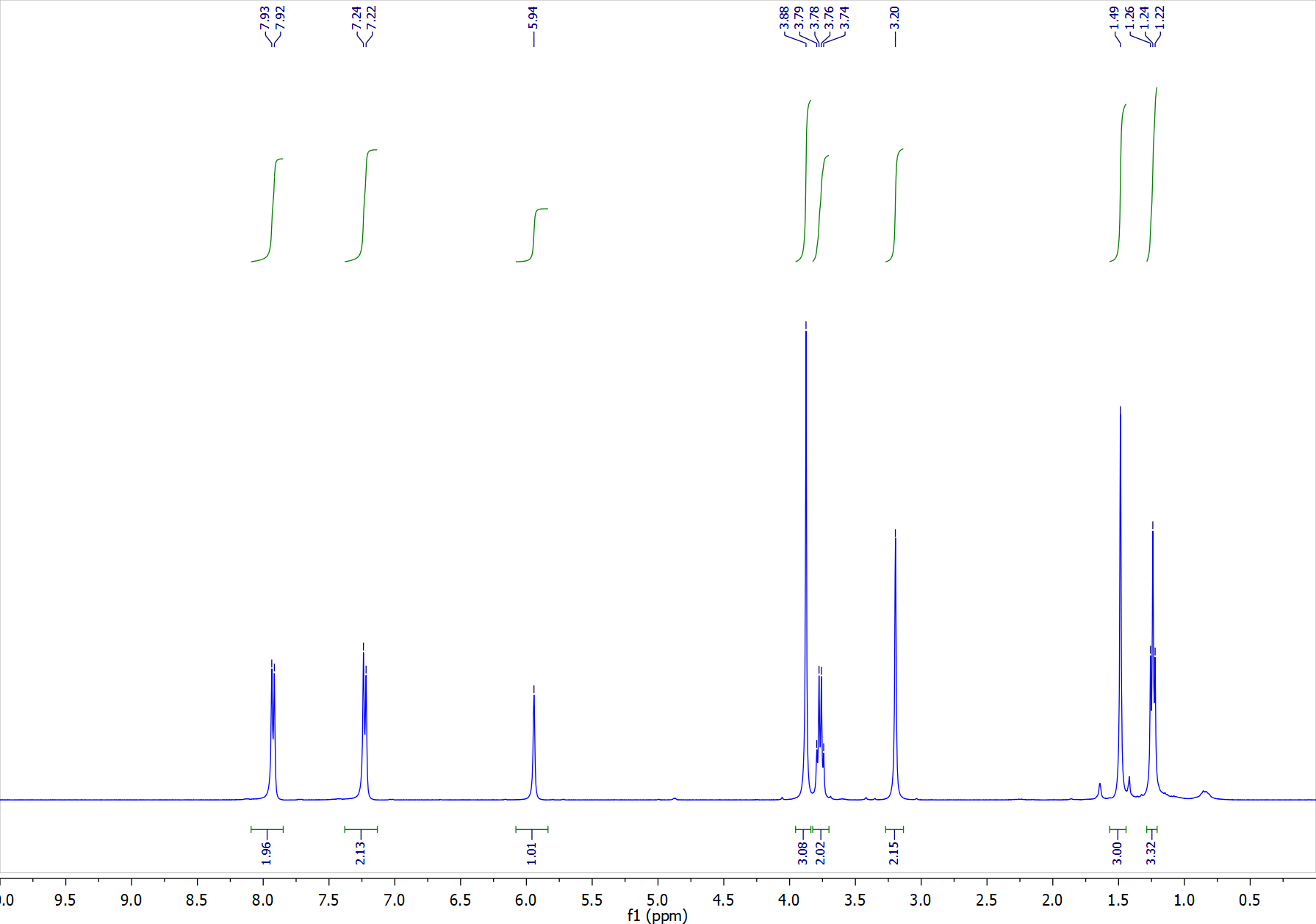
 **Supplementary Figure 114.** ^1^H NMR spectrum of **38**


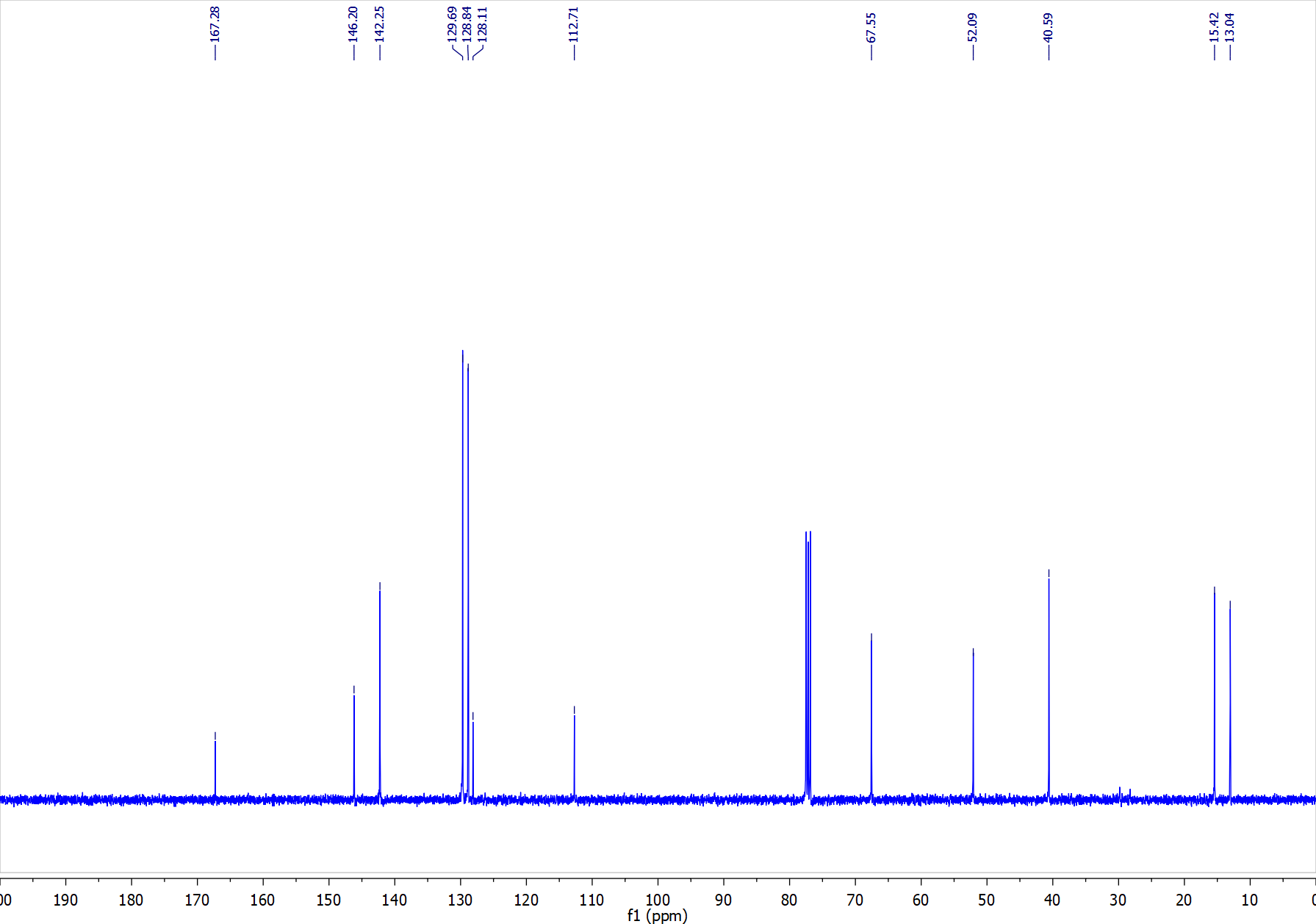
**Supplementary Figure 115.** ^13^C NMR spectrum of **38**

**Supplementary Figure 116.** ^1^H NMR spectrum of **39**

**Supplementary Figure 117.** ^13^C NMR spectrum of **39**

**Supplementary Figure 118.** ^1^H NMR spectrum of **40**

**Supplementary Figure 119.** ^13^C NMR spectrum of **40**

**Supplementary Figure 120.** ^1^H NMR spectrum of **41**

**Supplementary Figure 121.** ^13^C NMR spectrum of **41**

**Supplementary Figure 122.** ^1^H NMR spectrum of **42**

**Supplementary Figure 123.** ^13^C NMR spectrum of **42**

**Supplementary Figure 124.** ^1^H NMR spectrum of **43**

**Supplementary Figure 125.** ^13^C NMR spectrum of **43**

**Supplementary Figure 126.** ^1^H NMR spectrum of **44**

**Supplementary Figure 127.** ^13^C NMR spectrum of **44**

**Supplementary Figure 128.** ^1^H NMR spectrum of **45**

**Supplementary Figure 129.** ^13^C NMR spectrum of **45**

**Supplementary Figure 130.** ^1^H-^1^H NOESY spectra of **45**

**Supplementary Figure 131.** ^1^H NMR spectrum of **46**

**Supplementary Figure 132.** ^13^C NMR spectrum of **46**

**Supplementary Figure 133.** ^1^H NMR spectrum of **47**

**Supplementary Figure 134.** ^13^C NMR spectrum of **47**

**Supplementary Figure 135.** ^1^H NMR spectrum of **48**

**Supplementary Figure 136.** ^13^C NMR spectrum of **48**

**Supplementary Figure 137.** ^1^H NMR spectrum of **4****9** **Supplementary Figure 138.** ^13^C NMR spectrum of **49**

**Supplementary Figure 139.** ^1^H NMR spectrum of **50** **Supplementary Figure 140.** ^13^C NMR spectrum of **50**

**Supplementary Figure 141.** ^1^H NMR spectrum of **51** **Supplementary Figure 142.** ^13^C NMR spectrum of **51**

**Supplementary Figure 142.** ^1^H NMR spectrum of **52** **Supplementary Figure 143.** ^13^C NMR spectrum of **52**

**Supplementary Figure 144.** ^1^H NMR spectrum of **53** **Supplementary Figure 145.** ^13^C NMR spectrum of **53** **Supplementary Figure 146.** ^19^F NMR spectrum of **5****3**

**Supplementary Figure 147.** ^1^H NMR spectrum of **54** **Supplementary Figure 148.** ^13^C NMR spectrum of **54**

**Supplementary Figure 149.** ^1^H NMR spectrum of **55** **Supplementary Figure 150.** ^13^C NMR spectrum of **55**

**Supplementary Figure 151.** ^1^H NMR spectrum of **56** **Supplementary Figure 152.** ^13^C NMR spectrum of **56**

**Supplementary Figure 153.** ^1^H NMR spectrum of **57**

**Supplementary Figure 154.** ^13^C NMR spectrum of **57**

**Supplementary Figure 155.** ^1^H NMR spectrum of **58** **Supplementary Figure 156.** ^13^C NMR spectrum of **58**

**Supplementary Figure 157.** ^1^H NMR spectrum of **59** **Supplementary Figure 158.** ^13^C NMR spectrum of **59**

**Supplementary Figure 159.** ^1^H NMR spectrum of **60** **Supplementary Figure 160.** ^13^C NMR spectrum of **60**

**Supplementary Figure 161.** ^1^H NMR spectrum of **61** **Supplementary Figure 162.** ^13^C NMR spectrum of **61**

**Supplementary Figure 163.** ^1^H NMR spectrum of **62** **Supplementary Figure 164.** ^13^C NMR spectrum of **62**

**Supplementary Figure 165.** ^1^H NMR spectrum of **63** **Supplementary Figure 166.** ^13^C NMR spectrum of **6****3** **Supplementary Figure 167.** ^13^C NMR spectrum of **6****3**

**Supplementary Figure 168.** ^1^H NMR spectrum of **64**

**Supplementary Figure 169.** ^13^C NMR spectrum of **64**

**Supplementary Figure 170.** ^1^H NMR spectrum of **65**

**Supplementary Figure 171.** ^13^C NMR spectrum of **65**

**Supplementary Figure 172.** ^1^H NMR spectrum of **70**

**Supplementary Figure 173.** ^13^C NMR spectrum of **70**

**Supplementary Figure 174.** ^1^H NMR spectrum of **72** **Supplementary Figure 175.** ^13^C NMR spectrum of **72**

**Supplementary Figure 176.** ^1^H NMR spectrum of **75** **Supplementary Figure 177.** ^13^C NMR spectrum of **75**

**Supplementary Figure 178.** ^1^H NMR spectrum of **76** **Supplementary Figure 179.** ^13^C NMR spectrum of **76**

**Supplementary Figure 180.** ^1^H-^1^H NOESY spectra of **76**

**Supplementary Figure 181.** ^1^H NMR spectrum of **77**

**Supplementary Figure 182.** ^13^C NMR spectrum of **77**

**Supplementary Figure 183.** ^1^H NMR spectrum of **78**

**Supplementary Figure 184.** ^13^C NMR spectrum of **78**

**Supplementary Figure 185.** ^1^H NMR spectrum of **80**

**Supplementary Figure 186.** ^13^C NMR spectrum of **80**

**Supplementary Figure 187.** ^1^H NMR spectrum of **81**

**Supplementary Figure 188.** ^13^C NMR spectrum of **81**

**Supplementary Figure 189.** ^1^H-^1^H NOESY spectrum of **81**

**Supplementary Figure 190.** ^1^H NMR spectrum of **82**

**Supplementary Figure 191.** ^13^C NMR spectrum of **8****2** **Supplementary Figure 192.** ^1^H-^1^H NOESY spectrum of **82**

**Supplementary Reference**

1 Su, C. & Williard, P. G. Isomerization of Allyl Ethers Initiated by Lithium Diisopropylamide. *Org. Lett.* **12**, 5378-5381 (2010).

2 Li, J., Qu, S. & Zhao, W. Rhodium-Catalyzed Remote C(sp3)−H Borylation of Silyl Enol Ethers. *Angew. Chem. Int. Ed.* **59**, 2360-2364 (2020).

3 Larsen, C. R. & Grotjahn, D. B. Stereoselective Alkene Isomerization over One Position. *J. Am. Chem. Soc.* **134**, 10357-10360 (2012).

4 Brannock, K. Notes- Reactions of Ethyl Isobutenyl Ether. *J. Org. Chem.* **25**, 258-260 (1960).

5 Nordmann, G. & Buchwald, S. L. A Domino Copper-Catalyzed C−O Coupling−Claisen Rearrangement Process. *J. Am. Chem. Soc.* **125**, 4978-4979 (2003).

6 Woźniak, Ł., Magagnano, G. & Melchiorre, P. Enantioselective Photochemical Organocascade Catalysis. *Angew. Chem. Int. Ed.* **57**, 1068-1072 (2018).

7 Chang, C.-Y., Lin, Y.-H. & Wu, Y.-K. Palladium-catalyzed N1-selective allylation of indoles with allylic alcohols promoted by titanium tetraisopropoxide. *Chem. Commun.* **55**, 1116-1119 (2019).

8 Barbier, P. & Benezra, C. Allergenic .alpha.-methylene-.gamma.-butyrolactones. .beta.-Hydroxy-.alpha.-methylene-.gamma.-butyrolactones. 2. Syntheses from ethyl 2-(phenylthio)propionate and .alpha.-acetoxy aldehydes. *J. Org. Chem.* **48**, 2705-2709 (1983).

9 Johnson, J. R., Tully, P. S., Mackenzie, P. B. & Sabat, M. A practical reversed-polarity alternative to organocuprate conjugate addition chemistry. Halocarbon coupling reactions of enal- and enone-derived allylnickel reagents. *J. Am. Chem. Soc.* **113**, 6172-6177 (1991).

10 Huang, L. & Rueping, M. Direct Cross-Coupling of Allylic C(sp3)−H Bonds with Aryl- and Vinylbromides by Combined Nickel and Visible-Light Catalysis. *Angew. Chem. Int. Ed.* **57**, 10333-10337 (2018).

11 Han, X., Zhang, Y. & Wu, J. Mild Two-Step Process for the Transition-Metal-Free Synthesis of Carbon−Carbon Bonds from Allylic Alcohols/Ethers and Grignard Reagents. *J. Am. Chem. Soc.* **132**, 4104-4106 (2010).

12 Papoian, V. & Minehan, T. Palladium-Catalyzed Reactions of Arylindium Reagents Prepared Directly from Aryl Iodides and Indium Metal. *J. Org. Chem.* **73**, 7376-7379 (2008).

13 Li, M.-B., Wang, Y. & Tian, S.-K. Regioselective and Stereospecific Cross-Coupling of Primary Allylic Amines with Boronic Acids and Boronates through Palladium-Catalyzed C-N Bond Cleavage. *Angew. Chem. Int. Ed.* **51**, 2968-2971 (2012).

14 Shen, Z.-L. *et al.* Palladium-Catalyzed Cross-Coupling of Indium Homoenolate with Aryl Halide with Wide Functional Group Compatibility. *Org. Lett.* **13**, 422-425 (2011).
