# Supplementary material for: Modulating stereoselectivity in allylic C(sp3)-H bond arylations via nickel and photoredox catalysis
Source: Nat Commun. 2023 Feb 1;14:548. doi: 10.1038/s41467-023-36103-0 (PMC9892578; doi:10.1038/s41467-023-36103-0)
Supplement: Supplementary file 2 — Description of Additional Supplementary Files [file 41467_2023_36103_MOESM2_ESM.docx]

**Description of Additional Supplementary Files**

**File Name: Supplementary Data 1
Description:** Cartesian coordinates (Å) of the optimized structures of all intermediates and transition states at PBE/def2-SVP/def2-def2TZVP level of theory. EeS represents the absolute electronic energy in Hartree at M06(SMD)/def2-TZVPP level of theory in THF solvent.
