# Supplementary material for: Modulating stereoselectivity in allylic C(sp3)-H bond arylations via nickel and photoredox catalysis
Source: Nat Commun. 2023 Feb 1;14:548. doi: 10.1038/s41467-023-36103-0 (PMC9892578; doi:10.1038/s41467-023-36103-0)
Supplement: Supplementary file 3 — Supplementary Data 1 [file 41467_2023_36103_MOESM3_ESM.docx]

Cartesian coordinates (Å) of the optimized structures of all intermediates and transition states at PBE/def2-SVP/def2-def2TZVP level of theory. *E*_e_^S^ represents the absolute electronic energy in Hartree at M06(SMD)/def2-TZVPP level of theory in THF solvent.

**^2^A1**

46

charge = 0; spin = 2

*E*_e_^S^: -4892.05358430

Zero-point correction=0.333414

Thermal correction to Energy=0.359889

Thermal correction to Enthalpy=0.360833

Thermal correction to Gibbs Free Energy=0.272405

C -1.154696 -0.125519 0.001490

C 0.734598 1.243986 0.000721

C -0.073723 2.389769 -0.001442

C -1.480448 2.291198 -0.002217

C -2.008890 0.980752 -0.000691

H -1.549728 -1.153714 0.002771

H 0.407643 3.378308 -0.002525

H -3.091813 0.800776 -0.001174

C 2.209829 1.242286 0.001637

C 3.020856 2.386164 0.000697

C 4.427343 2.284215 0.001865

H 2.541856 3.375852 -0.000971

C 4.095860 -0.131722 0.004740

C 4.952687 0.972529 0.003968

H 4.488341 -1.160896 0.006292

H 6.035183 0.790002 0.005037

Ni 1.468930 -1.470762 0.004799

Br 1.471285 -3.725644 0.007575

N 0.191468 -0.014900 0.002214

N 2.750007 -0.017818 0.003621

C -2.353330 3.557322 -0.004502

C 5.303227 3.548268 0.000778

C -2.035508 4.390510 -1.269858

H -2.653179 5.312086 -1.287121

H -0.972011 4.701057 -1.307237

H -2.252257 3.815080 -2.192556

C -3.856105 3.220552 -0.005122

H -4.151999 2.642681 0.893778

H -4.449626 4.156748 -0.006787

H -4.150720 2.640288 -0.902900

C -2.037265 4.393770 1.259143

H -2.654957 5.315389 1.273180

H -2.255291 3.820720 2.183019

H -0.973816 4.704403 1.297189

C 4.989420 4.381862 -1.265313

H 3.926710 4.694874 -1.304490

H 5.609281 5.301982 -1.281825

H 5.206307 3.805672 -2.187503

C 4.987101 4.385784 1.263694

H 5.606973 5.305924 1.278495

H 3.924344 4.698984 1.299948

H 5.202265 3.812450 2.188065

C 6.805204 3.207954 0.002702

H 7.098232 2.629478 0.902152

H 7.099965 2.626912 -0.894524

H 7.400926 4.142753 0.001931

**R_E_**

33

charge = 0; spin = 2

*E*_e_^S^: -758.275609029

Zero-point correction=0.247009

Thermal correction to Energy=0.267109

Thermal correction to Enthalpy=0.268053

Thermal correction to Gibbs Free Energy=0.199821

Si -2.936795 0.941923 0.000678

C -3.215922 -0.953334 0.002406

C -2.565876 -1.561894 1.262264

H -2.692708 -2.667550 1.265949

H -1.478719 -1.347131 1.304979

H -3.024428 -1.176871 2.196899

C -4.731113 -1.244660 0.002927

H -5.238914 -0.832046 -0.894164

H -4.912779 -2.342552 0.003854

H -5.238597 -0.830592 0.899529

C -2.566334 -1.564097 -1.256622

H -2.693123 -2.669762 -1.258303

H -3.025272 -1.180743 -2.191755

H -1.479207 -1.349348 -1.300146

C -3.604821 1.791282 1.549341

H -3.180558 1.346672 2.471913

H -3.348015 2.870605 1.545891

H -4.710136 1.712980 1.605011

C -3.605228 1.788502 -1.549329

H -3.348502 2.867849 -1.547791

H -3.181067 1.342326 -2.471192

H -4.710545 1.709997 -1.604689

O -1.218504 1.088386 0.000292

C -0.512551 2.246120 -0.000481

H -1.075211 3.198743 -0.000605

C 0.894334 2.235099 -0.001021

C 1.620348 3.424307 -0.001728

H 2.719791 3.418371 -0.002136

H 1.114633 4.402637 -0.001899

C 1.592340 0.892110 -0.000741

H 1.306644 0.291229 0.887680

H 1.305993 0.290520 -0.888472

H 2.692393 1.012247 -0.001190

**^1^A2_E_**

79

charge = 0; spin = 1

*E*_e_^S^: -5650.36067898

Zero-point correction=0.583463

Thermal correction to Energy=0.630648

Thermal correction to Enthalpy=0.631592

Thermal correction to Gibbs Free Energy=0.499204

C -0.792764 -1.195670 0.475610

C 0.205299 0.696875 1.390888

C 0.392003 1.238309 0.106348

C -0.042002 0.552421 -1.043554

C -0.651130 -0.701809 -0.825202

H -1.230124 -2.187518 0.665622

H 0.904280 2.205458 0.007104

H -1.012375 -1.317615 -1.658821

C 0.650756 1.346953 2.642061

C 1.149447 2.659718 2.707663

C 1.579881 3.216105 3.927635

H 1.193798 3.259566 1.787405

C 0.947548 1.086958 4.924314

C 1.468984 2.377867 5.058587

H 0.857040 0.430487 5.805295

H 1.787444 2.713830 6.054352

Ni -0.489843 -1.200966 3.419962

C -2.414032 -0.744998 3.748579

H -3.087699 -0.683595 2.877342

H -2.410004 0.146047 4.401430

C -2.074346 -2.027660 4.276739

C -1.039817 -1.932930 5.272132

H -0.988675 -1.010783 5.892299

C -2.630700 -3.346125 3.813463

H -3.101622 -3.251519 2.815188

H -1.834594 -4.113217 3.762287

H -3.408745 -3.708122 4.521846

O -0.561388 -3.061845 5.843052

Si 0.618329 -3.237190 7.087560

C 0.782163 -5.138312 7.269018

C 1.639036 -5.456190 8.512273

H 1.171687 -5.091366 9.451204

H 1.769920 -6.556483 8.617869

H 2.656207 -5.014942 8.446294

C 1.460467 -5.708934 6.005966

H 0.909571 -5.441959 5.081294

H 2.496969 -5.331733 5.885972

H 1.516489 -6.819106 6.069063

C -0.618846 -5.762114 7.431686

H -1.141424 -5.390489 8.338496

H -1.263898 -5.545198 6.556856

H -0.538072 -6.868031 7.529399

C 2.230467 -2.403627 6.583144

H 2.440258 -2.616307 5.513855

H 2.178965 -1.302804 6.708653

H 3.076717 -2.769219 7.201082

C -0.089069 -2.426373 8.647033

H -0.310854 -1.353296 8.468879

H -1.028423 -2.914270 8.977091

H 0.637711 -2.476552 9.484127

N -0.394261 -0.513442 1.564783

N 0.539979 0.562613 3.753999

C 2.127805 4.652404 3.985079

C 0.171929 1.157529 -2.441939

C 2.547226 5.051744 5.412085

H 3.349987 4.396419 5.806585

H 2.936961 6.089546 5.411798

H 1.693984 5.018575 6.119558

C 1.032167 5.633130 3.501916

H 0.714879 5.421281 2.461129

H 0.132220 5.576909 4.147359

H 1.410310 6.675981 3.530531

C 3.364483 4.761559 3.060778

H 4.166994 4.067689 3.383123

H 3.117108 4.524369 2.006628

H 3.772072 5.793346 3.084558

C -0.389946 0.251813 -3.553524

H 0.107172 -0.739010 -3.571642

H -1.481503 0.090004 -3.444016

H -0.222145 0.724339 -4.542184

C -0.541624 2.528714 -2.517323

H -0.151368 3.242429 -1.764282

H -0.392408 2.984219 -3.518028

H -1.632463 2.422079 -2.348634

C 1.689161 1.348034 -2.682308

H 2.145804 2.029156 -1.936522

H 2.226928 0.380043 -2.628180

H 1.866002 1.784491 -3.687051

Br 1.130877 -2.929689 2.920533

**^3^A2_E_**

79

charge = 0; spin = 3

*E*_e_^S^: -5650.34630835

Zero-point correction=0.582049

Thermal correction to Energy=0.630015

Thermal correction to Enthalpy=0.630959

Thermal correction to Gibbs Free Energy=0.493285

C -0.185445 -1.274207 -0.057612

C 0.435925 0.715732 0.999896

C 0.328241 1.412625 -0.219425

C -0.034892 0.752465 -1.405737

C -0.284642 -0.638597 -1.296787

H -0.364105 -2.355816 0.053701

H 0.535394 2.492359 -0.233655

H -0.550865 -1.243234 -2.173259

C 0.839938 1.305366 2.279532

C 1.257165 2.638831 2.452577

C 1.660096 3.127544 3.706601

H 1.277296 3.300077 1.574525

C 1.181761 0.892976 4.551606

C 1.616557 2.199588 4.777057

H 1.147561 0.162199 5.374918

H 1.923047 2.480041 5.792961

Ni 0.082740 -1.368293 2.901325

C -1.822641 -1.029644 3.504797

H -2.532641 -1.143740 2.668478

H -1.846094 -0.035591 3.984018

C -1.594749 -2.187778 4.335138

C -0.957915 -2.068862 5.572293

H -0.677704 -1.075976 5.974562

C -2.061643 -3.558005 3.896463

H -2.410463 -3.543138 2.846658

H -1.248673 -4.304956 3.978343

H -2.901801 -3.896951 4.539541

O -0.696640 -3.151213 6.328646

Si 0.549745 -3.322560 7.517160

C 0.523850 -5.196000 7.911357

C 1.491383 -5.480800 9.079164

H 1.195392 -4.952683 10.009999

H 1.504194 -6.569222 9.310809

H 2.535994 -5.188852 8.840717

C 0.970170 -5.979606 6.659267

H 0.318452 -5.771176 5.786868

H 2.009533 -5.732844 6.359993

H 0.932876 -7.074104 6.857728

C -0.906920 -5.617022 8.304630

H -1.261227 -5.095640 9.218739

H -1.631257 -5.406480 7.492157

H -0.941924 -6.709243 8.515684

C 2.171804 -2.739721 6.754460

H 2.256302 -3.099148 5.706911

H 2.238770 -1.632267 6.739940

H 3.043798 -3.113985 7.329527

C 0.073414 -2.256040 9.004262

H -0.030383 -1.191373 8.708484

H -0.889417 -2.578543 9.449201

H 0.851279 -2.304576 9.794162

N 0.156233 -0.623336 1.071846

N 0.790907 0.435386 3.343889

C 2.125587 4.585090 3.868810

C -0.141437 1.527943 -2.731126

C 2.510468 4.907659 5.324832

H 3.349666 4.276679 5.681401

H 2.835801 5.964657 5.401367

H 1.656178 4.772658 6.018871

C 0.981294 5.535767 3.441830

H 0.684287 5.379484 2.385381

H 0.080800 5.385728 4.071334

H 1.300641 6.593396 3.546167

C 3.361768 4.826865 2.969108

H 4.198874 4.156980 3.251842

H 3.136135 4.650507 1.898285

H 3.711619 5.875237 3.069365

C -0.543126 0.613168 -3.903250

H 0.200610 -0.191043 -4.074879

H -1.532534 0.140175 -3.739435

H -0.610123 1.207481 -4.836719

C -1.210896 2.637395 -2.586043

H -0.956895 3.355843 -1.780927

H -1.300371 3.211922 -3.531330

H -2.205091 2.205739 -2.351707

C 1.228625 2.169784 -3.057600

H 1.554999 2.875711 -2.267704

H 2.016181 1.396754 -3.165414

H 1.171008 2.736826 -4.009793

Br 1.327478 -3.394371 2.684464

**^3^[A2_E_-A2_Z_]^‡^**

79

charge = 0; spin = 3

*E*_e_^S^: -5650.31494600

Zero-point correction=0.581489

Thermal correction to Energy=0.628673

Thermal correction to Enthalpy=0.629617

Thermal correction to Gibbs Free Energy=0.494197

C -0.918684 -0.968201 -0.066101

C 0.071447 0.903600 0.897982

C 0.324633 1.448951 -0.371502

C -0.066539 0.772531 -1.543322

C -0.705197 -0.473328 -1.355819

H -1.385919 -1.952004 0.097300

H 0.843969 2.415193 -0.443022

H -1.036186 -1.080621 -2.208236

C 0.455186 1.530453 2.176735

C 1.090798 2.777279 2.298800

C 1.433666 3.301403 3.560260

H 1.320187 3.346491 1.386931

C 0.451605 1.275920 4.492724

C 1.091668 2.505782 4.675432

H 0.154808 0.644148 5.347972

H 1.315194 2.828875 5.700542

Ni -0.735843 -0.972888 2.909839

C -2.490704 -0.543228 3.835470

H -3.317878 -0.678609 3.116275

H -2.400277 0.460325 4.280798

C -1.942362 -1.670089 4.489178

C -1.154518 -1.543748 5.748037

H -0.064757 -1.744396 5.726992

C -2.515603 -3.058091 4.226447

H -3.091455 -3.093262 3.279851

H -1.712251 -3.819097 4.171529

H -3.200642 -3.333371 5.057850

O -1.820830 -1.874584 6.899340

Si -1.067865 -2.237533 8.404462

C -2.552591 -2.484251 9.593813

C -2.029044 -2.817191 11.005913

H -1.400177 -2.002835 11.423591

H -2.878898 -2.966182 11.709144

H -1.427049 -3.750115 11.020495

C -3.430331 -3.642090 9.075783

H -3.804549 -3.443007 8.050926

H -2.880488 -4.605994 9.054036

H -4.314437 -3.782956 9.737410

C -3.387692 -1.188058 9.634875

H -2.808677 -0.330925 10.038021

H -3.750051 -0.907249 8.625049

H -4.277260 -1.320736 10.290804

C -0.016430 -3.791100 8.196688

H -0.633113 -4.660819 7.893326

H 0.751664 -3.637792 7.410861

H 0.511322 -4.051739 9.137253

C 0.024198 -0.773725 8.896342

H 0.841223 -0.636469 8.158174

H -0.557756 0.168929 8.940636

H 0.495577 -0.935711 9.887623

N -0.557310 -0.298179 1.045530

N 0.141084 0.784849 3.277452

C 2.137197 4.664534 3.675525

C 0.215495 1.379954 -2.928315

C 2.418178 5.045362 5.141013

H 3.081849 4.310661 5.640164

H 2.925173 6.030291 5.181302

H 1.485041 5.128703 5.734184

C 1.236451 5.755356 3.047516

H 1.031299 5.559388 1.975950

H 0.261636 5.820281 3.572016

H 1.730069 6.746609 3.117102

C 3.484501 4.603859 2.915840

H 4.150575 3.827693 3.343915

H 3.345477 4.373201 1.840605

H 4.006968 5.580369 2.983579

C -0.303444 0.482567 -4.067209

H 0.184117 -0.513202 -4.065351

H -1.400441 0.331486 -4.008779

H -0.085619 0.955779 -5.045718

C -0.482758 2.757395 -3.030870

H -0.120753 3.465669 -2.258749

H -0.285359 3.214833 -4.022327

H -1.580809 2.659269 -2.911205

C 1.743369 1.558950 -3.100446

H 2.171994 2.232642 -2.331554

H 2.270370 0.586201 -3.027978

H 1.968508 1.999065 -4.093856

Br 0.712154 -2.864666 2.745600

**^3^A2_Z_**

79

charge = 0; spin = 3

*E*_e_^S^: -5650.34738302

Zero-point correction=0.582554

Thermal correction to Energy=0.630343

Thermal correction to Enthalpy=0.631287

Thermal correction to Gibbs Free Energy=0.494089

C -0.307425 -1.087650 -0.126976

C 0.471621 0.909925 0.807190

C 0.434861 1.530445 -0.455120

C 0.010137 0.830917 -1.598644

C -0.366812 -0.520057 -1.401032

H -0.570289 -2.142418 0.051287

H 0.752116 2.579760 -0.538809

H -0.702400 -1.148928 -2.235631

C 0.889257 1.555428 2.057898

C 1.250228 2.908904 2.185721

C 1.627208 3.452901 3.426386

H 1.228780 3.546634 1.290375

C 1.211540 1.234620 4.350131

C 1.595869 2.564119 4.528955

H 1.146065 0.537750 5.199817

H 1.864338 2.896174 5.540423

Ni 0.218401 -1.101031 2.795359

C -1.578378 -0.819609 3.755163

H -2.300250 -0.711502 2.924120

H -1.439169 0.117461 4.323342

C -1.756508 -2.008070 4.568370

C -1.242324 -2.122642 5.842606

H -1.418548 -3.042472 6.430217

C -2.485757 -3.197172 3.991044

H -1.900957 -3.632524 3.151694

H -2.634000 -3.999021 4.741667

H -3.481711 -2.905674 3.595081

O -0.505695 -1.145816 6.438790

Si 0.309867 -1.301845 7.951104

C 1.882669 -2.390526 7.736571

C 2.683064 -2.341695 9.056433

H 2.102162 -2.735151 9.917417

H 3.600326 -2.966232 8.971072

H 3.012481 -1.312631 9.311833

C 2.744940 -1.840522 6.581758

H 2.215850 -1.894369 5.607818

H 3.057295 -0.788472 6.752327

H 3.675062 -2.442462 6.478552

C 1.491761 -3.849925 7.423185

H 0.862789 -4.299136 8.220200

H 0.949219 -3.937961 6.459439

H 2.406202 -4.478120 7.335761

C 0.728999 0.481759 8.398329

H 1.488140 0.917244 7.717652

H -0.183345 1.109364 8.339158

H 1.124558 0.550175 9.432084

C -0.895877 -2.020179 9.216812

H -1.795568 -1.376046 9.295099

H -1.232290 -3.042975 8.953429

H -0.426974 -2.071740 10.221001

N 0.093105 -0.397793 0.960528

N 0.881564 0.718143 3.146542

C 2.031187 4.932901 3.543526

C -0.021086 1.523877 -2.972375

C 2.410034 5.313298 4.987076

H 3.271653 4.722401 5.358553

H 2.698649 6.382805 5.029597

H 1.563698 5.170537 5.689121

C 0.845563 5.821941 3.096730

H 0.551155 5.624328 2.046483

H -0.045025 5.650739 3.734779

H 1.119930 6.894672 3.170705

C 3.251333 5.200141 2.629370

H 4.118012 4.575753 2.926871

H 3.028373 4.982337 1.565612

H 3.556186 6.265074 2.695937

C -0.524693 0.581665 -4.081716

H 0.129656 -0.305417 -4.200942

H -1.556963 0.226710 -3.886917

H -0.535852 1.117739 -5.052006

C -0.964853 2.748688 -2.903188

H -0.631504 3.487967 -2.147422

H -0.996473 3.265330 -3.884761

H -1.998525 2.442752 -2.643295

C 1.408514 1.991878 -3.337162

H 1.812557 2.709346 -2.595020

H 2.108046 1.133355 -3.391927

H 1.406999 2.497469 -4.325003

Br 0.986307 -3.310580 2.558775

**^1^A2_Z_**

79

charge = 0; spin = 1

*E*_e_^S^: -5650.35655057

Zero-point correction=0.583987

Thermal correction to Energy=0.630952

Thermal correction to Enthalpy=0.631896

Thermal correction to Gibbs Free Energy=0.500598

C -1.139971 -1.146741 0.064278

C 0.006968 0.532759 1.193812

C 0.178987 1.243629 -0.006940

C -0.334654 0.758556 -1.223068

C -1.006601 -0.482294 -1.156742

H -1.633827 -2.127456 0.120327

H 0.738725 2.189197 0.011928

H -1.427298 -0.954788 -2.053807

C 0.561151 0.944874 2.491612

C 1.298945 2.123516 2.694660

C 1.849406 2.442042 3.948748

H 1.448570 2.802359 1.843565

C 0.881441 0.342047 4.714874

C 1.621679 1.496673 4.973253

H 0.725118 -0.403668 5.506513

H 2.021912 1.639660 5.985434

Ni -0.675531 -1.537425 2.997838

C -2.259214 -2.777860 2.757100

H -2.931479 -2.595902 1.901733

H -1.833817 -3.788329 2.846108

C -2.368930 -1.940916 3.904085

C -1.317553 -2.187178 4.862342

H -1.157255 -1.417271 5.647770

C -3.367677 -0.822225 4.063832

H -3.678310 -0.409399 3.083278

H -4.283416 -1.188883 4.579630

H -2.957768 0.008648 4.673383

O -0.956820 -3.451220 5.192164

Si 0.115261 -3.987212 6.436764

C 0.137462 -5.890299 6.216676

C 0.831355 -6.526971 7.439646

H 0.285048 -6.330062 8.386117

H 0.886028 -7.631813 7.316750

H 1.873669 -6.164978 7.567680

C 0.914056 -6.247528 4.932207

H 0.496086 -5.747763 4.034748

H 1.980469 -5.948545 4.996483

H 0.887060 -7.347679 4.763242

C -1.309816 -6.412776 6.112256

H -1.903875 -6.190098 7.023921

H -1.840614 -5.968731 5.246638

H -1.309867 -7.518225 5.982111

C 1.811501 -3.194613 6.233914

H 2.080128 -3.169373 5.155624

H 1.830344 -2.156941 6.626381

H 2.583968 -3.768954 6.786279

C -0.667470 -3.467877 8.082842

H -0.811372 -2.367842 8.124245

H -1.656690 -3.943794 8.238911

H -0.013974 -3.744535 8.935956

N -0.670375 -0.655073 1.229141

N 0.334806 0.054918 3.512879

C 2.654891 3.736625 4.149065

C -0.138037 1.548388 -2.528402

C 3.154674 3.883646 5.598308

H 3.830212 3.054155 5.889831

H 3.725724 4.827882 5.704026

H 2.316833 3.919836 6.323916

C 1.757364 4.951722 3.812537

H 1.395640 4.923635 2.765067

H 0.869427 4.989389 4.475777

H 2.324459 5.896284 3.944635

C 3.881269 3.720953 3.204641

H 4.541432 2.856921 3.420976

H 3.583515 3.658332 2.138757

H 4.475927 4.649131 3.332016

C -0.786182 0.839969 -3.732493

H -0.343887 -0.160773 -3.911760

H -1.880389 0.716824 -3.601073

H -0.627916 1.440936 -4.650481

C -0.780430 2.948252 -2.377486

H -0.327451 3.525921 -1.546827

H -0.641914 3.536771 -3.307934

H -1.868906 2.869909 -2.180364

C 1.377460 1.700252 -2.804575

H 1.895626 2.244274 -1.989556

H 1.865187 0.710423 -2.911628

H 1.541425 2.268600 -3.743443

Br 1.205660 -3.069744 2.434800

**^2^A3_E_**

78

charge = 0; spin = 2

*E*_e_^S^: -3076.23364836

Zero-point correction=0.581823

Thermal correction to Energy=0.626698

Thermal correction to Enthalpy=0.627642

Thermal correction to Gibbs Free Energy=0.501172

C -1.263860 -1.063498 0.106095

C 0.106252 0.544879 1.149774

C -0.145501 1.437455 0.077055

C -0.970940 1.096381 -0.995179

C -1.547913 -0.213224 -0.956318

H -1.697132 -2.074121 0.137645

H 0.338427 2.425046 0.105282

H -2.215513 -0.574131 -1.748353

C 0.933717 0.817495 2.292439

C 1.611838 2.034938 2.558700

C 2.449215 2.191690 3.664518

H 1.464777 2.870745 1.858641

C 1.874047 -0.110604 4.242359

C 2.582241 1.054835 4.523315

H 1.954323 -0.992055 4.896933

H 3.236739 1.069362 5.404041

Ni 0.012263 -1.785261 2.670577

C -0.838912 -3.517234 2.267697

H -0.622094 -4.018866 1.309314

H -1.914906 -3.399700 2.495112

C 0.105342 -3.655590 3.339153

C -0.173137 -2.816419 4.456195

H -1.220727 -2.516293 4.661072

C 1.377023 -4.458679 3.245333

H 1.715840 -4.552020 2.195110

H 2.188393 -4.007525 3.847206

H 1.204790 -5.486946 3.634257

O 0.691737 -2.741742 5.511531

Si 0.280015 -2.344467 7.142984

C -0.253119 -3.971650 8.021550

C -0.596658 -3.661815 9.493722

H -1.440632 -2.946426 9.586439

H -0.898910 -4.592627 10.024009

H 0.267610 -3.239064 10.047710

C 0.908874 -4.983744 7.957376

H 1.201417 -5.206767 6.910614

H 1.811693 -4.617042 8.488701

H 0.613713 -5.944326 8.435825

C -1.490527 -4.565399 7.317773

H -2.361044 -3.877077 7.345832

H -1.288678 -4.812813 6.255041

H -1.804352 -5.507687 7.820318

C 1.875106 -1.644500 7.857371

H 2.733928 -2.315948 7.656822

H 2.098813 -0.655835 7.407650

H 1.795892 -1.506600 8.954997

C -1.087441 -1.043025 7.138104

H -0.793461 -0.187777 6.495434

H -2.057335 -1.431754 6.768120

H -1.252907 -0.654750 8.164202

N -0.467542 -0.730099 1.150146

N 1.059937 -0.258395 3.174288

C 3.171929 3.527791 3.922031

C -1.219528 2.090094 -2.145750

C 4.069872 3.465321 5.172431

H 4.859376 2.692709 5.075345

H 4.575120 4.441042 5.323221

H 3.486469 3.247771 6.089988

C 2.121756 4.645139 4.131931

H 1.466990 4.764872 3.245631

H 1.471157 4.420904 5.001688

H 2.620362 5.619947 4.317461

C 4.059878 3.879799 2.704572

H 4.824354 3.095575 2.530163

H 3.464584 3.980561 1.775116

H 4.586593 4.842904 2.871892

C -2.172147 1.512657 -3.210048

H -1.761858 0.594616 -3.677375

H -3.166569 1.267541 -2.784944

H -2.329297 2.255566 -4.018395

C -1.849980 3.385670 -1.581068

H -1.191071 3.873183 -0.834965

H -2.033667 4.117151 -2.396086

H -2.818871 3.172972 -1.085163

C 0.126291 2.429889 -2.830398

H 0.844301 2.888261 -2.121191

H 0.601558 1.518535 -3.246848

H -0.029878 3.147885 -3.663056

**ArBr**

18

charge = 0; spin = 1

*E*_e_^S^: -3033.47056455

Zero-point correction=0.115726

Thermal correction to Energy=0.127094

Thermal correction to Enthalpy=0.128038

Thermal correction to Gibbs Free Energy=0.077729

C -3.081126 1.048710 0.000102

C -1.681386 1.002006 0.000415

C -0.955237 2.205207 -0.000099

C -1.608168 3.449981 -0.000918

C -3.007000 3.482845 -0.001225

C -3.754617 2.287781 -0.000721

H -3.664163 0.117160 0.000500

H -1.152474 0.038650 0.001053

H -1.023650 4.380617 -0.001310

H -3.550442 4.439522 -0.001862

Br 0.945401 2.147707 0.000321

C -5.245160 2.398207 -0.001091

O -5.861952 3.450569 -0.001760

O -5.849102 1.181307 -0.000597

C -7.280498 1.209731 -0.000960

H -7.608723 0.155046 -0.001714

H -7.666014 1.734294 0.896823

H -7.665562 1.735545 -0.898190

**^2^[A3_E_-A4_E_]^‡^**

96

charge = 0; spin = 2

*E*_e_^S^: -6109.67074489

Zero-point correction=0.696020

Thermal correction to Energy=0.754541

Thermal correction to Enthalpy=0.755486

Thermal correction to Gibbs Free Energy=0.595502

C -1.285800 -2.146954 1.924399

C -0.370832 -0.204708 2.859994

C -0.543455 0.472376 1.625409

C -1.074245 -0.172120 0.505620

C -1.448680 -1.539022 0.682813

H -1.586992 -3.195172 2.081696

H -0.256531 1.530064 1.568357

H -1.863080 -2.131629 -0.145277

C 0.199740 0.390007 4.056477

C 0.747656 1.693125 4.131844

C 1.200860 2.231748 5.342719

H 0.798964 2.292131 3.211345

C 0.634159 0.081795 6.337528

C 1.133415 1.378698 6.476402

H 0.575679 -0.590452 7.207315

H 1.444401 1.714617 7.473393

Ni -0.497313 -2.237004 4.825488

C 0.551625 -3.286683 6.137296

H 1.223850 -2.711756 6.797169

H -0.096251 -3.999376 6.680012

C 1.103875 -3.716520 4.865694

C 0.479912 -4.739206 4.146421

H -0.379825 -5.285655 4.574929

C 2.381386 -3.107741 4.324893

H 2.319267 -2.932366 3.233443

H 2.606000 -2.145312 4.822089

H 3.239494 -3.793251 4.498366

O 0.902928 -5.100066 2.910750

Si 0.445134 -6.523080 2.048984

C 1.222942 -6.265405 0.317125

C 0.964812 -7.519399 -0.544235

H -0.118735 -7.718148 -0.684423

H 1.405232 -7.388238 -1.557721

H 1.422229 -8.431167 -0.106048

C 2.741552 -6.040752 0.470776

H 2.962043 -5.156456 1.102168

H 3.249617 -6.916697 0.925480

H 3.208240 -5.871533 -0.525255

C 0.585378 -5.030482 -0.352496

H -0.502873 -5.165882 -0.525893

H 0.718758 -4.113827 0.257616

H 1.053877 -4.845494 -1.345000

C 1.157038 -8.007990 2.971272

H 0.763131 -8.039882 4.008041

H 0.882070 -8.963058 2.478376

H 2.262636 -7.956545 3.032721

C -1.440989 -6.635123 2.003410

H -1.860232 -6.738232 3.025527

H -1.898136 -5.738755 1.538081

H -1.767274 -7.523557 1.424050

N -0.751623 -1.526295 2.998630

N 0.160184 -0.414541 5.180649

C 1.696625 3.687244 5.418140

C -1.268818 0.514281 -0.855544

C 2.207930 4.049754 6.825313

H 3.054362 3.404174 7.135935

H 2.566454 5.099024 6.836226

H 1.410350 3.964005 7.590675

C 0.512649 4.623621 5.070585

H 0.146265 4.453794 4.038131

H -0.340722 4.454524 5.758973

H 0.820252 5.687399 5.150481

C 2.846948 3.906994 4.408505

H 3.709421 3.248359 4.637084

H 2.531641 3.700155 3.366286

H 3.198931 4.958956 4.447493

C -2.767721 0.468966 -1.239775

H -3.147524 -0.570316 -1.301988

H -3.385899 1.009316 -0.494253

H -2.928254 0.945210 -2.229452

C -0.814714 1.985197 -0.836455

H 0.262115 2.081921 -0.589651

H -0.968407 2.440289 -1.835784

H -1.392503 2.586662 -0.105515

C -0.440671 -0.240491 -1.923994

H 0.640901 -0.213957 -1.680314

H -0.741110 -1.304249 -2.005162

H -0.580209 0.225421 -2.921735

Br -2.792379 -3.476922 5.082214

C -2.484242 -1.700292 5.879722

C -3.026342 -0.579289 5.198196

C -2.219978 -1.630958 7.272263

C -3.126194 0.641065 5.864758

H -3.313881 -0.667104 4.141772

C -2.336611 -0.403199 7.922644

H -1.897734 -2.529072 7.816383

C -2.761191 0.755148 7.228533

H -3.490175 1.529205 5.329285

H -2.092007 -0.307820 8.991446

C -2.798604 2.037064 7.970128

O -2.504887 2.179819 9.149287

O -3.193251 3.084120 7.182235

C -3.254999 4.347561 7.844360

H -2.254492 4.655048 8.213961

H -3.622964 5.071673 7.094702

H -3.941552 4.311182 8.715044

**^2^A4_E_**

96

charge = 0; spin = 2

*E*_e_^S^: -6109.73173473

Zero-point correction=0.699143

Thermal correction to Energy=0.758058

Thermal correction to Enthalpy=0.759003

Thermal correction to Gibbs Free Energy=0.595560

C -1.371354 -1.048277 -0.199876

C 0.023487 0.436362 0.918797

C -0.265858 1.440418 -0.020251

C -1.143809 1.202090 -1.096274

C -1.704480 -0.089392 -1.163998

H -1.805158 -2.059811 -0.236497

H 0.199524 2.428888 0.095109

H -2.406926 -0.369631 -1.959575

C 0.910556 0.630617 2.091197

C 1.555988 1.840968 2.395596

C 2.366290 1.963907 3.541669

H 1.419775 2.703435 1.728868

C 1.811511 -0.362130 3.986877

C 2.480482 0.813386 4.347461

H 1.884308 -1.267375 4.608914

H 3.084737 0.811568 5.263615

Ni 0.036291 -2.121841 2.270850

C -0.880673 -3.770984 1.627809

H -0.629494 -3.908318 0.558471

H -1.918727 -3.410645 1.762312

C -0.543382 -4.955538 2.439615

C -1.055197 -5.071313 3.708379

H -1.673774 -4.253268 4.123209

C 0.337022 -6.031796 1.857516

H 1.251656 -5.599597 1.403742

H 0.635833 -6.771047 2.624279

H -0.196788 -6.574433 1.046113

O -0.805263 -6.137872 4.503664

Si -1.387754 -6.358536 6.115580

C -3.199365 -7.004655 6.023449

C -3.688187 -7.328904 7.450849

H -3.686839 -6.434222 8.108322

H -4.733709 -7.710266 7.423079

H -3.069578 -8.110203 7.940317

C -3.239152 -8.279454 5.156439

H -2.861229 -8.090217 4.130869

H -2.632792 -9.100725 5.592321

H -4.283440 -8.654392 5.066816

C -4.111088 -5.928731 5.398856

H -4.112858 -4.985655 5.983610

H -3.811883 -5.677677 4.360532

H -5.162381 -6.292880 5.358100

C -0.210992 -7.648770 6.819615

H -0.226847 -8.581757 6.221401

H 0.827609 -7.260094 6.812043

H -0.470855 -7.905819 7.866706

C -1.244790 -4.722605 7.043666

H -0.179809 -4.419639 7.112763

H -1.791629 -3.897924 6.542335

H -1.636915 -4.816527 8.077294

N -0.526396 -0.803335 0.813242

N 1.048953 -0.459384 2.888469

C 3.064166 3.295995 3.864960

C -1.452303 2.313502 -2.113603

C 3.904916 3.207646 5.152135

H 4.707844 2.447205 5.071555

H 4.391919 4.183864 5.348190

H 3.283483 2.964419 6.037566

C 1.990025 4.394824 4.051990

H 1.377454 4.537988 3.139314

H 1.301794 4.144548 4.884395

H 2.473664 5.365600 4.285605

C 3.998714 3.675999 2.691247

H 4.777339 2.902747 2.532514

H 3.443990 3.797714 1.739290

H 4.508640 4.637725 2.905378

C -2.433905 1.841094 -3.201994

H -2.027940 0.990642 -3.786346

H -3.410303 1.534725 -2.775218

H -2.629120 2.668070 -3.913710

C -2.081575 3.517148 -1.371038

H -1.402061 3.932983 -0.600324

H -2.309998 4.331372 -2.089115

H -3.026218 3.228041 -0.867791

C -0.134464 2.754238 -2.795371

H 0.601859 3.145766 -2.065136

H 0.340121 1.909392 -3.334349

H -0.336129 3.560047 -3.530743

Br -1.743073 -1.520582 3.888334

C 1.670485 -2.836757 1.540981

C 2.076898 -2.708663 0.195901

C 2.568934 -3.441661 2.450785

C 3.338608 -3.152094 -0.228823

H 1.399954 -2.254392 -0.546057

C 3.832905 -3.882399 2.037370

H 2.273342 -3.594780 3.503330

C 4.233376 -3.739636 0.691266

H 3.643143 -3.046928 -1.280585

H 4.535331 -4.352570 2.742927

C 5.586908 -4.224084 0.307742

O 5.846332 -4.043942 -1.020875

O 6.397495 -4.728819 1.069941

C 7.131065 -4.493074 -1.452599

H 7.943059 -3.960655 -0.915448

H 7.258420 -5.579612 -1.267181

H 7.184985 -4.280776 -2.535960

**^2^[A4_E_-A1]^‡^**

96

charge = 0; spin = 2

*E*_e_^S^: -6109.72415021

Zero-point correction=0.698137

Thermal correction to Energy=0.756507

Thermal correction to Enthalpy=0.757452

Thermal correction to Gibbs Free Energy=0.596460

C -1.608929 -0.831953 0.251436

C -0.046231 0.699479 1.037079

C -0.367273 1.582727 -0.006755

C -1.347679 1.259507 -0.965179

C -1.977899 0.007447 -0.804560

H -2.095929 -1.810933 0.381516

H 0.154981 2.547852 -0.062894

H -2.762179 -0.333273 -1.492864

C 0.941775 0.993286 2.096529

C 1.735139 2.151430 2.146781

C 2.647012 2.359249 3.200719

H 1.642247 2.899942 1.347453

C 1.881602 0.219076 4.080389

C 2.700571 1.348568 4.183990

H 1.884944 -0.576007 4.842736

H 3.375971 1.423592 5.045928

Ni -0.014784 -1.645260 2.736446

C -0.684726 -3.507232 2.005168

H -0.703368 -3.456210 0.901575

H -1.592211 -3.018880 2.433470

C -0.516069 -4.887159 2.499981

C -0.974186 -5.220437 3.744393

H -1.429789 -4.445132 4.387990

C 0.162085 -5.900372 1.613866

H 1.206140 -5.597549 1.384431

H 0.187239 -6.894923 2.096758

H -0.363730 -5.998590 0.638855

O -0.859382 -6.470532 4.255096

Si -1.382220 -6.994069 5.813667

C -3.282815 -7.305750 5.749344

C -3.737768 -7.910089 7.094346

H -3.540425 -7.228688 7.948442

H -4.834008 -8.104113 7.079652

H -3.238633 -8.877842 7.311034

C -3.596989 -8.288981 4.603699

H -3.253171 -7.900000 3.623398

H -3.118155 -9.278270 4.759153

H -4.694246 -8.463087 4.531630

C -4.027875 -5.977489 5.505328

H -3.829072 -5.230191 6.301241

H -3.751175 -5.515420 4.535405

H -5.127509 -6.151334 5.483895

C -0.417444 -8.590958 6.069285

H -0.639176 -9.332613 5.275935

H 0.671423 -8.382489 6.042525

H -0.651690 -9.053481 7.049687

C -0.902803 -5.694824 7.092694

H 0.201425 -5.597563 7.135336

H -1.310963 -4.690837 6.856470

H -1.256815 -5.982016 8.104262

N -0.662047 -0.514627 1.155158

N 1.024083 0.036022 3.060498

C 3.516538 3.627476 3.241909

C -1.688704 2.241071 -2.098505

C 4.438560 3.653540 4.475139

H 5.141229 2.795982 4.485679

H 5.046911 4.580139 4.467724

H 3.863579 3.642285 5.423072

C 2.596434 4.871271 3.288839

H 1.934705 4.933498 2.401610

H 1.952093 4.858661 4.191090

H 3.206511 5.797591 3.315659

C 4.395521 3.679333 1.968867

H 5.063199 2.796299 1.907501

H 3.786396 3.707326 1.043058

H 5.029908 4.589535 1.978903

C -2.781959 1.686489 -3.030524

H -2.469624 0.742110 -3.520567

H -3.732237 1.499855 -2.490534

H -2.996805 2.420116 -3.833097

C -2.192134 3.569117 -1.482744

H -1.429646 4.041876 -0.831763

H -2.440090 4.292676 -2.286326

H -3.104170 3.407839 -0.873219

C -0.414575 2.507366 -2.936258

H 0.400028 2.947783 -2.327058

H -0.030005 1.571344 -3.389496

H -0.639265 3.218705 -3.757367

Br -0.725996 -1.871064 5.066564

C 1.365017 -2.796394 2.036449

C 1.892587 -2.642630 0.733260

C 2.162473 -3.431833 3.018689

C 3.200038 -3.047095 0.434721

H 1.277899 -2.189280 -0.061192

C 3.473973 -3.820112 2.726386

H 1.738006 -3.622395 4.017134

C 4.011382 -3.631129 1.432851

H 3.606855 -2.912988 -0.578176

H 4.111926 -4.289488 3.490985

C 5.408421 -4.071787 1.181915

O 5.797806 -3.855701 -0.110367

O 6.151434 -4.571948 2.013200

C 7.132007 -4.262000 -0.414851

H 7.869414 -3.720649 0.213351

H 7.270227 -5.348606 -0.237522

H 7.291584 -4.025315 -1.482697

**3**

50

charge = 0; spin = 1

*E*_e_^S^: -1217.72867029

Zero-point correction=0.366487

Thermal correction to Energy=0.396752

Thermal correction to Enthalpy=0.397696

Thermal correction to Gibbs Free Energy=0.301596

C 0.494163 -1.858148 2.010311

H 0.571383 -1.817322 0.900286

H 0.024900 -0.897294 2.319576

C -0.402732 -3.004323 2.422835

C -1.514350 -2.754010 3.159123

H -1.757097 -1.713491 3.456348

C -0.022238 -4.390306 1.981617

H 0.987708 -4.669027 2.353271

H -0.747318 -5.141912 2.345610

H 0.023171 -4.458731 0.871926

O -2.386096 -3.719957 3.554749

Si -3.752584 -3.497569 4.574947

C -4.529196 -5.246952 4.664619

C -5.788733 -5.200102 5.554458

H -6.562104 -4.512385 5.152294

H -6.252716 -6.209471 5.620038

H -5.558278 -4.880283 6.592481

C -3.502381 -6.229437 5.264858

H -2.571397 -6.266612 4.663449

H -3.222967 -5.958397 6.304316

H -3.925683 -7.258213 5.296693

C -4.910665 -5.706837 3.242387

H -5.676340 -5.048243 2.781783

H -4.030057 -5.724074 2.568619

H -5.336148 -6.734847 3.270193

C -3.153226 -2.869727 6.252807

H -2.429949 -3.571656 6.714455

H -2.648826 -1.887153 6.146144

H -3.998835 -2.733101 6.958076

C -4.901038 -2.224444 3.780335

H -4.396775 -1.240661 3.685278

H -5.219204 -2.540218 2.766446

H -5.811989 -2.069586 4.394615

C 1.902822 -1.914000 2.584792

C 3.034509 -1.741954 1.761727

C 2.106922 -2.122210 3.966499

C 4.330267 -1.765505 2.294279

H 2.893937 -1.585360 0.679840

C 3.395529 -2.146148 4.507274

H 1.231512 -2.273914 4.617991

C 4.521601 -1.967229 3.676146

H 5.205385 -1.630798 1.643126

H 3.563410 -2.304916 5.583072

C 5.872433 -2.004066 4.309434

O 6.872107 -1.821998 3.403349

O 6.081295 -2.175411 5.499729

C 8.195349 -1.845349 3.944403

H 8.329762 -1.046045 4.701723

H 8.408204 -2.818010 4.433372

H 8.879858 -1.685913 3.091858

**^2^[A3_Z_-A4_Z_]^‡^**

96

charge = 0; spin = 2

*E*_e_^S^: -6109.67691102

Zero-point correction=0.696264

Thermal correction to Energy=0.754661

Thermal correction to Enthalpy=0.755605

Thermal correction to Gibbs Free Energy=0.596352

C -0.759901 -2.025103 2.116550

C -0.068338 -0.069012 3.197493

C -0.102586 0.628782 1.965881

C -0.445621 -0.012738 0.771267

C -0.781232 -1.395109 0.875307

H -1.036917 -3.087429 2.212607

H 0.144845 1.697967 1.965716

H -1.058772 -1.984142 -0.010675

C 0.312687 0.520530 4.474723

C 0.735857 1.856152 4.655413

C 0.999551 2.377329 5.930362

H 0.834281 2.499506 3.769117

C 0.508559 0.145996 6.776360

C 0.876978 1.471135 7.015254

H 0.416575 -0.569357 7.607799

H 1.045784 1.786585 8.052511

Ni -0.302241 -2.158418 5.088237

C 0.596132 -3.186013 6.513189

H 1.379156 -2.562864 6.981818

H -0.076902 -3.665146 7.248432

C 1.038204 -4.045366 5.421160

C 2.164406 -3.764926 4.653451

H 2.439827 -4.428400 3.811206

C 0.374211 -5.387965 5.184988

H 0.732127 -6.132696 5.929136

H -0.726842 -5.334588 5.296000

H 0.595305 -5.792731 4.175814

O 2.977805 -2.710174 4.899009

Si 4.270577 -2.174632 3.892697

C 5.251349 -0.963575 5.009283

C 6.485481 -0.451599 4.237495

H 6.205660 0.086367 3.307151

H 7.063346 0.262947 4.865342

H 7.178396 -1.272934 3.958322

C 5.702417 -1.703182 6.285633

H 4.836770 -2.102384 6.851954

H 6.380448 -2.552742 6.059793

H 6.257234 -1.010416 6.957294

C 4.348734 0.225792 5.394996

H 4.030252 0.814095 4.509674

H 3.431754 -0.103073 5.924383

H 4.896340 0.920452 6.071349

C 5.300776 -3.676849 3.383412

H 4.711514 -4.374904 2.753984

H 6.182003 -3.363306 2.786526

H 5.666649 -4.240474 4.265149

C 3.530421 -1.349574 2.365735

H 2.917924 -2.077773 1.795056

H 2.868203 -0.505087 2.643004

H 4.321141 -0.967302 1.687532

N -0.398301 -1.407642 3.264254

N 0.211560 -0.335625 5.553637

C 1.366643 3.860941 6.110770

C -0.472300 0.693453 -0.593267

C 1.600330 4.219590 7.590454

H 2.435025 3.637017 8.030337

H 1.860934 5.293649 7.680169

H 0.694978 4.043759 8.206154

C 0.199521 4.727381 5.576195

H 0.034336 4.570601 4.491254

H -0.748499 4.479877 6.096194

H 0.413153 5.805628 5.731293

C 2.656117 4.179000 5.317850

H 3.508584 3.570076 5.682233

H 2.536273 3.976169 4.234548

H 2.925493 5.250026 5.429863

C -1.895832 0.594214 -1.193154

H -2.216751 -0.458201 -1.326062

H -2.639230 1.093091 -0.538658

H -1.931080 1.083978 -2.188547

C -0.091019 2.181288 -0.486091

H 0.934648 2.317593 -0.086929

H -0.121292 2.651250 -1.489814

H -0.792813 2.742289 0.164033

C 0.535229 -0.003988 -1.539691

H 1.566293 0.054476 -1.135168

H 0.291189 -1.075153 -1.686092

H 0.528429 0.481457 -2.537821

Br -2.589116 -3.328113 5.049779

C -2.600340 -1.564947 5.871250

C -3.034512 -0.463570 5.096919

C -2.516292 -1.472929 7.280367

C -3.243729 0.765718 5.724076

H -3.162736 -0.569452 4.011192

C -2.733664 -0.236555 7.888197

H -2.253414 -2.357661 7.876119

C -3.076309 0.903882 7.122833

H -3.539891 1.640748 5.128598

H -2.636609 -0.121138 8.978250

C -3.238033 2.194910 7.832595

O -3.102661 2.359639 9.037169

O -3.553312 3.221772 6.984282

C -3.741593 4.489368 7.615040

H -2.805506 4.838809 8.098447

H -4.041044 5.190822 6.815102

H -4.527657 4.435092 8.395795

**^2^A4_Z_**

96

charge = 0; spin = 2

*E*_e_^S^: -6109.72637448

Zero-point correction=0.699000

Thermal correction to Energy=0.757761

Thermal correction to Enthalpy=0.758705

Thermal correction to Gibbs Free Energy=0.596937

C -1.307490 -1.362974 0.572551

C -0.151141 0.398383 1.548504

C 0.389350 0.752198 0.300965

C 0.082441 0.024257 -0.864958

C -0.792872 -1.067915 -0.694156

H -1.994927 -2.211341 0.710955

H 1.067476 1.614546 0.243005

H -1.088441 -1.705161 -1.537709

C 0.165627 1.103759 2.809150

C 1.045110 2.196109 2.902703

C 1.307262 2.810971 4.142853

H 1.533813 2.571283 1.993058

C -0.208200 1.163309 5.101960

C 0.649614 2.257891 5.260597

H -0.709854 0.688078 5.959799

H 0.794501 2.663455 6.270164

Ni -1.823681 -0.882801 3.565472

C -3.152996 -2.272848 2.968169

H -3.630398 -1.768548 2.105039

H -2.388979 -3.013923 2.653851

C -4.136943 -2.873838 3.874029

C -3.934603 -4.118879 4.406367

H -4.700716 -4.565463 5.071882

C -5.418488 -2.145572 4.189469

H -5.226260 -1.215547 4.763327

H -5.946344 -1.832997 3.262344

H -6.113213 -2.775478 4.780937

O -2.847440 -4.874857 4.137364

Si -2.341362 -6.284328 4.990117

C -0.731705 -6.800456 4.084442

C -0.246709 -8.153474 4.645876

H -0.983017 -8.967605 4.478792

H 0.699827 -8.460849 4.147177

H -0.037501 -8.104583 5.735438

C 0.349533 -5.724139 4.312562

H 0.016323 -4.720516 3.978582

H 0.626314 -5.634284 5.383143

H 1.275716 -5.985216 3.752286

C -1.015556 -6.937162 2.574739

H -1.773605 -7.719456 2.359383

H -1.382170 -5.984401 2.142069

H -0.086112 -7.222420 2.032184

C -2.071524 -5.834350 6.799181

H -1.425935 -4.936302 6.878936

H -3.037269 -5.595833 7.290237

H -1.602793 -6.667206 7.362868

C -3.704088 -7.587204 4.825710

H -4.658503 -7.214367 5.251736

H -3.889725 -7.857223 3.766498

H -3.438142 -8.513300 5.376100

N -1.009361 -0.655548 1.678175

N -0.454752 0.600404 3.907719

C 2.264007 4.012110 4.231954

C 0.685540 0.425025 -2.221142

C 2.405708 4.529043 5.675604

H 2.818876 3.755858 6.354405

H 3.099416 5.393336 5.696844

H 1.436188 4.872007 6.089856

C 1.716594 5.159793 3.349271

H 1.632645 4.862699 2.284588

H 0.712244 5.482784 3.690163

H 2.394612 6.036567 3.398985

C 3.661594 3.585198 3.721836

H 4.076786 2.759983 4.335062

H 3.633874 3.242535 2.667921

H 4.365813 4.440793 3.776061

C 0.221245 -0.507727 -3.355095

H 0.527859 -1.558770 -3.179707

H -0.879434 -0.484571 -3.486938

H 0.675845 -0.186322 -4.313503

C 0.247896 1.870566 -2.560341

H 0.589821 2.599011 -1.798048

H 0.677385 2.180663 -3.535091

H -0.855811 1.948826 -2.631581

C 2.229258 0.358032 -2.128220

H 2.630329 1.043300 -1.354773

H 2.571842 -0.667420 -1.882278

H 2.682943 0.647724 -3.098260

Br -1.171229 -2.041511 5.575230

C -3.243365 0.438605 3.511380

C -3.784106 0.976760 2.325855

C -3.682295 0.963279 4.748493

C -4.723078 2.019096 2.364979

H -3.475226 0.583569 1.343153

C -4.619536 2.004369 4.794267

H -3.292147 0.547461 5.691652

C -5.148713 2.545823 3.602836

H -5.135971 2.433990 1.433615

H -4.966252 2.418098 5.753948

C -6.138206 3.651640 3.713392

O -6.529683 4.146081 4.759648

O -6.581496 4.076383 2.493225

C -7.538465 5.135215 2.532705

H -7.119604 6.035830 3.027488

H -7.794706 5.359996 1.481277

H -8.447029 4.832993 3.093361

**^2^[A4_Z_-A1]^‡^**

96

charge = 0; spin = 2

*E*_e_^S^: -6109.72519251

Zero-point correction=0.698267

Thermal correction to Energy=0.756484

Thermal correction to Enthalpy=0.757428

Thermal correction to Gibbs Free Energy=0.597539

C -1.593241 -1.086164 0.822872

C -0.502341 0.680982 1.866416

C 0.535410 0.616239 0.922207

C 0.519474 -0.331494 -0.119964

C -0.595920 -1.194501 -0.151858

H -2.464523 -1.759196 0.806094

H 1.367989 1.328993 1.000030

H -0.707506 -1.960640 -0.930004

C -0.568516 1.677308 2.957083

C 0.426609 2.631903 3.224192

C 0.278157 3.557644 4.276352

H 1.333014 2.650246 2.603105

C -1.874507 2.497047 4.700642

C -0.917821 3.467208 5.019038

H -2.829210 2.417448 5.244285

H -1.127088 4.149781 5.852776

Ni -2.939822 0.080649 3.321484

C -3.756163 -1.852388 3.208083

H -4.258593 -1.268984 2.396808

H -2.991856 -2.532137 2.795266

C -4.754680 -2.584697 4.014792

C -4.506283 -3.858869 4.435794

H -5.280583 -4.408258 5.005732

C -6.083136 -1.950250 4.335391

H -5.963228 -1.006309 4.905389

H -6.627640 -1.663688 3.409952

H -6.729270 -2.637329 4.918358

O -3.359828 -4.536989 4.160209

Si -2.894671 -6.044391 4.852374

C -1.516109 -6.684346 3.679441

C -0.985639 -8.031117 4.213881

H -1.777724 -8.807920 4.259324

H -0.184131 -8.422151 3.547817

H -0.547050 -7.934700 5.229079

C -0.364332 -5.660526 3.625899

H -0.712158 -4.674513 3.257197

H 0.099615 -5.500417 4.621116

H 0.437180 -6.018080 2.940757

C -2.098895 -6.876609 2.264632

H -2.907175 -7.637117 2.245062

H -2.516866 -5.930320 1.864195

H -1.306800 -7.221538 1.562417

C -2.293365 -5.754084 6.615049

H -1.399418 -5.100179 6.653391

H -3.090954 -5.260348 7.207817

H -2.045834 -6.712525 7.116531

C -4.398783 -7.192940 4.868126

H -5.176653 -6.829316 5.570608

H -4.861248 -7.281464 3.864435

H -4.108425 -8.210272 5.202906

N -1.559187 -0.182802 1.820700

N -1.706673 1.614470 3.699975

C 1.378522 4.592836 4.565737

C 1.663813 -0.386679 -1.145417

C 1.014165 5.503541 5.752895

H 0.876158 4.927399 6.690070

H 1.830441 6.232447 5.929262

H 0.088135 6.082743 5.562367

C 1.584459 5.477058 3.312095

H 1.891875 4.882065 2.428689

H 0.654204 6.019292 3.047512

H 2.378275 6.228918 3.500597

C 2.695126 3.850757 4.900138

H 2.575119 3.205567 5.793801

H 3.035311 3.208314 4.063207

H 3.503255 4.581419 5.110050

C 1.442907 -1.491400 -2.195294

H 1.391050 -2.498230 -1.733667

H 0.514747 -1.328954 -2.779759

H 2.287845 -1.499691 -2.912573

C 1.756156 0.975617 -1.874681

H 1.959069 1.810442 -1.174307

H 2.579854 0.955630 -2.617552

H 0.814690 1.206744 -2.412807

C 2.992390 -0.669749 -0.403138

H 3.228994 0.116215 0.341810

H 2.954356 -1.640966 0.130420

H 3.832917 -0.708440 -1.126155

Br -4.963826 1.410044 3.551555

C -2.426566 -1.144633 4.715603

C -3.008424 -0.882805 5.981954

C -1.211209 -1.868143 4.671497

C -2.366922 -1.280373 7.160282

H -3.968321 -0.347601 6.036934

C -0.577971 -2.277865 5.848124

H -0.750227 -2.111044 3.701300

C -1.146535 -1.991727 7.108728

H -2.816188 -1.052805 8.137990

H 0.370937 -2.835445 5.818161

C -0.444464 -2.486678 8.319510

O 0.548531 -3.202062 8.314442

O -1.035260 -2.065528 9.475637

C -0.418939 -2.530744 10.676527

H 0.634500 -2.188925 10.744470

H -1.011718 -2.112455 11.510300

H -0.418766 -3.639450 10.722941

**4**

50

charge = 0; spin = 1

*E*_e_^S^: -1217.72931679

Zero-point correction=0.366487

Thermal correction to Energy=0.396752

Thermal correction to Enthalpy=0.397696

Thermal correction to Gibbs Free Energy=0.301596

C 0.477888 -1.740640 2.014157

H 0.551577 -1.665176 0.907185

H 0.030193 -0.791661 2.376503

C -0.446109 -2.881908 2.386116

C -1.459351 -2.706462 3.269147

H -2.126142 -3.554317 3.524152

C -0.189698 -4.223492 1.756551

H 0.835982 -4.588548 1.984682

H -0.907252 -4.991879 2.107868

H -0.263187 -4.172612 0.647117

O -1.715988 -1.519132 3.886957

Si -3.015905 -1.194160 4.966190

C -2.747545 0.641297 5.447404

C -3.847113 1.069097 6.441975

H -3.823807 0.470923 7.377056

H -3.710403 2.134350 6.733270

H -4.865076 0.978938 6.007750

C -2.820828 1.518657 4.180649

H -2.057003 1.223006 3.432920

H -3.814806 1.462454 3.689814

H -2.642816 2.586148 4.440037

C -1.360255 0.800329 6.103342

H -1.270075 0.210357 7.039247

H -0.545002 0.481514 5.422327

H -1.179251 1.865684 6.369278

C -4.643919 -1.487690 4.054028

H -4.720722 -0.856415 3.146029

H -4.730053 -2.547354 3.736500

H -5.515282 -1.264771 4.703719

C -2.879120 -2.367284 6.440910

H -2.960854 -3.424282 6.113516

H -1.909528 -2.250847 6.965615

H -3.691196 -2.185170 7.174703

C 1.877581 -1.876938 2.590062

C 3.017274 -1.901538 1.760518

C 2.064591 -1.982578 3.987008

C 4.305504 -2.020513 2.299664

H 2.889765 -1.822988 0.668699

C 3.345393 -2.098636 4.533258

H 1.181776 -1.974639 4.645577

C 4.480483 -2.119447 3.694562

H 5.186944 -2.039362 1.643365

H 3.500652 -2.176457 5.619777

C 5.821862 -2.248080 4.334772

O 6.831585 -2.256234 3.421002

O 6.017267 -2.337046 5.536456

C 8.146489 -2.376986 3.968821

H 8.370888 -1.534710 4.654943

H 8.254221 -3.321067 4.541250

H 8.840723 -2.367618 3.109263

**^1^[A2_E_-A2_Z_]^‡^**

79

charge = 0; spin = 1

*E*_e_^S^: -5650.28027040

Zero-point correction=0.581527

Thermal correction to Energy=0.628628

Thermal correction to Enthalpy=0.629572

Thermal correction to Gibbs Free Energy=0.495977

C -0.817525 -0.819482 0.985961

C 0.245728 1.202732 1.379838

C 0.397308 1.414670 -0.003116

C -0.085186 0.479277 -0.938098

C -0.705856 -0.670060 -0.401150

H -1.280834 -1.720518 1.418507

H 0.913607 2.319967 -0.353341

H -1.102409 -1.463101 -1.048941

C 0.757398 2.117488 2.419990

C 1.379109 3.349156 2.151632

C 1.882274 4.161255 3.184502

H 1.470666 3.676042 1.106102

C 1.090861 2.429476 4.704031

C 1.724351 3.655376 4.494843

H 0.966337 2.022538 5.718895

H 2.093222 4.205787 5.370292

Ni -0.360759 0.009082 3.987631

C -2.099184 0.431512 4.776586

H -2.812627 0.837504 4.035956

H -2.017989 1.014212 5.709719

C -1.886312 -0.999437 4.787001

C -1.281003 -1.535807 6.006807

H -0.187286 -1.483922 6.179252

C -2.745007 -1.942381 3.955876

H -3.159005 -1.416691 3.072989

H -2.160375 -2.817480 3.603946

H -3.605833 -2.328316 4.546439

O -2.037921 -2.123365 6.914048

Si -1.389483 -2.926547 8.356601

C -2.979155 -3.400082 9.308025

C -2.579389 -4.135255 10.605108

H -1.962049 -3.500664 11.275228

H -3.487981 -4.425977 11.177694

H -2.010906 -5.067089 10.401647

C -3.846165 -4.323968 8.427477

H -4.131441 -3.834056 7.474211

H -3.327802 -5.273405 8.179807

H -4.784600 -4.591792 8.961753

C -3.770196 -2.120256 9.649700

H -3.198414 -1.439476 10.314084

H -4.045880 -1.552807 8.737299

H -4.712488 -2.381428 10.180512

C -0.384275 -4.382714 7.726056

H -1.013738 -5.105866 7.170428

H 0.408050 -4.021996 7.037046

H 0.106577 -4.920694 8.562901

C -0.310393 -1.667960 9.248574

H 0.559568 -1.385357 8.621105

H -0.872557 -0.745925 9.498045

H 0.085672 -2.095485 10.192767

N -0.373750 0.091964 1.867984

N 0.599470 1.663158 3.703731

C 2.560620 5.505360 2.867913

C 0.087382 0.722589 -2.448038

C 3.037734 6.223411 4.144121

H 3.784367 5.622947 4.702250

H 3.519270 7.185475 3.876142

H 2.196452 6.452845 4.829202

C 1.553633 6.424393 2.135350

H 1.199742 5.976855 1.184950

H 0.663467 6.624032 2.765812

H 2.027044 7.398045 1.891055

C 3.787815 5.254845 1.958574

H 4.527290 4.596978 2.458362

H 3.502998 4.774141 1.001238

H 4.289619 6.214348 1.715050

C -0.517095 -0.417628 -3.288474

H -0.029492 -1.390852 -3.077689

H -1.605795 -0.530673 -3.110640

H -0.377178 -0.203431 -4.367275

C -0.618929 2.044199 -2.834633

H -0.200396 2.913174 -2.288353

H -0.498929 2.241785 -3.920083

H -1.704374 1.997028 -2.612404

C 1.596354 0.827118 -2.776135

H 2.082425 1.661221 -2.231486

H 2.129178 -0.106882 -2.505817

H 1.742678 1.005284 -3.861748

Br 1.395797 -1.691633 4.267217

**^2^B1**

28

charge = 0; spin = 2

*E*_e_^S^: -4656.28499894

Zero-point correction=0.186938

Thermal correction to Energy=0.204337

Thermal correction to Enthalpy=0.205281

Thermal correction to Gibbs Free Energy=0.137777

C -1.176070 -0.147312 0.000146

C 0.736413 1.244216 -0.000036

C -0.057587 2.403355 -0.001757

C -1.450370 2.271942 -0.002538

C -2.009567 0.984735 -0.001555

H 0.409756 3.397505 -0.002501

H -3.100292 0.845104 -0.002091

C 2.210897 1.242963 0.000949

C 3.006853 2.400761 0.000737

C 4.399412 2.266996 0.001811

H 2.541189 3.395697 -0.000229

C 4.121020 -0.151794 0.003220

C 4.956429 0.978847 0.003054

H 6.046917 0.837372 0.003898

Ni 1.471330 -1.461677 0.002505

Br 1.469705 -3.725700 0.003677

N 0.180287 -0.007111 0.000859

N 2.764904 -0.009303 0.002175

H -2.095443 3.162915 -0.003892

H 5.045990 3.156878 0.001682

C 4.669124 -1.547763 0.004643

H 4.304741 -2.111569 0.888253

H 4.303460 -2.113928 -0.876907

H 5.774952 -1.546560 0.003955

C -1.726513 -1.542362 0.001126

H -1.363063 -2.108307 -0.881502

H -1.361817 -2.107606 0.883668

H -2.832337 -1.539310 0.001808

**^1^B2_E_**

61

charge = 0; spin = 1

*E*_e_^S^: -5414.59359190

Zero-point correction=0.437666

Thermal correction to Energy=0.475375

Thermal correction to Enthalpy=0.476319

Thermal correction to Gibbs Free Energy=0.366202

C -0.086122 -1.580479 -0.051908

C 1.021943 0.199246 1.004859

C -0.196220 0.783082 1.401615

C -1.393231 0.144203 1.063997

C -1.334002 -1.030091 0.302617

H -0.209993 1.729616 1.958539

H -2.254857 -1.528312 -0.035243

C 2.332272 0.859277 1.194080

C 2.459314 2.184333 1.651756

C 3.728971 2.764735 1.714451

H 1.569383 2.762359 1.932750

C 4.659760 0.682398 0.864156

C 4.833936 2.008654 1.305661

H 5.846845 2.435968 1.318774

Ni 3.014541 -1.750118 0.317032

Br 3.566126 -1.562889 -2.080855

C 2.877063 -3.719475 0.215891

H 3.078206 -4.203757 -0.752142

H 1.924101 -4.000257 0.698614

C 3.979219 -3.332994 1.047619

C 3.524028 -2.569149 2.169654

C 5.415769 -3.672497 0.757438

H 5.598955 -4.746425 0.980022

H 5.651460 -3.504776 -0.312460

H 6.113160 -3.079027 1.378071

N 1.073702 -0.997518 0.353163

N 3.420382 0.120722 0.829343

C -0.008262 -2.801588 -0.924283

H 0.890924 -2.745318 -1.570033

H 0.069406 -3.729844 -0.321395

H -0.917605 -2.889385 -1.549395

C 5.821421 -0.146004 0.416175

H 5.994783 -0.968263 1.137549

H 5.583365 -0.617429 -0.562420

H 6.741701 0.462485 0.337181

H 3.853745 3.801299 2.061831

H -2.361045 0.573088 1.363893

O 4.417909 -1.974315 3.014586

Si 4.116642 -1.595302 4.671417

C 3.469848 -3.152215 5.525100

H 4.181102 -3.996879 5.427062

H 3.298152 -2.970417 6.606028

H 2.502448 -3.472758 5.086803

C 5.828413 -1.050482 5.343747

C 5.685859 -0.703545 6.841046

H 6.667409 -0.384881 7.257318

H 4.973541 0.130630 7.012799

H 5.343523 -1.570807 7.443643

C 6.325414 0.188617 4.571733

H 7.320014 0.507383 4.956417

H 6.436292 -0.018964 3.488433

H 5.638610 1.053609 4.680144

C 6.837915 -2.203920 5.170243

H 6.946488 -2.496521 4.106061

H 7.841740 -1.897655 5.540223

H 6.541110 -3.108666 5.740472

C 2.821828 -0.224517 4.763326

H 1.871492 -0.553375 4.294376

H 2.599198 0.041843 5.817330

H 3.157119 0.691230 4.236606

H 2.521297 -2.785817 2.593116

**^3^B2_E_**

61

charge = 0; spin = 3

*E*_e_^S^: -5414.57739930

Zero-point correction=0.436031

Thermal correction to Energy=0.474499

Thermal correction to Enthalpy=0.475444

Thermal correction to Gibbs Free Energy=0.361721

C 0.106596 -0.601897 1.235088

C 1.967929 0.856418 1.334240

C 1.229737 1.804916 2.070497

C -0.102479 1.535008 2.384633

C -0.668464 0.320552 1.958714

H 1.696159 2.749018 2.382627

H -1.719485 0.081227 2.175614

C 3.353555 1.057406 0.893775

C 4.124608 2.201943 1.182159

C 5.400700 2.318016 0.629482

H 3.721056 2.995390 1.825598

C 5.083247 0.163553 -0.458512

C 5.874018 1.298110 -0.216679

H 6.858448 1.377579 -0.699825

Ni 2.700677 -1.543851 0.059679

Br 2.090226 -2.970100 -1.702561

C 4.824816 -4.186061 0.599997

H 3.961450 -4.706033 0.156991

H 5.829495 -4.552592 0.338014

C 4.663292 -3.092622 1.404582

C 3.322402 -2.574374 1.742979

C 5.852574 -2.372067 1.994316

H 5.866904 -2.469124 3.099550

H 6.803770 -2.777506 1.598574

H 5.815585 -1.282561 1.788740

N 1.404062 -0.325764 0.931308

N 3.855599 0.043605 0.121481

C -0.461449 -1.909235 0.768042

H -0.257732 -2.062822 -0.311472

H 0.028777 -2.758102 1.287784

H -1.549046 -1.964022 0.959968

C 5.525049 -0.940918 -1.372812

H 5.582005 -1.908471 -0.834082

H 4.776959 -1.093584 -2.178385

H 6.510058 -0.719113 -1.823620

H 6.020494 3.202453 0.838784

H -0.701277 2.263386 2.951258

O 3.262273 -1.790729 2.898494

Si 2.757881 -2.390575 4.428272

C 3.695707 -3.987632 4.812624

H 4.775752 -3.808869 4.987556

H 3.281325 -4.483896 5.714298

H 3.608352 -4.699669 3.966310

C 3.178915 -0.987390 5.672413

C 2.915037 -1.497231 7.104859

H 3.140539 -0.698413 7.846579

H 1.855434 -1.792286 7.257999

H 3.549298 -2.370781 7.362891

C 2.297766 0.245481 5.392037

H 2.551331 1.070585 6.095544

H 2.441372 0.625571 4.360525

H 1.217391 0.025562 5.520118

C 4.663822 -0.598919 5.524141

H 4.887708 -0.231800 4.502338

H 4.926226 0.209413 6.243298

H 5.342260 -1.452828 5.732162

C 0.903714 -2.762230 4.375311

H 0.689537 -3.597417 3.676857

H 0.532169 -3.068337 5.375206

H 0.319333 -1.880060 4.044591

H 2.513069 -3.336027 1.671538

**^3^[B2_E_-B2_Z_]^‡^**

61

charge = 0; spin = 3

*E*_e_^S^: -5414.56116769

Zero-point correction=0.436265

Thermal correction to Energy=0.473740

Thermal correction to Enthalpy=0.474684

Thermal correction to Gibbs Free Energy=0.362606

C 0.113078 -1.836548 -0.159664

C 0.648290 0.285747 0.749136

C -0.714861 0.607376 0.893304

C -1.677260 -0.325223 0.498077

C -1.257540 -1.554082 -0.039839

H -1.015069 1.578391 1.310101

H -1.990076 -2.301393 -0.377523

C 1.759225 1.185556 1.099116

C 1.602520 2.513519 1.540156

C 2.742147 3.274056 1.813045

H 0.599760 2.946645 1.655943

C 4.119949 1.359078 1.212406

C 4.010018 2.690986 1.647827

H 4.925836 3.264859 1.850231

Ni 2.993371 -1.204304 0.234000

Br 4.026210 -1.324030 -1.895980

C 4.298759 -4.167669 0.449575

H 4.532390 -3.433043 -0.337318

H 4.629630 -5.207710 0.289338

C 3.628310 -3.827261 1.571344

C 3.154464 -2.413654 1.890270

C 3.331493 -4.861300 2.638360

H 2.247513 -4.896216 2.882537

H 3.651113 -5.875653 2.327416

H 3.858965 -4.611603 3.583004

N 1.038670 -0.925358 0.246465

N 3.000237 0.635133 0.944529

C 0.627110 -3.113555 -0.754661

H 1.264776 -2.889305 -1.636100

H 1.284612 -3.653192 -0.041561

H -0.200034 -3.780765 -1.059643

C 5.437509 0.674855 1.011580

H 5.461250 -0.267977 1.596510

H 5.553783 0.384630 -0.054623

H 6.281966 1.321281 1.313946

H 2.647360 4.316601 2.151151

H -2.748586 -0.097699 0.600870

O 3.991076 -1.848906 2.896782

Si 3.585032 -1.384740 4.492334

C 2.354427 -2.599320 5.270015

H 2.790156 -3.601593 5.449994

H 1.997600 -2.200894 6.242321

H 1.463530 -2.730273 4.621767

C 5.267582 -1.385115 5.427825

C 5.013653 -1.105950 6.923916

H 5.975541 -1.097141 7.484206

H 4.531152 -0.119970 7.092307

H 4.371036 -1.880477 7.392202

C 6.193089 -0.294559 4.852223

H 7.168644 -0.292848 5.388849

H 6.407481 -0.462598 3.777484

H 5.759354 0.721922 4.958285

C 5.939111 -2.763040 5.260328

H 6.114812 -2.999046 4.191473

H 6.921973 -2.783299 5.782597

H 5.324637 -3.581507 5.691222

C 2.772065 0.323860 4.515428

H 1.792672 0.294292 3.995203

H 2.583759 0.649787 5.559773

H 3.394383 1.096191 4.022217

H 2.100901 -2.462345 2.260510

**^3^B2_Z_**

61

charge = 0; spin = 3

*E*_e_^S^: -5414.58087599

Zero-point correction=0.435918

Thermal correction to Energy=0.474451

Thermal correction to Enthalpy=0.475395

Thermal correction to Gibbs Free Energy=0.361379

C -0.011006 -0.833791 0.965289

C 1.729569 0.714931 1.382555

C 0.856388 1.554499 2.102142

C -0.482202 1.184160 2.241733

C -0.919403 -0.019246 1.662309

H 1.223364 2.492803 2.539012

H -1.969040 -0.336281 1.743562

C 3.142969 1.026071 1.123509

C 3.797310 2.184215 1.588300

C 5.128523 2.401083 1.227627

H 3.266151 2.908277 2.220564

C 5.084072 0.320182 -0.034312

C 5.771725 1.467327 0.397937

H 6.810542 1.622403 0.072984

Ni 2.738921 -1.515961 -0.004166

Br 2.471459 -2.686832 -2.014156

C 5.676043 -2.989218 1.852935

H 5.789400 -2.088053 2.473252

H 6.576973 -3.581273 1.629505

C 4.451217 -3.373662 1.381222

C 3.226969 -2.609420 1.689019

C 4.286717 -4.634051 0.564342

H 3.781458 -4.430993 -0.403405

H 5.265283 -5.107452 0.354994

H 3.658534 -5.373002 1.107724

N 1.290009 -0.458539 0.831828

N 3.795664 0.108938 0.347467

C -0.431367 -2.135804 0.349047

H -0.063720 -2.213928 -0.694731

H 0.020753 -2.990652 0.894358

H -1.530615 -2.253385 0.372320

C 5.721634 -0.704291 -0.924308

H 5.883963 -1.646751 -0.359964

H 5.050636 -0.957213 -1.770822

H 6.694462 -0.348987 -1.311972

H 5.661520 3.297453 1.578031

H -1.184640 1.827822 2.791777

O 3.301900 -1.746616 2.787859

Si 2.825897 -2.202219 4.372801

C 3.523243 -3.909964 4.786124

H 4.631045 -3.918578 4.774344

H 3.177184 -4.249945 5.784059

H 3.179093 -4.655907 4.040391

C 3.533903 -0.835704 5.527807

C 3.284468 -1.249824 6.993533

H 3.667190 -0.466906 7.686205

H 2.204269 -1.380426 7.216260

H 3.799825 -2.197184 7.255217

C 2.837932 0.511163 5.249567

H 3.251295 1.304206 5.913233

H 2.990973 0.837061 4.200974

H 1.744515 0.464605 5.434221

C 5.048538 -0.686424 5.280731

H 5.258751 -0.363956 4.241032

H 5.479772 0.076959 5.966949

H 5.597748 -1.634662 5.457154

C 0.935070 -2.274725 4.442392

H 0.551603 -3.075485 3.776303

H 0.579018 -2.503658 5.468272

H 0.474550 -1.318846 4.121263

H 2.308635 -3.244166 1.684424

**^1^B2_Z_**

61

charge = 0; spin = 1

*E*_e_^S^: -5414.58589012

Zero-point correction=0.437539

Thermal correction to Energy=0.475340

Thermal correction to Enthalpy=0.476284

Thermal correction to Gibbs Free Energy=0.365260

C 0.118850 -1.589732 0.196076

C 1.221682 0.295996 1.083399

C -0.004777 0.937138 1.340682

C -1.197205 0.286029 1.016260

C -1.130005 -0.980543 0.423398

H -0.022532 1.948357 1.767412

H -2.044083 -1.514523 0.126399

C 2.529008 0.956232 1.290827

C 2.663241 2.218257 1.901161

C 3.923875 2.821797 1.933152

H 1.792667 2.727741 2.335967

C 4.809449 0.895356 0.735172

C 4.996931 2.165326 1.316726

H 5.990643 2.634837 1.268973

Ni 3.113812 -1.697062 0.402485

Br 3.252565 -1.757178 -2.072962

C 4.807239 -2.751690 0.629591

H 5.404991 -2.195129 1.368046

H 5.329588 -3.090322 -0.278031

C 3.656367 -3.462496 1.083140

C 2.980709 -2.765460 2.149136

C 3.160980 -4.740875 0.458526

H 2.114190 -4.966728 0.743182

H 3.224762 -4.695360 -0.647402

H 3.783564 -5.594917 0.805008

N 1.273852 -0.960501 0.550009

N 3.596998 0.282000 0.778675

C 0.215796 -2.937679 -0.447040

H 0.997954 -2.914189 -1.237676

H 0.528285 -3.704113 0.290152

H -0.756336 -3.246415 -0.874817

C 5.935737 0.209561 0.015203

H 6.491844 -0.472655 0.690487

H 5.533833 -0.398617 -0.819731

H 6.658055 0.953805 -0.371962

H 4.062157 3.805457 2.406231

H -2.167778 0.769015 1.203896

O 3.667880 -2.158525 3.173802

Si 3.087831 -1.902036 4.773195

C 2.589986 -3.574248 5.500672

H 3.436018 -4.290479 5.491344

H 2.240165 -3.467340 6.548209

H 1.759051 -4.025403 4.919965

C 4.590411 -1.119902 5.671221

C 4.213665 -0.840650 7.141234

H 5.074760 -0.386777 7.680704

H 3.364042 -0.131290 7.229907

H 3.939518 -1.767102 7.688327

C 4.974483 0.199884 4.971523

H 5.864367 0.652400 5.463762

H 5.224330 0.041471 3.902820

H 4.158320 0.950781 5.016106

C 5.782078 -2.097861 5.614489

H 6.061789 -2.340946 4.569333

H 6.673023 -1.649753 6.108504

H 5.564196 -3.052699 6.136813

C 1.585937 -0.756762 4.723640

H 0.771330 -1.200337 4.114871

H 1.183527 -0.584412 5.743342

H 1.840914 0.226337 4.279894

H 1.952126 -3.094892 2.405198

**^1^[B2_E_-B2_Z_]^‡^**

61

charge = 0; spin = 1

*E*_e_^S^: -5414.50825476

Zero-point correction=0.435205

Thermal correction to Energy=0.472949

Thermal correction to Enthalpy=0.473893

Thermal correction to Gibbs Free Energy=0.361819

C 0.034168 -2.220969 -1.222262

C 0.554357 -0.143326 -0.218175

C -0.745283 0.339858 -0.460979

C -1.675201 -0.486382 -1.094847

C -1.266703 -1.768091 -1.497624

H -1.017718 1.362976 -0.168705

H -1.954771 -2.432465 -2.040497

C 1.637444 0.682821 0.337694

C 1.491809 2.044828 0.667694

C 2.609677 2.766711 1.091436

H 0.514657 2.537822 0.574978

C 3.949853 0.753883 0.806633

C 3.854300 2.116060 1.138348

H 4.765718 2.664397 1.418837

Ni 2.673858 -1.924245 0.111153

Br 3.992138 -2.234214 -2.022923

C 2.585444 -3.736529 0.797305

H 2.877847 -4.518241 0.074300

H 1.636541 -3.921025 1.330955

C 3.665904 -3.051199 1.484210

C 3.314301 -2.351833 2.710664

C 5.091404 -3.570639 1.332176

H 5.246670 -4.512120 1.902719

H 5.283483 -3.769543 0.258343

H 5.846785 -2.836201 1.676963

N 0.918953 -1.427589 -0.545318

N 2.841420 0.037563 0.459015

C 0.500633 -3.569407 -1.678575

H 1.526989 -3.484615 -2.097009

H 0.560151 -4.277799 -0.827522

H -0.189252 -3.991206 -2.433852

C 5.271375 0.041036 0.783054

H 5.388097 -0.622040 1.663808

H 5.321795 -0.611447 -0.115818

H 6.111717 0.761179 0.781146

H 2.521626 3.831244 1.355020

H -2.696417 -0.131036 -1.297051

O 3.512848 -2.922545 3.882994

Si 3.137615 -2.326315 5.502610

C 2.502100 -0.559573 5.355178

H 1.560432 -0.490822 4.774576

H 2.303337 -0.148628 6.366747

H 3.253014 0.098606 4.872020

C 1.834487 -3.566491 6.157918

C 1.445021 -3.155543 7.594400

H 0.693866 -3.866304 8.004291

H 2.313401 -3.167099 8.286130

H 0.990102 -2.143609 7.635103

C 2.437762 -4.986588 6.160411

H 1.682111 -5.721452 6.515430

H 2.758447 -5.299041 5.145795

H 3.315009 -5.067694 6.835092

C 0.590269 -3.532903 5.246586

H 0.831977 -3.819684 4.202270

H -0.172955 -4.252417 5.616337

H 0.110355 -2.532427 5.224074

C 4.786483 -2.408333 6.397832

H 5.507604 -1.694024 5.951337

H 4.669499 -2.147244 7.469636

H 5.227594 -3.422873 6.336055

H 2.869818 -1.337302 2.719039

**^2^B3_E_**

60

charge = 0; spin = 2

*E*_e_^S^: -2840.45233209

Zero-point correction=0.435644

Thermal correction to Energy=0.471226

Thermal correction to Enthalpy=0.472171

Thermal correction to Gibbs Free Energy=0.367823

C -0.164679 -1.588046 -0.404418

C 0.764946 0.298630 0.694027

C -0.478307 0.974314 0.625136

C -1.574970 0.352718 0.045928

C -1.403331 -0.942139 -0.499285

H -0.558206 2.000669 1.009015

H -2.234719 -1.455512 -1.002949

C 1.977466 0.911826 1.183908

C 2.060681 2.215865 1.733249

C 3.304130 2.775067 1.999335

H 1.141569 2.784597 1.932314

C 4.346520 0.722153 1.195542

C 4.468552 2.028514 1.679687

H 5.469857 2.469435 1.788527

Ni 2.670236 -1.727443 0.746436

C 2.607973 -3.698555 0.511495

H 2.690725 -4.152980 -0.490285

H 1.759650 -4.078691 1.109082

C 3.821269 -3.317314 1.179396

C 3.589877 -2.618461 2.399338

C 5.195962 -3.600723 0.629945

H 5.465124 -4.659384 0.838146

H 5.227589 -3.465327 -0.469689

H 5.973004 -2.963101 1.089245

N 0.903950 -1.008264 0.224840

N 3.123079 0.140807 1.010947

C -0.002371 -2.952082 -1.016099

H 0.943090 -3.014554 -1.587967

H 0.037219 -3.745110 -0.242917

H -0.850623 -3.177659 -1.689684

C 5.559882 -0.079597 0.820004

H 5.722064 -0.898942 1.547016

H 5.419892 -0.546238 -0.176109

H 6.465161 0.556251 0.797332

H 3.385370 3.788412 2.419839

H -2.545645 0.866414 -0.018448

O 4.610032 -2.127702 3.156510

Si 4.411345 -1.606819 4.800647

C 3.692090 -3.060051 5.773037

H 4.357005 -3.946560 5.740269

H 3.536819 -2.785056 6.836550

H 2.705448 -3.361633 5.365254

C 6.187817 -1.169159 5.378276

C 6.140809 -0.836461 6.885531

H 7.157318 -0.568738 7.250723

H 5.479296 0.028058 7.104862

H 5.790860 -1.694773 7.496114

C 6.718404 0.052859 4.601720

H 7.742613 0.313274 4.951253

H 6.779203 -0.142848 3.512638

H 6.082006 0.950223 4.745606

C 7.118934 -2.375911 5.141346

H 7.159951 -2.656767 4.069472

H 8.154772 -2.134321 5.468828

H 6.796212 -3.271757 5.711822

C 3.225285 -0.144774 4.833614

H 2.218683 -0.451113 4.482525

H 3.113759 0.253887 5.863156

H 3.570843 0.676768 4.174399

H 2.628889 -2.785142 2.929453

**^2^[B3_E_-B4_E_]^‡^**

78

charge = 0; spin = 2

*E*_e_^S^: -5873.90305451

Zero-point correction=0.550955

Thermal correction to Energy=0.599913

Thermal correction to Enthalpy=0.600857

Thermal correction to Gibbs Free Energy=0.463733

C -0.433718 -1.397284 0.449875

C 0.863962 0.358209 1.312279

C -0.264730 0.933188 1.934138

C -1.511196 0.324301 1.785189

C -1.595550 -0.851293 1.025975

H -0.161007 1.839011 2.545857

H -2.559793 -1.357069 0.872280

C 2.209669 0.956530 1.434831

C 2.404060 2.272091 1.902864

C 3.702485 2.765892 2.036359

H 1.542200 2.908074 2.143671

C 4.527550 0.623493 1.235948

C 4.775174 1.925353 1.708728

H 5.814326 2.268853 1.814959

Ni 2.691124 -1.606192 0.163386

C 2.587213 -3.015690 1.650691

H 2.530094 -3.974419 1.101842

H 1.621288 -2.763866 2.129982

C 3.717458 -2.925005 2.571300

C 4.799323 -3.766718 2.442293

C 3.706787 -1.914001 3.696541

H 2.719548 -1.420467 3.783521

H 3.943674 -2.398714 4.666551

H 4.469377 -1.117656 3.558167

N 0.777154 -0.797120 0.592438

N 3.258547 0.153275 1.086892

C -0.501545 -2.656184 -0.359841

H -0.150815 -2.455616 -1.392447

H 0.171519 -3.427163 0.062593

H -1.531883 -3.055622 -0.398474

C 5.671701 -0.280914 0.878946

H 5.463755 -1.322771 1.193616

H 5.828642 -0.288766 -0.220388

H 6.612674 0.065689 1.346431

H 3.878548 3.791677 2.392914

H -2.405519 0.753875 2.260714

O 5.866365 -3.683301 3.276132

Si 7.250404 -4.721182 3.303461

C 8.224011 -4.489987 1.705555

H 8.363582 -3.418925 1.455507

H 9.227641 -4.957294 1.784252

H 7.689521 -4.994375 0.873248

C 8.190529 -4.099193 4.858002

C 9.486140 -4.917236 5.034431

H 10.045014 -4.568375 5.931643

H 9.283021 -5.999273 5.178402

H 10.169199 -4.815070 4.165205

C 7.293856 -4.271646 6.100843

H 7.813832 -3.895552 7.010592

H 6.343158 -3.709769 5.998681

H 7.038866 -5.336293 6.284947

C 8.538125 -2.607575 4.675025

H 7.626826 -1.993492 4.524717

H 9.065363 -2.217930 5.574904

H 9.206933 -2.440853 3.804631

C 6.672600 -6.511564 3.446523

H 6.242320 -6.841810 2.478354

H 7.528012 -7.182346 3.670383

H 5.912800 -6.643032 4.242752

H 4.826412 -4.545209 1.658942

C 2.957509 -2.849982 -1.305356

C 4.379384 -2.866491 -1.119170

C 2.307424 -4.079481 -1.648942

C 5.045542 -4.083858 -0.951646

H 4.950561 -1.930609 -1.154762

C 2.994062 -5.278451 -1.514198

H 1.255947 -4.083488 -1.964334

C 4.359334 -5.310492 -1.099245

H 6.123318 -4.100721 -0.731499

H 2.475972 -6.229867 -1.703682

Br 2.386867 -1.074787 -2.386755

C 5.081271 -6.561437 -0.811522

O 6.185963 -6.637609 -0.273626

O 4.379655 -7.674284 -1.179255

C 5.005993 -8.920883 -0.878666

H 4.350595 -9.704946 -1.299913

H 6.017970 -8.980485 -1.328623

H 5.111629 -9.063486 0.217436

**^2^B4_E_**

78

charge = 0; spin = 2

*E*_e_^S^: -5873.95262807

Zero-point correction=0.552968

Thermal correction to Energy=0.602504

Thermal correction to Enthalpy=0.603448

Thermal correction to Gibbs Free Energy=0.462834

C -0.238640 -1.490707 -0.193890

C 0.524798 0.258934 1.181864

C -0.789720 0.626550 1.524004

C -1.854183 -0.111329 0.998143

C -1.575649 -1.153179 0.107448

H -0.977412 1.487612 2.178884

H -2.388082 -1.714773 -0.375979

C 1.708070 1.073354 1.545879

C 1.628302 2.319586 2.195861

C 2.800293 3.059189 2.381220

H 0.661789 2.715574 2.533809

C 4.043457 1.286201 1.277402

C 4.014039 2.545659 1.906419

H 4.950561 3.109891 2.021130

Ni 2.829064 -1.279538 0.365238

C 2.820210 -2.023993 2.276153

H 2.278964 -2.978815 2.181480

H 2.185021 -1.281018 2.797728

C 4.149264 -2.148221 2.902559

C 4.892719 -3.290167 2.731319

C 4.663000 -1.057382 3.815310

H 4.251538 -0.065552 3.546591

H 4.356524 -1.252618 4.867355

H 5.769258 -0.998821 3.811262

N 0.783441 -0.826360 0.401155

N 2.898568 0.574471 1.125910

C 0.078499 -2.537676 -1.216897

H 0.872971 -2.159164 -1.893664

H 0.479737 -3.459193 -0.754533

H -0.824173 -2.801859 -1.798415

C 5.307363 0.689252 0.738404

H 5.457094 -0.337610 1.131115

H 5.215061 0.592917 -0.365460

H 6.188925 1.306914 0.990678

H 2.765883 4.037935 2.882676

H -2.892828 0.146537 1.253833

O 6.084238 -3.474035 3.340692

Si 7.130117 -4.846768 3.170068

C 8.047244 -4.700263 1.532205

H 8.536673 -3.711603 1.424661

H 8.828295 -5.483348 1.442406

H 7.347381 -4.834069 0.682371

C 8.286133 -4.684775 4.692925

C 9.294265 -5.852570 4.691891

H 9.978581 -5.772801 5.565967

H 8.792767 -6.840757 4.760901

H 9.928994 -5.858751 3.781103

C 7.441768 -4.724676 5.982904

H 8.094137 -4.605386 6.876809

H 6.689632 -3.909889 6.003190

H 6.901246 -5.687391 6.098254

C 9.044037 -3.343347 4.614976

H 8.347052 -2.480843 4.592618

H 9.704391 -3.220391 5.502583

H 9.689890 -3.280718 3.714495

C 6.081821 -6.416948 3.206064

H 5.423622 -6.480875 2.314844

H 6.734617 -7.314384 3.195927

H 5.443685 -6.467956 4.111166

H 4.526103 -4.105849 2.082139

C 3.299575 -3.080618 -0.195586

C 4.543338 -3.180322 -0.870719

C 2.662571 -4.297210 0.147463

C 5.139356 -4.421366 -1.130783

H 5.056079 -2.267895 -1.210034

C 3.239561 -5.546675 -0.123404

H 1.700353 -4.288927 0.687142

C 4.504194 -5.621336 -0.746320

H 6.110510 -4.486010 -1.645847

H 2.725595 -6.474648 0.167577

Br 3.073439 -0.337705 -1.923326

C 5.209005 -6.906032 -1.000755

O 6.310427 -7.015232 -1.518779

O 4.496481 -7.985107 -0.560759

C 5.124589 -9.251548 -0.766638

H 4.430658 -10.010150 -0.361255

H 5.308473 -9.435699 -1.845049

H 6.101008 -9.303387 -0.242087

**^2^[B4_E_-B1]^‡^**

78

charge = 0; spin = 2

*E*_e_^S^: -5873.93311500

Zero-point correction=0.551424

Thermal correction to Energy=0.600667

Thermal correction to Enthalpy=0.601611

Thermal correction to Gibbs Free Energy=0.461788

C 0.596096 -1.773827 -1.033862

C 0.289364 -0.222493 0.720714

C -1.102263 -0.428689 0.714928

C -1.647847 -1.352056 -0.181314

C -0.793104 -1.999061 -1.082428

H -1.753616 0.152681 1.380646

H -1.192070 -2.683160 -1.845353

C 0.944607 0.839863 1.509904

C 0.262717 1.608731 2.471825

C 0.928261 2.674081 3.083713

H -0.777738 1.377057 2.735945

C 2.895687 2.131554 1.754979

C 2.247576 2.948425 2.702771

H 2.792616 3.801057 3.133621

Ni 3.071712 -0.592620 0.185474

C 4.210694 -1.250410 1.937259

H 3.465613 -1.603390 2.669603

H 4.256896 -0.144065 1.934632

C 5.564707 -1.807711 2.118156

C 5.711236 -3.002644 2.765252

C 6.738986 -1.059826 1.546623

H 6.549996 -0.768148 0.490963

H 6.923471 -0.114186 2.103725

H 7.662865 -1.665780 1.593866

N 1.122505 -0.940187 -0.090620

N 2.251766 1.073737 1.188468

C 1.508494 -2.409002 -2.041135

H 2.337983 -1.721133 -2.302618

H 1.972113 -3.333530 -1.641740

H 0.941328 -2.675306 -2.953433

C 4.311689 2.420314 1.344126

H 4.557565 3.486165 1.511005

H 5.033155 1.821258 1.940895

H 4.475423 2.147720 0.281921

H 0.419826 3.292890 3.838051

H -2.732473 -1.534654 -0.205241

O 6.908435 -3.582102 2.992896

Si 7.257397 -5.247581 3.297871

C 7.874965 -5.997534 1.684954

H 8.679238 -5.379055 1.238453

H 8.280205 -7.017583 1.849668

H 7.055654 -6.081468 0.941575

C 8.616848 -5.183666 4.652093

C 9.054979 -6.618075 5.012213

H 9.847874 -6.595409 5.793005

H 8.217636 -7.224544 5.417315

H 9.474067 -7.160887 4.139205

C 8.053737 -4.479901 5.903367

H 8.836207 -4.404295 6.691288

H 7.710267 -3.450631 5.673129

H 7.198076 -5.032919 6.344594

C 9.825150 -4.388521 4.115264

H 9.539050 -3.357027 3.824967

H 10.615652 -4.312773 4.895268

H 10.283918 -4.874753 3.229289

C 5.684898 -6.120486 3.877711

H 4.925039 -6.148792 3.069291

H 5.915987 -7.171427 4.149410

H 5.231275 -5.635563 4.765691

H 4.818889 -3.549138 3.127318

C 3.603191 -2.469693 0.362413

C 4.631350 -2.985806 -0.474384

C 2.721848 -3.409756 0.966367

C 4.774452 -4.357741 -0.677533

H 5.316193 -2.287008 -0.976106

C 2.867820 -4.786880 0.770927

H 1.911631 -3.053781 1.623411

C 3.914102 -5.283573 -0.039083

H 5.568505 -4.750301 -1.331348

H 2.180448 -5.494114 1.257978

Br 4.467859 0.291145 -1.592564

C 4.172501 -6.732544 -0.224968

O 5.105534 -7.207575 -0.858198

O 3.257405 -7.513742 0.423973

C 3.465426 -8.921642 0.299464

H 2.658273 -9.405642 0.878845

H 3.420503 -9.240069 -0.762319

H 4.457312 -9.216459 0.699959

**^2^[B3_Z_-B4_Z_]^‡^**

78

charge = 0; spin = 2

*E*_e_^S^: -5873.89762903

Zero-point correction=0.550538

Thermal correction to Energy=0.599565

Thermal correction to Enthalpy=0.600509

Thermal correction to Gibbs Free Energy=0.462866

C -0.366150 -0.685826 0.433977

C 1.193918 0.866795 1.256909

C 0.176733 1.570990 1.939392

C -1.144712 1.141374 1.836077

C -1.418129 0.002665 1.061288

H 0.426461 2.440109 2.561929

H -2.447422 -0.364093 0.938090

C 2.603094 1.297684 1.310522

C 2.970383 2.578180 1.780721

C 4.315361 2.939970 1.811821

H 2.199513 3.293332 2.095787

C 4.852898 0.747468 0.912785

C 5.269199 2.007759 1.373183

H 6.341511 2.250638 1.380402

Ni 2.664391 -1.383284 0.179023

C 2.325326 -2.271520 2.006267

H 1.233941 -2.443140 2.022490

H 2.602484 -1.400296 2.633878

C 3.080086 -3.473872 2.345164

C 2.500873 -4.716835 2.386422

C 4.541178 -3.361162 2.706281

H 4.698259 -2.599413 3.500695

H 4.959111 -4.322659 3.065991

H 5.152567 -3.047141 1.833246

N 0.923409 -0.259032 0.527951

N 3.537853 0.395617 0.879624

C -0.638464 -1.922935 -0.365910

H -0.261255 -1.798873 -1.400972

H -0.100153 -2.786038 0.073706

H -1.719817 -2.151743 -0.397943

C 5.870707 -0.246867 0.434055

H 5.704070 -1.234105 0.907443

H 5.790200 -0.386642 -0.664269

H 6.898694 0.092370 0.659165

H 4.621036 3.935997 2.165171

H -1.951956 1.677042 2.357347

O 1.187736 -4.946333 2.112679

Si 0.330782 -6.344070 2.642957

C 0.245367 -6.296864 4.529778

H -0.291358 -5.397569 4.893208

H -0.264197 -7.193837 4.938099

H 1.270050 -6.270269 4.954604

C -1.396752 -6.178360 1.825575

C -2.283448 -7.356116 2.281782

H -3.293036 -7.280353 1.819509

H -1.863066 -8.339486 1.983460

H -2.429125 -7.370381 3.382313

C -1.244124 -6.207198 0.291262

H -2.237128 -6.094598 -0.199301

H -0.592410 -5.386031 -0.069873

H -0.807873 -7.163422 -0.065840

C -2.045359 -4.846940 2.256887

H -1.422735 -3.975138 1.971841

H -3.040179 -4.727683 1.771362

H -2.209166 -4.798921 3.353621

C 1.262412 -7.885743 2.073142

H 2.193190 -8.025775 2.660865

H 0.645689 -8.797345 2.214239

H 1.547660 -7.814073 1.004099

H 3.109271 -5.601609 2.658647

C 2.690387 -2.752537 -1.206930

C 4.078098 -2.969363 -0.917179

C 1.861244 -3.884126 -1.488611

C 4.547298 -4.273366 -0.693319

H 4.796307 -2.145972 -1.007477

C 2.357110 -5.164842 -1.291812

H 0.835290 -3.742716 -1.852219

C 3.699294 -5.383477 -0.864428

H 5.602942 -4.457896 -0.442217

H 1.708610 -6.033162 -1.475689

Br 2.438322 -1.060062 -2.433538

C 4.268815 -6.732463 -0.654231

O 5.415468 -6.972570 -0.301112

O 3.357696 -7.730151 -0.896450

C 3.862256 -9.056726 -0.734976

H 3.028999 -9.737372 -0.988232

H 4.726003 -9.238679 -1.406828

H 4.198673 -9.234046 0.307466

**^2^B4_Z_**

78

charge = 0; spin = 2

*E*_e_^S^: -5873.95041376

Zero-point correction=0.552947

Thermal correction to Energy=0.602228

Thermal correction to Enthalpy=0.603173

Thermal correction to Gibbs Free Energy=0.464533

C 0.762024 -1.180030 -0.095779

C 1.664453 0.638203 1.098122

C 0.386742 1.167637 1.353669

C -0.735033 0.473158 0.889981

C -0.540915 -0.681074 0.125505

H 0.271812 2.117576 1.891964

H -1.393607 -1.208378 -0.326434

C 2.915265 1.384309 1.370458

C 2.935215 2.714693 1.829119

C 4.164828 3.369487 1.947220

H 2.001545 3.237550 2.073728

C 5.262894 1.349114 1.156731

C 5.334941 2.685546 1.597616

H 6.317236 3.174731 1.664402

Ni 3.779564 -1.252719 0.698388

C 3.720346 -1.543832 2.751210

H 2.657710 -1.769225 2.957128

H 3.960033 -0.508297 3.063701

C 4.633714 -2.504027 3.398655

C 4.196241 -3.571456 4.132705

C 6.122315 -2.287148 3.287848

H 6.693593 -2.994429 3.921894

H 6.472353 -2.429640 2.241409

H 6.407913 -1.256359 3.593639

N 1.829253 -0.562023 0.474026

N 4.062581 0.724326 1.060984

C 0.983257 -2.340606 -1.018881

H 1.925299 -2.193229 -1.585532

H 1.090462 -3.296784 -0.470758

H 0.129713 -2.436135 -1.716473

C 6.487583 0.576885 0.775256

H 6.585565 -0.333268 1.400477

H 6.385990 0.223870 -0.273651

H 7.402678 1.186972 0.887180

H 4.209142 4.410503 2.300667

H -1.748543 0.854045 1.085779

O 2.892793 -3.880541 4.338091

Si 2.352145 -5.114579 5.421652

C 2.985502 -4.672862 7.148491

H 2.607775 -3.685764 7.483208

H 2.678595 -5.432288 7.896786

H 4.094123 -4.629203 7.156830

C 0.438410 -5.026038 5.315072

C -0.159025 -6.028869 6.325151

H -1.270845 -6.000222 6.284139

H 0.147004 -7.074011 6.109276

H 0.134576 -5.798629 7.370715

C -0.029083 -5.389600 3.891642

H -1.139404 -5.342735 3.827185

H 0.379307 -4.688403 3.136135

H 0.274589 -6.416256 3.599898

C -0.029324 -3.597544 5.661022

H 0.403686 -2.847519 4.968275

H -1.137970 -3.526470 5.589792

H 0.251489 -3.304015 6.694060

C 3.061044 -6.778260 4.888262

H 4.168045 -6.767830 4.967087

H 2.695634 -7.586507 5.555612

H 2.806056 -7.051464 3.844158

H 4.941943 -4.234555 4.615536

Br 4.388262 -0.969167 -1.678823

C 3.765986 -3.175231 0.634015

C 4.896402 -3.838355 0.097917

C 2.727414 -3.972853 1.164997

C 4.995320 -5.237951 0.121537

H 5.707454 -3.256305 -0.364870

C 2.808245 -5.368275 1.168618

H 1.844644 -3.499126 1.621155

C 3.953451 -6.019534 0.664797

H 5.885694 -5.736133 -0.289462

H 1.994046 -5.982926 1.580543

C 4.008790 -7.500954 0.761107

O 3.192856 -8.197981 1.351765

O 5.092041 -8.028499 0.124876

C 5.212353 -9.449494 0.202163

H 6.135171 -9.711374 -0.346610

H 5.281181 -9.788727 1.256414

H 4.336239 -9.950132 -0.259029

**^2^[B4_Z_-B1]^‡^**

78

charge = 0; spin = 2

*E*_e_^S^: -5873.93610581

Zero-point correction=0.551612

Thermal correction to Energy=0.600689

Thermal correction to Enthalpy=0.601633

Thermal correction to Gibbs Free Energy=0.462591

C 1.719297 -1.613223 -1.248237

C 1.521526 0.207539 0.241242

C 0.209026 0.471949 -0.190079

C -0.363844 -0.351261 -1.163936

C 0.413267 -1.374588 -1.719272

H -0.344939 1.332707 0.206865

H 0.022114 -2.000126 -2.534720

C 2.273206 1.112599 1.135663

C 1.679834 2.213301 1.782041

C 2.490856 3.091779 2.505430

H 0.596761 2.382158 1.716614

C 4.410298 1.718572 1.907488

C 3.869594 2.852560 2.546547

H 4.545644 3.541060 3.074372

Ni 4.006882 -1.155829 0.658033

C 4.233419 -1.815924 2.689794

H 3.234567 -1.804624 3.156215

H 4.675870 -0.799744 2.668319

C 5.176667 -2.740233 3.344237

C 4.750336 -3.702730 4.215823

C 6.649938 -2.573765 3.075678

H 7.252889 -3.360074 3.571868

H 6.871472 -2.596760 1.987418

H 7.017074 -1.588150 3.439698

N 2.226706 -0.864077 -0.227720

N 3.609201 0.849614 1.232811

C 2.581173 -2.659226 -1.891402

H 3.644627 -2.346367 -1.883449

H 2.521964 -3.623626 -1.347497

H 2.250001 -2.833841 -2.933089

C 5.887560 1.450384 1.955555

H 6.140618 0.758302 2.787737

H 6.227755 0.960328 1.020784

H 6.450319 2.387378 2.127939

H 2.054808 3.961444 3.019315

H -1.390176 -0.170057 -1.516301

O 3.451422 -3.897210 4.543449

Si 2.844560 -5.176559 5.536235

C 3.939627 -5.298912 7.073982

H 4.013830 -4.328878 7.605556

H 3.532713 -6.048658 7.783564

H 4.967716 -5.623503 6.812313

C 1.059584 -4.619555 5.962271

C 0.403595 -5.692040 6.857461

H -0.636576 -5.393375 7.117087

H 0.342347 -6.678539 6.352071

H 0.949256 -5.834105 7.813955

C 0.241301 -4.458740 4.664988

H -0.793219 -4.123401 4.902471

H 0.693755 -3.707027 3.987213

H 0.157930 -5.411642 4.102521

C 1.108085 -3.272030 6.711003

H 1.597940 -2.484480 6.102654

H 0.077603 -2.924271 6.948007

H 1.657225 -3.346851 7.672808

C 2.915145 -6.790178 4.566456

H 3.956431 -6.989712 4.238780

H 2.594350 -7.644426 5.198146

H 2.278174 -6.772610 3.659637

H 5.494705 -4.350974 4.718941

Br 6.056502 -1.061877 -0.654758

C 3.744338 -3.053948 1.039476

C 4.704505 -3.992743 0.576842

C 2.448100 -3.543339 1.354015

C 4.371397 -5.337607 0.395979

H 5.713732 -3.646033 0.312460

C 2.116204 -4.889727 1.185129

H 1.680513 -2.852975 1.738037

C 3.074247 -5.809784 0.707712

H 5.121170 -6.042893 0.008567

H 1.109151 -5.260874 1.429578

C 2.677011 -7.235640 0.592714

O 1.618176 -7.703917 0.991809

O 3.636303 -7.999924 -0.002219

C 3.322872 -9.388603 -0.118734

H 4.193714 -9.861582 -0.607754

H 3.146002 -9.843456 0.877774

H 2.409032 -9.542083 -0.728844

**^3^A2_E_-I**

60

charge = 0; spin = 3

*E*_e_^S^: -2840.45233209

Zero-point correction=0.582760

Thermal correction to Energy=0.630268

Thermal correction to Enthalpy=0.631212

Thermal correction to Gibbs Free Energy=0.495625

C -0.164679 -1.588046 -0.404418

C 0.764946 0.298630 0.694027

C -0.478307 0.974314 0.625136

C -1.574970 0.352718 0.045928

C -1.403331 -0.942139 -0.499285

H -0.558206 2.000669 1.009015

H -2.234719 -1.455512 -1.002949

C 1.977466 0.911826 1.183908

C 2.060681 2.215865 1.733249

C 3.304130 2.775067 1.999335

H 1.141569 2.784597 1.932314

C 4.346520 0.722153 1.195542

C 4.468552 2.028514 1.679687

H 5.469857 2.469435 1.788527

Ni 2.670236 -1.727443 0.746436

C 2.607973 -3.698555 0.511495

H 2.690725 -4.152980 -0.490285

H 1.759650 -4.078691 1.109082

C 3.821269 -3.317314 1.179396

C 3.589877 -2.618461 2.399338

C 5.195962 -3.600723 0.629945

H 5.465124 -4.659384 0.838146

H 5.227589 -3.465327 -0.469689

H 5.973004 -2.963101 1.089245

N 0.903950 -1.008264 0.224840

N 3.123079 0.140807 1.010947

C -0.002371 -2.952082 -1.016099

H 0.943090 -3.014554 -1.587967

H 0.037219 -3.745110 -0.242917

H -0.850623 -3.177659 -1.689684

C 5.559882 -0.079597 0.820004

H 5.722064 -0.898942 1.547016

H 5.419892 -0.546238 -0.176109

H 6.465161 0.556251 0.797332

H 3.385370 3.788412 2.419839

H -2.545645 0.866414 -0.018448

O 4.610032 -2.127702 3.156510

Si 4.411345 -1.606819 4.800647

C 3.692090 -3.060051 5.773037

H 4.357005 -3.946560 5.740269

H 3.536819 -2.785056 6.836550

H 2.705448 -3.361633 5.365254

C 6.187817 -1.169159 5.378276

C 6.140809 -0.836461 6.885531

H 7.157318 -0.568738 7.250723

H 5.479296 0.028058 7.104862

H 5.790860 -1.694773 7.496114

C 6.718404 0.052859 4.601720

H 7.742613 0.313274 4.951253

H 6.779203 -0.142848 3.512638

H 6.082006 0.950223 4.745606

C 7.118934 -2.375911 5.141346

H 7.159951 -2.656767 4.069472

H 8.154772 -2.134321 5.468828

H 6.796212 -3.271757 5.711822

C 3.225285 -0.144774 4.833614

H 2.218683 -0.451113 4.482525

H 3.113759 0.253887 5.863156

H 3.570843 0.676768 4.174399

H 2.628889 -2.785142 2.929453

**^3^[A2_E_-A2_Z_-I]^‡^**

79

charge = 0; spin = 3

*E*_e_^S^: -5650.31940376

Zero-point correction=0.582254

Thermal correction to Energy=0.628894

Thermal correction to Enthalpy=0.629839

Thermal correction to Gibbs Free Energy=0.499215

C 0.252434 -1.937726 -0.292025

C 0.507632 0.198687 0.696451

C -0.870595 0.419607 0.479742

C -1.709122 -0.530268 -0.134632

C -1.106849 -1.744987 -0.529262

H -1.318379 1.364236 0.787191

H -1.674316 -2.543702 -1.023598

C 1.527784 1.121003 1.276126

C 1.413010 2.451894 1.832283

C 2.617483 3.091573 2.181615

C 3.906691 1.175735 1.569389

C 3.873926 2.480904 2.038855

H 4.802051 3.007846 2.302176

Ni 2.899560 -1.343244 0.709160

Br 4.817540 -2.032965 -0.405757

C 1.171444 -4.058726 2.057795

H 0.295709 -3.404807 2.194906

H 0.981227 -5.106683 1.779110

C 2.447022 -3.595535 2.240812

C 2.685312 -2.178445 2.574974

C 3.641293 -4.509033 2.136202

H 4.174041 -4.561172 3.108700

H 3.337969 -5.532696 1.844696

H 4.371176 -4.121287 1.394736

N 1.039628 -1.013554 0.296039

N 2.778313 0.532525 1.216172

H 2.583232 4.109198 2.586853

O 3.858164 -1.897885 3.264656

Si 3.959350 -1.443326 4.916952

C 3.147617 -2.793151 5.963810

H 3.640602 -3.775707 5.821887

H 3.181951 -2.543119 7.044474

H 2.081448 -2.910012 5.679459

C 5.844972 -1.286146 5.235168

C 6.074934 -0.785066 6.676097

H 7.164731 -0.706095 6.888167

H 5.635978 0.220575 6.845864

H 5.645628 -1.473551 7.434516

C 6.457141 -0.290187 4.229218

H 7.551066 -0.186641 4.407069

H 6.317246 -0.634452 3.184377

H 6.012647 0.723451 4.320072

C 6.509201 -2.666070 5.049074

H 6.339516 -3.064940 4.028310

H 7.609077 -2.588263 5.200947

H 6.130737 -3.414010 5.776681

C 3.031754 0.183909 5.186599

H 1.966647 0.079705 4.891898

H 3.051002 0.475514 6.257103

H 3.465949 1.012472 4.592267

H 1.790786 -1.649975 2.972774

H 4.838845 0.601618 1.451547

H 0.753947 -2.874211 -0.581636

C -3.201891 -0.219437 -0.348875

C 0.095879 3.229061 2.089127

C -0.532768 3.655554 0.731416

H -1.572321 4.017631 0.874903

H 0.056045 4.491072 0.302570

H -0.544133 2.862447 -0.036287

C -3.935449 -1.384115 -1.040233

H -5.004898 -1.125801 -1.176131

H -3.516713 -1.600041 -2.043973

H -3.893342 -2.315448 -0.439914

C -3.341867 1.041652 -1.235381

H -4.413380 1.275860 -1.403902

H -2.876170 1.932670 -0.768600

H -2.864833 0.889505 -2.224749

C -3.871850 0.035713 1.022982

H -3.789716 -0.854375 1.679364

H -3.413725 0.892616 1.556638

H -4.949483 0.263792 0.888418

C 0.322958 4.553840 2.858760

H 0.797793 4.391403 3.847043

H 0.932501 5.282853 2.288826

H -0.662203 5.029502 3.039559

C -0.835612 2.397966 3.014830

H -0.413518 2.384426 4.040149

H -1.840739 2.865034 3.074034

H -0.964756 1.344389 2.714368

**lutidine**

17

*E*_e_^S^: -326.795027304

Zero-point correction=0.124241

Thermal correction to Energy=0.133027

Thermal correction to Enthalpy=0.133971

Thermal correction to Gibbs Free Energy=0.091455

C -3.216708 2.113285 -0.375929

C -1.242639 3.193271 0.220596

C -1.913523 4.424526 0.380094

C -3.293639 4.474811 0.145440

C -3.960809 3.304508 -0.238718

H -1.358082 5.325077 0.683249

H -3.846046 5.420538 0.261318

H -5.044511 3.308300 -0.431036

N -1.889801 2.071358 -0.148913

C 0.242243 3.062975 0.455333

H 0.744651 2.697866 -0.463849

H 0.705064 4.021543 0.757700

H 0.440653 2.306297 1.241847

C -3.866947 0.814798 -0.785918

H -3.701794 0.041487 -0.007923

H -4.955126 0.924989 -0.953859

H -3.399219 0.428836 -1.714817

**lutidine_C**

17

*E*_e_^S^: -326.547111758

Zero-point correction=0.122622

Thermal correction to Energy=0.131749

Thermal correction to Enthalpy=0.132693

Thermal correction to Gibbs Free Energy=0.089413

C -3.260703 2.065657 -0.303498

C -1.187518 3.222375 0.145413

C -1.891988 4.464283 0.252034

C -3.287676 4.463665 0.144084

C -3.992376 3.280837 -0.108788

H -1.327262 5.358820 0.557747

H -3.840944 5.410191 0.260176

H -5.075785 3.273718 -0.305619

N -1.954647 2.183003 -0.135621

C 0.268424 3.054167 0.453602

H 0.827218 3.011578 -0.508085

H 0.654187 3.907603 1.038527

H 0.459197 2.105363 0.990639

C -3.871094 0.786964 -0.788696

H -4.011779 0.112451 0.085448

H -4.859089 0.965093 -1.248567

H -3.204402 0.268695 -1.504178

**^3^A-NiBr_2_**

47

*E*_e_^S^: -7466.16912708

Zero-point correction=0.334621

Thermal correction to Energy=0.363368

Thermal correction to Enthalpy=0.364312

Thermal correction to Gibbs Free Energy=0.268762

C -1.167443 -0.150408 0.002154

C 0.732014 1.205494 0.000956

C -0.068222 2.358249 -0.000904

C -1.475080 2.265720 -0.001336

C -2.015326 0.962597 0.000265

H -1.564965 -1.177181 0.003668

H 0.417017 3.344116 -0.001971

H -3.099355 0.791664 0.000152

C 2.212265 1.203723 0.001689

C 3.015247 2.354566 0.000231

C 4.421880 2.258685 0.001105

H 2.532352 3.341583 -0.001642

C 4.108482 -0.156703 0.004638

C 4.959016 0.954279 0.003362

H 4.503555 -1.184419 0.006167

H 6.042635 0.780764 0.004096

Ni 1.468876 -1.527208 0.005136

Br 1.460554 -2.425473 2.163930

N 0.170488 -0.035188 0.002392

N 2.770828 -0.038296 0.003948

C -2.339336 3.537631 -0.003347

C 5.289149 3.528540 -0.000396

C -2.013957 4.366205 -1.269865

H -2.627366 5.290255 -1.287868

H -0.949235 4.672515 -1.306311

H -2.232791 3.791448 -2.192365

C -3.844295 3.211239 -0.003312

H -4.144628 2.636607 0.896126

H -4.429962 4.152118 -0.004766

H -4.144142 2.634136 -0.901328

C -2.014681 4.369749 1.261027

H -2.628135 5.293821 1.276106

H -2.234008 3.797565 2.185009

H -0.949994 4.676210 1.297213

C 4.966617 4.357844 -1.267157

H 3.902659 4.666707 -1.304353

H 5.582246 5.280422 -1.284763

H 5.184710 3.782534 -2.189488

C 4.965586 4.361454 1.263738

H 5.581193 5.284088 1.279218

H 3.901590 4.670396 1.299183

H 5.182938 3.788779 2.187882

C 6.793335 3.198596 0.000679

H 7.091718 2.623332 0.900362

H 7.092424 2.620718 -0.897092

H 7.381217 4.138094 -0.000451

Br 1.475022 -2.429734 -2.151995

**R_Z_**

33

*E*_e_^S^: -758.275016244

Zero-point correction=0.246920

Thermal correction to Energy=0.267091

Thermal correction to Enthalpy=0.268036

Thermal correction to Gibbs Free Energy=0.199357

Si -2.954942 0.919877 0.011664

C -3.257521 -0.971228 0.000469

C -2.603779 -1.595916 1.250582

H -2.738469 -2.700626 1.244804

H -1.515027 -1.388638 1.289275

H -3.054170 -1.216345 2.191427

C -4.775556 -1.246718 0.010343

H -5.285841 -0.822029 -0.879713

H -4.968651 -2.342653 0.004222

H -5.272051 -0.834515 0.913976

C -2.623583 -1.577852 -1.268607

H -2.758934 -2.682464 -1.276845

H -3.088215 -1.184430 -2.196761

H -1.535438 -1.370496 -1.321142

C -3.598041 1.766000 1.572880

H -3.169828 1.309296 2.487672

H -3.328314 2.842239 1.574750

H -4.703618 1.700810 1.639185

C -3.626183 1.788686 -1.525054

H -3.355097 2.864551 -1.516775

H -3.215897 1.344548 -2.454139

H -4.732897 1.725612 -1.571377

O -1.236465 1.042799 -0.002995

C -0.507238 2.181753 -0.001432

H -1.055352 3.143201 0.010687

C 0.897580 2.157234 -0.014725

C 1.618604 0.963581 -0.030164

H 2.718650 0.967503 -0.040502

H 1.101346 -0.006251 -0.032326

C 1.618776 3.491364 -0.011777

H 2.261832 3.603834 -0.909540

H 0.912511 4.345565 0.002043

H 2.280043 3.590023 0.874296

**[R_E_-R_Z_]^‡^**

33

*E*_e_^S^: -758.246937136

Zero-point correction= 0.246685

Thermal correction to Energy=0.266082

Thermal correction to Enthalpy=0.267026

Thermal correction to Gibbs Free Energy=0.200294

Si -2.949858 0.928120 -0.052256

C -3.223829 -0.946572 0.238202

C -2.632071 -1.332574 1.609595

H -2.743083 -2.426086 1.784813

H -1.552587 -1.086334 1.669966

H -3.144975 -0.813320 2.445922

C -4.734791 -1.255530 0.212250

H -5.198864 -1.000969 -0.763951

H -4.910964 -2.341041 0.383497

H -5.288733 -0.707182 1.003061

C -2.507187 -1.742527 -0.871877

H -2.625096 -2.836171 -0.701542

H -2.920637 -1.521585 -1.878161

H -1.420732 -1.520599 -0.894902

C -3.723660 2.010121 1.287294

H -3.389452 1.703755 2.298845

H -3.421672 3.067475 1.140069

H -4.831817 1.966657 1.260009

C -3.526849 1.496988 -1.760641

H -3.326456 2.578025 -1.909329

H -3.013456 0.941200 -2.570932

H -4.619654 1.345731 -1.880321

O -1.239063 1.091831 0.041056

C -0.544262 2.259357 -0.116514

H -0.975494 3.009637 -0.812453

C 0.933414 2.168578 0.028479

C 1.534802 2.470881 1.199354

H 2.628449 2.394622 1.322465

H 0.945781 2.803009 2.068409

C 1.703124 1.706317 -1.189879

H 1.357968 0.696502 -1.500089

H 1.517432 2.378946 -2.055215

H 2.795819 1.664061 -1.007875
